# Immunization with full-length *Plasmodium falciparum* merozoite surface protein 1 is safe and elicits functional cytophilic antibodies in a randomized first-in-human trial

**DOI:** 10.1038/s41541-020-0160-2

**Published:** 2020-01-31

**Authors:** Antje Blank, Kristin Fürle, Anja Jäschke, Gerd Mikus, Monika Lehmann, Johannes Hüsing, Kirsten Heiss, Thomas Giese, Darrick Carter, Ernst Böhnlein, Michael Lanzer, Walter E. Haefeli, Hermann Bujard

**Affiliations:** 1 Klinische Pharmakologie und Pharmakoepidemiologie, Universitätsklinikum Heidelberg, Im Neuenheimer Feld 410, 69120 Heidelberg, Germany; 2 Center of Infectious Diseases, Parasitology, Universitätsklinikum Heidelberg, Im Neuenheimer Feld 324, 69120 Heidelberg, Germany; 3 Koordinierungszentrum für Klinische Studien (KKS), Universitätsklinikum Heidelberg, Im Neuenheimer Feld 130.3, 69120 Heidelberg, Germany; 4 PEPperPRINT GmbH, Rischerstrasse 12, 69123 Heidelberg, Germany; 5 Institut für Immunologie, Universitätsklinikum Heidelberg und Deutsches Zentrum für Infektionsforschung (DZIF) Standort Heidelberg, Im Neuenheimer Feld 305, 69120 Heidelberg, Germany; 6 PAI Life Sciences, 1616 Eastlake Ave E, Suite 550, Seattle, WA 98102 USA; 7 Sumaya Biotech GmbH & Co. KG, Im Neuenheimer Feld 582, 69120 Heidelberg, Germany; 8 Zentrum für Molekulare Biologie Heidelberg, Universität Heidelberg, Im Neuenheimer Feld 282, 69120 Heidelberg, Germany

**Keywords:** Malaria, Protein vaccines

## Abstract

A vaccine remains a priority in the global fight against malaria. Here, we report on a single-center, randomized, double-blind, placebo and adjuvant-controlled, dose escalation phase 1a safety and immunogenicity clinical trial of full-length *Plasmodium falciparum* merozoite surface protein 1 (MSP1) in combination with GLA-SE adjuvant. Thirty-two healthy volunteers were vaccinated at least three times with MSP1 plus adjuvant, adjuvant alone, or placebo (24:4:4) to evaluate the safety and immunogenicity. MSP1 was safe, well tolerated and immunogenic, with all vaccinees sero-converting independent of the dose. The MSP1-specific IgG and IgM titers persisted above levels found in malaria semi-immune humans for at least 6 months after the last immunization. The antibodies were variant- and strain-transcending and stimulated respiratory activity in granulocytes. Furthermore, full-length MSP1 induced memory T-cells. Our findings encourage challenge studies as the next step to evaluate the efficacy of full-length MSP1 as a vaccine candidate against falciparum malaria (EudraCT 2016-002463-33).

## Introduction

In recent years, progress in global malaria control has stalled at ~219 million clinical cases and 435,000 deaths annually following a decade of decreasing disease burden.^1^ Apparently, the current intervention regimens, i.e., vector control and optimized drug treatment strategies,^2,3^ are insufficient to achieve a sustainable, steady reduction in malaria incidence, and eventually an elimination of this infectious disease. A key priority is therefore the development of a long-lasting, effective vaccine with a primary focus on the virulent and deadly form of malaria caused by the protozoan parasite *Plasmodium falciparum*.^4,5^

Hopes for a malaria vaccine have come from immuno-epidemiological studies, showing that people living in endemic areas can acquire immunity to clinical disease with time and age after repeated episodes of infection with *P. falciparum* and the attainment of a strain-transcending antigenic memory.^6^ A critical component of this immunity are antibodies as convincingly demonstrated in passive immunization studies in which IgG from malaria-immune adults were transfused to juvenile malaria patients and drastically reduced blood stage parasitemia.^7^ Although great effort has been invested in the identification of protective antigens, previous vaccination strategies have generally been unsatisfactory and only the pre-erythrocytic vaccine RTS,S (Mosquirix^TM^, GSK Bio), based on the *P. falciparum* circumsporozoite antigen, is under pilot implementation studies in three African countries.^8–11^ Nonetheless, its efficacy is moderate and short-lived (39% reduction in overall malaria incidence and 31.5% in life-threatening complications over a follow-up period of 48 months in children who received four injections^12,13^), possibly due to a decay in complement-fixing antibodies.^14^

An antigen that has been widely considered as a component of a malaria vaccine is the *P. falciparum* merozoite surface protein 1 (MSP1). MSP1 plays an essential role during blood-stage development of the parasite. It is synthesized as a precursor of ~196 kDa, which is processed into four subunits by a subtilisin-like protease shortly before the infected erythrocyte ruptures at the end of the 48 h replicative cycle to release merozoites.^15^ The MSP1 subunits remain non-covalently attached in a complex anchored to the parasite plasma membrane via a GPI anchor. Processing of MSP1 activates a spectrin-binding function of MSP1, which, in turn, promotes red blood cell rupture by destabilizing the membrane skeleton of the host erythrocytes.^16^ Other studies have shown that the MSP1 complex recruits variable peripheral proteins and that the ensuing supermolecular complex interacts with ligands on the red blood cell surface during invasion.^17–22^ Much of MSP1 is shed from the merozoite surface as the parasite invades, leaving only the GPI-anchored p19 fragment attached to the invading parasite.^23^ MSP1 is also presented on the nascent merozoites during pre-erythrocytic liver stage development of *P. falciparum*.^24,25^

MSP1 elicits a humoral immune response in natural infections and some — but not all — studies have found a correlation between MSP1 antibody titers and protection from clinical malaria.^26–30^ Antibodies against MSP1 can induce multiple effector mechanisms to control blood stage parasitemia, including directly blocking invasion of erythrocytes by merozoites and/or intraerythrocytic development,^23,31–34^ activation of complement,^35^ and opsonization for monocyte-mediated phagocytosis and neutrophil granulocyte-mediated release of reactive oxygen species (ROS).^36,37^ MSP1 can further elicit CD8^+^ T-cell-mediated cellular immune responses against liver stage parasites.^38–40^ In animal studies, a MSP1 vaccine has shown efficacy,^41–45^ but human immunization trials using MSP1 were disappointing and could not demonstrate protection against malaria.^46,47^ However — in contrast to the successful animal studies — clinical trials in humans only used small fragments of MSP1, such as the p19 or the p42 fragment, while largely ignoring the remaining ~80% of the protein, which contains numerous T-cell and B-cell epitopes that likely contribute to the MSP1-elicited immune responses.^32,40,48–52^

Using full-length MSP1 in clinical trials has been hampered by the sheer size of the protein of 196 kDa and problems in producing it in heterologous systems. We have succeeded in producing the entire MSP1 in *E. coli* and isolating it to >99% purity under good manufacturing practice (GMP) conditions.^53^ This GMP material passed all regulatory preclinical tests without showing any signs of toxicity. We therefore conducted a phase 1 first-in-human study to assess the safety and immunogenicity of full-length MSP1. Our data show that full-length MSP1 is safe and immunogenic. All vaccinees sero-converted and produced high MSP1-specific antibody titers. The induced MSP1-specific antibodies activated the complement system and also opsonized merozoites and activated human neutrophil granulocytes to release a respiratory burst in vitro. Furthermore, vaccination with full-length MSP1 induced IFN-γ producing memory T-cells.

## Results

### Full-length MSP1 in combination with GLA-SE is safe

Between April 2017 and November 2018, 32 healthy volunteers (19 females) were recruited in a double-blind dose-escalation, placebo, and adjuvant-controlled first-in-human phase 1 clinical trial to assess the safety and immunogenicity of SumayaVAC-1, a combination of full-length MSP1 and GLA-SE as adjuvant. GLA-SE is a stable oil-in-water nanoemulsion of the TLR4 agonist glucopyranosyl lipid A. GLA-SE was chosen as an adjuvant due to its favorable safety record^54–56^ and because it stimulates Th1 CD4^+^ T-cell responses to co-administered antigens,^57^ a feature we consider important since CD4^+^ T-cells contribute via their helper and effector functions to protective immunity to blood stage malaria infection.^58^

**Fig. 1:**
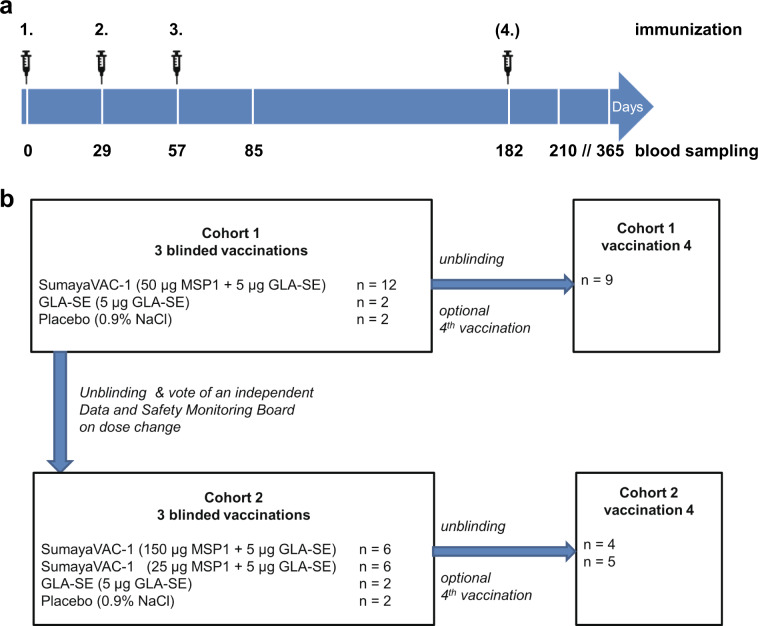
Immunization scheme and study design. **a** Volunteers were immunized with SumayaVAC-1 (full length MSP1 plus GLA-SE as adjuvant, 500 µl volume) on days 0, 29, 57, and optionally after unblinding of the cohort on day 182. Blood samples were taken for serological analysis on the days indicated. The safety follow-up was scheduled 6 months after the last vaccination. **b** 32 healthy volunteers were recruited in two consecutive cohorts. Sixteen volunteers each were randomly assigned within a cohort. The number of volunteers receiving three or four vaccinations is indicated for each cohort.

None of the volunteers had a known prior malaria infection or had been vaccinated against malaria before. Median age of the vaccinated population was 27.5 years (range 19–57). The majority (81%) was of White-Caucasian ethnic background. The mean body mass index at screening was comparable between groups and showed a range of means between 22.9 and 26.1 kg m^−2^ (range 17.1–33.0 kg m^−2^). Trial design and participant disposition are displayed in Fig. 1a, b. The vaccination was generally well tolerated. There were no serious adverse events, no dose-limiting toxicities, and no events resulting in permanent disability or premature withdrawal from the study (Table 1). There was further no pattern of adverse events, suggestive of off-target effects. A total of 562 adverse events occurred in the 32 volunteers (480 in 32 volunteers vaccinated three times and 82 in the 18 volunteers who opted for a fourth vaccination), with local injection site reactions exhibiting the highest frequency. A complete list of adverse events (assigned according to MedDRA High Level Coded Terms) can be found in the Supplementary Table 1.

**Table 1:** Distribution of solicited and unsolicited adverse reactions and distribution of all assessed adverse events, grouped by blinded vaccination 1–3 and the optional 4th booster vaccination.

MSP1	Vaccination 1–3 (blinded)					Vaccination 4 (optional—open)		
	SumayaVac-1			GLA-SE	0.9% NaCl	SumayaVac-1		
	25 µg	50 µg	150 µg	5 µg	1 ml	25 µg	50 µg	150 µg
	*N* = 6	*N* = 12	*N* = 6	*N* = 4	*N* = 4	*N* = 5	*N* = 9	*N* = 4
Solicited systemic reaction	7 (2)	15 (7)	8 (3)	1 (1)	2 (1)	0 (0)	4 (1)	0 (0)
Immediate	1 (1)	2 (1)	1 (1)	0 (0)	0 (0)	0 (0)	0 (0)	0 (0)
Delayed	6 (3)	13 (7)	7 (3)	1 (1)	2 (1)	0 (0)	4 (1)	0 (0)
Solicited local reaction	15 (5)	58 (12)	12 (4)	10 (4)	11 (3)	3 (3)	8 (5)	3 (3)
Immediate	5 (3)	4 (4)	1 (1)	1 (1)	3 (2)	0 (0)	0 (0)	1 (1)
Delayed	10 (3)	54 (12)	11 (4)	9 (4)	8 (4)	3 (3)	8 (5)	2 (2)
Unsolicited adverse events	63 (6)	147 (12)	55 (6)	43 (4)	33 (33)	19 (5)	30 (9)	15 (4)
All adverse events	85 (6)	220 (12)	75 (6)	54 (4)	46 (4)	22 (5)	42 (9)	18 (4)
Serious	0 (0)	0 (0)	0 (0)	0 (0)	0 (0)	0 (0)	0 (0)	0 (0)
Permanent	0 (0)	1 (1)	1 (1)	0 (0)	0 (0)	0 (0)	0 (0)	0 (0)
Mild/moderate/severe	73/12/0	209/10/1	72/3/0	51/3/0	46/0/0	17/5/0	38/4/0	17/0/1
Related/unrelated	54/31	160/60	55/20	33/21	35/11	15/7	33/9	14/4

Please observe different numbers of volunteers (*N*) in each group. The figures in cells (except bottom two lines) represent the number of events followed by the number of participants in brackets. The original clinical data underpinning this table are compiled in the Supplementary Table 3.

Solicited systemic reaction to the vaccine: solicited predefined systemic symptoms, at least possibly related.

Solicited local reaction to the vaccine: solicited predefined local symptoms, at least possibly related.

Immediate: occurring within 30 min.

Delayed: occurring between >30 min and 29 days.

Laboratory values did not show any clinically relevant pattern of change except for an increase in C-reactive protein (CRP, a marker for systemic inflammation) in the first days after each vaccination. In all vaccination observation cycles, 93% of adverse events were of mild nature and transient. Only two severe events occurred, which were assessed as being not related to the vaccination. One event was a hypertension grade III (according to the Common Terminology Criteria for Adverse Events 4.0) prior to vaccination, which resolved without treatment and was attributed to the tension prior to the application of the study drug. The other was a tendon rupture occurring 50 days after the last vaccination. Overall, tolerability was comparable in all dose groups and the GLA-SE group, and the vaccine did not show any dose-dependent adverse effects. In the post-trial follow up, one patient showed a moderate event of a transient inflammatory disease, and one severe event occurred, which was, however, an unrelated accidental injury from sports. All other post trial contacts did not reveal any clinically relevant adverse events.

### Full-length MSP1 elicits a humoral immune response

**Fig. 2:**
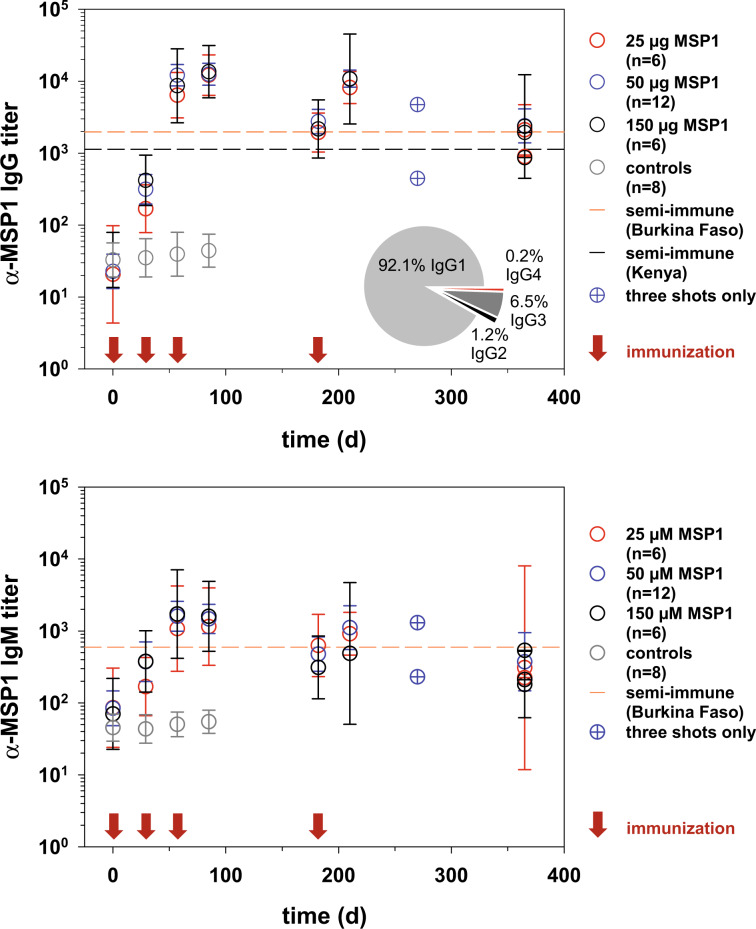
IgG and IgM antibody titers against MSP1-D after immunization with SumayaVac-1. Blood samples were collected from the vaccines on the days indicated and the **a** IgG-specific and **b** IgM-specific MSP1-D antibody titers were determined by ELISA. The data points represent the geometric means ± 95% confidence interval of each cohort. Crossed circles indicate samples from single individuals. Red arrows indicate the days of immunization. Dashed lines indicate reference titers obtained from pooled sera from semi-immune individuals from Nouna, Burkina Faso (orange line, *n* = 11)^48^ and from Kisumu, Kenya (black line, NIBSC code 10/198).^104^ Controls comprise the placebo and the GLA-SE vaccinees. The pie chart shows the average percentile distribution of IgG subclasses after three immunizations with SumayaVac-1 (day 85, *n* = 24).

All vaccinees who received SumayaVac-1 seroconverted irrespective of the dose, whereas none of the placebo or GLA-SE alone recipients did (Fig. 2a, b). Seroconversion was defined as an increase in MSP1-specific antibody titer, as compared with the baseline value, and involved the immunoglobulin classes IgG and IgM. The MSP1-specific IgG and IgM antibody titers peaked around day 85, four weeks after the third immunization, exceeding the titers found in semi-immune individuals from malaria endemic areas in Burkina Faso and Kenya (Fig. 2a, b). The fourth immunization at day 182 boosted declining MSP1 antibody titers (the peaks after the 3rd and 4th vaccination were not statistically different from one another, with *p* > 0.1, according to Kruskal–Wallis one-way ANOVA on ranks). Following the 4th immunization, specific IgG antibodies persisted for at least 6 months at levels seen in malaria semi-immune individuals. Long-lasting MSP1-specific antibody titers were also observed for IgM (Fig. 2b). In general, we noted comparable induction and decay kinetics for both IgG and IgM titers (Fig. 2a, b). There were no significant differences between the three dose levels regarding the IgG or IgM titers or their time courses (*p* = 0.3 according to Kruskal–Wallis one-way ANOVA on ranks) (Fig. 2a, b). However, participants who received only three immunizations instead of four generally had lower MSP1-specific IgM and IgG levels 12 months after the first immunization (Fig. 2a. b). Subtyping the MSP1-specific IgG antibodies revealed induction of all four IgG subclasses in vaccinees, with IgG1 being the predominant isotype, accounting for 92.1% of total MSP1-specific IgG antibodies, followed by IgG3 with 6.5% (Fig. 2a).

**Fig. 3:**
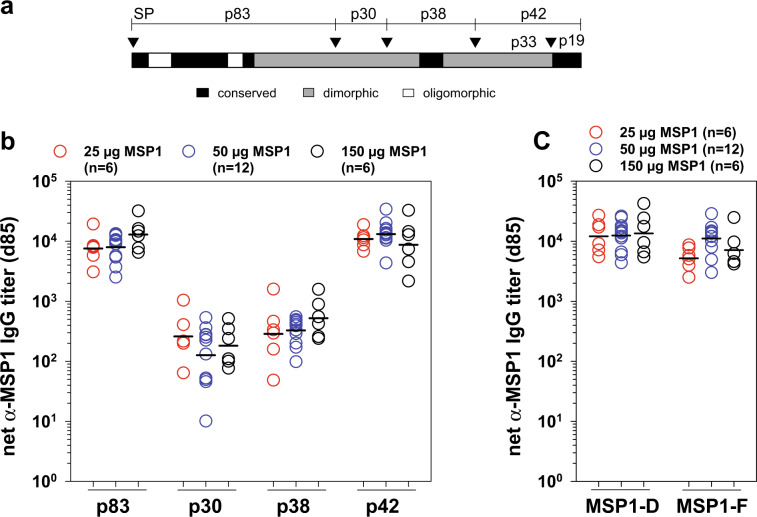
IgG responses against MSP1-D sub-fragments and cross-reactivity against MSP1-F. **a** Structural organization of MSP1. MSP1 is synthesized as a precursor protein of 196 kDa. During merozoite maturation, MSP1 is cleaved by PfSub1 into four major MSP1 fragments, termed p83, p30, p38, and p42. The cleavage sites are indicated by arrowheads. Conserved, dimorphic and oligomorphic domains of MSP1 are indicated. **b** The net α-MSP1-D titers of the vaccinees on day 85 are shown for the four major MSP1 fragments. Each data point represents the result (performed in duplicates) from one individual. Black marks indicate the mean value of the corresponding cohort. **c** Antibody titers against MSP1-D and MSP1-F after three immunizations on day 85.

All processing fragments of MSP1 reacted with the antibodies, as determined using subunit-specific ELISAs (Fig. 3a, b), consistent with previous studies using sera from semi-immune individuals or MSP1-vaccinated animals.^32^ There were no statistical differences between the dosing groups regarding the reactivity of the different fragments (*p* = 0.4, according to Kruskal–Wallis one-way ANOVA on ranks).

MSP1 is a large dimorphic protein, well represented by the two prototypic sequences MSP1-D from the MAD20 strain and MSP1-F from the WELLCOME strain. Both forms are present in African populations, with different distributions between East and West Africa.^59,60^ We therefore assessed whether human antibodies generated against MSP1-D used in this clinical trial cross-reacted with MSP1-F. This was indeed the case. Both forms reacted equally well with the human IgG antibodies (*p* = 0.054 according to Kruskal–Wallis one-way ANOVA on ranks) (Fig. 3c).

**Fig. 4:**
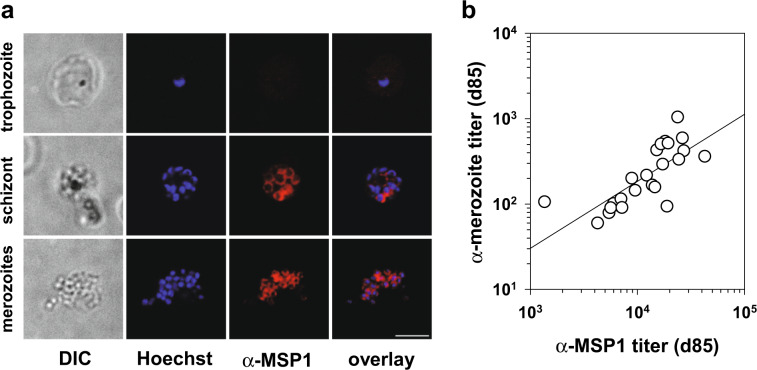
Recognition of native MSP1-D by induced antibodies. **a** Representative indirect immuno-fluorescence assay (IFA) showing reactivity of serum from an individual immunized 3× with SumayaVac-1 with developing merozoites during schizogony and free mature merozoites. Trophozoites served as a negative control. Trophozoite and schizont samples were fixed for analysis, whereas merozoites were unfixed. Scale bar, 5 µm. **b** Correlation between the α-merozoite titer and the α-MSP1-D IgG titer for all volunteers vaccinated 3x with SumayaVac-1 (day 85, *n* = 24). Each data point represents the results from one vaccinee. Titers were determined by quantitative ELISAs, whereby one set of plates was coated with purified full-length MSP1 and the other with purified *P. falciparum* merozoites. The correlation between two parameters was assessed using Pearson product moment correlation (correlation coefficient, 0.624; *p* = 0.0011).

We next investigated whether the induced antibodies can recognize native MSP1 on the merozoite surface. Sera from vaccinees collected at day 85 recognized MSP1 both in schizonts and on the surface of free, unfixed merozoites as shown by immune fluorescence (Fig. 4a). Moreover, the antibody titers as determined using a whole cell merozoite ELISA directly correlated with the titers obtained using the MSP1 protein ELISA (Fig. 4b) (correlation coefficient, 0.624; *p* = 0.0011, according to Pearson product moment correlation).

### MSP1 antibodies have no direct growth inhibitory effect in vitro

**Fig. 5:**
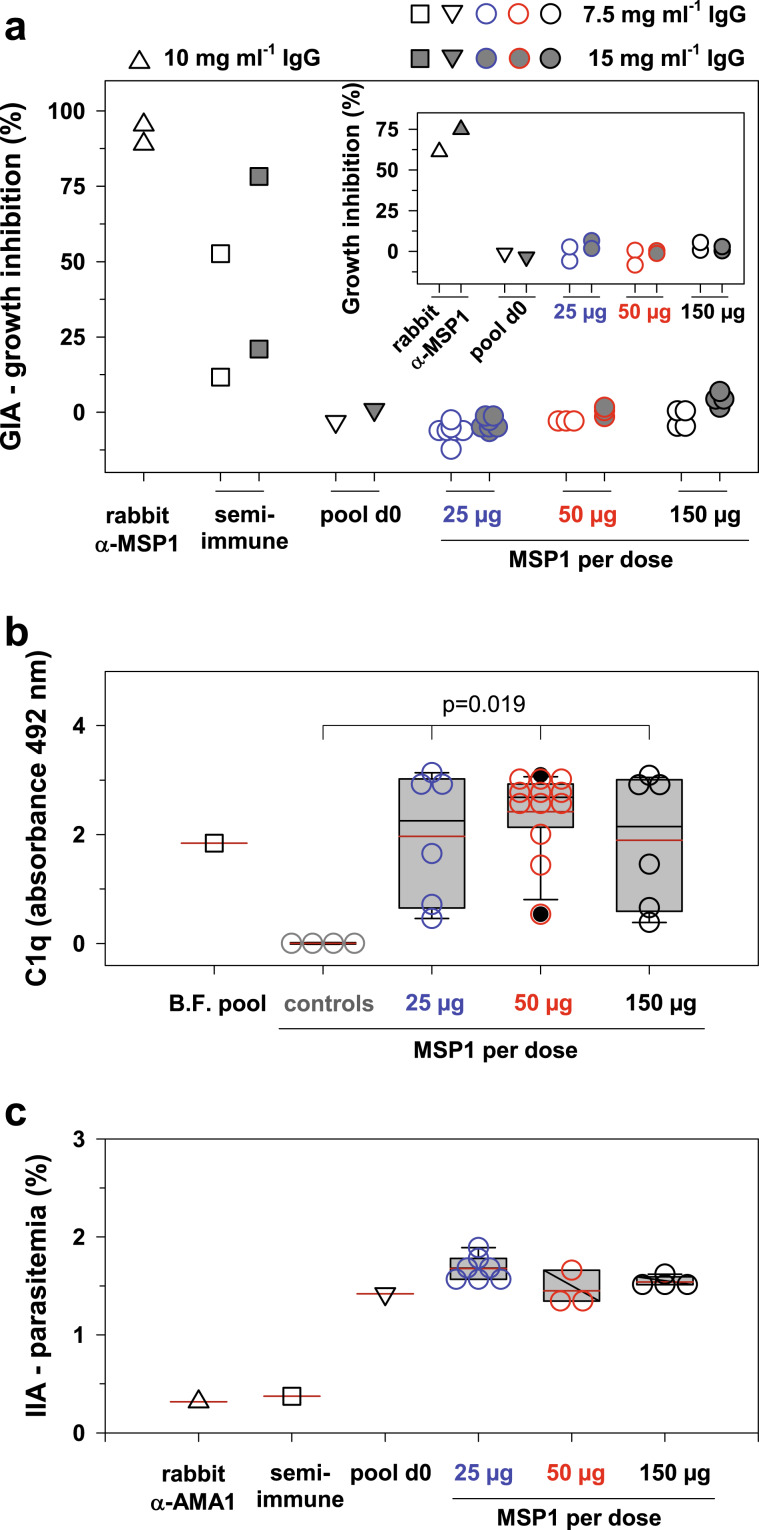
Effect of induced α-MSP1 antibodies on parasite growth inhibition, complement activation, and invasion inhibition. **a** Growth inhibition assay (GIA). The *P. falciparum* strain 3D7 was cultured for one cycle in the presence of total IgGs from volunteers vaccinated 3x with SumayaVac-1 (day 85; *n* = 14, circles). Two IgG preparations each from MSP1-immunized rabbits (triangles) and semi-immune adults from Burkina Faso (squares) served as positive controls, and pooled IgGs from volunteers collected on day 0 (inverted triangles) as negative controls. The concentrations of purified total IgG antibodies used in the assay are indicated. Inset: GIA activity in the presence of active complement. **b** Sera from volunteers vaccinated 3x with SumayaVac-1 (day 85; *n* = 24) fix complement in the presence of purified MSP1, as determined using a C1q-specific ELISA. The readout is the absorbance at a wavelength of 492 nm. A serum pool from semi-immune adults from Burkina Faso (*n* = 11) served as a positive control and the sera from the placebo and GLA-SE vacinees as negative control. Each data point represents the result (performed in duplicates) from one individual. A box plot analysis is overlaid over the individual data points with the median (black line), mean (red lines), and 25% and 75% quartile ranges being shown. The error bars above and below the box indicate the 90th and 10th percentiles. Filled black circles indicated outliers. Statistical significance was assessed using one way ANOVA. **c** Direct invasion assay (IIA). Purified merozoites (3D7) were incubated with erythrocytes in the presence of 3 mg ml^−1^ purified IgG for 30 min before the number of invaded erythrocyte was determined and the parasitemia calculated. IgG preparations from rabbits immunized with apical membrane antigen 1 (AMA-1)^105^ and a semi-immune adult from Burkina Faso served as positive controls and an IgG pool from the placebo cohort as negative control. Each data point represents the result (performed in duplicates) from one individual. Box plot analysis as described above.

Having shown that the human antibodies can recognize native MSP1 on the merozoite surface, we next investigated whether these antibodies exert a functional activity, e.g., by inhibiting parasite growth, opsonizing merozoites, and/or stimulating effector immune responses. We tested purified IgG collected at day 85 from a representative number of 14 MSP1 immunized volunteers in the standard growth inhibition assay (GIA). To this end, highly synchronized parasites at the schizont stage (38–42 h post invasion) were cultured in media containing 7.5 or 15 mg ml^−1^ purified IgG, respectively, for one cycle before parasite growth was determined. No GIA activity was observed (Fig. 5a). In comparison, α-MSP1 IgG from rabbits and malaria semi-immune adults from Burkina Faso strongly inhibited parasite growth by 95% and 75%, respectively (Fig. 5a).

Boyle et al. (2015) have shown that human anti-merozoite antibodies require active complement and complement fixation via the classical pathway for potent growth inhibition.^35^ To explore this possibility, we repeated the GIA assays in the presence of active complement. However, the antibodies from the vaccinees remained neutral in the GIA assay (Fig. 5a inset), in spite of the fact that they activated complement via the classical pathway in the presence of MSP1, as shown using a semi-quantitative C1q ELISA (Fig. 5b).

We further investigated the direct effect of IgG from the vaccinees in the invasion inhibition assay (IIA),^61^ where merozoites were incubated with 3 mg ml^−1^ purified IgG (from day 85) and uninfected red blood cells for 30 min before the cells were washed and then returned to culture for 40 h. No growth inhibitory effect was observed for any of the sera from the vaccinees (Fig. 5c). In comparison, the two positive controls, a rabbit α-apical membrane antigen 1 (AMA1) IgG pool and total IgG from a malaria semi-immune adult,^32^ reduced invasion efficiency by approximated 80% (Fig. 5c).

### Fine scale mapping of MSP1 B-cell epitopes

**Fig. 6:**
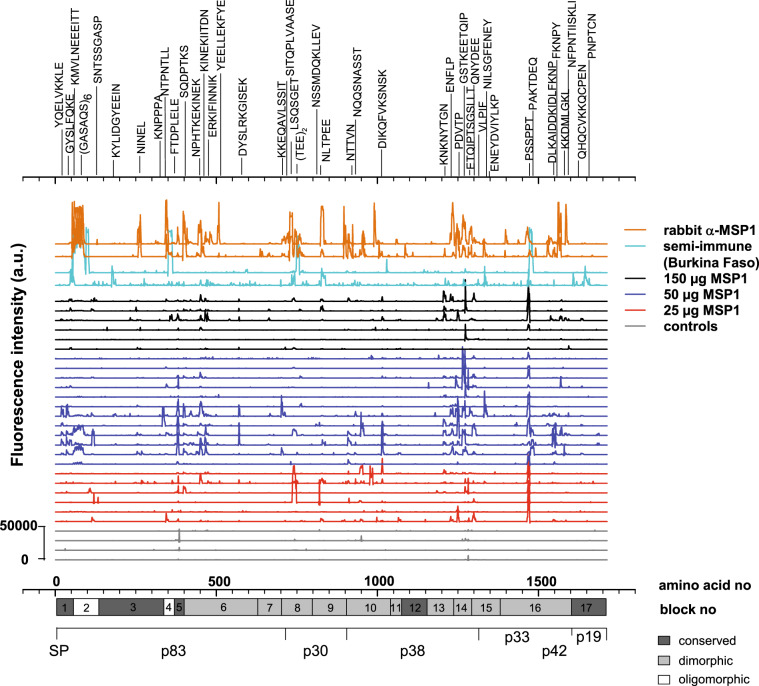
Mapping of linear B-cell epitopes across MSP1. Sera from vaccinees (1:1000), two malaria semi-immune adults from Burkina Faso (1:1000), and two MSP1 immunized rabbits (1:10,000) were applied to a custom-made MSP1 peptide microarray chip consisting of 1706 oligomeric peptides of 15 amino acids with a peptide-to-peptide overlap of 14 amino acids. Each peptide was printed in duplicate on the chip. As secondary antibodies the appropriate DyLight680 conjugated anti-IgG (Fc) antibodies were used. The fluorescence intensity landscapes across MSP1 are shown for each sample. Relevant epitopes have been highlighted. Controls comprise the placebo and the GLA-SE vaccinees. For orientation, the structural organization of MSP1 is shown below the fluorescence profiles. Full details of epitopes and fluorescence intensities are provided in the Supplementary Table 2.

In an effort to better characterize the MSP1-specific antibody responses from vaccinees, we performed a comparative high-resolution mapping of linear B-cell epitopes, using an MSP1 peptide chip consisting of 1706 15mer oligopeptides with a neighbor-to-neighbor overlap of 14 amino acids. In parallel assays, we investigated two sera each from MSP1-immunized rabbits and from malaria semi-immune adults. As seen in Fig. 6, MSP1 harbors numerous B-cell epitopes, spread over the entire protein, which elicited an IgG antibody response in MSP1-immunized volunteers, malaria semi-immune adults and rabbits. Dominant cross-species epitopes included low complexity sequences, such as (GASAQS)_6_ (position 62–97) and (TEE)_2_ (position 748–753) but also epitopes in conserved (NINEL, position 266–270; and FTDPLELE, position 384–391) and dimorphic MSP1 domains (GSTKEETQIP, position 1269–1278; and PSSPPT, position 1474–1479) (Fig. 6 and Supplementary Table 2). A comparative analysis of the epitope landscapes revealed a trend for a higher reactivity of sera from volunteers who had been vaccinated with 50 µg of MSP1 than with 25 or 150 µg (Fig. 6).

**Fig. 7:**
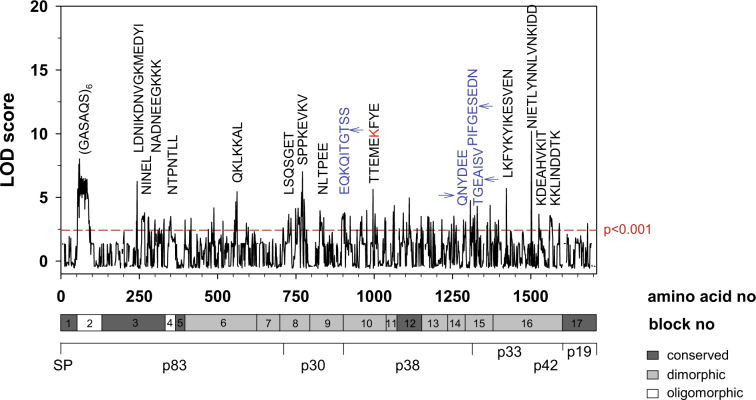
Association of GIA activity with MSP1 epitopes. Major epitopes putatively associated with GIA activity as defined by LOD scores > 2.5 (<*p* = 0.001) for at least three consecutive peptides are highlighted. The GIA activity underpinning the correlation are depicted in Fig. 5a, with sera from MSP1-immunized rabbits and semi-immune adults from Burkina Faso showing GIA activity, whereas sera from vaccinees being GIA inactive. Epitopes that map to MSP1 processing sites^16,83^ are indicated in blue, with the cleavage site being pointed out by an arrow. Note that three processing sites were detected between the p38/p42 junction: the canonical cleavage site (TGEAISV) and two adjacent alternative sites. Another epitope includes a lysine (highlighted in red) that is posttranslationally modified by acetylation. The correlation was determined using a Pearson function followed by a Bonferroni correction and conversion of the resulting *p*-values into a LOD score (logarithm of odds). The confidence line as defined by *p* < 0.001 is indicated. For orientation, the structural organization of MSP1 is shown below the graph. Full details of epitopes and LOD scores are provided in the Supplementary Table 2.

To identify B-cell epitopes possibly playing a role in inhibiting parasite growth, we performed a global, cross-species correlation between the IgG responses and GIA activity. This analysis revealed several candidate epitopes with high correlation scores, including (GASAQS)_6_ (position 62–97), NTPNTLL (position 350–356), LSQSGET (position 736–742), EQKQITGTSS (position 903–912), TTEMEKFYE (position 996–1004), QNYDEE (position 1294–1299), PIFGESEDN (position 1307–1315), and TGEAISV (position 1324– 1330) (Fig. 7).

### MSP1 antibodies stimulate polymorphonuclear neutrophil (PMN) granulocytes

**Fig. 8:**
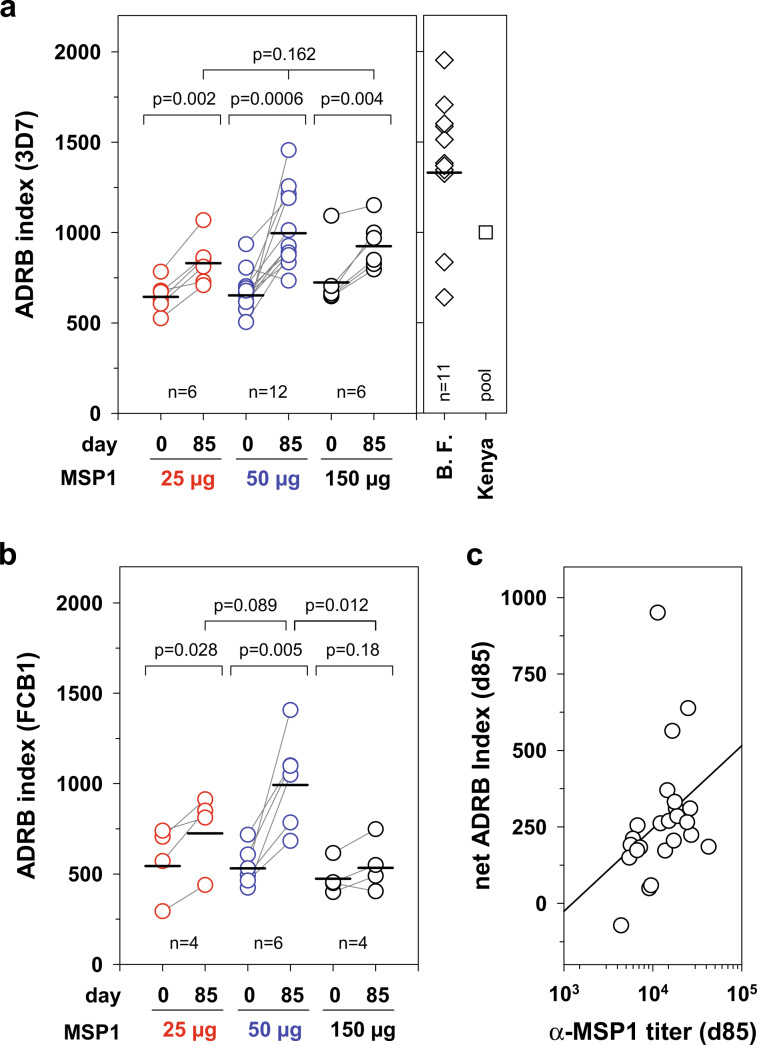
Activation of neutrophils by IgG from volunteers immunized with SumayaVac-1. *P. falciparum* merozoites from the **a** 3D7 strain expressing MSP1-D or the **b** FCB1 strain expressing MSP1-F and neutrophils were incubated in the presence of purified IgG from volunteers immunized three times with SumayaVac-1 (collected at days 0 and 85). Opsonized merozoites activated neutrophils to produce a respiratory burst, which was quantitatively expressed in terms of an ADRB index. Each data point represents the mean ADRB Index of at least two independent experiments each performed in duplicates from one volunteer. Black marks indicate the geometric mean values of the corresponding cohort. Statistical significance was assessed using paired *t*-test (between day 0 and day 85) and the Kruskal–Wallis one-way ANOVA on ranks (between different dosing groups). ADRB activities for purified IgG from semi-immune adults from Nouna, Burkina Faso (*n* = 11), and from Kenya (pooled IgG) are indicated as reference. **c** Correlation between the net ADRB index (corrected for the background activity at day 0) and the α-MSP1-D titer (at day 85) (correlation coefficient, 0.545; *p* = 0.006, according to Spearman rank order correlation). Each data point represents the results from one volunteer (*n* = 24).

We next assessed the ability of IgG from vaccinees to stimulate immune effector cells upon opsonization of merozoites in the antibody-dependent respiratory burst (ADRB) assay.^36,62^ ADRB activity has been associated with clinical protection from malaria in several studies.^62–64^ As seen in Fig. 8a, b, IgG from MSP1 vaccinees stimulated neutrophils to produce ROS in the ADRB assay independent of whether merozoites were used from the parasite strain 3D7 expressing MSP1-D or from FCB1 expressing MSP1-F (*p* < 0.01 according to paired *t*-test). The induced ADRB activities were comparable to those observed using total IgG pools from semi-immune Kenyans, but were on average lower than those induced by total IgG from semi-immune adults from a malaria high transmission region in Burkina Faso (Fig. 8a). Note that malaria semi-immune subjects have antibodies not only to MSP1 but also to other merozoite proteins, which explains the higher ADRB activity observed in the malaria exposed individuals as compared with the malaria-naïve vaccinees of this study.^48^ The ADRB activity was independent of the MSP1 dose with which the volunteers were immunized (*p* > 0.05, according to Kruskal–Wallis one way ANOVA on ranks) (Fig. 8a) and correlated with the MSP1-specific IgG antibody titers in a monotonic relationship (correlation coefficient, 0.545; *p* = 0.006, according to Spearman rank order correlation) (Fig. 8c).

### MSP1 can recall a memory T-cell response

**Fig. 9:**
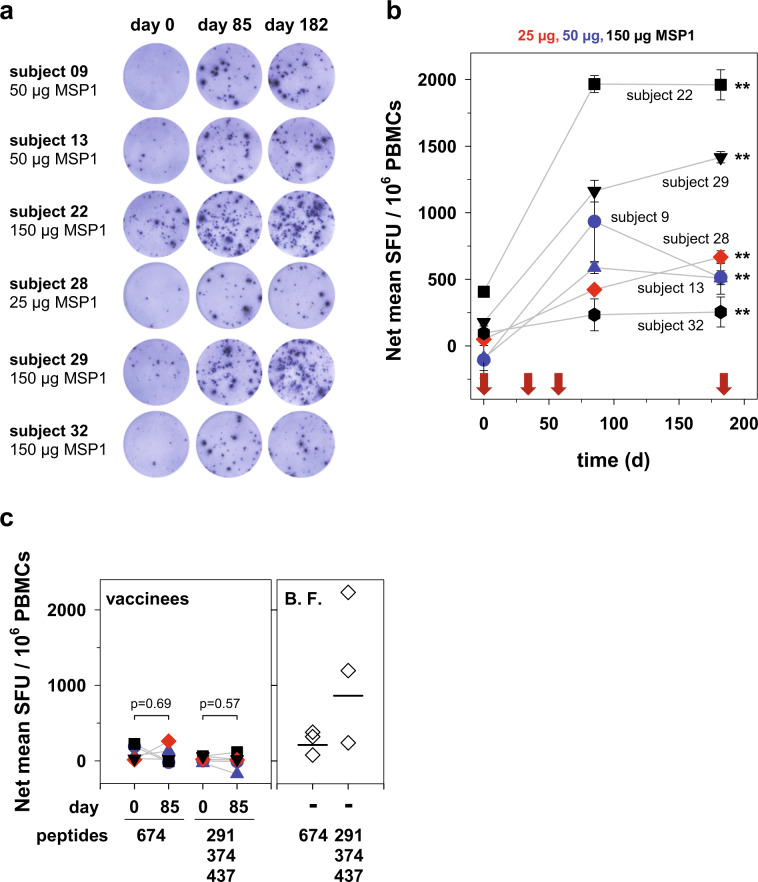
MSP1-specific recall T-cell response. PBMCs from six volunteers immunized three times with SumayaVac-1 were exposed to purified MSP1-D (5 µg ml^−1^ for 24 h) and the recall T-cell response as defined by IFN-γ production was measured in the cultured ELISpot assay. **a** Representative images of ELISpot wells are shown for different vaccinees. **b** The net mean spot-forming units (SFU) per 1 million PBMCs were calculated and analyzed as a function of time post the first immunization. Each data point represents the mean from three determinations ± SD. The red arrows indicate the days of immunization with SumayaVac-1. Significance was assessed using Kruskal–Wallis one way ANOVA on ranks. **c** Stimulation of PBMCs by peptides corresponding to HLA-A0201-restricted MSP1 CD8^+^ T-cell epitopes. PBMCs from the same vaccinees as above were analyzed (same color code as above), in addition to PBMCs from three semi-immune adults from Burkina Faso (B.F.). The following peptides were used: 291, GLHHLITEL; 374, SLLTELQQV; 437, VIYLKPLAGV; 674, KLKEFIPKV.^40,68,69^ Statistical significance was assessed using the two tailed *t*-test.

To investigate whether vaccination with SumayaVAC-1 induced the generation of memory T-cells, we isolated peripheral blood mononuclear cells (PBMCs) from all HLA-A0201-positive vaccinees at days 0, 85, and 182 post priming. HLA-A0201-positive vaccinees were chosen because they allowed us to investigate MSP1-specific CD4^+^ and CD8^+^ T-cell responses in paired assays (Note that MSP1-specific CD8^+^ T-cell epitopes have thus far been mapped in the HLA-A0201 background) (see below). PBMCs were exposed to full-length MSP1 (5 µg ml^−1^) as a recall antigen in the cultured IFN‐γ ELISpot assay (enzyme‐linked immunosorbent spot‐forming cell assay).^58,65,66^ We found that the cultured ELISpot responses significantly increased from day 0 to day 85 (4 weeks after the third immunization) (*p* < 0.001 according to paired *t*-test) and then remained stable in 5 out of 6 selected vaccinees for more than 4 months (Fig. 9a, b). Only PBMCs from volunteer 9 had a decrease in recall stimulation at day 182. As a positive control, we used a monoclonal antibody against CD3, a receptor that is associated with the T-cell receptor complex and which plays an important role in T-cell activation and signal transduction^67^ (Supplementary Fig. 1).

Previous studies have described four HLA-A0201-restricted CD8^+^ T-cell epitopes within MSP1.^40,68,69^ A peptide mix (10 mg ml^−1^) corresponding to three of the four MSP1 CD8^+^ T-cell epitopes (291, GLHHLITEL; 374, SLLTELQQV; 437, VIYLKPLAGV)^40,68,69^ stimulated PBMCs from malaria semi-immune, HLA-A0201-positive individuals from Burkina Faso in the IFN‐γ ELISpot assay but not from the HLA-matched vaccinees (Fig. 9c). A peptide corresponding to the fourth epitope (674, KLKEFIPKV) had no effect (Fig. 9c).

## Discussion

Our study differs from previous clinical trials in that we vaccinated volunteers with full-length MSP1 instead of rather small MSP1 fragments. The rationale behind pursuing the development of MSP1 as a blood stage and as a pre-erythrocytic vaccine candidate in spite of discouraging results from two phase 2 challenge trials in humans,^46,47^ is that the full-length MSP1 contains B-cell and T-cell epitopes not present in previous MSP1-based vaccine candidates (see Fig. 6),^32,40,48–52^ which comprised merely the p42 fragment or composites made of conserved MSP1 domains.^46,47^ Further supporting the vaccine potential of full-length MSP1, monkeys immunized with the native MSP1 purified from cultured parasites were protected from experimental infection with *P. falciparum* or could spontaneously clear a blood stage parasitemia.^43–45^ In contrast, monkeys immunized with p42, p19, or a MSP1 domain composite were only partly protected, i.e., some animals developed sterile immunity, whereas other animals were not protected at all.^70–72^

Full-length MSP1 formulated with GLA-SE was safe and well tolerated at primary and booster vaccinations at all dose levels tested up to 150 µg. Vaccinees experienced local and systemic events, typical of vaccine-induced physiological reactions. These events were mostly mild and always of transient nature (Table 1). The trial tested the highest target dose for vaccination without detecting any patterns suggestive of adverse off-target effects. However, our clinical trial did not include patients with malnutrition, genetic pre-dispositions, such as haemoglobinopathies and glucose-6-phosphate dehydrogenase deficiency, or any other condition that is frequent in malaria endemic countries and which might affect the safety and tolerability of SumayaVAC-1. We further acknowledge that minor trends might have escaped our attention due to the small sample size of six volunteers in some cohort groups. However, these limitations are not seen as major obstacles to the interpretation of our results.

All vaccinees who received SumayaVAC-1 sero-converted (Fig. 2a). The α-MSP1-specific IgG and IgM titers peaked after the third immunization and then persisted for ~6 months above or at levels found in semi-immune adults from malaria endemic areas. A fourth immunization administered at day 182 re-boosted the antibody titers, but did not increase them above the initial peak level at day 85. The half-life of ~70 days of the α-MSP1-specific IgG titers appears short in comparison to the long-lived antibody responses elicited by other vaccines, such as tetanus vaccine (half-life of 14 years) or diphtheria vaccine (half-life of 27 years).^73^ However, it is substantially longer than the half-life of circulating *P. falciparum*-specific IgG antibodies in natural infections that have a longevity of ~10 days in young children and quickly wane in malaria naïve individuals following an acute infection.^74,75^ However, it remains to be seen whether the α-MSP1-specific IgG titers induced by vaccination persist under a blood-stage challenge or whether they decrease in a manner similar to what is found in infected individuals in the field.

Vaccinees also mounted high, long-lasting α-MSP1-specific IgM titers (Fig. 2b). IgM are generally regarded as short-lived and of low affinity. However, recent studies have revised this notion by showing that *P. falciparum*-specific IgM have high affinities and contribute to protection from malaria, e.g., by mediating complement fixation and subsequent parasite lysis and/or phagocytosis.^76,77^ Moreover, a *Plasmodium* infection can elicit hypermutated specific IgM memory B cells that are long-lived and are at the front line of defense against a rechallenge.^76^ We consider the long-lasting IgG and IgM antibody titers a quality feature of the full-length MSP1/GLA-SE immunization concept and hypothesize that they arise from longer-lived plasma cells and possibly memory B-cells, although further studies are needed to validate this hypothesis.

The finding that the antibody titers were independent of the immunization dose suggests that the lowest dose of 25 µg of full-length MSP1 used in our study fully sufficed to elicit a saturating humoral immune response, at least in malaria-naïve individuals. However, the fine-scale epitope mapping revealed a more nuanced picture by showing that vaccinees who received 50 µg of MSP1 seemed to mount a more diverse and stronger immune response against this protein than individuals in the other two dosing groups (Fig. 6). In general, the epitope landscapes appeared more similar in the 50 µg MSP1 group to those of malaria semi-immune adults and MSP1-immunized rabbits than to those of the 25 and 150 µg MSP1 groups. However, further studies are needed to support this observation given the limited sample sizes in each study group. While a 50 µg dose seems optimal for malaria naïve individuals, it is unclear whether this dose is suitable in other clinical settings. Firstly, individuals living in malaria endemic areas (adults and infants) might require a higher dose given their lower responsiveness to vaccination compared with healthy and well nurtured malaria naïve adults.^73^ Secondly, MSP1 is highly immunogenic and antibodies acquired in natural exposure might interfere, in a positive or negative manner, with MSP1 vaccine immunogenicity and efficacy. Regardless, vaccination with the MSP1 subunit p42 yielded high antigen-specific antibody titers, albeit no protective efficacy, in a phase 2b trial with 400 Kenyan children.^46^ Thus, naturally acquired MSP1 antibodies do not necessarily dampen the immunogenicity of an MSP1 based vaccine.

Antibodies can limit blood stage development of *P. falciparum* in multiple ways, i.e., by directly inhibiting merozoite invasion and/or intraerythrocytic development and by recruiting immune effector cells. Several in vitro assays have been described to assess the functionality of malaria-protective antibodies. However, a full understanding of the underlying protective processes is lacking and a correlate of protection from falciparum malaria has yet to be defined.^7^

The MSP1-specific antibodies mounted by vaccinees were capable of recognizing native MSP1 presented on merozoites, as shown by immunofluorescence and merozoite-specific ELISA (Fig. 4). Intuitively, one would assume that these α-MSP1 antibodies should directly inhibit parasite growth given the pivotal role of MSP1 in host cell recognition and invasion. Although some studies have reported growth inhibition by human α-MSP1 antibodies under in vitro conditions,^23,31–34^ other studies failed to detect such an effect or reported a lack of association between GIA activity and protection from clinical malaria.^6,46,47,78–80^ In a recent development, it has been shown that antibody-mediated complement fixation on merozoites via the classical pathway and the subsequent formation of the membrane attack complex is more strongly associated with immunity against malaria than is GIA activity.^81^ The absence of an appreciable GIA or IIA activity as observed in our study is, therefore, not unexpected and does not afford a qualified assessment of the potential clinical efficacy of full-length MSP1 against malaria.

Irrespectively, the question remains why some sera and IgG preparations inhibit in vitro parasite growth whereas others do not. This discrepancy has been explained by differences in antibody titers and the need for high titers to interfere with parasite growth.^82^ Alternatively it has been suggested that complement is necessary for anti-merozoite antibodies to deploy their full parasite killing potential.^35^ However, these two explanations cannot easily account for the lack of GIA and IIA activity in our study. α-MSP1 IgG antibody titers elicited by immunization with full-length MSP1 were high, even exceeding those found in GIA and IIA-active IgG preparations from malaria semi-immune adults and MSP1-immunized rabbits. Furthermore, active complement did not enable GIA activity, although α-MSP1 IgG antibodies from vaccinees were able to activate complement via the classical pathway in the presence of merozoites, as shown using a semi-quantitative C1q ELISA (Fig. 5c). Since antibody titers and lack of complement activation could not explain our GIA and IIA results, we explored a third option, namely differences in epitope profiles. Indeed fine-scale epitope mapping revealed different epitope landscapes between GIA active and inactive IgG preparations (Fig. 7), which might even be more extensive when including conformational epitopes in addition to the linear epitopes mapped herein.

Epitopes associated with GIA activity mapped to the MSP1 processing sites between p30 and p38 and between p38 and p33^16^ (Fig. 7). This finding agrees with previous studies showing that antibodies blocking maturation of MSP1 inhibit invasion.^83^ Another epitope included a lysine that is posttranslationally modified by acetylation (Fig. 7). How antibody binding to this and the additional epitopes identified in our screen affects MSP1 function awaits further studies. A possible explanation might be that these GIA-associated epitopes are involved in MSP1 complex formation and might directly or indirectly facilitate assembly of MSP1 subunits and/or the recruitment of MSP6, MSP7, or other peripheral factors.^17,18,84^ Thus, the lack of GIA activity seen in sera from MSP1 vaccinees is most likely owed to the inability of SumayaVac-1 to induce antibodies against certain MSP1 epitopes involved in processing and/or protein/protein interactions.

The high-resolution epitope mapping further supports previous studies in naturally exposed adults suggesting that the antigenic repertoire of MSP1 extends beyond the p42 domain and involves the entire protein (Fig. 6).^32^ Of particular interest are epitopes KYLIDGYEEIN (position 180–190), (NINEL) (position 266–270), and (FTDPLELE) (position 384–391), which map to conserved domains of MSP1 and which might explain the broad cross-reactivity of the vaccine-induced MSP1-specific antibodies against both MSP1 prototypes.

In addition to triggering complement activation, α-MSP1 IgG antibodies from vaccinees were able to opsonize merozoites and stimulate immune effector cells, as exemplified by ROS-producing neutrophils (Fig. 8). ROS can kill both free and phagocytosed parasites in vitro,^36,62,85^ and immune-epidemiological studies have associated ROS production with parasite clearance and protection from clinical malaria.^62,86^ Consistent with the observed ADRB activity, the vast majority of the MSP1-specific antibodies produced by the vaccinees belonged to the cytophilic, opsonizing subclasses IgG1 and IgG3 (92.1% and 6.5%) (Fig. 2a), which can recruit Fc-receptor presenting immune cells via their Fc domain. Opsonizing antibodies, including opsonizing antibodies against MSP1, are critically involved in controlling a blood stage parasitemia.^87–91^ In addition to stimulating neutrophils, they can activate human natural killer cells^92^ and monocytes, the latter eliminating parasites through phagocytosis or the release of TNF-α and other soluble factors.^87–91,93,94^

CD4^+^ T-cells have a critical function in the immune response to malaria.^95^ They help B-cells produce the specific antibodies that control blood stage parasitemia. They further secrete IFN-γ and other cytokines that themselves are parasitocidal or modulate the innate immune response.^95^ Given that the GLA-SE adjuvant is known to stimulate Th1 CD4^+^ T-cell responses to co-administered antigens,^57^ it is reasonable to assume that SumayaVAC-1 acted accordingly and primed CD4^+^ T-cells to produce IFN-γ, as determined in the ELISpot assay (Fig. 9a, b). Although natural killer cells and CD8^+^ T-cells are also able to secrete IFN-γ,^67,96^ we do not think that they were the major source of IFN-γ production in the ELISpot assays. This conclusion is partly supported by the finding that peptides corresponding to established MSP1 CD8^+^ T-cell epitopes failed to stimulate the PBMCs from vaccinees (Fig. 9c). Thus, while we cannot exclude the possibility of SumayaVac-1 inducing an NK cell and/or a CD8^+^ T-cell response, we find it unlikely on the basis of the currently available information.

Re-stimulation of PBMCs with purified MSP1 demonstrated a T-cell response more than 4 months after the last immunization, which was possibly mediated by CD4^+^ T-cells. These findings are consistent with the presence of circulating memory CD4^+^ T-cells in vaccinees. Thus, there is evidence of SumayaVAC-1 eliciting a memory immune response, which would be a prerequisite for providing lasting protection against a *P. falciparum* infection.

In summary, immunization with SumayaVAC-1 was well tolerated and elicited a humoral immune response that resulted in the generation of functional antibodies that were able to trigger downstream effector mechanisms associated with immunity against malaria. However, to realize the full potential of full-length MSP1 as both a blood stage and a pre-erythrocytic malaria vaccine candidate, it might be necessary to combine the protein/GLA-SE preparation with a viral delivery system in a prime boost regimen in an effort to induce a CD8^+^ T-cell response, in addition, to a humoral immune response. CD8^+^ T-cell responses against MSP1 would then be able to target pre-erythrocytic liver stages, thereby neutralizing merozoites before they are released into the blood stream to establish the intraerythrocytic life cycle that produced the pathological disease manifestation. This study sets the stage for a subsequent clinical challenge trial in humans.

## Methods

### SumayaVAC-1

MSP1 was produced in *E. coli* as a heterodimer consisting of the p83/p30 and p38/p42 subunits.^53^ The heterodimer was purified to >99.9% under GMP conditions by Biomeva GmbH, Heidelberg. The material was lyophilized during “fill & finish” by Praxis Pharmaceutics SA, Minano, Spain, following a procedure developed by Project Pharmaceutics GmbH, München. MSP1 is stored in 2 ml vials as a white cake of 0.150 mg per vial at −65 °C under GMP conditions by Glycotope Biotechnology GmbH, Heidelberg. GLA-SE was obtained from IDRI (Seattle). 25, 50, or 150 µg of full-length MSP1 were applied with 5 µg GLA-SE in a volume of 500 µl.

### Patients and clinical study design

The trial was performed following the principles of good clinical practice (GCP) and in accordance with the ethical principles described in the then applicable version of the Declaration of Helsinki (6th revision, 2008). The trial was registered with EudraCT (No. 2016-002463-33; date of first approval 31 January 2018) and the protocol and subsequent amendments were approved by the responsible Ethics Committee of the Medical Faculty of Heidelberg (Ethical vote AFmo-538/2016) and the relevant regulatory authority (Paul Ehrlich Institute, Langen, Germany). All volunteers were fully informed about the trial and gave their written consent prior to any study procedures. The study started in April 2017 (first enrolment of a participant) and ended after full recruitment and last participants visit in December 2018.

We conducted a single-center, randomized, double-blind, placebo and adjuvant-controlled phase 1a first-in-human dose escalation trial to evaluate the local and systemic tolerability, as well as the immunogenicity of the vaccine SumayaVAC-1 in 32 healthy volunteers after intramuscular administration. The trial was conducted at the ISO-certified clinical trial unit of the Clinical Pharmacology and Pharmacoepidemiology Department of the Heidelberg University Hospital. Eligibility criteria were chosen to enroll healthy volunteers. Sixteen participants were included in two consecutive cohorts, each randomizing 12 volunteers into a vaccine + adjuvant group, and two participants each receiving placebo or adjuvant only. The safety of the study and any dose increase was supervised by an independent Data and Safety Monitoring Board (DSMB).

Cohort 1 randomized 12 volunteers to 50 µg of MSP1. After completion and unblinding of cohort 1, a positive DSMB vote opened randomization into cohort 2. Originally, 12 volunteers should have been randomized to 150 µg MSP1 in cohort 2, but based on the antibody results from cohort 1, randomization of six volunteers each for 150 and 25 µg MSP1 was carried out after a protocol amendment. All volunteers received at least three vaccinations at intervals of 29 ± 3 days, 18 volunteers on active treatment (5, 9, and 4 volunteers in the 25, 50, and 150 µg MSP1 vaccination cohorts) opted for a fourth vaccination about 4 months after the third vaccination, which was offered to volunteers on active treatment only after unblinding of the cohort. A post-trial safety follow-up was carried out 6 months after the last vaccination (Fig. 1a, b). The co-primary objective of the trial was to evaluate the safety and the immunogenicity of the vaccine. Safety endpoints assessed the immediate and delayed systemic reactions to the vaccine, immediate and delayed local reactions to the vaccine, adverse events leading to permanent sequelae, serious adverse events, and changes in laboratory parameters. For the safety outcome solicited and unsolicited adverse events were recorded after each vaccination for 29 d and during the safety follow-up and assessed for seriousness, severity (Common Terminology Criteria for Adverse Events 4.0 (CTC-AE)), and the casual relationship to the study drug of the event. Immediate reactions were defined as adverse events with a positive relationship appearing within 30 min of application. Delayed reactions were defined as adverse events with a positive relationship occurring > 30 min after application of the study drug until the last assessment of the cycle after 29 ± 3 d. Solicited systemic reactions to the injection included fatigue, chills, sweating, myalgia, arthralgia, gastrointestinal symptoms (e.g. nausea, vomiting), headache, fever >38 °C, rash, or symptoms specifically reported by the volunteers. Solicited local reactions to the injection included warmth, erythema, itching, edema, pain, infection, lipodystrophy, ulceration, induration, necrosis, and other tissue damage. Safety evaluation was based on the respective guidance from the authorities.^97,98^ As per definition in a first-in-human trial all events were unexpected.

The pre-specified co-primary objective immunogenicity was assessed by evaluating antibody responses to MSP1 (IgG and IgM) via ELISA, the cross-reactivity of antibodies to the MSP1-D and MSP1-F prototypes, the parasite growth inhibitory properties of antibodies (GIA), the ability of IgG antibodies to opsonize merozoites and stimulate a respiratory burst by granulocytes in the ADRB assay, and cellular immune responses in the ELISpot assay. In addition, exploratory immunogenicity endpoints were defined to further characterize the antibody responses. This included studies to assess the ability of induced antibodies to inhibit parasite invasion (IAA), react with native MSP1 on the surface of merozoites, recognize different MSP1 subunits, and activate complement. Additional exploratory immunogenicity endpoints were comparative B-cell epitope maps. Blood samples for immunogenicity measures were taken as indicated in Fig. 1a. There were no clinical efficacy endpoints assessed in this trial. Pre-specified endpoints and measures to allow evaluation of the objectives were not changed after trial commencement. Further details on the responsibility of the DSMB are available in the Supplementary material on study conduct. The full study protocol is available as Supplementary material.

### Sera from malaria semi-immune adults

A WHO reference reagent containing pooled sera derived from individuals based in Kimusu, Kenya, with a history of malaria was obtained from the National Institute for Biological Standards and Control (NIBSC code 10/198). Sera from adults based in Nouna, Burkina Faso, with history of malaria have recently been described.^48^ Where indicated 11 of these sera were pooled and are referred to herein as the Burkina Faso (B.F.) pool.

### Purification of human sera and PBMCs

Blood samples were collected in S-Monovette serum-gel vacutainers treated with clotting activator (Sarstedt) for serum preparation and in vacutainer cell preparation tubes (CPT) with sodium-citrate (BD Biosciences) for isolation of PBMCs. Serum was prepared from clotted blood by centrifugation for 10 min at 2500 × *g* and stored at −80 °C. For PBMC isolation, whole blood was centrifuged for 30 min at 1700 × *g*, and the PBMC layer was then transferred and washed twice with PBS. The pellet was re-suspended in 0.5 ml freezing medium (either 50% FBS and 50% RPMI 1640 or 12.5% HSA and 87.5% RPMI 1640) and the cell number was determined by trypan blue staining. Following addition of 0.5 ml DMSO medium (either 50% FBS, 30% RPMI 1640, and 20% DMSO or 12.5% HSA, 67.5% RPMI 1640, and 20% DMSO) dropwise, the cells were frozen immediately in a freezing container at −80 °C. After 24 h, PBMCs were transferred into liquid nitrogen.

### Purification of IgG from human sera

Total IgG was purified from heat inactivated sera (30 min at 56 °C) by protein-G affinity chromatography (Pierce, Thermo Fisher Scientific), according to the manufacturer’s instructions.^48^ The IgG eluate was sterile filtered (0.22 µm), dialyzed into RPMI 1640 and concentrated with Amicon ultra centrifugal filters (Millipore). The IgG concentration was adjusted to 30 mg ml^−1^ (for GIA and IIA) and 5 mg ml^−1^ (for ADRB) in RPMI 1640, and aliquots were stored at −20 °C.

### Enzyme-linked immunosorbent assay (ELISA)

Total IgG antibody levels were determined by ELISA.^48^ To this end, MaxiSorp plates (Thermo Fisher Scientific) were coated with 100 nM recombinant MSP1 (Glycotope Biotechnology GmbH, Heidelberg) or MSP1 subunits in 0.1 ml-coating buffer (34 mM Na_2_CO_3_, 16 mM NaHCO_3_, pH 10.6). The production and purification of MSP1 subunits has been described.^99,100^ For the merozoite ELISA, plates were coated with 1 µg *P. falciparum* merozoites in 0.1 ml PBS. Sera were titrated in two-fold dilutions and incubated for 2 h. To detect binding antibodies, the secondary antibody goat anti-human IgG-alkaline phosphatase conjugate (Sigma-Aldrich) was used at a dilution of 1:20,000 for 1 h. The substrate *p*-nitrophenyl-phosphate (1 mg ml^−1^ in 0.96% diethanolamine and 1 mM MgCl_2_, pH 9.5) was added and the reaction was then incubated for 1 h in the dark, and then stopped by addition of 0.1 ml of 1 M NaOH. The absorbance at 405 nm was determined using the plate reader Cytation 3 (BioTek). For the IgG subtype ELISA, the same protocol was applied, with the exception that subclass-specific secondary antibodies were used. IgG subtype antibodies were detected by peroxidase-conjugated anti-human IgG1, anti-human IgG2, anti-human IgG3, and anti-human IgG4 antibodies diluted according to the manufacturer’s instructions (The Binding Site GmbH). The substrate 1-step turbo TMB (Thermo Fisher) was added for 20 min in the dark, then stopped by adding 1 M HCl. Optical density was recorded at 450 nm. As a positive control, purified human myeloma protein was used according to the manufacturer’s instructions (The Binding Site GmbH). IgM was analyzed in the same way, but as secondary antibody a goat anti-human IgM HRP conjugate (Sigma-Aldrich) was used (dilution: 1:20,000). The substrate SigmaFAST OPD (Sigma-Aldrich) was incubated for 20 min in the dark before the reaction was stopped by the addition of 1 M HCl and measured at 492 nm.

### C1q ELISA

The MSP1-specific C1q ELISA was performed using the following protocol.^101^ Plates were coated with recombinant MSP1 (100 nM in PBS) and incubated with serum samples in duplicates for 2 h (diluted 1:25 in 0.1% casein/PBS). Anti-C1q antibodies conjugated with HRP (1:100; Abcam) were incubated for 1 h at 37 °C and detected using the substrate SigmaFAST OPD (Sigma-Aldrich) (incubating for 30 min to 1 h in the dark). Reactions were stopped with 1 M HCl and analyzed at 492 nm.

### Immunofluorescence assay (IFA)

Parasite cultures were synchronized using 5% sorbitol.^61,102^ Trophozoites (24–36 h post invasion) and schizonts (40–48 h post invasion) were fixed in 4% paraformaldehyde and 0.0075% glutaraldehyde in PBS for 30 min at 37 °C while shaking. Merozoites were not fixed and analyzed immediately. Cells were washed with PBS and blocked using PBS containing 3% BSA for 1 h, followed by an overnight incubation with α-MSP1 sera from vaccinees (1:25 dilution in blocking buffer) at 4 °C, while gently agitating the tube the entire time. The next day, the parasites were washed thoroughly with PBS, incubated with Alexa Fluor 647 goat anti-human (1:1000, BioLegend) for 1 h followed by three washing steps, the last one with the addition of Hoechst 33342 (1 µg ml^−1^, Thermo Fisher Scientific). Samples were transferred onto concanavalin A (500 µg ml^−1^, Sigma-Aldrich)-coated slides and observed using a Nikon Eclipse Ti microscope (Nikon Instruments Europe B.V.) equipped with an Orca Flash 4.0 camera (Hamamatsu Photonics K.K.).

### *P. falciparum* culture and isolation of merozoites

The *P. falciparum* strains 3D7 and FCB1 were cultured in RPMI 1640 medium supplemented with 0.1 mM hypoxanthine, 20 µg ml^−1^ gentamycin and 0.25% Albumax I. Cultures were synchronized by sorbitol treatment.^60,102^ The merozoites for ADRB were isolated from a synchronous late stage culture. Infected erythrocytes were purified by magnet separation (CS columns, MACS vario, Miltenyi Biotec), then cultured for at least 10 h under standard conditions in the absence of erythrocytes. Merozoites were recovered from the supernatant by filtering through a 1.2 µm Acrodisc 32 mm syringe filter (Pall), followed by a centrifugation step at 3000×*g* for 10 min. The merozoite pellet was re-suspended in RPMI 1640 and aliquots were stored at −20 °C. Purity of merozoites was confirmed by Giemsa-stained smears.

### Growth (GIA) and invasion (IIA) inhibition assays

For GIA, purified total IgG at the concentrations indicated were added to highly synchronized *P. falciparum* cultures (strain 3D7) at the late schizont stage (0.6% parasitemia, 4% hematocrit). The cells were returned to culture for 40 h before the cells were harvested and the lactate dehydrogenase (LDH) activity was determined as an indicator of parasite development.^48^ The activity of the *Plasmodium* LDH, was detected at a wavelength of 650 nm, using the Cytation 3 microplate reader (Biotek). All experiments were performed in triplicates. For the IIA,^61^ purified total IgG at a concentration of 3 mg ml^−1^ was added to merozoites (4% hematocrit). The cells were subsequently returned to culture for 30 min, while gently shaking. After two washing steps with culture media, parasites were cultured under standard conditions for 30–40 h. Parasitemia was determined by flow cytometry using Sybr Green (Thermo Fisher Scientific) staining. The rabbit α-apical membrane protein 1 serum pool was purchased from BioGenes, Berlin (reference number: BG98).

### Purification of neutrophil granulocytes

PMNs were isolated from whole blood of healthy malaria-naïve individuals.^48^ To this end, whole-blood from three malaria-naive healthy donors (18 ml each) was pooled, mixed 1:1 with 3% dextran (Carl Roth) in 0.9% NaCl, and incubated for 18 min at room temperature to pellet red blood cell. After centrifugation (500 × *g* for 10 min at 4 °C), the thin white layer was collected and resuspended in 0.9% NaCl. This suspension was layered on top of Ficoll-Histopaque (Sigma-Aldrich) and centrifuged at 400×*g* for 35 min at room temperature. The thin PMN layer was resuspended in ice-cold distilled water and kept on ice for 30 s to lyse remaining erythrocytes before an equal volume of 1.8% NaCl was added. After centrifugation (500 × *g* for 5 min at 4 °C), the pellet was washed with Hanks’ balanced salt solution (Thermo Fisher Scientific) and resuspended in cold PBS. The quality of the preparation and the number of PMN were determined in a hemacytometer after trypan blue staining. The PMN concentration was adjusted to 2.5 × 10^7^ PMNs ml^−1^ in sterile PBS.

### ADRB assay

Opsonizing antibodies that activate neutrophil granulocytes to produce ROS were measured via the ADRB assay.^48,62^ Purified human IgG (5 mg ml^−1^, 10 µl per well) and 50 µl sterile PBS were added to white 96-well Lumitrac microplates (Greiner Bio-One) and pre-incubated for 1 h at 37 °C. Following addition of thawed *P. falciparum* merozoites in RPMI 1640 (~0.3 mg ml^−1^, 40 µl per well) the plate was incubated for 1.5 h at 37 °C. Freshly purified human neutrophil granulocytes (PMNs, 2.5 × 10^7^ ml^−1^ in PBS, 50 µl per well) and isoluminol (100 µl per well, 0.04 mg ml^−1^ in PBS, Santa Cruz Biotechnology) were pipetted rapidly with a multichannel pipette. Chemiluminescence activity was immediately detected by the Cytation 3 microplate reader (Biotek) measuring every minute over 1 h. The ADRB index was calculated by normalizing the detected chemiluminescence activity (light units, LU) of each sample to an IgG pool from malaria-exposed individuals from Kenya (NIBSC code 10/198) using the equation: ADRB Index = (LUsample max./LUKenya pool max.) × 1000. Controls on each plate were (i) only isoluminol, (ii) individuals before immunization (day 0) as IgG pool, (iii) IgG pool from semi-immune individuals from Burkina Faso, (iv) IgG pool from malaria-exposed individuals from Kenya. Different time points of one individual were measured on the same plate.

### Enzyme-linked immunospot assay (ELISpot)

T-cell responses against MSP1-D protein were examined for HLA-A0201-positive vaccinees via an IFN-γ ELISpot assay (Mabtech, Sweden). Frozen PBMCs were thawed using warm ELISpot medium (RPMI 1640, 10% fetal calf serum, 200 mM l-glutamine, 1% penicillin/streptomycin) supplemented with 50 U ml^−1^ benzonase (Novagen Millipore GmbH). After centrifugation for 5 min at 550×*g* the cells were allowed to rest for 1.5 h at 37 °C in ELISpot medium, centrifuged again, counted and adjusted to 5 × 10^6^ cells ml^−1^ with ELISpot medium. For pre-stimulation, 300 µl PBMCs (1.5 × 10^6^ cells) and 300 µl ELISpot medium (unstimulated control) or 300 µl complete GMP-grade MSP1-D protein (final concentration of 5 µg ml^−1^; BIOMEVA GmbH, Heidelberg) or MSP1 peptides (GLHHLITEL, SLLTELQQV, VIYLKPLAGV, KLKEFIPKV purchased from JPT Peptide Technologies GmbH and Peptide Speciality Laboratories GmbH) in RPMI 1640 at a final concentration of 10 µg ml^−1^ were incubated in polypropylene tubes (Greiner) for 22–24 h at 37 °C. 96-well multiscreen-IP filter plates (Merck Millipore GmbH, MAIPS4510) coated overnight with IFN-γ antibody (1-D1K, 1:100 in PBS) were washed 3× with sterile PBS, blocked with ELISpot medium for 1 h and the stimuli described above were added in triplicates (50 µl per well) with a fourth well containing a human α-CD3 mAb control (0.1 µg ml^−1^, Mabtech). The pre-stimulated PBMCs were centrifuged for 6 min at 550×*g*, resuspended in 250 µl ELISpot medium, and 50 µl per well (~200,000 cells) were added to the ELISpot plate. The plate was incubated for 24 h at 37 °C and the exact number of transferred living PBMCs from each tube was determined by cell counting after staining with trypan blue. The IFN-γ ELISpot assay was performed according to the manufacturer’s instructions (Mabtech) using biotinylated detection antibodies, streptavidin-alkaline phosphatase and BCIP/NBT-plus substrate. The plate was scanned and counted on the ImmunoSpot Reader S6 Ultra-00-6052 (C.T.L.) using the software ImmunoSpot 6.0.0 (C.T.L.). The number of activated cells secreting IFN-γ (spot forming units, SFU) in 10^6^ PBMCs was calculated in Excel using the equation: Net SFU/10^6^ cells = (meanspots in stimulated wells–meanspots in unstimulated wells)/living cells per well × 10^6^.

### MSP1 epitope mapping

High-resolution epitope mapping was performed by PEPperPRINT GmbH Heidelberg. MSP1 sequence (3D7) was elongated with neutral GSGSGSG linkers at the C-terminus and the N-terminus to avoid truncated peptides. The elongated antigen sequence was translated into linear peptides consisting of 15 amino acids with a peptide–peptide overlap of 14 amino acids. The resulting peptide microarrays contained 1706 different peptides printed in duplicate (3440 peptide spots) and were framed by additional HA (YPYDVPDYAG, 62 spots) and polio (KEVPALTAVETGAT, 60 spots) control peptides. After blocking a peptide microarray copy for 30 min with blocking buffer (Rockland MB-070), pre-staining was done with the respective secondary antibodies goat anti-human IgG (Fc) DyLight680 (0.1 µg ml^−1^), goat anti-human IgM (µ chain) DyLight800 (0.2 µg/ml), anti-rabbit IgG (Fc) DyLight680 (0.2 µg ml^−1^), and the monoclonal mouse anti-HA control antibody (12CA5) DyLight800 (0.5 µg ml^−1^) in incubation buffer for 45 min to investigate background interactions with the printed peptides that could interfere with the main assays. Subsequent incubation of other peptide microarray copies with the human serum samples and controls (1:1000) and the rabbit controls (1:10,000) for 16 h at 4 °C, shaking at 140 rpm, was followed by staining with labeled secondary and control antibodies. Read-out was done at scanning intensities of 7/7 (red/green) with a LI-COR Odyssey Imaging System. Additional HA control peptides framing the peptide microarrays were simultaneously stained as internal quality control to confirm the assay quality and the peptide microarray integrity. Quantification of spot intensities were based on the 16-bit gray scale tiff files at scanning intensities of 7/7 that exhibit a higher dynamic range than the 24-bit colorized tiff files. Microarray image analysis was done with PepSlide Analyzer. A software algorithm breaks down fluorescence intensities of each spot into raw, foreground, and background signal for each channel (700 nm — red, IgG and 800 nm — green, IgM). As each peptide on the peptide array is printed as duplicate, the raw peptide spot fluorescence intensity is the average of the corresponding two spot duplicates. A maximum spot-to-spot deviation of 40% was tolerated, otherwise the corresponding intensity value was zeroed. Averaged spot intensities were plotted against the microarray content (overlapping MSP1 peptides from the N- to the C-terminus).

### Data management

All clinical data were monitored and entered into a clinical database. Database closure occurred after resolution of any open queries.

### Statistics and reproducibility

All participants were included in the safety and immunogenicity analyses. Safety data were analyzed for vaccination 1–3, and analyzed separately for the open label fourth vaccination. Denominators are indicated in Fig. 1 and figure legends. Safety data were analyzed by descriptive methods, location/spread measures like mean ± standard deviation, median/range, and relative frequencies in per cent. Subgroup analyses were carried out for safety analyses on participants who received boost vaccination (methods did not vary from presentation of full collective). Immunogenicity data were analyzed by assessing statistical significances using paired *t*-test, *t*-test, or Kruskal–Wallis one-way ANOVA on ranks, where indicated. *P*-values are indicated on the graphs. *P* ≤ 0.01 was considered statistically significant. LOD scores were calculated as described.^103^ Immunogenicity data are given as geometric mean ± 95% confidence interval throughout this study, if not indicated otherwise. Data were analyzed using Sigma Plot version 13.0 (Systat Software).

### Ethics approval

The trial was registered with EudraCT (No. 2016-002463-33) and approved by the responsible Ethics Committee of the Medical Faculty of Heidelberg (ethical vote AFmo- 538/2016) and the relevant regulatory authority (Paul Ehrlich Institute, Langen, Germany). Written informed consent was given by all volunteers. Ethical approval for blood donations in Nouna, Burkina Faso, was granted by the Ethics Committee of the Medical Faculty of Heidelberg (Ethical vote 369/2003) and B. Kouyaté, director of the Centre de Recherche en Santé de Nouna (CRSN). Written informed consent was given by all blood donors.

### Reporting summary

Further information on research design is available in the Nature Research Reporting Summary linked to this article.

## Supplementary information

full supplementary data

reporting summary

## Data Availability

The authors declare that the data supporting the findings of this study are available within the article and its supplementary information files, or are available from the authors upon request. The original data underlying this article are available at DRYAD 10.5061/dryad.kwh70rz0f.
