# Supplementary material for: Immunization with full-length Plasmodium falciparum merozoite surface protein 1 is safe and elicits functional cytophilic antibodies in a randomized first-in-human trial
Source: NPJ Vaccines. 2020 Jan 31;5:10. doi: 10.1038/s41541-020-0160-2 (PMC6994672; doi:10.1038/s41541-020-0160-2)
Supplement: Supplementary file 1 — full supplementary data [file 41541_2020_160_MOESM1_ESM.pdf]

Supplementary Table 1: Distribution of solicited and unsolicited adverse events by MedDRA High Level Group Term by treatment group and cycle (regular: 1-3, boost: 4). The figures in cells represent number of events followed by the number of participants in brackets.

	**Vaccination 1 to 3 (blinded)**					**Vaccination 4 (optional - open)**		
	SumayaVac-1			GLA-SE	0.9 % NaCl	SumayaVac-1		
	25 µg	50 µg	150 µg	5 µg	1 ml	25 µg	50 µg	150 µg
	N = 6	*N = 12*	*N = 6*	*N = 4*	*N = 4*	*N =5*	*N = 9*	*N = 4*
Administration site reactions	28 (6)	101 (12)	29 (6)	16 (4)	14 (4)	8 (5)	17 (7)	8 (3)
Headaches	12 (5)	13 (7)	8 (4)	5 (3)	10 (2)	2 (1)	5 (3)	
General system disorders NEC	4 (2)	13 (8)	9 (4)	3 (1)	4 (3)		4 (2)	
Infections - pathogen unspecified	11 (6)	10 (7)	3 (3)	1 (1)	2 (2)	1 (1)	4 (3)	2 (2)
Epidermal and dermal conditions	1 (1)	14 (7)	2 (2)	3 (2)	2 (2)	1 (1)		1 (1)
Protein and chemistry analyses NEC	1 (1)	5 (3)	5 (3)	3 (2)		2 (2)	3 (2)	1 (1)
Gastrointestinal signs and symptoms	3 (3)	6 (4)	2 (1)	5 (1)			2 (2)	
Vascular haemorrhagic disorders	1 (1)	6 (4)	1 (1)	6 (3)				
Hepatobiliary investigations	2 (1)	5 (4)		2 (2)	1 (1)			1 (1)
Skin appendage conditions	3 (2)	3 (3)	2 (2)	1 (1)	1 (1)			
Haematology investigations (incl blood groups)	1 (1)	3 (3)	5 (2)					
Injuries NEC		3 (3)	3 (2)	1 (1)				2 (2)
Gastrointestinal motility and defaecation conditions		6 (4)	1 (1)		1 (1)			
Neurological disorders NEC	1 (1)	2 (1)	1 (1)	1 (1)	3 (1)			
Spleen, lymphatic and reticuloendothelial system disorders	1 (1)	5 (3)	1 (1)					1 (1)
Respiratory tract signs and symptoms	1 (1)	3 (2)		1 (1)		2 (1)		
Joint disorders	1 (1)				1 (1)	2 (1)	2 (2)	
Muscle disorders	2 (2)	1 (1)	2 (2)			1 (1)		
Musculoskeletal and connective tissue disorders NEC	2 (1)	2 (1)			1 (1)		1 (1)	
Body temperature conditions	2 (2)	2 (2)		1 (1)				

Supplementary Table 2: Original data underlying Fig 6. (MSP1 microarray) and Fig. 7 (LOD score analysis). The table shows the results of an MSP1 overlapping peptide array hybridized with sera from MSP1-immunized rabbits, donors from a malaria holoendemic area in Burkina Faso, and volunteers vaccinated with MSP1 at the dose indiated.
The hybridization intensities are depicted. The peptides cover the MSP1 sequence from N- to C-terminus. The organization of MSP1 in conserved, dimorphic and polymorphic blocks is indicated. The LOD score correlate the epitope signal strength with GIA activity.

	Peptides	Rabbit 1784	Rabbit 1787	Burkina Faso	Burkina Faso	Placebo #2	Placebo #8	Placebo #20	Placebo #23	25µg #21	25µg #24	25µg #25	25µg #28	25µg #30	25µg #31	50µg #3	50µg #4	50µg #6	50µg #7	50µg #9	50µg #10	50µg #11	50µg #12	50µg #13	50µg #14	50µg #15	50µg #16	150µg #19	150µg #22	150µg #26	150µg #27	150µg #29	150µg #32	Block No	LOD score
1	MKIIFFLCSFLFFII	0	0	0	0	0	0	0	0	0	0	0	0	0	0	0	0	0	0	0	0	0	0	0	0	0	0	0	0	0	0	0	0	1	
2	KIIFFLCSFLFFIIN	0	0	0	0	0	0	0	0	0	0	0	0	0	0	0	0	0	0	0	0	0	0	0	0	0	615.5	0	0	0	0	0	0		-0.41
3	IIFFLCSFLFFIINT	0	0	0	0	0	0	0	0	0	0	0	0	0	0	0	0	0	0	0	0	0	0	0	0	0	0	0	0	0	0	0	0		
4	IFFLCSFLFFIINTQ	0	0	0	0	0	0	0	0	0	0	0	0	0	0	0	0	0	0	0	0	0	0	0	0	0	0	0	0	0	0	0	0		
5	FFLCSFLFFIINTQC	0	0	0	241.5	0	0	0	0	0	0	0	0	0	0	0	0	0	0	22.5	0	0	0	0	0	0	0	0	0	0	0	0	0		1.32
6	FLCSFLFFIINTQCV	0	0	0	41.5	0	0	0	0	0	0	0	0	0	0	0	0	0	0	0	0	0	0	0	0	0	0	0	0	0	0	0	0		1.37
7	LCSFLFFIINTQCVT	0	0	0	0	0	0	0	0	0	0	0	0	0	0	0	0	0	0	0	0	0	0	0	0	0	0	0	0	0	0	0	0		
8	CSFLFFIINTQCVTH	0	0	0	0	0	0	0	0	0	0	0	0	0	0	0	0	0	0	0	0	0	0	0	0	0	0	0	0	0	0	0	0		
9	SFLFFIINTQCVTHE	0	0	0	0	0	0	0	0	0	0	0	0	0	0	0	0	0	0	0	0	0	0	7	0	0	0	0	28	0	0	0	0		-0.37
10	FLFFIINTQCVTHES	0	0	0	0	0	0	0	0	0	0	0	0	0	0	0	0	0	0	0	0	0	0	0	0	0	0	0	0	0	0	0	0		
11	LFFIINTQCVTHESY	0	0	0	478.25	0	0	0	7	0	0	0	0	0	3	0	0	0	0	0	0	0	0	0	0	0	0	0	20	0	0	0	0		1.35
12	FFIINTQCVTHESYQ	0	0	0	0	0	0	0	2.5	0	0	0	0	0	0	0	0	0	0	5.75	0	0	0	0	0	0	0	0	0	0	0	0	0		-0.41
13	FIINTQCVTHESYQE	0	0	0	728	0	6	58	11.5	33	0	0	0	64	121.5	62.5	0	0	10.5	37	85	0	0	0	0	0	0	4	140.5	0	0	0	0		0.92
14	IINTQCVTHESYQEL	0	0	0	1581	0	0	23	0	0	0	0	0	0	124.5	0	0	0	0	16	0	0	0	0	0	0	0	0	48.5	0	0	0	0		1.31
15	INTQCVTHESYQELV	0	0	0	0	0	0	24	0	0	0	0	0	0	62	0	0	0	0	0	42	0	0	11	0	0	0	0	41	195.5	0	0	0		-0.27
16	NTQCVTHESYQELVK	0	0	0	0	0	0	0	0	0	0	0	0	0	0	0	0	0	0	0	0	22	0	0	0	0	0	0	0	0	0	0	0		-0.41
17	TQCVTHESYQELVKK	0	0	0	0	0	0	0	0	0	0	0	0	758	0	0	0	384.5	0	0	0	30.5	0	0	0	0	0	0	0	0	1835.5	0	0		-0.31
18	QCVTHESYQELVKKL	0	0	0	0	0	0	0	0	0	0	0	0	128.5	0	0	0	179.5	362.75	0	1784.5	0	0	0	0	0	0	0	0	0	0	0	0		-0.34
19	CVTHESYQELVKKLE	0	0	0	0	0	0	0	0	516	0	74.5	598.5	464	497	0	1891	8481.25	4361.25	3261.5	12991	657	0	900.25	544.5	0	0	0	0	0	2039	239.5	705.25		-0.08
20	VTHESYQELVKKLEA	68	0	0	0	0	0	0	0	0	0	0	151.5	112	348.25	32	2053.25	7407.25	5380.5	3724	11391	901	0	340	576	0	0	0	0	0	1232	25	623		-0.10
21	THESYQELVKKLEAL	0	0	0	0	0	0	0	0	0	0	0	0	0	0	0	1325.75	4358	2438	2017.5	6523	588	0	64	172.5	0	0	0	0	0	0	0	249.25		-0.15
22	HESYQELVKKLEALE	382.75	0	0	306	0	0	0	0	100	0	0	353.5	0	241	31.5	1717	5201.5	4453.25	4135.25	4440	927.5	29.5	139	422.75	0	0	0	0	0	1290.25	70.5	563		-0.11
23	ESYQELVKKLEALED	797.5	0	0	193	74	27.5	26.5	15.5	291.25	61.5	127.5	402.5	382	215	35	1282.5	4600	3672.5	4160.5	5630	681	43	217.5	482.25	0	0	0	150.5	94	1473	0	383.5		-0.15
24	SYQELVKKLEALEDA	362.5	0	0	0	0	0	0	0	0	0	0	0	0	0	0	499.5	1624.5	1839.25	1348.5	2882	451.25	0	23.5	0	0	0	0	0	0	254.25	0	0		-0.25
25	YQELVKKLEALEDAV	295.5	1.5	0	580.25	0	6	0	0	326.5	0	0	36.5	0	230.5	0	1933.25	4010	1971.5	1201.5	10617.5	427.5	0	0	527.75	0	0	15.5	0	0	0	0	556.5		-0.32
26	QELVKKLEALEDAVL	115.5	0	0	772.25	0	0	0	0	0	0	0	0	0	0	0	0	0	0	0	0	0	0	0	0	0	0	0	0	0	0	0	0		1.94
27	ELVKKLEALEDAVLT	149.5	0	0	858	0	0	0	0	0	72.5	0	0	333.75	107	25.5	0	447	598.25	0	271	0	0	42.5	0	0	0	0	0	0	0	0	0		0.30
28	LVKKLEALEDAVLTG	132	0	0	757.75	0	0	0	0	0	0	0	0	0	0	0	0	0	553	0	0	0	0	0	0	0	0	0	0	0	0	0	0		0.93
29	VKKLEALEDAVLTGY	118.5	13	0	625.5	0	0	0	0	0	0	0	0	166.5	0	0	0	0	482	0	0	15	0	0	0	0	60	14.25	0	0	0	0	0		0.78
30	KKLEALEDAVLTGYS	168.25	21.5	0	873.5	0	0	0	0	0	0	0	0	16	0	0	0	0	456	0	100.5	38	0	0	0	0	301.5	0	0	0	0	0	0		1.08
31	KLEALEDAVLTGYSL	199.5	85.75	0	606	0	2472.75	19.5	0	0	28.5	0	0	0	0	0	0	0	342.75	152.5	0	28	0	0	0	0	82.5	0	0	0	0	0	0		1.81
32	LEALEDAVLTGYSLF	184	65	0	0	16.5	0	19	0	0	0	0	0	0	72.5	0	0	208	216	116.5	0	0	0	0	0	0	0	0	47	0	0	0	0		-0.11
33	EALEDAVLTGYSLFQ	0	0	0	0	229	0	0	0	0	184.5	0	0	585.5	322.5	0	0	0	0	0	0	0	0	7.5	0	0	0	0	3.5	0	0	0	0		-0.28
34	ALEDAVLTGYSLFQK	0	0	0	0	0	0	0	0	0	0	0	0	2287.25	0	0	0	0	0	0	0	0	0	0	0	0	0	0	0	0	0	0	419.75		-0.38
35	LEDAVLTGYSLFQKE	428	0	0	0	0	0	10	0	812	90.25	0	2114.5	1870.25	222.5	61.5	7094	12082.75	6897.25	941.5	16867.25	1077.5	0	1539.75	277.25	370	45	117	128.5	195.5	949.25	900.75	0		-0.09
36	EDAVLTGYSLFQKEK	1.5	0	0	0	0	0	0	0	0	52.25	0	1072	343.75	156	85	4449	5918.25	3869.25	164.25	6608.5	0	242	369	0	0	0	13	0	0	998.5	0	0		-0.09
37	DAVLTGYSLFQKEKM	0	0	0	0	0	0	0	0	0	0	0	349	0	0	0	2914.25	3887	2999.25	25.25	3931.5	15.5	0	115	0	0	0	0	0	0	325.5	2.5	0		-0.14
38	AVLTGYSLFQKEKMV	0	0	0	0	0	0	0	0	0	0	0	380.75	0	0	0	3690.5	5688	5762	453.75	18409	1013.5	0	186.25	0	0	0	0	0	0	0	0	329.5		-0.24
39	VLTGYSLFQKEKMVL	0	0	0	0	7.5	0	0	0	0	7.5	0	317	0	0	0	3628.75	4737.5	3340	231.5	9192.5	0	0	154	0	0	0	32.5	0	0	546.75	33.75	294.75		-0.18
40	LTGYSLFQKEKMVLN	0	0	0	0	0	0	0	0	0	0	0	158	0	0	0	1689.5	1437.25	2073.5	72	6255.75	351	0	0	0	0	0	0	0	0	260	0	0		-0.24
41	TGYSLFQKEKMVLNE	0	630.5	0	966.25	14	0	0	0	0	0	0	255	0	0	0	1565	1929.25	2004.5	51	7305	351.5	0	0	0	0	0	0	0	0	199.5	0	0		-0.49
42	GYSLFQKEKMVLNEE	22.5	0	30.5	789.25	52	0	0	0	29	0	0	167	299	0	0	901.5	1502.75	2218.25	10.5	3600	290.75	0	0	0	0	0	0	0	0	77.75	0	263		-0.41
43	YSLFQKEKMVLNEEE	50.5	0	250	1271	83	18.5	31	45.5	85	0	0	316	451.5	116	36.5	258.5	608.75	1683.75	0	1145	94.5	66	429.5	154	0	0	36	220.5	61	153.5	0	2564		-0.53
44	SLFQKEKMVLNEEEI	170	978.5	1331.25	0	70.5	22.75	0	0	17.5	0	0	0	0	74.5	306.5	0	310	1379	0	0	0	0	114.5	167	15.5	0	0	134.5	235	0	0	2384.5		0.15
45	LFQKEKMVLNEEEIT	68	1614.75	929.5	2407.75	33	6	0	0	0	361	136.75	0	332.5	412	59.5	442	7266.25	2218	164	448.25	526	278	1146	419.25	0	17.25	810.5	0	316.5	543.5	678.5	2837		-0.34
46	FQKEKMVLNEEEITT	411.5	2398.5	889.25	3693.25	0	0	0	0	0	1062.5	341.5	0	252	708.25	108	382	7145.5	1679.75	121.5	0	621	428.5	2085.75	788	0	181.5	1928.5	0	789.5	494.5	2615	3584.25		-0.13
47	QKEKMVLNEEEITTK	208	2151	254	18030.75	0	0	0	0	0	476.25	0	2	0	645	33	388	6130	2848	128	0	524.75	231.5	2782.75	535	0	127.75	2068	0	623.25	1249	4810.5	1571		0.96
48	KEKMVLNEEEITTKG	116	1547.5	89.25	24364.75	0	0	0	0	0	420.5	0	0	0	417.5	0	234.5	6203.5	1661.5	103.5	0	439	229.5	1463.5	0	0	169	1526	0	133.5	837	2931.25	1298.25		1.16
49	EKMVLNEEEITTKGA	78.5	1556.75	0	21720.25	0	0	0	0	0	518.5	347.5	0	666	837	23	435	6425	916.25	140.5	0	437.5	0	1566.25	106.5	0	0	1235	0	589	1187	2245.5	941		1.10
50	KMVLNEEEITTKGAS	113.5	1799	0	6255.5	0	0	0	0	0	506.5	345.75	0	0	806.75	0	344	6798.25	933.5	144.75	0	628.5	0	1593	0	0	216.5	2447.5	0	148	1537.25	2079.75	1782.75		0.11
51	MVLNEEEITTKGASA	230.25	0	174	8777.5	0	0	0	0	0	0	0	0	0	38	0	0	0	3418.5	0	0	0	0	465.5	146	0	0	0	32	0	0	0	3441.5		0.79
52	VLNEEEITTKGASAQ	0	859.25	0	15960	0	0	0	0	0	0	0	0	0	0	0	0	0	1617.25	0	0	0	0	188	0	0	0	0	0	0	0	0	495		1.48
53	LNEEEITTKGASAQS	24035.25	65017	126.25	41900.5	0	48	0	0	0	0	0	0	0	0	0	0	0	949.5	0	0	0	0	0	0	0	0	0	0	0	742	0	0		5.57
54	NEEEITTKGASAQSG	8633	17780	0	8807	0	0	0	0	0	0	0	6	0	0	0	0	0	678.25	0	0	0	0	0	0	0	0	0	0	0	102	0	0	2	5.68
55	EEEITTKGASAQSGA	9066.25	29855.75	1284.75	10163.75	0	102	0	0	0	223.5	0	0	1555.25	71.5	0	1745.5	0	4415	0	0	0	0	0	0	0	0	0	0	0	0	359	0		4.30
56	EEITTKGASAQSGAS	14184.5	27786.5	63238	14216.25	0	62.5	0	0	0	91	0	0	1575.5	145	1271.5	2137.25	0	7090.75	0	620	0	0	0	15.75	0	135	0	0	0	0	420	182		5.90
57	EITTKGASAQSGASA	28086.25	41352.75	63238	11742.25	0	0	0	0	0	160.25	0	0	0	0	1631	6014.25	132	10779.25	0	625	0	0	0	0	0	167	0	0	0	84.75	364	178.5		7.61
58	ITTKGASAQSGASAQ	8001.5	16070.25	63238	7694.5	0	0	0	0	0	170	0	0	0	0	1254.5	4028.25	0	11097	0	355.5	0	225.5	0	0	0	183	0	0	16	0	286.5	144		3.42
59	TTKGASAQSGASAQS	54852.75	65017	63238	6758.5	0	0	0	0	0	388.25	0	0	75.5	29.5	471	6471.25	343.5	13129.5	0	738	0	372.75	0	273.5	0	163	36.5	0	0	89	503	499		8.02
60	TKGASAQSGASAQSG	16959.25	49704.5	48197	2902	0	0	0	0	0	0	0	0	19	0	0	5183.5	1128	4974	0	509.5	0	0	0	13.5	19.75	0	0	0	0	396.5	110	0		5.79
61	KGASAQSGASAQSGA	20885.5	54595	30160.5	1710.5	12.5	0	0	0	0	207.25	0	0	177.5	0	139	9879.75	897.5	9235.25	0	670.5	0	0	0	129	0	852	0	0	0	190	339	742		5.13
62	GASAQSGASAQSGAS	37070.5	54595	45435	0	13	0	0	0	37	391.5	0	0	264	87	1919	10788.75	1995.75	9416.75	0	1712	0	154.5	0	262.5	0	408.5	0	0	0	436	534.5	936.25		6.30
63	ASAQSGASAQSGASA	40884	54595	45435	0	0	0	0	0	0	454	0	0	381.5	87	2023	11797.5	4048	6299	0	1311.75	0	325.5	0	252.5	0	516	0	0	0	663.5	479	1033.5		6.48
64	SAQSGASAQSGASAQ	19574.5	38892.75	45435	0	0	0	0	0	0	175	0	0	243	115	1390	10942.5	1893.5	7995.75	0	1484	0	26.75	0	273	0	142	0	0	0	438.5	562.75	494		5.29
65	AQSGASAQSGASAQS	59385	54595	45435	91.5	0	0	0	0	0	349.5	0	0	126.25	110.5	974	10484.5	2813	9217.25	0	688.75	0	0	0	297	0	229	0	0	0	272	215.5	691.75		6.66
66	QSGASAQSGASAQSG	31146.25	54595	45435	217.5	0	0	0	0	0	133.5	0	0	72.5	55	197	6908.5	1936	7188.5	26	1002	0	0	0	97.5	0	0	0	0	0	160.5	283	468.5		6.30
67	SGASAQSGASAQSGA	20644.5	54595	38784	0	0	0	0	0	0	188.75	0	0	249	67	398.5	12072	902.75	10548.5	0	671	0	0	0	0	0	20.5	0	0	0	244	504.5	1030.25		4.98
68	GASAQSGASAQSGAS	31433.5	54595	45435	0	0	0	0	0	17	528.75	0	0	348.75	89	2556.5	11743.25	1501	9303.5	0	1946.5	0	191.25	0	255.5	0	0	0	0	0	427.25	395	1000		5.94
69	ASAQSGASAQSGASA	59385	54595	45435	0	0	0	0	0	48	685.5	0	0	435.5	235.5	2714.25	12097.5	1987.75	12515	9	1737.75	0	978	0	538.5	0	435.75	0	0	0	677.5	749.25	1345.5		6.41
70	SAQSGASAQSGASAQ	22663.5	34931	45435	0	0	0	0	0	0	441.5	0	0	252	171.5	1789	9596.25	986	13414.25	14.5	1960.5	0	315.5	0	224.75	0	386.25	0	0	0	365	498.5	554.5		5.27
71	AQSGASAQSGASAQS	59385	54595	45435	141.5	0	0	0	0	0	0	0	0	138.5	133	1090.25	10031.75	2613.5	13864.5	0	1698.5	0	719.25	0	225.5	0	264.5	45.5	0	0	211	306	597.5		6.48
72	QSGASAQSGASAQSG	26057.5	50965.5	45435	135.5	0	0	0	0	0	234	0	0	100.5	0	223.5	7355.5	1638	9670.5	0	1026.25	0	0	0	123	0	0	19.75	0	0	282.5	201.5	551.25		5.90
73	SGASAQSGASAQSGA	26044.5	54595	45435	0	0	0	0	0	0	296.5	0	0	286	90	413	11866	634.5	16065	0	1079.75	0	159	0	89	0	0	0	0	0	395.75	478.75	1210.5		5.26
74	GASAQSGASAQSGAS	42739	54595	45435	0	0	0	0	0	0	357	0	0	451.25	120	1737.5	13736.75	895.5	15334.5	0	2064.75	7	500.25	0	270	0	210.5	0	0	0	667.5	756	796		6.08
75	ASAQSGASAQSGASA	46475.25	54595	45435	0	0	0	0	0	0	455	0	0	581.25	145.5	2664.5	11727.5	925	14911	0	1643	0	1286.75	0	502	0	378.75	0	0	10	488	667.25	1022.5		6.27
76	SAQSGASAQSGASAQ	15229.25	22335	45435	0	0	0	0	0	0	300.5	0	0	122.5	0	2501.5	10492.5	0	11228.75	0	1643.25	0	0	0	0	0	330	0	0	0	364.25	480.25	456.5		4.08
77	AQSGASAQSGASAQS	59385	54595	45435	326.75	0	0	0	0	77	444	0	0	220	204	800.5	10274	1686	14387.75	0	1202.5	0	376.5	0	463.5	0	175	0	0	0	274.5	287.25	514		6.49
78	QSGASAQSGASAQSG	27009.5	54595	45435	0	0	0	0	0	0	105	0	0	112	0	271	7060	898.75	8412.5	15.25	692.5	0	0	0	225	0	5	0	0	0	0	229.5	354.5		5.93
79	SGASAQSGASAQSGA	19153.5	54595	38540.25	0	0	0	0	0	0	280	0	0	313	70.5	341.5	10195.25	332.5	12074.25	0	541.25	0	0	0	142	0	14	0	0	0	453.5	716.5	1064.25		4.85
80	GASAQSGASAQSGAS	50507.25	54595	45435	0	0	0	0	0	0	547.75	0	0	327.5	68.5	1616.25	13645	892	14364.75	0	1643	0	274.5	0	233	0	407	0	0	0	395	712	737.5		6.29
81	ASAQSGASAQSGASA	48931	54595	45435	0	0	0	0	0	0	789.75	0	0	491	0	3395	10833.75	1297	14331.75	0	1673.25	0	1051	0	411	0	569.5	0	0	0	685.5	809.5	1479.75		6.37
82	SAQSGASAQSGASAQ	25131.5	33285	45435	0	0	0	0	0	0	244	0	0	276	121	1720	8171.75	826.75	14925	0	1538	0	480.5	0	243.5	0	438.5	0	0	0	601.5	600.75	432.5		5.38
83	AQSGASAQSGASAQS	59385	54595	45435	115.5	0	0	0	0	0	428.25	0	0	226	138.5	653.75	9600.5	1845.75	15524.5	0	1477	0	428.5	0	384.5	0	200	0	0	0	355.5	330	500		6.42
84	QSGASAQSGASAQSG	24069	22964	45435	0	0	0	0	0	0	98.5	0	0	62.5	24	189.5	7031	1132.5	13853.75	36	914.5	0	150	0	0	0	0	0	0	0	140	190	309		4.92
85	SGASAQSGASAQSGT	23507.5	54595	45435	167	0	0	0	0	0	0	0	0	296.5	194.5	0	8904.5	635.5	13998	0	776	0	0	0	285.25	0	0	0	0	0	235.5	372.75	682.75		5.33
86	GASAQSGASAQSGTS	23511.75	54595	45435	0	0	0	0	74	0	0	0	0	145.5	0	396.5	10264	604.5	15655.25	5.75	736	0	250	0	172	0	0	0	0	0	234.5	291	558.5		5.16
87	ASAQSGASAQSGTSG	14229.75	33017.75	45435	0	0	0	0	53.5	0	0	0	0	139	0	171	4407	226	9459.5	0	325.5	0	0	0	58.75	0	0	0	0	0	214	375	486		4.90
88	SAQSGASAQSGTSGP	12383.75	30665.75	45435	161.5	0	0	0	41.5	0	0	0	0	70	83	125.25	2937.75	0	8845.5	0	183.5	0	39.25	0	0	0	0	0	0	0	0	48	50.5		4.73
89	AQSGASAQSGTSGPS	3447.25	9166.25	30379	0	0	0	0	22.5	0	631	0	0	0	0	0	914	0	3514.75	0	0	0	0	0	0	0	0	0	0	0	0	92.5	43.5		2.95
90	QSGASAQSGTSGPSG	4303.75	6545	24417.75	0	0	0	0	9.5	0	191	0	0	0	0	0	470	0	2170	0	0	0	0	0	0	0	0	0	0	0	0	0	0		3.19
91	SGASAQSGTSGPSGP	1394.5	3236.75	24297.75	703.25	0	0	0	0	0	265	0	0	0	0	0	766.5	45	5108	0	0	0	0	0	0	0	0	0	0	0	0	73.5	79		2.02
92	GASAQSGTSGPSGPS	0	1114.25	23178.5	0	0	0	0	0	0	348.75	0	0	0	0	0	334.5	426.5	1950.75	0	0	0	0	0	0	0	0	0	0	0	0	93.5	0		1.47
93	ASAQSGTSGPSGPSG	680.75	915.75	4377	0	0	0	0	4.5	0	0	0	0	0	0	0	0	261	637.5	0	0	0	0	0	0	0	0	0	0	20	0	159.5	0		2.70
94	SAQSGTSGPSGPSGT	0	746	11297.75	1854.5	0	0	0	12	0	0	0	0	0	0	0	0	414.25	0	0	0	0	0	0	0	0	0	0	0	0	0	0	0		2.28
95	AQSGTSGPSGPSGTS	0	0	0	741.5	0	0	0	12.5	0	0	0	0	0	0	0	0	561.5	0	0	0	0	0	0	0	0	0	0	0	0	0	0	0		0.51
96	QSGTSGPSGPSGTSP	647	0	63933	573.25	0	0	0	0	0	305	0	0	38.5	210	0	57.5	398.25	0	0	0	0	118.5	0	0	0	0	0	0	0	249.5	0	165.25		1.43
97	SGTSGPSGPSGTSPS	516.5	0	63933	0	0	0	0	0	0	0	0	0	0	65.5	0	159.5	375.25	0	0	0	0	82.5	0	9.5	0	0	0	0	0	279.25	0	0		1.40
98	GTSGPSGPSGTSPSS	0	0	63933	1004.5	0	0	0	0	0	0	0	136.5	0	0	0	174	0	0	0	100	0	94	0	0	0	0	0	0	0	141.5	0	189		1.43
99	TSGPSGPSGTSPSSR	0	0	63933	314.5	0	0	0	0	0	0	0	244.5	0	13	21	136	0	1411.75	0	2218	15.75	0	0	0	0	0	0	0	0	493.5	36.5	233.5		1.35
100	SGPSGPSGTSPSSRS	0	0	63933	0	0	0	0	0	0	0	0	884.25	0	0	0	139.5	0	1639.5	0	2586	14	0	0	1.5	0	0	0	0	0	317	34	137.5		1.33
101	GPSGPSGTSPSSRSN	0	0	63933	154.5	15	0	7.5	0	0	0	0	0	0	0	0	126.5	0	1184.25	0	2150	0	0	0	0	0	0	0	0	0	0	0	89.75		1.35
102	PSGPSGTSPSSRSNT	0	0	63933	0	0	0	0	0	0	0	0	0	0	0	134.5	274.5	0	837.75	0	1240	0	0	1.5	0	0	0	0	0	0	0	0	0		1.35
103	SGPSGTSPSSRSNTL	0	0	63933	218.5	0	0	0	0	0	0	0	123	0	0	0	0	0	0	0	0	0	0	0	0	0	0	0	0	0	0	0	0		1.38
104	GPSGTSPSSRSNTLP	0	0	22246.75	0	0	0	0	0	0	0	0	581	0	0	0	0	0	0	0	0	0	0	0	0	0	0	0	0	0	0	0	0		1.36
105	PSGTSPSSRSNTLPR	0	21.75	12883.75	0	0	0	0	0	0	0	0	3674.25	0	0	99	0	0	0	0	0	1.5	0	0	0	0	0	0	0	0	120.5	0	0		1.12
106	SGTSPSSRSNTLPRS	0	0	0	0	0	0	0	0	0	0	0	5685.5	0	89	0	0	0	0	0	0	0	0	0	0	0	0	0	0	0	175	0	0		-0.40
107	GTSPSSRSNTLPRSN	0	0	0	0	0	0	0	0	0	0	0	2190.5	0	0	0	0	0	0	0	0	0	0	0	0	0	0	0	0	0	80.5	0	0		-0.41
108	TSPSSRSNTLPRSNT	0	0	0	0	0	0	0	0	0	0	0	7217.5	0	0	0	0	0	0	0	0	0	0	0	0	0	0	0	0	0	111.5	0	0		-0.41
109	SPSSRSNTLPRSNTS	0	0	0	0	0	0	0	0	0	0	0	4325.75	0	0	0	0	0	0	0	174	0	0	0	0	157.5	0	0	0	0	10.25	0	0		-0.40
110	PSSRSNTLPRSNTSS	0	0	0	0	0	0	0	0	0	0	0	3949.25	0	0	0	0	0	698.5	0	0	0	0	0	0	0	0	0	0	0	0	0	0		-0.38
111	SSRSNTLPRSNTSSG	0	0	0	0	0	0	0	98.5	0	0	0	1657.25	0	0	0	88	849.5	37	0	0	0	0	0	0	0	0	0	0	0	0	0	0		-0.33
112	SRSNTLPRSNTSSGA	0	0	0	0	0	0	0	0	226	247.5	0	901	431.5	357	179.5	629.5	1037.25	217	0	3464	29	72	0	367.25	0	0	0	0	60.5	183.5	2625.75	2441.75		0.03
113	RSNTLPRSNTSSGAS	0	0	628.5	0	0	0	0	0	74	207.5	0	0	582	489.75	0	924.5	363	374	0	5272.25	123	122	0	1400.5	0	0	49	0	0	153	3157.75	1654		-0.24
114	SNTLPRSNTSSGASP	0	0	0	0	0	0	0	0	6310.25	88	0	6	303.25	202	86	877.5	14291	277	0	3424.75	46.5	112.25	0	742	0	0	45.5	0	35.5	179.5	2824.75	1461.75		-0.20
115	NTLPRSNTSSGASPP	0	0	258	0	0	0	0	0	4883.75	18.75	0	8.5	337.25	223.25	0	852	21612.5	219	0	2663.75	69.5	24	0	826	0	127	33	0	0	98.5	1688.5	1082		-0.31
116	TLPRSNTSSGASPPA	0	0	253	0	0	0	0	0	4800.75	0	0	0	282	0	14	661.75	18377	129.5	0	2057.5	0	0	0	836.25	0	0	0	0	0	107.5	2002	591.75		-0.31
117	LPRSNTSSGASPPAD	163	0	912.5	1132.5	0	0	0	0	2626	0	0	164	237.5	167.5	0	906.5	24765.25	305	0	2226	69.5	0	4	643.75	0	0	140.5	0	0	95.5	2185	614.5		-0.43
118	PRSNTSSGASPPADA	0	0	464.5	3821.75	0	0	0	0	2126.5	0	0	0	0	0	0	510.5	16196.75	140.5	0	1053.5	12.5	12.75	0	573	0	0	187	0	0	81	772.75	0		-0.54
119	RSNTSSGASPPADAS	0	0	0	0	0	0	0	0	2063	58.5	157.5	0	231	89.5	0	281.25	17814.5	0	0	880	0	0	0	1094.5	0	9	14.75	0	0	34	775	439		-0.34
120	SNTSSGASPPADASD	0	0	101.5	3820	32	0	17	160	2170.5	0	13799.5	0	196.5	141	0	285	21462.75	157.5	50	854.5	12.25	130	60.5	0	0	0	75.5	0	0	0	1056.5	4440.25		-0.44
121	NTSSGASPPADASDS	0	0	0	0	0	0	0	21	927.5	0	340.5	0	0	0	0	0	13852	0	0	0	0	0	0	0	0	0	0	0	0	0	0	1503.5		-0.37
122	TSSGASPPADASDSD	0	0	0	6400.5	19.5	0	23	0	805.5	0	88	0	0	0	0	178.5	0	0	65	0	0	0	0	0	0	0	0	25.5	0	0	0	5333		0.38
123	SSGASPPADASDSDA	0	0	0	1644	110	0	29.75	0	375	0	94.5	0	0	0	0	0	0	0	135	0	0	0	0	0	0	0	9.5	0	0	0	0	406		0.94
124	SGASPPADASDSDAK	0	0	0	2685	0	0	0	0	0	0	0	0	0	0	0	0	0	0	64.5	0	0	0	0	0	0	0	0	0	0	0	0	834.75		1.08
125	GASPPADASDSDAKS	0	0	0	0	0	0	0	0	0	0	0	0	0	0	0	0	0	0	0	0	0	0	0	0	0	0	0	0	0	0	0	1138.5		-0.41
126	ASPPADASDSDAKSY	0	0	0	1406.25	0	0	0	0	0	0	0	0	0	0	0	0	0	0	0	0	0	0	0	0	0	0	0	12	7.5	0	0	1280		0.35
127	SPPADASDSDAKSYA	0	0	0	0	0	0	0	0	0	0	0	0	0	0	0	0	0	0	0	0	0	0	0	0	0	0	0	0	0	0	0	122		-0.41

Color code

LOD score ≥ 3

organization of MSP1
conserved
dimorphic
oligomorphic

128 PPADASDSDAKSYAD	0	0	0	1247.5	8.5	15	27	12	37.5	0	82.5	0	103.5	0	0	0	58.75	0	52	0	0	0	0	0	0	0	84	162.5	0	0	0	2191		-0.22
129 PADASDSDAKSYADL	0	0	0	919.5	0	0	0	0	44.5	0	55	0	0	0	0	0	0	0	0	74	4	0	12.5	0	0	0	782	65	0	0	0	872		-0.06
130 ADASDSDAKSYADLK	0	6852.75	0	0	0	0	0	0	0	0	95.75	0	0	0	0	0	0	0	0	0	0	0	0	0	0	0	0	0	0	0	0	452.5		1.33
131 DASDSDAKSYADLKH	0	0	0	0	0	0	0	0	0	0	0	0	0	0	0	0	0	0	0	0	0	0	0	0	0	0	0	0	0	0	0	0		
132 ASDSDAKSYADLKHR	0	0	0	0	0	0	0	0	0	0	283.75	0	0	0	0	0	0	0	0	0	0	0	0	0	0	0	0	0	0	0	0	0		-0.41
133 SDSDAKSYADLKHRV	0	0	0	0	0	0	0	52	0	0	8087	0	0	0	0	0	0	0	0	0	0	0	0	0	0	0	0	0	18	0	0	0	3	-0.41
134 DSDAKSYADLKHRVR	0	0	0	0	0	0	0	0	0	126.5	62.5	0	0	0	0	0	0	0	0	0	0	0	0	0	0	0	0	0	0	0	0	0		-0.34
135 SDAKSYADLKHRVRN	0	0	0	6222.5	0	0	0	0	0	0	0	0	0	0	0	0	0	0	0	0	0	0	0	0	0	0	0	0	0	0	0	0		1.37
136 DAKSYADLKHRVRNY	0	0	0	1077	0	0	0	0	0	0	0	0	0	0	0	0	0	0	0	0	0	0	0	0	100	0	0	0	0	0	0	0		1.32
137 AKSYADLKHRVRNYL	0	0	0	0	0	0	0	0	0	0	0	0	0	0	0	0	0	0	0	0	0	0	0	0	63	139.5	0	0	0	0	0	0		-0.35
138 KSYADLKHRVRNYLF	0	0	0	0	0	0	0	0	0	0	0	0	0	0	0	0	0	0	0	0	0	0	0	0	16.5	1351	0	0	0	0	0	0		-0.41
139 SYADLKHRVRNYLFT	0	0	0	0	0	0	0	0	0	0	0	0	0	0	0	0	0	0	0	0	0	0	0	0	0	0	0	0	0	0	0	0		
140 YADLKHRVRNYLFTI	0	0	0	0	0	0	0	0	0	0	0	0	0	0	0	0	0	0	0	0	0	0	0	0	0	0	0	0	0	0	0	0		
141 ADLKHRVRNYLFTIK	0	0	0	0	0	0	0	0	0	0	0	0	0	0	0	0	0	0	0	0	0	0	0	0	42.5	0	0	0	0	0	0	0		-0.41
142 DLKHRVRNYLFTIKE	0	0	0	0	0	0	0	0	0	0	0	0	0	0	0	0	0	0	0	0	0	0	0	0	0	0	0	0	0	0	0	0		
143 LKHRVRNYLFTIKEL	0	0	0	0	0	0	0	0	0	0	0	0	0	0	0	0	0	0	0	0	0	918.5	0	0	0	0	0	0	0	0	0	0		-0.41
144 KHRVRNYLFTIKELK	0	0	0	0	0	0	0	2.5	0	0	0	9	0	0	0	0	0	0	0	0	0	0	0	153.5	842.5	1072.25	0	0	0	0	0	0		-0.30
145 HRVRNYLFTIKELKY	0	0	0	0	0	0	0	0	0	0	0	12	0	45	0	0	0	0	0	0	0	0	0	0	0	0	0	0	0	0	0	0		-0.37
146 RVRNYLFTIKELKYP	0	0	0	0	0	0	0	0	0	0	0	0	0	0	0	0	0	0	67.5	0	0	0	0	0	0	0	0	0	0	0	0	0		-0.41
147 VRNYLFTIKELKYPE	0	0	0	0	0	0	0	0	0	0	10.5	0	0	0	0	0	22	0	207	0	0	26.5	0	0	0	0	0	0	0	0	0	0		-0.36
148 RNYLFTIKELKYPEL	0	0	0	5876	0	3.5	0	0	0	0	0	7	0	54	0	0	0	0	533.5	0	0	83	0	0	0	0	0	0	0	0	0	0		1.31
149 NYLFTIKELKYPELF	0	0	0	747	0	0	9	0	102	0	0	29	0	0	0	0	0	0	367.5	0	0	53.5	0	0	0	0	0	0	0	0	0	0		0.75
150 YLFTIKELKYPELFD	6	0	0	1092.75	35.5	16	25.5	4	153.5	7	76	21	28	126.75	175.5	36	0	0	788	62.25	37	17.5	33	0	21.5	0	15	80.5	649.5	5.5	0	28.5		0.07
151 LFTIKELKYPELFDL	0	0	0	1370	18.5	11	30	0	90.5	0	20.5	8.5	0	107	0	0	0	0	741	510	18	57.5	24	0	14.75	0	0	48	176.5	0	0	24		0.44
152 FTIKELKYPELFDLT	0	0	0	0	5	5	0	0	0	0	12.5	0	0	0	0	0	0	0	559.25	379	0	234.25	0	0	0	0	0	0	64.5	0	0	0		-0.25
153 TIKELKYPELFDLTN	0	0	0	966	0	0	27	0	0	0	9.5	0	0	0	0	0	0	136.75	581	0	0	0	0	0	4	0	0	0	143.25	0	0	0		0.59
154 IKELKYPELFDLTNH	0	0	0	0	0	0	0	0	0	0	9	0	0	0	0	0	0	0	326.5	0	0	54	0	0	0	38.5	0	0	40.5	0	0	0		-0.33
155 KELKYPELFDLTNHM	0	0	0	0	0	0	0	0	0	0	0	0	0	0	6	0	0	0	476	0	0	1175.5	0	0	0	349.5	0	0	0	0	0	0		-0.30
156 ELKYPELFDLTNHML	0	0	0	786	0	0	0	0	0	55	0	0	241	294.5	33.25	0	0	0	0	78.25	0	0	238	0	32	0	0	20	24	0	0	0		0.53
157 LKYPELFDLTNHMLT	0	0	0	174	0	0	0	0	0	0	0	0	0	0	0	0	0	0	0	0	0	0	0	0	0	0	0	0	0	0	0	0		1.37
158 KYPELFDLTNHMLTL	0	0	0	582	0	0	0	0	0	0	0	0	0	0	0	0	0	0	0	0	0	0	0	0	0	362.5	0	0	0	0	0	0		0.67
159 YPELFDLTNHMLTLC	0	0	0	858	0	0	13.5	0	0	0	18	0	0	56	0	0	0	0	35.5	36	13	0	9	0	0	0	9.25	35	23	0	0	0		1.23
160 PELFDLTNHMLTLCD	47	0	0	3081	0	0	46.5	0	78.5	0	74.5	0	18.5	108	32.75	0	10	33	0	86.5	0	0	46	9.5	12	0	24	138.5	169	10	0	14		1.29
161 ELFDLTNHMLTLCDN	0	0	0	0	77.5	0	13	0	0	94.5	0	0	219	296.5	6.75	12.5	0	38.5	0	63.75	0	0	99.5	4	0	0	0	85.5	4458.5	0	0	0		-0.37
162 LFDLTNHMLTLCDNI	0	0	0	598.5	0	0	8	0	0	0	0	0	0	0	0	0	0	14.25	0	345.75	0	0	8	0	0	0	0	15	15.5	0	0	0		0.71
163 FDLTNHMLTLCDNIH	0	0	0	0	0	0	0	0	0	0	0	0	0	0	0	0	0	23	0	109.5	0	0	0	0	0	0	0	0	4	0	0	0		-0.37
164 DLTNHMLTLCDNIHG	0	0	0	0	0	0	0	0	0	0	0	0	0	0	0	0	0	0	0	0	0	0	0	0	0	0	0	0	0	0	0	0		
165 LTNHMLTLCDNIHGF	0	0	0	248	0	0	0	0	0	0	0	0	0	0	0	0	17.5	11	8.75	11.5	0	0	0	0	0	0	0	0	0	0	0	0		1.27
166 TNHMLTLCDNIHGFK	0	0	0	0	0	0	0	0	0	0	0	0	0	0	0	0	0	0	0	0	0	0	0	0	0	0	0	0	0	0	0	0		
167 NHMLTLCDNIHGFKY	0	0	0	0	0	0	0	0	0	0	0	0	0	0	0	0	0	0	0	0	0	0	0	0	0	0	0	0	0	0	0	0		
168 HMLTLCDNIHGFKYL	0	0	0	0	0	0	0	0	0	0	0	0	0	0	0	0	0	0	0	0	0	0	0	0	0	0	0	0	0	0	0	0		
169 MLTLCDNIHGFKYLI	0	0	0	0	0	0	0	0	0	0	0	0	0	0	0	0	0	0	0	0	0	0	0	0	0	0	0	0	0	0	0	0		
170 LTLCDNIHGFKYLID	0	32.5	0	74	0	0	12	0	32.5	0	0	0	0	0	17.5	0	54.5	13	6	38.5	7.5	0	0	0	0	0	0	0	17.5	0	0	0		0.60
171 TLCDNIHGFKYLIDG	0	0	0	0	0	0	0	0	0	0	0	0	0	0	0	0	0	0	0	0	0	0	0	0	0	0	0	0	0	0	0	0		
172 LCDNIHGFKYLIDGY	0	0	0	0	0	0	0	0	0	0	0	0	0	0	0	0	0	0	18	0	26.5	0	0	0	0	0	0	0	0	0	0	0		-0.33
173 CDNIHGFKYLIDGYE	0	0	177.25	201	0	0	40.5	0	104	34	0	0	0	353	0	63.5	0	86.5	55	141	32.5	0	24	0	0	37.5	23	184.5	55	0	0	0		-0.10
174 DNIHGFKYLIDGYEE	0	111	494.25	0	80	1	37	0	97	52	0	0	373.5	164.5	27	96	67.5	191	63	121.5	9.5	21	0	0	13.5	32	48	429.25	48.5	0	21.25	26		-0.08
175 NIHGFKYLIDGYEEI	260.5	94	249	0	51	0	33.5	0	0	13.5	0	0	0	106.5	0	79	532.5	44	40	133	24	0	0	0	0	0	0	155	0	0	0	0		0.40
176 IHGF KYLIDGYEEIN	0	0	24.5	26774.5	24	0	12	0	0	0	0	0	26	36.5	0	40	0	30	0	0	32.5	0	0	0	0	0	0	27	0	0	0	0		1.37
177 HGF KYLIDGYEEIN E	0	776	344	27962.75	57	0	27	0	18.25	30.5	0	0	0	119.5	11.25	75.5	171.5	104.5	94	0	19.5	37.5	0	0	0	0	40.5	227.5	37.5	0	0	14.25		1.51
178 GF KYLIDGYEEIN EL	0	59	0	23011	71.5	0	50	0	0	26.25	0	0	42.5	240	0	0	0	39.5	53	0	0	44	42.5	0	13	0	0	227	11.5	0	0	104		1.37
179 F KYLIDGYEEIN ELL	0	15.5	0	22581.25	49	0	68	11	0	0	0	0	0	162.5	0	0	0	0	19.5	466.75	0	5.5	0	0	0	0	0	187.5	12	0	0	438		1.35
180 KYLIDGYEEIN ELLY	0	40.5	234.5	19315	72	19	83	21.75	45.5	16.5	0	0	133	200	0	39.5	0	120	47.5	2406	37.5	29	29.5	0	0	93.5	18.5	219	48	5.25	0	418		1.31
181 YLIDGYEEINELLYK	0	0	0	3193.25	33	0	13.5	3	0	0	0	0	0	0	0	0	0	0	0	0	0	0	0	0	0	0	0	111.5	0	0	0	0		1.35
182 LIDGYEEINELLYKL	0	0	0	4893.75	11	0	38.5	0	0	0	0	0	0	0	0	0	0	0	0	0	0	0	0	0	0	0	0	0	0	0	0	29.5		1.37
183 IDGYEEINELLYKLN	0	0	0	2924.75	11.5	41.5	20.5	0	0	0	0	0	0	0	0	0	0	0	0	2104.75	0	0	0	0	0	0	0	53.5	16	0	0	0		0.55
184 DGYEEINELLYKLNF	0	1199.5	0	2201.25	0	0	24	0	0	0	35	0	0	0	0	0	0	0	0	3212.5	0	0	0	0	0	0	0	81.5	0	0	0	0		0.54
185 GYEEINELLYKLNFY	0	197.5	0	1995	29	0	34	14	0	5.5	0	0	0	15	0	0	260	48	26.5	2696.25	37.5	13	0	0	0	0	25	67.5	24.5	0	0	11.5		0.08
186 YEEINELLYKLNFYF	0	163.5	0	3440.5	28	0	22	10.5	0	0	0	0	1338	75	26	0	371.25	0	41	3144	0	21	0	0	0	0	0	39	25	0	0	0		0.25
187 EEINELLYKLNFYF D	0	405.75	379.5	9558.25	52	92.5	68	19.5	94	232	0	4	3007.5	481	589.5	141.75	1805	186.5	106.5	3946.5	50	60.5	111	42.25	0	0	35.5	284	72.5	0	25	19.5		0.79
188 EINELLYKLNFYF DL	0	127	0	3246	23	0	71	20	0	647	0	0	100.25	198.5	0	0	703.25	88	61	1605.25	0	0	77.5	0	0	0	0	51.5	67.5	0	0	0		0.68
189 INELLYKLNFYFDLL	0	0	0	0	20.25	0	13	0	0	0	0	0	0	0	0	0	1544	452.75	65	1855.75	46.5	0	30.5	0	0	0	0	0	9	0	0	0		-0.27
190 NELLYKLNFYFDLLR	215.5	0	0	0	0	0	12.25	0	0	0	0	0	0	0	0	0	440	0	21.5	641.5	0	0	0	0	0	0	0	0	0	0	0	0		-0.54
191 ELLYKLNFYFDLLRA	0	0	0	0	0	0	0	0	0	89	0	0	36.5	119	0	0	0	0	0	211	0	0	119.25	0	0	0	0	0	0	0	0	0		-0.17
192 LLYKLNFYFDLLRAK	0	0	0	0	0	0	0	0	0	0	0	0	0	0	0	0	0	0	0	0	0	0	0	0	0	0	0	0	0	0	0	0		
193 LYKLNFYFDLLRAKL	0	0	0	0	0	0	0	0	0	0	0	0	0	0	0	0	0	0	0	0	0	0	0	0	0	0	0	0	0	0	0	0		
194 YKLNFYFDLLRAKLN	0	0	0	0	0	0	0	0	0	0	0	0	0	0	0	0	0	0	0	0	0	0	0	0	0	0	0	0	0	0	0	0		
195 KLNFYFDLLRAKLND	0	0	0	281.75	0	0	0	2.75	0	0	0	0	0	0	0	0	0	88	0	0	0	0	11	0	7.5	0	14.25	52	0	0	0	0		0.93
196 LNFYFDLLRAKLNDV	0	0	0	479.5	0	0	0	0	64	0	0	0	0	291.5	0	0	0	26.5	0	50	0	0	0	0	0	0	0	0	0	0	0	0		0.59
197 NFYFDLLRAKLNDVC	0	0	0	281	0	0	0	7	61	0	0	0	0	52.75	0	0	0	0	32	0	0	0	0	0	32	0	9	9.25	16.5	0	0	7.5		0.90
198 FYFDLLRAKLNDVCA	0	0	0	0	0	0	0	0	0	0	0	0	0	0	0	0	0	0	0	0	0	0	0	0	0	0	0	0	0	0	0	0		
199 YFDLLRAKLNDVCAN	0	0	527.5	0	0	0	0	0	0	0	0	0	0	0	0	0	0	0	0	0	0	0	0	0	0	0	0	0	0	0	0	0		1.37
200 FDLLRAKLNDVCAND	0	1236	211.5	0	0	0	0	18.5	49	0	0	0	0	18.75	0	0	74.5	0	0	75	0	0	0	0	0	0	15.5	97	0	0	0	0		1.85
201 DLLRAKLNDVCANDY	0	198	525.5	445.5	21.5	16.5	8	10	78.5	3	0	0	0	136	0	0	156	183.5	32	437	51	0	19	12.25	0	0	0	80	31	0	15	15.5		2.33
202 LLRAKLNDVCANDYC	0	0	385	0	29.5	14	0	0	44	0	12.5	0	0	82	6.75	0	138.5	217.5	169.5	536.5	67	0	10.5	0	3	0	0	0	34	0	17	10.5		-0.33
203 LRAKLNDVCANDYCQ	0	0	114	0	0	0	0	0	0	0	0	0	0	20	0	0	77	64	0	343	0	0	0	0	0	0	0	0	0	0	0	0		-0.50
204 RAKLNDVCANDYCQI	0	0	61.5	0	0	0	0	0	0	0	0	0	0	0	0	0	0	47.75	0	246.5	0	0	0	0	0	0	0	0	0	0	0	0		-0.53
205 AKLNDVCANDYCQIP	0	0	0	0	0	0	0	0	0	0	0	0	0	16	0	0	65	177	0	86	229.5	0	0	0	0	0	0	0	0	0	0	0		-0.21
206 KLNDVCANDYCQIPF	0	0	0	0	0	0	0	3	34	4.25	0	0	0	0	0	0	110	298.5	0	355	103.5	0	7	0	0	245	0	0	0	6.5	0	0		-0.13
207 LNDVCANDYCQIPFN	0	0	62	0	0	0	0	0	0	0	0	0	0	73.5	0	0	11.5	267.5	0	213	160	0	0	0	0	0	0	0	0	0	0	23.5		-0.41
208 NDVCANDYCQIPFNL	0	0	0	0	0	0	0	0	0	0	0	22	0	0	0	0	0	0	0	95.5	0	0	0	0	0	0	0	0	0	0	189.75	74		-0.26
209 DVCANDYCQIPFNLK	0	0	0	0	0	0	0	0	0	0	0	0	0	0	0	0	0	0	0	0	0	0	0	0	0	0	0	0	0	0	0	0		
210 VCANDYCQIPFNLKI	0	0	0	0	0	0	0	0	0	0	0	0	0	0	0	40	0	0	0	0	0	0	0	0	0	0	0	0	0	0	0	0		-0.41
211 CANDYCQIPFNLKIR	0	0	1461	0	0	0	0	0	0	0	0	0	0	0	0	0	0	0	0	0	0	0	0	0	0	0	0	0	0	0	0	0		1.37
212 ANDYCQIPFNLKIRA	0	0	519	0	0	0	0	0	0	0	0	0	0	0	0	0	0	0	0	0	0	0	0	0	0	0	0	0	0	0	0	0		1.37
213 NDYCQIPFNLKIRAN	0	0	0	0	0	0	0	0	0	0	0	0	0	0	0	0	0	0	0	0	0	0	0	0	0	0	0	0	0	0	0	0		
214 DYCQIPFNLKIRANE	0	1084	0	0	68.5	0	0	0	0	0	0	0	0	0	0	0	46.5	0	0	43.5	0	0	0	0	0	0	0	0	0	0	0	0		1.33
215 YCQIPFNLKIRANEL	0	0	0	340.5	42.5	0	0	0	0	0	0	0	0	15.5	0	0	0	0	0	0	0	0	0	0	0	0	56	0	0	0	0	0		1.23
216 CQIPFNLKIRANELD	383	0	0	918	48	0	16	0	20	0	0	23	47.5	47	0	0	0	110	23.5	1805.5	54.5	0	10.5	0	130	0	150.5	0	0	0	0	0		-0.02
217 QIPFNLKIRANELDV	0	0	0	602.5	46.5	0	0	0	0	0	0	0	34	0	0	0	0	0	0	411.5	0	0	5	0	0	0	40.5	0	0	0	0	0		0.56
218 IPFNLKIRANELDVL	0	0	0	227	54.5	0	0	0	0	0	0	0	0	0	0	0	0	0	0	50	0	0	0	0	0	0	0	0	0	0	0	0		1.19
219 PFNLKIRANELDVLK	0	0	0	0	21.5	0	0	0	0	0	0	0	0	0	0	0	0	0	0	0	0	0	0	0	0	0	0	0	0	0	0	0		
220 FNLKIRANELDVLKK	0	0	0	0	0	0	0	0	0	0	0	0	0	0	0	240.5	0	0	0	0	0	0	0	0	0	0	0	0	0	0	0	0		-0.41
221 NLKIRANELDVLKKL	0	0	0	0	0	0	0	0	0	0	0	0	0	0	0	55	0	296	0	0	0	0	0	0	0	0	0	0	0	0	0	0		-0.38
222 LKIRANELDVLKKLV	0	0	0	0	0	0	0	0	0	0	0	0	0	0	0	0	0	256.5	0	0	0	0	0	0	0	0	0	0	0	0	0	0		-0.41
223 KIRANELDVLKKLVF	0	0	0	0	0	0	0	0	0	0	0	0	0	0	0	0	0	63	0	0	30.5	0	0	0	0	300	0	0	0	0	0	0		-0.36
224 IRANELDVLKKLVFG	0	0	0	0	20	0	0	0	0	0	0	0	0	0	0	0	0	0	0	0	0	0	0	0	0	0	0	0	0	0	0	0		
225 RANELDVLKKLVFGY	0	0	0	0	9.25	0	0	0	0	0	0	0	0	0	0	0	0	26	0	0	0	0	0	0	0	0	0	0	0	0	0	0		-0.41
226 ANELDVLKKLVFGYR	0	0	0	0	31.5	0	0	0	0	0	0	0	0	0	0	0	0	0	0	0	0	0	0	0	0	0	0	0	0	0	0	0		
227 NELDVLKKLVFGYRK	0	0	0	0	0	0	0	0	0	0	0	6	0	0	0	0	0	209.25	0	0	0	0	0	0	0	0	0	0	0	0	0	0		-0.41
228 ELDVLKKLVFGYRKP	0	0	0	0	0	0	0	0	0	554.5	0	0	0	137	0	0	0	504.5	0	0	0	0	0	0	0	0	0	0	0	0	0	0		-0.29
229 LDVLKKLVFGYRKPL	0	0	0	0	0	0	0	0	0	0	0	0	0	0	0	0	0	379	0	80	0	0	0	0	0	0	0	0	0	448.5	0	0		-0.30
230 DVLKKLVFGYRKPLD	0	0	0	0	0	0	0	0	0	23	0	0	0	46.5	0	0	0	8	0	66.5	7	0	0	0	0	0	0	0	0	494	0	0		-0.35
231 VLKKLVFGYRKPLDN	0	11	0	1241.75	0	0	0	0	0	0	0	0	0	22.5	0	0	0	0	0	89	0	0	0	0	0	0	0	0	0	490	0	0		0.97
232 LKKLVFGYRKPLDNI	0	50.5	0	0	0	0	0	0	0	0	0	0	0	0	0	0	0	42	0	4239	0	0	0	0	0	64	0	0	0	234	0	0		-0.41
233 KKLVFGYRKPLDNIK	4	117.25	0	3190.5	0	0	9.75	0	0	0	0	18.5	0	50.5	0	118	0	586	0	1345	398.5	0	123	0	0	1092	0	0	0	4425.5	588.75	1458.5		-0.26
234 KLVFGYRKPLDNIKD	0	44.5	591.75	2412.75	0	0	0	0	0	0	0	0	0	0	0	68	0	186.5	0	0	0	0	16.25	0	0	239	0	0	0	1412	0	862		1.07
235 LVFGYRKPLDNIKDN	0	0	0	0	0	0	0	0	42.5	0	0	0	0	0	0	0	0	203.5	0	232	0	0	0	0	0	0	0	0	0	543.5	90.5	369		-0.16
236 VFGYRKPLDNIKDNV	0	0	0	1042.5	0	0	0	0	0	0	0	0	0	0	0	0	0	92.25	0	183	66	0	0	0	0	0	0	0	0	520.5	24.5	432.5		0.45
237 FGYRKPLDNIKDNVG	0	0	934.75	0	0	0	0	0	0	0	0	0	0	0	0	0	0	0	0	0	0	0	0	0	0	0	0	0	0	405	0	222		0.79
238 GYRKPLDNIKDNVGK	0	0	0	0	0	0	0	0	0	0	0	0	0	0	0	0	0	0	0	0	0	0	0	0	0	0	0	0	0	1032.75	96	747.75		-0.31
239 YRKPLDNIKDNVGKM	0	0	0	562.5	0	0	0	0	0	0	0	14	0	0	0	0	0	0	0	0	0	0	0	0	0	0	0	0	0	374.5	0	230.75		0.41
240 RKPLDNIKDNVGKME	811.5	0	0	613.5	0	0	53.5	0	0	0	0	0	0	0	0	0	0	0	0	219	0	0	0	0	0	0	0	0	0	929.75	115.5	0		1.08
241 KPLDNIKDNVGKMED	2843.25	0	743	485	0	0	0	0	0	0	0	0	0	0	0	0	0	0	0	0	0	0	0	0	0	61.5	0	0	0	230.5	37	0		3.14
242 PLDNIKDNVGKMED Y	1928.25	0	0	550.5	0	0	62	0	0	0	20	0	0	0	0	0	0	0	0	38	11	0	0	0	5	0	0	110.25	0	0	0	0		2.37
243 LDNIKDNVGKMED YI	1184.25	0	740	993	0	0	16	0	0	0	0	0	306.5	0	0	0	0	21.5	0	0	0	0	0	0	0	0	30	0	0	0	0	0		6.26
244 DNIKDNVGKMEDYIK	83.5	0	0	0	0	0	0	0	0	0	0	0	266	0	0	0	0	0	0	0	0	0	0	0	0	0	0	0	0	0	0	0		-0.44
245 NIKDNVGKMEDYIKK	0	0	0	0	0	0	0	0	0	0	0	74	1005.5	307.5	0	480	111	0	0	0	0	0	0	0	0	0	0	0	0	0	0	0		-0.26
246 IKDNVGKMEDYIKKN	0	0	644	0	0	0	0	0	0	0	0	0	210.75	0	0	83.5	0	0	0	0	0	0	0	0	0	76	0	0	17.5	0	0	0		0.93
247 KDNVGKMEDYIKKNK	0	115	0	0	0	0	0	0	0	0	0	0	263.5	0	0	28.5	0	0	0	0	0	0	0	0	0	451.5	0	0	0	0	0	0		-0.56
248 DNVGKMEDYIKKNKT	0	55.75	0	200.75	0	0	0	0	0	0	0	0	568	0	0	97	0	33	0	0	0	0	0	0	0	0	0	0	0	0	227.75	0		-0.42
249 NVGKMEDYIKKNKTT	0	80.5	236	0	0	0	0	0	0	0	0	22	694	195	0	307.5	0	0	0	0	0	0	0	0	0	0	0	0	0	0	1240	0		-0.51
250 VGKMEDYIKKNKTTI	454	0	0	0	0	0	0	0	0	0	0	0	1551	470.5	0	554	372.5	1064.25	0	0	0	0	0	0	0	0	0	0	0	0	2757	0		-0.36
251 GKMEDYIKKNKTTIA	433.5	219.75	0	798	0	0	0	0	0	0	0	0	1113.5	116.5	0	526.5	301.25	494	0	0	22	0	0	0	0	0	0	0	0	0	5738.25	0		-0.56
252 KMEDYIKKNKTTIAN	275.25	0	0	0	0	0	0	0	0	27	0	0	416	0	0	303.5	16.5	669.5	0	152.5	0	0	0	0	0	157	0	0	0	0	0	0		-0.56
253 MEDYIKKNKTTIANI	500.25	0	0	0	0	0	0	0	0	0	0	0	0	0	0	488	0	0	0	0	0	0	0	0	0	0	201	0	0	0	0	0		0.16
254 EDYIKKNKTTIANIN	0	0	0	0	23	0	0	0	0	0	0	380	272.75	319	8.75	4273.5	1059	3435.5	10	310.5	1406.5	0	0	721.5	0	179.25	685.25	0	518.5	1341.5	917.75	894.25		0.10
255 DYIKKNKTTIANINE	140.25	871.5	118.25	450.75	0	0	0	0	0	19.5	0	441	136	151	0	4249	1615	4228	19.5	319.5	2259.5	52	535	345.75	0	343.25	2620.5	0	803.5	936	663	1401		-0.23
256 YIKKNKTTI ANINEL	0	21172.5	0	0	0	0	0	0	0	0	0	172	146.5	56.25	0	2095	204.5	1726.5	0	155	703.75	0	0	333	0	107.5	1198	0	453.75	422.25	369	630.75		1.16
257 IKKNKTTIA NINEL I	471.75	20632	0	739	0	0	0	0	0	0	0	196	244.5	80.5	0	1671.5	0	1792	0	0	377.75	0	11.5	415.5	0	366	613.5	0	594	889.5	269	764		1.38
258 KKNKTTIA NINEL IE	14445.5	23665.25	162	1046.5	0	0	0	0	11.5	34	0	616.75	373.5	293	8.5	1963.5	1279	1008	0	1352	898	0	117.5	303.25	0	748	829	0	216	0	0	727		3.31
259 KNKTTIA NINEL IEG	14382	16815.75	0	120	0	0	0	0	0	17	0	172	151.5	0	0	1926.5	760.5	973.75	0	278.5	368	0	56	169.5	0	0	365.5	0	134	789	27.5	387.5		3.35
260 NKTTIA NINEL IEGS	25243	24325	0	227	0	0	7.5	0	0	0	0	225	166	58	0	1936	583.5	1075	386	223	476	0	50.5	69	0	0	232.5	0	0	632.5	65	473.5		3.49

261	KTTIANINELIEGSK	40007	26318.5	0	131.75	0	0	0	0	0	0	0	155.5	0	9	0	1854	704.25	716.75	0	185.75	469.75	0	27.5	0	0	1242.5	122.5	0	89	593.5	0	379.5		3.29
262	TTIANINELIEGSKK	23086.75	20608	0	0	0	0	0	0	0	0	0	0	0	0	0	0	0	0	12	0	125	0	0	0	0	0	18	0	0	832	6	0		3.54
263	TIANINELIEGSKKT	35738.25	33931.5	0	155.5	0	0	0	0	0	0	21	0	0	0	0	164	315	1413.5	473.5	0	0	0	0	0	0	0	140.75	65	52	954.5	0	0		3.55
264	IANINELIEGSKKTI	64939	33893	160.75	561.75	0	0	0	0	0	0	0	6.5	381	0	0	0	238.5	0	409.5	0	102	0	0	0	1.5	0	1669	0	4126.25	608.5	0	0		3.10
265	ANINELIEGSKKTID	47947.75	29894.75	173	0	0	7	52	2	9	0	0	4.5	5.5	0	0	22.5	0	1838.5	535.75	145	317	0	0	63.5	4.5	0	34.5	101.5	72	1010.75	0	0		3.28
266	NINELIEGSKKTIDQ	14722	13596.25	34.75	0	0	0	0	0	0	0	0	35.5	0	0	0	0	0	1541.5	0	82.5	109	0	0	0	0	0	8.5	39	7	526.5	0	0		3.48
267	INELIEGSKKTIDQN	4493.75	708	0	2492.5	0	0	17	0	0	0	0	0	0	0	0	0	0	970	0	139.75	0	0	10.5	0	0	0	0	0	8	1	0	0		3.80
268	NELIEGSKKTIDQNK	0	0	0	0	0	0	0	0	0	0	0	0	4704.5	0	0	4938.75	562.75	900.75	0	421	0	95.75	47.5	218.5	0	0	366	0	0	0	0	0		-0.24
269	ELIEGSKKTIDQNKN	0	0	0	0	81.5	0	0	0	0	176.75	0	0	0	355.5	0	4177.5	0	619.5	0	237.25	0	217	77.5	215	0	0	0	0	0	0	0	0		-0.32
270	LIEGSKKTIDQNKNA	0	0	0	0	0	0	0	0	0	0	0	0	1403.25	0	0	2538	0	269.5	0	57.5	22.5	92	0	68.5	0	0	23	71.5	0	0	0	0		-0.29
271	IEGSKKTIDQNKNAD	0	0	202	0	0	0	0	0	11	0	0	0	3693	0	0	2046.5	0	0	0	0	0	45	0	199.5	0	0	33.5	42	0	0	0	0		-0.38
272	EGSKKTIDQNKNADN	0	0	789	0	0	0	0	0	0	345	0	0	1064.5	108	6.75	1400.75	0	103.75	0	0	0	24	0	0	0	0	0	214	0	0	0	0		-0.45
273	GSKKTIDQNKNADNE	0	280.5	0	870	0	0	3.5	0	34	0	0	0	493.5	0	5	946.75	139	79.5	0	0	0	8.25	0	30	0	0	0	67	0	0	0	0		0.36
274	SKKTIDQNKNADNEE	0	0	146.5	1717.5	24.5	4.5	0	0	40.5	23.75	0	25	1143	38.5	95.5	692	92.5	115.5	0	0	0	35.5	30.5	620	0	0	73.5	270	25.5	0	0	0		0.31
275	KKTIDQNKNADNEEG	0	0	317	430.25	0	0	13.75	0	0	15.5	0	0	1000.25	0	0	219	0	0	0	0	0	0	0	19.5	0	220.75	6.5	98.5	18	0	0	0		-0.05
276	KTIDQNKNADNEEGK	0	0	0	10538.5	0	0	0	0	0	0	0	0	148	0	0	0	0	0	0	0	0	0	0	0	0	0	0	0	0	0	0	0		1.37
277	TIDQNKNADNEEGKK	0	0	0	0	6.5	0	0	0	0	0	0	0	0	0	0	34	0	0	0	0	0	0	0	0	0	0	0	0	0	0	0	0		-0.41
278	IDQNKNADNEEGKKK	0	0	2421	1725	0	0	0	0	0	0	0	0	0	0	0	393.5	0	0	0	0	0	0	0	0	0	0	0	0	0	0	0	0		3.27
279	DQNKNADNEEGKKKL	0	0	3054.75	3696.25	0	0	0	0	0	0	0	0	0	0	0	308	0	0	0	0	0	0	0	0	0	0	0	0	0	0	0	0		3.47
280	QNKNADNEEGKKKLY	0	0	2042	2666	0	0	0	0	0	0	0	0	0	0	0	204.75	0	0	0	0	0	0	0	0	0	0	0	0	0	0	0	0		3.43
281	NKNADNEEGKKKLYQ	0	0	0	1323.5	0	0	0	0	0	0	0	12.5	0	0	0	101	0	0	0	0	0	0	0	0	0	15	0	0	0	0	0	0		1.32
282	KNADNEEGKKKLYQA	0	0	0	1122.25	0	0	0	0	0	0	0	0	0	0	0	0	0	0	0	0	0	0	0	0	0	414	0	0	0	0	0	0		1.01
283	NADNEEGKKKLYQAQ	0	0	225	875	0	0	0	0	0	0	0	8	0	0	0	35.5	0	0	0	0	0	0	0	0	0	0	0	0	0	0	0	0		2.30
284	ADNEEGKKKLYQAQY	0	0	0	563.5	0	0	0	0	0	0	25	0	0	0	0	0	0	0	0	0	0	0	0	0	0	0	0	0	0	0	0	0		1.35
285	DNEEGKKKLYQAQYD	0	0	0	2015.5	16.25	9	13.5	0	70	0	29	56.5	0	0	100.5	119.5	432.5	73.75	0	0	33	3.25	0	0	0	0	4	50.5	20	0	49.5	49.5		1.06
286	NEEGKKKLYQAQYDL	0	0	0	0	10.25	0	0	0	0	0	0	45.5	0	20	0	149.5	331.75	147.25	0	0	0	0	0	0	0	0	0	0	70.5	0	177	217.5		-0.05
287	EEGKKKLYQAQYDLS	0	0	0	0	13.5	87	0	0	0	59.5	0	0	2383	162.5	0	0	179.5	52	37.5	0	0	0	0	0	0	0	0	0	0	0	0	104.5		-0.36
288	EGKKKLYQAQYDLSI	0	413.75	100.5	520.75	0	0	0	0	0	132.5	0	0	0	131	0	90	456.75	153.5	0	0	225.25	0	0	0	0	0	0	0	21	0	70.75	130.5		1.46
289	GKKKLYQAQYDLSIY	0	0	493	643	0	0	0	0	0	0	0	0	0	0	0	0	369	113	0	0	10	0	0	0	0	0	0	0	0	0	18.5	0		2.32
290	KKKLYQAQYDLSIYN	0	0	0	212.5	0	0	0	0	0	0	0	0	0	0	0	0	159.5	102	0	0	0	0	0	0	0	815.5	0	0	0	0	0	0		-0.54
291	KKLYQAQYDLSIYNK	0	0	0	0	0	0	0	0	0	0	0	0	0	0	0	0	0	62	0	0	0	0	0	0	0	1007.5	0	0	0	0	0	0		-0.40
292	KLYQAQYDLSIYNKQ	0	0	0	0	0	0	0	0	0	0	0	0	0	0	0	387.5	0	0	0	0	0	0	0	0	0	0	0	0	0	0	0	0		-0.41
293	LYQAQYDLSIYNKQL	0	0	0	0	0	0	0	0	0	0	0	0	0	0	0	337	0	0	0	0	0	0	0	0	0	0	0	0	0	0	0	0		-0.41
294	YQAQYDLSIYNKQLE	0	0	0	1244.5	0	0	0	0	33.5	0	0	0	0	0	0	524.5	0	0	0	0	0	0	0	0	0	0	0	0	0	0	0	0		0.93
295	QAQYDLSIYNKQLEE	392.5	0	0	309	18	6	0	0	89.5	0	0	0	278	70.5	3	633.25	0	53	0	53	0	0	0	0	0	4.5	24.5	119.5	0	6	0	23.5		0.26
296	AQYDLSIYNKQLEEA	0	0	0	0	0	0	0	0	0	0	0	0	0	20.25	0	643.5	0	0	0	0	0	0	0	0	0	0	0	46	0	2	0	3		-0.39
297	QYDLSIYNKQLEEAH	0	408.75	0	0	0	0	0	0	0	0	0	0	0	0	0	232.5	0	410.5	0	0	0	0	0	0	0	0	0	0	0	0	0	0		0.07
298	YDLSIYNKQLEEAHN	0	195	0	0	0	0	0	0	0	0	0	0	0	0	0	103	0	272	0	0	0	0	0	0	0	0	0	0	0	0	0	0		-0.11
299	DLSIYNKQLEEAHNL	100	923.75	0	368.5	0	0	0	0	0	0	0	0	0	0	0	198	0	197.5	0	0	0	0	0	0	0	0	0	0	0	0	0	0		2.86
300	LSIYNKQLEEAHNLI	0	208.5	0	300	0	0	0	0	0	0	0	0	0	0	0	0	0	51	0	0	0	0	0	0	0	0	0	0	0	0	0	0		3.24
301	SIYNKQLEEAHNLIS	0	0	0	251.5	0	0	0	0	0	0	0	0	0	0	0	0	0	0	0	0	0	0	0	0	0	0	0	0	0	0	0	0		1.37
302	IYNKQLEEAHNLISV	0	0	0	307	0	0	0	0	0	0	0	0	0	0	0	0	0	49.5	0	0	0	0	0	0	0	0	0	0	0	0	0	0		1.26
303	YNKQLEEAHNLISVL	0	0	0	0	0	0	0	0	0	0	0	0	0	0	0	0	0	0	0	0	0	0	0	0	0	0	0	0	0	0	0	0		
304	NKQLEEAHNLISVLE	394.5	38	0	1798	0	0	0	0	91.5	0	0	58.5	0	27	0	108	0	48.5	0	0	0	0	0	0	0	0	40.5	41	65	0	0	0		2.14
305	KQLEEAHNLISVLEK	111.5	0	0	1866.25	0	0	0	0	0	3	0	0	33	0	51.5	0	0	0	0	0	0	0	0	0	0	199	0	0	0	0	0	0		1.50
306	QLEEAHNLISVLEKR	0	0	0	0	2.25	1195.5	0	0	0	0	0	0	0	0	0	0	0	0	0	0	0	0	0	0	0	0	0	0	0	0	0	0		
307	LEEAHNLISVLEKRI	0	0	0	348.5	0	0	0	0	0	0	0	0	0	0	0	0	0	0	0	0	0	0	0	0	0	0	0	0	0	0	0	0		1.37
308	EEAHNLISVLEKRID	0	0	0	553.75	7.5	36.5	11	0	0	1761.75	0	0	2746.5	196.5	0	0	0	0	0	0	0	0	0	0	0	0	0	0	0	0	1	0		-0.51
309	EAHNLISVLEKRIDT	0	339	0	1305.5	0	0	0	0	0	223	0	0	145	133	0	0	0	0	0	0	0	0	0	0	0	0	0	0	0	0	0	0		2.02
310	AHNLISVLEKRIDTL	0	756	0	373.25	0	0	0	0	0	0	0	0	0	0	0	0	0	0	275.5	0	0	0	0	0	0	0	0	0	28	0	0	0		2.46
311	HNLISVLEKRIDTLK	0	1563.5	0	0	0	0	0	0	0	0	0	0	350	0	0	0	0	0	0	0	0	0	0	0	0	0	0	0	0	0	0	0		1.19
312	NLISVLEKRIDTLKK	0	5222.5	0	0	0	0	0	0	0	0	0	0	1278.5	0	0	0	0	239.25	0	0	0	0	0	0	0	0	0	0	0	0	0	0		1.15
313	LISVLEKRIDTLKKN	0	1764.5	0	204.5	0	0	0	0	0	0	0	0	0	0	0	0	0	0	0	0	0	0	0	0	0	0	0	0	0	0	0	0		1.81
314	ISVLEKRIDTLKKNE	0	1283.25	3561	0	0	0	0	0	0	0	0	83	150.25	0	0	0	0	0	0	0	0	0	0	0	0	0	0	0	223	0	0	0		2.59
315	SVLEKRIDTLKKNEN	0	553.5	0	0	0	0	0	0	0	0	0	0	0	0	0	0	0	0	0	0	0	0	0	0	0	0	0	0	0	0	0	0		1.37
316	VLEKRIDTLKKNENI	0	0	150	1246.5	0	0	0	0	0	0	0	0	0	0	0	0	0	0	0	0	0	0	0	0	0	0	0	0	0	0	0	0		1.83
317	LEKRIDTLKKNENIK	0	1174	0	0	0	0	0	0	0	0	0	0	0	0	0	0	0	0	0	0	0	0	0	0	0	0	0	0	0	0	0	0		1.37
318	EKRIDTLKKNENIKK	0	2782.75	21.5	570.5	0	0	0	99.5	0	0	0	0	102.5	0	0	0	0	0	0	0	0	139.5	0	0	0	0	0	0	0	0	0	0		2.13
319	KRIDTLKKNENIKKL	0	2875.5	0	331.75	0	0	4.75	269.5	0	0	0	0	0	0	0	151.5	0	125.25	0	0	0	289.5	0	303.5	0	421.25	0	0	0	0	0	0		1.50
320	RIDTLKKNENIKKLL	0	280.75	23	985	0	0	0	0	0	0	0	0	0	0	0	0	0	0	0	0	0	123	0	0	0	0	0	0	0	0	0	0		2.44
321	IDTLKKNENIKKLLD	0	0	0	0	0	0	0	0	0	0	0	0	0	0	0	0	0	0	0	0	0	0	0	0	0	0	0	0	843.5	0	0	0		-0.41
322	DTLKKNENIKKLLDK	0	0	0	0	0	0	0	0	0	0	0	0	0	0	0	40.5	0	57	0	0	41.5	0	0	0	0	0	0	0	0	0	0	0		-0.25
323	TLKKNENIKKLLDKI	368.25	578.25	0	0	0	0	0	299.5	0	0	0	0	0	0	0	241.25	0	170.5	0	0	96	79	0	0	0	0	0	0	0	0	36	0		2.13
324	LKKNENIKKLLDKIN	0	936.25	0	0	0	0	0	0	0	0	0	0	0	0	0	1133.5	836	1903.25	10	2295	0	0	29	0	0	0	0	0	0	1667	25	0		-0.47
325	KKNENIKKLLDKINE	0	1035	89	480.5	0	0	0	0	36.5	0	0	0	0	0	0	911.75	535	1550.5	0	1557.5	0	0	9.5	0	0	191	0	0	0	0	0	0		-0.21
326	KNENIKKLLDKINEI	0	504.75	0	729.75	0	0	0	0	0	0	0	0	0	0	0	195.75	250.5	405.5	0	700.75	0	17	0	0	98.5	0	0	0	0	0	0	0		0.86
327	NENIKKLLDKINEIK	0	442.25	0	0	0	0	0	0	0	0	0	3	0	0	0	0	0	0	0	0	0	0	0	0	0	0	0	0	0	0	0	0		1.37
328	ENIKKLLDKINEIKN	0	366	0	858.5	0	0	0	0	0	0	0	0	0	14	0	0	0	0	0	0	0	0	0	0	0	0	0	0	0	0	0	0		2.87
329	NIKKLLDKINEIKNP	0	558	0	344.75	0	0	0	0	0	0	0	0	0	0	0	0	0	0	0	0	0	0	0	0	0	0	0	0	0	0	0	0		3.30
330	IKKLLDKINEIKNPP	0	0	0	0	0	0	0	0	0	0	0	0	0	0	0	0	0	0	0	0	0	0	0	0	0	0	8.5	0	0	0	0	0		-0.41
331	KKLLDKINEIKNPPP	0	0	0	0	0	0	0	0	0	0	0	0	0	0	0	0	0	0	0	0	0	0	113	0	0	263.75	0	0	0	0	0	0		-0.35
332	KLLDKINEIKNPPPA	0	0	0	0	0	0	0	0	0	9	0	0	0	0	0	0	0	0	2588.5	0	0	0	61	0	0	0	0	0	0	0	0	0		-0.41
333	LLDKINEIKNPPPAN	0	0	0	0	0	0	0	0	0	0	0	0	0	0	0	0	0	0	26312.25	0	0	0	0	0	0	0	0	0	0	0	0	297.5		-0.41
334	LDKINEIKNPPPANS	0	0	0	0	0	0	0	0	0	0	0	0	0	0	0	0	0	0	25432.75	0	0	0	0	0	0	0	0	0	0	0	0	69.5		-0.41
335	DKINEIKNPPPANSG	0	0	0	0	0	0	0	0	0	0	0	0	0	164.5	0	0	0	0	16070.25	0	0	0	0	0	0	0	0	0	0	0	0	47.5	4	-0.41
336	KINEIKNPPPANSGN	0	0	0	1656	0	0	0	0	0	0	0	0	0	0	0	0	0	0	16684.25	1752.25	0	0	0	0	0	493.5	0	0	0	0	977	41		-0.48
337	INEIKNPPPANSGNT	0	0	0	1851	0	0	0	0	0	0	0	0	0	0	0	0	96.25	0	29451.5	1053.5	0	0	0	0	0	0	0	0	0	0	240.75	107.5		-0.47
338	NEIKNPPPANSGNTP	0	206	2330.5	0	0	0	0	0	0	0	0	0	82	0	0	0	56	0	15414	1623.5	0	0	0	0	0	0	0	0	0	0	881.5	85		-0.55
339	EIKNPPPANSGNTPN	0	0	0	903.75	0	0	0	0	0	0	0	0	0	0	0	0	0	0	7902.75	1146	15	0	0	0	0	0	0	0	0	0	258	37.5		-0.50
340	IKNPPPANSGNTPNT	0	0	0	0	0	0	0	0	0	0	0	0	0	0	0	694.75	82	0	6663.25	1791.5	0	0	0	0	0	0	0	0	0	0	371.25	0		-0.33
341	KNPPPANSGNTPNTL	0	7763.25	106	0	0	0	0	0	0	0	0	0	0	0	182	926	0	0	4212.25	988.75	0	0	493.5	0	177.5	395.5	0	0	0	0	302.5	0		0.65
342	NPPPANSGNTPNTLL	393.75	22444	0	0	0	0	0	0	0	0	0	54.5	0	0	445	715.5	0	0	0	1597.5	0	0	0	0	945.5	0	0	0	0	0	159.5	0		1.34
343	PPPANSGNTPNTLLD	1275.25	64078	191	0	0	0	2.5	0	84.5	0	10	131.5	0	0	581	1456	0	0	0	1321	43.5	2.5	0	112.5	2202.75	0	0	11.5	0	0	366.5	14		1.41
344	PPANSGNTPNTLLDK	412.75	64078	0	0	0	0	0	5.75	11003.75	0	0	82	276.75	0	97.5	1309.5	80	4265.5	0	1047.25	163	29.5	2488.75	103.5	2015.75	0	80.5	0	0	254	177.25	3193.5		1.15
345	PANSGNTPNTLLDKN	32297	64078	0	0	0	0	0	0	11421.5	0	0	34.5	255.25	0	57	1228	112.5	4067.5	0	1259	33.5	0	5182.25	83.5	1363.25	0	79.5	0	0	201	0	2961.25		2.71
346	ANSGNTPNTLLDKNK	26079	64078	0	0	0	0	0	0	6344	0	0	0	157	0	0	734.75	233.75	3848	0	987	0	0	4256.75	35	729.5	0	64	0	0	134.5	0	1974.25		2.60
347	NSGNTPNTLLDKNKK	8925.5	21567	0	0	0	0	0	88	2648	0	0	0	195.5	28.5	0	520.5	287	2960.75	0	906.5	0	0	2143	17	250.5	240.75	604.25	0	0	226	0	983		2.43
348	SGNTPNTLLDKNKKI	20427.75	40381	0	2128.5	0	0	0	39	1899	0	0	0	155	17	0	697.5	523	3526.5	0	2554	0	0	2875	0	254.5	30.25	322.75	0	0	192.5	0	1146		3.14
349	GNTPNTLLDKNKKIE	10936.75	25001.75	0	1248.5	0	0	0	0	3033	0	0	0	0	0	0	688.5	168	3184.5	0	905	0	0	1401.5	0	0	0	54	0	0	159.5	0	729.25		2.86
350	NTPNTLLDKNKKIEE	17214	16887.75	0	1219.5	0	0	0	0	3262.5	0	0	0	241.5	0	0	260.5	102.5	3456	0	855.25	0	0	527.25	0	0	0	36.5	0	0	0	0	759.75		3.51
351	TPNTLLDKNKKIEEH	9784	11260.75	57255	0	0	0	0	0	2098.5	0	0	0	0	0	0	0	0	2644.5	0	385.5	0	0	324.5	0	0	0	0	0	0	0	0	341		2.88
352	PNTLLDKNKKIEEHE	3977.25	0	64343	4081.5	0	0	0	0	281.75	0	33	0	0	0	0	0	0	812.25	0	130.5	0	0	116.5	3.5	0	0	44.5	0	0	0	0	231.5		1.85
353	NTLLDKNKKIEEHEE	4825.5	0	64343	5870.25	0	0	8.5	0	217	25	58.25	0	159.5	40	19	79	0	2175	0	0	0	0	154.5	0	41	53	115	141.5	0	0	0	250.5		2.01
354	TLLDKNKKIEEHEEK	4307.25	0	64343	1468.5	0	0	0	0	30	0	17.25	0	0	0	0	0	0	197.5	271.5	0	0	0	123.5	16	0	0	0	0	0	0	0	139		1.71
355	LLDKNKKIEEHEEKI	5874.5	0	64343	3294.25	0	8.5	8	0	0	0	0	85.5	151.5	0	0	0	0	830.5	470.5	0	9	0	207.5	0	0	0	12	0	0	0	0	0		1.92
356	LDKNKKIEEHEEKIK	3931.75	0	64343	529.5	0	0	0	0	0	0	0	0	0	0	0	0	0	205.5	315.25	0	0	0	0	0	0	0	0	0	0	6946	0	0		1.56
357	DKNKKIEEHEEKIKE	2939.5	0	64343	170	0	0	0	0	83.5	0	0	0	113.5	0	0	0	0	495	247.25	0	0	0	0	5	0	0	0	0	0	3925.75	0	0		1.51
358	KNKKIEEHEEKIKEI	5830.5	0	64343	1104.5	0	0	0	0	31.5	22	0	0	61	22	0	0	0	947.25	585	0	20	1366	0	0	0	146.5	25.5	0	0	4296.5	0	0		1.72
359	NKKIEEHEEKIKEIA	4448.25	57.5	64343	408	0	0	0	0	0	0	0	0	0	0	0	0	0	299	219.5	0	0	894.5	0	0	0	0	13	0	0	2759	0	0		1.63
360	KKIEEHEEKIKEIAK	3591.75	246.5	56221	0	0	0	0	0	0	0	0	0	91.5	0	0	0	0	403.5	0	0	0	147.25	0	0	0	494.5	0	0	0	3143.75	0	0		1.59
361	KIEEHEEKIKEIAKT	7143	1429.75	64343	0	0	62.5	88.5	0	0	52.5	0	0	0	0	0	0	0	1907.5	883.75	0	0	97	67.5	0	0	310	59	0	0	8357	0	207.5		1.75
362	IEEHEEKIKEIAKTI	1731.25	1747.25	64343	5882.5	0	0	0	0	65.25	105.5	81	0	0	0	0	0	178.75	56.75	325	43	0	157	0	0	0	0	0	0	0	8276	0	229		1.84
363	EEHEEKIKEIAKTIK	254.5	1379.25	44744.75	1185.5	0	126	0	0	0	3389.25	45	0	4714	99.5	37	0	0	0	0	277	100.25	98	40	0	0	0	0	0	0	3009.25	0	0		1.45
364	EHEEKIKEIAKTIKF	0	1929	18758.5	2022	0	0	0	0	0	1170	0	0	784	58	35.5	0	0	0	0	0	0	39	54	0	0	0	0	0	0	264	0	17.5		2.15
365	HEEKIKEIAKTIKFN	0	1280.75	0	116.75	0	0	0	0	0	256.5	0	0	0	0	0	0	0	0	0	0	0	0	0	0	0	0	0	0	0	0	0	0		1.54
366	EEKIKEIAKTIKFNI	0	674	665.25	0	0	83	0	0	0	21.5	0	0	1833	81.25	0	0	0	0	0	0	0	0	0	0	0	0	0	0	0	0	0	0		0.08
367	EKIKEIAKTIKFNID	0	876	0	0	0	0	0	0	0	0	0	0	0	60	17.5	0	0	0	0	0	0	131	17	0	0	0	0	0	0	0	0	0		1.21
368	KIKEIAKTIKFNIDS	0	1446	0	0	0	0	0	0	0	0	0	0	0	0	0	0	0	0	0	0	0	0	0	0	0	592	0	0	0	0	0	0		0.96
369	IKEIAKTIKFNIDSL	0	2146.25	0	0	0	0	0	0	0	0	0	0	0	0	0	0	0	0	53.5	139	0	34.5	0	0	0	67.5	0	0	0	0	0	0	5	1.30
370	KEIAKTIKFNIDSLF	0	308.5	0	0	0	0	0	0	0	143	0	0	164	0	0	0	0	0	0	268.5	6.5	887.75	0	2.5	0	343.5	0	0	0	0	0	56		-0.57
371	EIAKTIKFNIDSLFT	0	731.25	0	0	0	0	0	0	0	36.5	0	9.5	0	0	0	0	0	508.5	0	0	0	0	0	0	0	0	0	0	0	280.5	0	346.5		0.20
372	IAKTIKFNIDSLFTD	0	438.25	142.5	259	0	0	0	0	0	0	0	1056.25	151.5	0	0	0	2115.25	758.25	25	300	0	0	404.5	0	0	0	0	0	50.5	21	0	701.25		-0.54
373	AKTIKFNIDSLFTDP	0	259	0	0	0	0	337.5	33	53	49.5	81	926	0	0	0	0	2315.25	644.25	267.75	497	39.5	21	606.5	0	0	27	0	0	48	862.25	0	690		-0.17
374	KTIKFNIDSLFTDPL	0	2.25	0	0	0	210.5	1407	22.5	0	52.5	25.5	557	0	0	0	93.75	1719.75	505.25	0	111.25	0	0	573	0	0	313.75	0	0	0	299	0	328.5		-0.09
375	TIKFNIDSLFTDPLE	0	0	107	1478.75	14	89.5	752.75	55.5	161	88.5	97.5	814.75	152.5	40	269.75	949	1178.5	1131.25	388.25	1475.25	0	43.5	709.5	114.5	10	0	0	0	47.5	1790.75	0	588.5		-0.55
376	IKFNIDSLFTDPLEL	326	269	0	0	0	0	335.5	15	53.5	0	61	627	237	51	137.5	0	1378	804.5	78	738.25	7	33	621.5	245.5	45.5	0	0	28.5	0	749.25	205	342.5		-0.29
377	KFNIDSLFTDPLELE	1331.5	1603.5	108.25	4220.25	60	0	304.5	0	100.5	54	18	455	0	194	996	9648	1753.5	1554	2667.25	5823.75	128	50	755.75	836.5	40.5	76	406.5	172	0	3078	363.5	847.5		-0.38
378	FNIDSLFTDPLELEY	1363	3587.5	670.5	2011.25	142.5	138	901.75	53.5	941.5	174.25	261	1699.25	379	1600	3283.5	12255.5	2151	3877.25	3017.5	8524.75	237	243.5	2364	1815.5	73.5	137	1064	458.5	478.75	4319.5	682	1025		-0.52
379	NIDSLFTDPLELEYY	1550.5	9061	809.5	3170.5	148	123.5	672	83	926.5	193.5	373.25	2544.25	466.25	1126.75	1993	29378.25	4032.25	16740.25	8545	13212	633	417	1950.5	2553	93.5	249.5	725.5	421.5	429.25	4370.25	454	906.5		-0.55
380	IDSLFTDPLELEYYL	5835.25	26345.5	657.5	2256.25	99.5	51.5	453	54	2479	173.5	484.25	3250	508.25	1452.5	3948.75	33162.5	6061.25	27308.5	15057	22441	1353.5	899	2083.5	3827.25	81.5	730.5	790.25	318	991.5	10200	1282	2169.5		-0.34
381	DSLFTDPLELEYYLR	0	6628.5	366	0	32.75	0	515	28	1279.5	327.5	781.5	887.5	1519.5	1014	2009.75	23507.25	3628.5	24852.5	9567.5	14062.5	1051	473	1082.5	2205	113.25	274.5	259	134	338	4661.75	588	1140		-0.31
382	SLFTDPLELEYYLRE	2731	12780	799.5	670	1048.5	674	1351.5	857.5	4060.5	1570	2106.5	10950.75	11571.75	1550	2300	31190	4041.25	28616.5	10615	26747.75	1702	1999	18976.5	3333	802	470.25	344	875.5	3051.75	6960.25	648.5	1379.75		-0.30
383	LFTDPLELEYYLREK	1305.25	9704.75	0	0	13	0	302	6	377.5	74.5	44.5	946.75	909	516.25	541	17869	3420.5	15065.5	4517.75	9670.5	592.5	296.5	765.5	1500.5	36	0	0	83	0	5731.5	0	263		-0.56
384	FTDPLELEYYLREKN	507	6633.25	0	5413.25	0	0	139	0	116.5	11	0	0	307	209.5	322.5	6111	1241	6841.75	2885.25	9995.5	544	0	0	314.5	0	0	0	4.5	0	1233	10	0		0.10
385	TDPLELEYYLREKNK	0	0	0	1594.25	0	4407.5	18112.25	0	0	0	0	0	0	0	0	0	336.5	1123	343.25	1112	0	0	0	258.5	0	0	52	0	0	805.75	0	0		-0.08
386	DPLELEYYLREKNKK	0	0	0	0	0	0	20	0	0	0	0	0	0	0	0	0	411.75	924.75	0	350.5	0	0	0	41.75	0	0	0	0	0	1714.25	0	0		-0.25
387	PLELEYYLREKNKKV	0	0	0	1157.5	0	0	54.5	0	0	0	0	0	0	0	0	114	0	773.5	0	367.5	0	0	163	0	0	0	0	0	0	1431.5	0	435.75		-0.23
388	LELEYYLREKNKKVD	0	0	0	0	0	0	0	0	0	15	0	0	0	0	0	0	0	0	0	467.75	0	0	0	0	0	0	0	11	0	0	0	0		-0.40
389	ELEYYLREKNKKVDV	0	0	0	0	0	0	22	0	0	2530.25	0	0	197	150	0	137.5	0	509	0	264.5	0	0	112.5	0	0	0	0	0	0	0	0	0		-0.31
390	LEYYLREKNKKVDVT	0	134	0	0	0	0	0	0	0	0	0	110	0	0	0	73.75	216.5	0	11.5	0	19.5	0	0	0	0	0	3.5	0	0	0	0	612		-0.52
391	EYYLREKNKKVDVTP	0	64.5	0	141.5	98	0	0	0	0	0	0	0	23.5	43.5	0	0	93.5	0	0	1060	0	0	0	0	0	0	0	0	0	1368	0	0		-0.45
392	YYLREKNKKVDVTPK	0	0	0	0	0	0	0	14	0	0	0	0	0	0	0	0	0	0	0	308	0	0	0	0	0	0	0	0	0	0	0	387.5		-0.33
393	YLREKNKKVDVTPKS	0	417.5	45.5	0	0	0	0	70	0	0	0	0	0	0	0	0	261.75	0	0	244.5	0	0	0	156	0	0	0	0	0	0	0	0		0.38

394 LREKNKKVDVTPKSQ	0	0	0	2053.25	0	0	0	0	0	0	0	0	0	0	0	0	0	0	0	209.5	0	0	0	9.5	0	0	0	0	0	0	0	0		1.31
395 REKNKKVDVTPKSQD	0	125	176	237	0	0	0	0	0	0	0	0	103.5	0	0	362.5	157.5	0	0	0	0	0	0	0	0	0	0	0	0	41	0	0		1.02
396 EKNKKVDVTPKSQDP	340	164.5	202	1237	0	0	0	0	0	9	0	18	38.5	93.5	0	306.75	281	0	0	0	0	0	0	0	0	0	0	0	0	77	0	0		3.24
397 KNKKVDVTPKSQDPT	64759	95.5	118.5	0	0	0	0	0	0	0	0	0	0	0	0	0	169	0	0	0	0	0	0	0	0	571	0	0	0	84.25	0	0		1.38
398 NKKVDVTPKSQDPTK	64759	0	46.5	0	0	0	0	0	0	0	0	10025.5	0	2439.5	532.5	6260.75	1109	2102.5	3627.25	19402.75	0	0	21	2882.25	0	0	0	0	0	812.5	1442	793.5		0.88
399 KKVDVTPKSQDPTKS	64759	0	0	5595	0	0	0	0	0	0	0	8126	0	1443.25	1123	9367	3244.75	3695.25	0	10673.25	0	0	0	3077.5	241.75	1394	0	0	0	744.25	2950	1711.75		1.25
400 KVDVTPKSQDPTKSV	59347	58	0	0	0	0	0	0	0	33	0	8172.25	0	1137	878.25	5369	2092.25	1456.5	2081.25	9553.5	0	0	13.5	1148.25	199	301.5	12.5	0	0	373	0	1228.5	6	1.05
401 VDVTPKSQDPTKSVQ	36765.75	0	0	0	0	0	0	0	0	0	0	6419	0	559.75	0	3809.5	1724.25	1331.75	1791.5	8831.5	0	0	0	1230	0	0	66	0	74.5	365.5	1131.25	583		0.92
402 DVTPKSQDPTKSVQI	55483	0	0	0	0	0	0	0	0	0	0	8911.5	0	866.5	0	4360.25	1522.25	1524.5	2459	13889	0	12.5	0	1152.5	194	0	0	0	0	687.5	705	1381		0.95
403 VTPKSQDPTKSVQIP	40165.5	0	0	0	0	0	0	0	0	0	0	8113.5	0	1004	550.5	4361.5	1969.5	1902.5	1499.5	0	0	0	0	972.75	156	0	0	0	0	409.75	0	645		1.05
404 TPKSQDPTKSVQIPK	26600	0	0	0	0	0	0	0	0	0	0	6518.25	0	857.5	52.5	5025	1856.75	1770.75	1822.25	0	222	0	0	913.5	558	0	35	0	0	738.5	934.25	770.5		0.88
405 PKSQDPTKSVQIPKV	30100	0	0	0	0	0	0	0	0	0	0	5745	0	684.75	180	4608	1752	1446	2454	0	0	51	91.5	1051.5	693	0	0	0	0	1024.5	1488.25	1145.5		0.96
406 KSQDPTKSVQIPKVP	16325	0	0	0	0	0	0	0	0	0	0	654.5	0	0	0	3587.25	1629.75	1488.75	1805.25	0	0	0	0	0	1096.5	1545.5	0	0	0	632	1246.5	812		0.88
407 SQDPTKSVQIPKVPY	7176.5	0	84.75	0	0	0	0	0	0	0	0	0	0	0	28.25	3266.25	1113	826.25	1802.75	0	88.5	0	0	86	0	0	0	0	0	232.25	401.75	0		0.64
408 QDPTKSVQIPKVPYP	0	1308.5	175	0	0	0	0	0	0	0	0	0	0	101.5	619.5	3398.5	1307.25	1003	1535	0	155.5	0	47.5	216	0	0	35	0	5	669.5	419.5	313.5		-0.54
409 DPTKSVQIPKVPYPN	0	24616	0	0	0	0	0	0	0	0	0	0	0	0	688.5	1967	992	620.5	607.5	0	0	0	0	417.75	0	0	22.5	0	0	965.5	1201.5	380.5		1.21
410 PTKSVQIPKVPYPNG	0	5837.5	136.5	0	0	0	0	0	0	0	0	0	0	0	0	0	0	37.5	0	0	53.5	0	0	0	0	0	0	0	0	0	0	0		1.45
411 TKSVQIPKVPYPNGI	1528	5080	0	0	0	0	0	0	0	0	0	0	0	0	5.25	0	0	75.5	279	0	0	0	343.5	0	0	0	0	0	0	46	0	0		2.38
412 KSVQIPKVPYPNGIV	830.5	6052.5	0	0	0	0	0	0	0	0	0	0	0	0	0	0	0	55.5	193	0	11.5	0	156.5	0	0	329	0	0	0	46	0	0		1.81
413 SVQIPKVPYPNGIVY	0	0	0	0	0	0	0	0	0	0	0	0	0	0	0	0	0	0	0	0	54.5	0	0	0	0	0	0	0	0	0	0	0		-0.41
414 VQIPKVPYPNGIVYP	122	3374.25	213.5	0	0	0	0	0	0	0	0	0	0	0	0	0	73.5	199.5	0	0	58.5	0	45	0	0	0	0	0	0	0	0	0		1.69
415 QIPKVPYPNGIVYPL	0	1433.75	362	295	0	0	0	0	0	0	0	0	0	0	0	0	0	0	0	0	0	0	19.5	0	0	0	0	0	0	0	0	0		3.37
416 IPKVPYPNGIVYPLP	0	1939.75	0	0	0	0	0	0	0	0	0	0	249	149.5	127	451	2200	393	0	804.75	49.5	0	35	0	0	0	0	0	28	0	0	0		-0.09
417 PKVPYPNGIVYPLPL	47	2009	0	19.5	0	0	33.5	0	0	0	0	0	333	440.5	103.5	763	2887.5	603.5	0	3677.75	39	0	47.5	0	0	0	0	0	32	0	0	342.5		-0.47
418 KVPYPNGIVYPLPLT	0	2184.5	0	0	0	0	0	0	0	0	0	0	139.5	501.75	446.5	901	2709.5	460.5	0	2908	30.5	195	0	86	0	686.5	0	0	0	0	596	446.75		-0.47
419 VPYPNGIVYPLPLTD	0	2981	0	581	0	0	51	0	0	459	0	1006	344	594	697.5	846.75	3292.25	583	17	2843.25	38.5	342.5	82.75	305.5	33.5	0	0	0	0	0	2224.5	1305.75		-0.37
420 PYPNGIVYPLPLTDI	221	3254.5	0	0	0	0	67	0	0	408	0	856	302.5	579	168.5	1528	3679	749.25	58	5918.5	137.5	325	37	438.5	49.5	0	0	10	0	0	1991.5	2334.5		-0.55
421 YPNGIVYPLPLTDIH	0	2036.5	0	0	0	0	0	0	0	122.5	40	343.25	123.5	276	0	1870.5	3131	356.5	73	8057	140.5	75.5	0	194.5	0	0	0	0	134.5	501	743.5	799.5		-0.49
422 PNGIVYPLPLTDIHN	0	1864	0	0	0	0	0	0	0	247	8.5	142.5	98	272.25	49	1823.25	1830.25	929	120	6804	50.5	28	0	199	0	0	0	0	236.5	47	530.5	1008.25		-0.51
423 NGIVYPLPLTDIHNS	522	2180.5	0	0	0	0	0	0	0	84	0	162	42.5	340.5	0	787.75	1069.75	421.5	64.5	3012.25	6.5	0	0	195	0	0	0	0	101	0	177.5	407.5		-0.07
424 GIVYPLPLTDIHNSL	776.75	1310	0	961	0	0	0	0	0	60	0	0	0	203	0	909	1114	232.5	252.5	3080	0	0	0	24.5	0	0	0	0	86.5	0	198.5	331.25		0.19
425 IVYPLPLTDIHNSLA	365.25	1007.25	0	0	0	0	0	0	0	0	0	106.5	0	137.5	7.25	392.25	459	0	73.5	1596.75	0	0	0	0	0	0	0	0	76	11.25	263.5	130.5		-0.03
426 VYPLPLTDIHNSLAA	0	821.5	0	0	0	0	0	0	0	0	0	0	0	0	0	435.75	31.25	0	339.5	361	0	0	0	0	0	0	0	0	0	0	0	0		0.32
427 YPLPLTDIHNSLAAD	585.5	548.5	0	0	0	0	0	673	0	71.5	0	84.75	0	0	0	0	2783.75	0	70.5	0	0	25	0	0	0	0	0	0	8	0	152.5	60.5		-0.36
428 PLPLTDIHNSLAADN	0	0	0	269.5	0	0	56.5	0	0	66	40.5	15	0	0	0	0	518.75	0	92.5	164	0	0	0	0	0	7.5	0	0	22.5	0	77.5	117.5		-0.44
429 LPLTDIHNSLAADND	0	0	0	443	8	0	0	46	0	0	54	0	0	0	0	0	3379.75	0	489.5	0	0	0	0	0	0	0	16	94.5	0	0	0	0		-0.51
430 PLTDIHNSLAADNDK	0	0	0	0	0	0	38.5	12	0	0	19.5	0	0	0	0	0	1462.25	0	0	0	0	344.5	6.5	1900.75	0	0	0	54.75	0	425.75	0	0		-0.25
431 LTDIHNSLAADNDKN	0	0	0	0	0	0	0	0	0	0	0	0	0	0	0	0	1220.5	0	0	0	0	164	0	1796.5	0	0	0	0	0	117	0	196		-0.28
432 TDIHNSLAADNDKNS	0	185	0	0	0	0	0	0	0	0	0	0	0	0	0	0	894	0	0	0	0	0	0	862	0	0	0	0	0	0	0	75.5		-0.48
433 DIHNSLAADNDKNSY	0	0	0	0	0	0	0	0	0	0	47.5	0	0	0	0	0	661	0	0	0	0	116	0	612	0	0	0	0	0	0	0	123.5		-0.26
434 IHNSLAADNDKNSYG	0	204.5	0	363	0	0	0	0	0	0	0	0	0	0	0	0	0	0	54	0	0	0	0	194.5	0	0	0	0	0	0	0	0		2.12
435 HNSLAADNDKNSYGD	0	454.75	73.25	2114.25	142	0	0	1065.25	0	0	780.25	0	17	0	0	0	305	25	401.5	0	0	101.5	0	197	0	0	0	0	174.5	0	0	0		1.48
436 NSLAADNDKNSYGDL	0	187.5	0	716.5	0	0	0	0	0	0	0	15	0	43.5	0	0	261	162	11	0	0	0	0	59	0	0	0	0	0	0	0	0		1.57
437 SLAADNDKNSYGDLM	0	0	0	0	0	0	0	0	0	0	17	0	0	148.5	0	1210.5	143.5	251	0	0	0	45	0	98.5	0	0	0	0	0	294.75	369.5	406.25		-0.13
438 LAADNDKNSYGDLMN	1712.25	0	0	281.5	0	0	0	0	0	0	0	8.5	0	667	0	4792.25	106.5	384	0	1816.5	63.5	254.5	0	338	0	0	0	0	0	365.5	563.5	630.25		-0.52
439 AADNDKNSYGDLMNP	4439.5	0	0	603.5	0	0	0	0	0	0	0	0	0	723.75	0	2167.5	894.25	243.5	0	1043.75	0	181.5	0	379	0	0	0	0	12	383	913	683.5		0.67
440 ADNDKNSYGDLMNPH	6424.75	0	0	0	0	0	0	0	0	0	0	0	0	0	0	3079.75	0	155.5	0	1313.25	0	0	0	732.5	0	0	0	0	0	0	125.5	244		0.69
441 DNDKNSYGDLMNPHT	4548.5	0	0	0	0	0	0	0	0	0	0	0	0	373.5	217.5	2238.5	0	0	111.5	1362	0	241	0	0	0	0	0	0	0	359	331.75	165		0.55
442 NDKNSYGDLMNPHTK	1787	0	0	0	0	0	0	0	0	0	0	0	0	0	0	4726.5	570	5113	80.5	316	0	0	0	0	0	0	0	0	0	244.5	791.5	0		-0.55
443 DKNSYGDLMNPHTKE	3573.5	0	0	0	0	0	0	0	0	0	51	0	0	284.25	0	3202.5	344	4358	0	876.5	0	0	0	69	0	0	0	0	0	223.5	1255	61		-0.26
444 KNSYGDLMNPHTKEK	2001.5	20287.75	0	0	0	0	0	0	0	138	0	0	0	154.5	220.75	3056.25	0	3429.5	0	1422	0	353.75	0	0	0	468	0	0	0	1498	1158	0		1.36
445 NSYGDLMNPHTKEKI	4420	30037	0	0	0	0	0	0	0	0	0	0	0	0	0	0	0	3533	0	0	0	81	0	0	13.5	0	0	0	0	1128.5	715.5	0		1.80
446 SYGDLMNPHTKEKIN	1590.5	27268.25	0	0	0	0	0	0	0	0	0	0	3	0	0	1232	346.25	2417	0	327.5	0	134	0	88	0	0	0	0	0	444.75	373.25	0		1.48
447 YGDLMNPHTKEKINE	1512	18853.25	0	0	0	0	0	0	0	0	0	0	0	0	0	0	305.5	1648.5	397	6043.25	0	141	0	728.5	0	0	0	0	0	118.75	398.25	0		1.24
448 GDLMNPHTKEKINEK	1325	18303	0	1133.5	0	0	0	0	0	181	0	99	128.75	0	0	3676.5	228	2236.75	246.25	3977.25	0	208	0	2370	0	0	0	0	0	226	438.75	0		1.33
449 DLMNPHTKEKINEKI	207.5	25029	0	2967.75	0	0	0	0	0	0	7	0	9337.75	200	0	13700.75	2794.25	966	1754.5	18788.25	430.5	463.5	1212.5	4000.75	0	18.5	278.5	0	2571.5	761.5	945.25	2965		0.17
450 LMNPHTKEKINEKII	209.5	31814.25	41.75	4386.5	0	0	0	0	1239.5	191	0	195.5	13580.5	520.25	0	15667	10089.25	896	3293.5	16085	626	1137.25	1704.25	8746.5	328.25	231.5	413.25	388.5	3201.5	5673	2739	5843.75		0.17
451 MNPHTKEKINEKIIT	429.25	36545	115.5	7999.75	0	0	0	0	0	273.5	165.25	277.5	14889.75	343	0	16646.5	10014.5	1824.5	3778	20913.5	1058.75	1041	1014	5416	215	0	660.5	502	3427.5	6545.5	1244.5	10086		0.35
452 NPHTKEKINEKIITD	649.5	30285.25	251	7174.75	0	0	0	0	1968	157	188	294	12186.5	406.5	0	16312.5	10323	1259.5	3690	22918.75	1018.5	1618.25	1701.75	6018.5	202	1706.75	679	206	4195.5	8016.75	1634	8722.25		0.12
453 PHTKEKINEKIITDN	14797	9534.75	0	2789.5	0	0	0	21	616.25	14.5	86.5	35	6184	0	0	11198	6004.5	1410	1721.75	19025.5	500	617.25	769	1169	0	0	245.5	111.75	2686	2719	404.75	4525.5		0.39
454 HTKEKINEKIITDN K	43109.5	2360.25	0	1101	0	0	75.5	0	229.5	0	0	0	4403.5	113	0	8166.5	3262.25	854.5	1395.5	21044.25	261.75	0	171	1236.25	0	849	40.25	0	1370.25	1066.5	273	1165.75		0.84
455 TKEKINEKIITDN KE	23653.5	0	0	0	0	0	0	0	256	0	0	0	4686.25	116.5	23	7681.5	1353.5	778.25	947.5	15566.75	39.5	276.25	248	2203	0	506.5	0	0	1807.5	895	176.5	2196.5		0.28
456 KEKINEKIITDN KER	9667.25	0	129	588.5	0	0	0	0	125	0	0	0	1895.25	122.5	27	6562.5	2858	135	582.75	12694	27.5	1490.75	145.5	1980	34.5	869.5	61.5	0	677.5	2613.75	46.5	1333.75		-0.26
457 EKINEKIITDN KERK	11319	0	0	0	0	0	13	0	0	12.5	0	0	60	139.75	0	0	0	93	532.5	7411	0	31.5	0	0	0	442.5	0	0	0	240.75	0	0		0.60
458 KINEKIITDN KERKI	7023.75	0	0	0	0	0	0	0	0	0	0	0	0	0	0	29.5	0	0	273.5	4406.5	0	0	0	0	0	1575	0	0	0	145.5	0	0		0.55
459 INEKIITDNKERKIF	7748	0	0	1351.5	0	0	0	0	0	0	0	0	0	6.5	0	0	0	0	352	5346.25	0	15	0	0	0	242.5	0	13.75	0	80.5	31.5	0		0.96
460 NEKIITDNKERKIFI	16103.25	676.5	0	0	0	0	0	0	0	0	0	0	0	0	0	51	0	0	413	7694.75	11	10	0	0	0	192.5	0	0	0	72.5	46	0		0.97
461 EKIITDNKERKIFIN	8890	451.25	0	0	0	0	0	0	0	23	0	0	35.5	100.5	0	0	0	0	0	1701.25	222	0	0	0	0	0	0	0	0	33	0	0		1.38
462 KIITDNKERKIFINN	5113.5	0	0	0	0	0	0	0	0	0	0	0	0	0	0	0	0	0	0	0	117.5	11.5	0	0	0	692.25	26.5	0	0	0	0	0		1.27
463 IITDNKERKIFINNI	2065	0	181.5	95	0	0	0	0	0	0	0	0	0	0	0	0	0	0	0	0	33.5	0	0	0	0	0	20.5	0	92.5	0	0	0		1.86
464 ITDNKERKIFINNIK	2259.75	1678	0	0	0	0	21	0	183.5	278.5	0	412.5	0	265.5	0	2236.75	6195.5	700.25	281.5	922	0	65.25	0	0	0	734.25	362	0	0	1522.25	385.25	2014		-0.41
465 TDNKERKIFINNIK K	0	12175	0	0	9	0	0	109.5	165.5	968.5	0	883.25	167	1793.75	0	4578.5	12335.5	4897.5	1526.5	2815	0	349.5	0	1491	215.5	1440.25	1111.5	0	0	14356.75	584	4665		-0.43
466 DNKERKIFINNIK KK	0	11453.25	184.5	0	67	0	105.5	649	294	935.75	0	301.75	289.5	2005	0	10406	7622	8360.75	0	2027.5	9	577.5	95.5	1472	145.5	1061	1151.5	149	0	7279	0	3318.25		-0.37
467 NKERKIFINNIK KKI	0	18372.75	1093	3041	163	38	233	825.5	585.25	1833.5	0	677.5	213	0	110	14606.25	9842.75	13065	1434.75	3118.25	57	1301	141.5	1530.5	171	2718.25	1651	550.5	0	8905	2452.5	4226.25		-0.07
468 KERKIFINNIK KKID	0	7488.25	325	1632.75	0	0	0	0	41.5	0	0	0	84	769	0	10424.5	4688.25	8646	980	3920.25	0	296	0	502.5	0	499.25	0	0	0	5003	276.75	1327		-0.37
469 ERKIFINNIK KKIDL	0	3279.5	0	0	0	0	0	0	13	54.5	0	138.25	19	195.5	0	8461	3033.25	3381.5	272	2182	0	129.5	0	318	0	122.25	0	0	0	3492.5	0	312.5		-0.54
470 RKIFI NNIKKKIDLE	0	6554.75	0	8701.25	0	0	13.5	0	57.5	4.75	0	0	0	0	0	4173.25	777.25	1241.5	0	0	0	0	0	0	0	0	0	0	0	211.5	0	221.5		2.50
471 KIFI NNIKKKIDLE E	0	7819.5	24	455.25	0	4.75	0	0	87	21.5	0	0	353	947.25	0	2787	641.5	1743	113	699	0	31.5	0	0	0	226	0	115.5	14.5	0	0	0		0.90
472 IFI NNIKKKIDLE EK	0	7450	0	0	0	0	0	1557.25	0	0	0	0	201	788.5	0	3595.5	3509.5	1883.25	91.5	1865.5	0	20.5	0	0	233.75	0	910.5	9	0	11888.5	0	37.5		-0.34
473 FI NNIKKKIDLE EKN	0	6810	0	6005	0	0	0	0	0	9.5	9	0	470.25	492.5	0	2202.75	1861.5	1604	126.5	1018	0	0	88.5	0	0	0	344.75	0	0	5871	0	0		1.41
474 INNIKKKIDLE EKNI	0	7488.75	0	7820.5	0	0	0	580.5	1147.5	600	33	79.5	3869.75	2695.5	0	1768.5	3316.5	3075.25	1688.25	2879	276.5	0	308.5	0	121	882.25	514.75	0	0	6275.75	1967	1368.25		0.80
475 NNIKKKIDLE EKNIN	0	4162.25	58.5	1821.75	0	0	0	69.5	245.75	68.5	12	99.25	1621	836.5	0	259	670	754	1110	1338.75	0	0	43	0	0	232.5	97	0	0	2853.25	418.75	323.5		0.77
476 NIKKKIDLEEKNINH	95.5	2923.25	113.5	1013.25	0	0	0	11	0	53.5	0	27	997.25	655	0	203	109.5	0	133.5	740	8	0	0	0	0	0	0	0	0	1248	0	129		1.49
477 IKKKIDLEEKNINHT	64.5	3143.25	0	505.5	0	0	0	0	0	40.5	0	0	1760.75	695	0	179	301	420.75	243.5	439	0	0	0	0	0	0	86	0	0	405.25	0	217.25		0.83
478 KKKIDLEEKNINHTK	0	2579.5	0	0	0	0	0	31.5	0	22	0	0	1111.25	0	0	0	488	261	1790	700	0	0	0	0	0	1579.5	0	0	0	797.5	823.5	0		-0.14
479 KKIDLEEKNINHTKE	134	823	0	0	0	0	0	25.5	50	83.5	15	0	0	467.75	0	107.5	357.5	440.5	2799.75	667.5	0	30.5	0	8	0	0	0	35	0	937.5	0	137.25		-0.55
480 KIDLEEKNINHTKEQ	517	18576.25	0	0	0	0	0	29.5	55	84.5	15.5	0	791	375	0	67.5	0	126	1000.25	1611	0	213.5	0	0	0	0	0	19.5	0	0	0	54		1.35
481 IDLEEKNINHTKEQN	203.25	10421.5	0	5161.25	0	0	0	0	9.5	0	73.5	11	150.5	0	0	0	0	2.5	700.25	635.5	0	65	0	0	0	0	0	17.5	0	0	0	0		3.05
482 DLEEKNINHTKEQNK	184.5	15135	0	3392.5	0	0	0	0	0	0	0	0	0	0	0	0	0	0	689	290	0	0	0	0	0	0	0	11.25	0	0	23.5	0		2.23
483 LEEKNINHTKEQNKK	164.5	13521.5	0	0	0	0	0	18	0	0	0	0	0	0	0	0	0	0	421.5	1084.75	0	0	0	0	0	0	0	0	0	0	106	0		1.35
484 EEKNINHTKEQNKKL	195.25	13952	0	3399	0	22	0	5	0	77	0	0	1810	125.5	0	0	0	0	644.25	0	0	0	0	0	0	0	0	0	0	0	0	0		2.20
485 EKNINHTKEQNKKLL	164	9601.25	0	1788	0	0	0	0	0	0	0	0	0	71.5	0	162	0	0	608	331	0	0	0	0	0	794.5	0	0	0	0	343.75	0		2.00
486 KNINHTKEQNKKLLE	0	4350.5	0	0	0	0	0	0	0	0	0	0	0	0	0	0	0	0	434.5	0	0	0	0	0	0	623.75	0	0	0	0	278	0		1.18
487 NINHTKEQNKKLLED	366	3999.5	0	219	0	0	0	0	0	0	0	0	0	0	0	0	0	0	850.75	0	0	0	0	36	0	395	0	0	0	0	0	0		1.67
488 INHTK EQNKKLLEDY	399.5	2228.5	54	1018.75	0	0	0	0	62	0	0	0	0	0	0	0	0	0	0	0	0	0	0	36.5	11.5	0	0	0	0	0	0	0		4.18
489 NHTKEQNKKLLEDYE	103	1637.5	363	3603.25	20	0	27	9	179.5	18	41.5	0	71	47	1.5	27.5	0	0	27.5	0	0	113.5	19	96.5	0	3006.5	12.5	80.5	59	110	22	0		1.62
490 HTKEQNKKLLEDYEK	0	279	0	0	0	0	54	0	0	71.5	0	0	0	0	542.25	0	0	0	0	0	0	0	0	79.5	0	0	0	0	0	0	0	0		-0.29
491 TKEQNKKLLEDYEKS	60.75	0	106.75	2910	0	0	0	0	0	70.5	0	0	0	0	0	0	0	0	0	0	0	30.5	0	0	0	0	0	0	0	0	0	0		1.57
492 KEQNKKLLEDYEKSK	0	0	81.75	0	0	0	0	0	0	109.5	0	0	405.5	0	0	0	0	0	0	0	0	114	0	24.5	0	175	0	0	0	0	0	0		-0.46
493 EQNKKLLEDYEKSKK	0	0	0	1619	0	0	0	0	0	199	0	0	98.75	59.5	0	0	0	0	0	179.5	0	0	0	0	0	0	0	0	0	0	0	0		1.18
494 QNKKLLEDYEKSKKD	0	0	0	0	0	0	0	0	0	100	0	0	0	0	0	0	0	0	0	0	0	0	0	0	0	0	0	0	0	0	0	0		-0.41
495 NKKLLEDYEKSKKDY	0	0	0	0	5	0	0	0	0	0	0	28.5	0	0	0	0	0	0	0	0	0	0	0	0	0	0	0	0	0	0	0	0		-0.41
496 KKLLEDYEKSKKDYE	0	0	0	0	40.5	0	0	0	110.25	77	17	0	0	0	0	81.5	0	109	0	0	0	176	0	0	0	160	18.5	50	33	0	0	46		0.14
497 KLLEDYEKSKKDYEE	148.5	0	249.5	0	93.5	46	0	0	95	147	69.5	62	286.5	154.5	27	106.5	52.5	138	42	0	0	847	118.25	0	24	9.5	70.5	292	293.5	74	0	232		-0.44
498 LLEDYEKSKKDYEEL	350	324.5	196	192	71.5	559	48	0	58	49.5	56	102	0	147.5	0	83	0	96	0	178.5	66.5	1311	79	0	0	0	24.5	88.5	35.5	52	0	575.75		-0.09
499 LEDYEKSKKDYEELL	94	664	32.5	976.75	54.5	29	23	0	54.5	15	59	0	60.25	106.5	0	28	0	10.5	44.5	2661.25	305	146	15	0	0	0	0	0	27.25	9	0	81		-0.08
500 EDYEKSKKDYEELLE	166.5	614.5	176.5	1053	226	0	28.5	8.75	162.25	137.5	75.5	186.75	303.5	329	40	92	0	0	102	1439.75	168.75	464.5	76	0	25	134	65.5	140.5	64	103.5	17.25	155.5		0.63
501 DYEKSKKDYEELLEK	19.5	689.25	0	0	34.5	28.5	3.5	0	0	0	0	199.25	0	0	0	0	0	0	0	1003	0	250.5	0	0	0	0	0	0	0	0	0	0		-0.12
502 YEKSKKDYEELLEKF	0	195	0	0	37.5	41.5	22	0	0	0	27.5	132	0	0	0	0	0	0	0	644.5	0	272	0	0	0	0	0	0	0	0	189.5	85		-0.54
503 EKSKKDYEELLEKFY	0	7283.5	169	713.25	31	51	0	0	0	91.5	56.5	8	279.5	193.25	40	0	2256.75	0	0	0	25.5	81.5	0	0	0	0	0	0	107.75	0	126.5	55		1.43
504 KSKKDYEELLEKFYE	712.5	24924	292	1072.75	161.5	89.5	49	0	155.5	43.5	146.5	33.5	98.5	179	84	316	1244.5	0	48	101	48.5	155.5	41.25	54.5	9	65	48	81.5	120.5	9.5	169	82		1.62
505 SKKDYEELLEKFYEM	544.5	27012	0	1975.5	85.5	58	45.5	0	33.5	0	68	83	89	124.5	64.5	124.5	902	127	0	0	6.5	102.5	26	188	13.75	0	38.5	0	70.5	18	0	76.75		1.69
506 KKDYEELLEKFYEMK	0	18888.5	0	0	28	0	11.5	0	0	0	0	29.5	95.5	0	0	0	437	0	0	0	0	87	0	0	0	224.5	0	0	0	0	0	0		1.35
507 KDYEELLEKFYEMKF	0	30788.5	0	914.5	32	19	30.5	0	59	0	26	0	153	0	0	0	288.5	0	0	0	0	3774.25	14	0	0	0	0	0	0	6	0	78.5		1.39
508 DYEELLEKFYEMKFN	5660	64943	0	1633	52	18	34.5	4.5	46	20.5	33.5	0	207	129.5	0	0	398.5	77	25.5	130.5	0	0	0	0	0	0	0	154.5	13	41.5	49	80		1.80
509 YEELLEKFYEMKFNN	1154	18109.75	0	551	33.5	22.5	26.5	0	402	0	91.5	0	989	53	0	0	0	0	0	0	0	0	6	0	0	0	48.75	36.75	0	0	0	30		1.68
510 EELLEKFYEMKFNNN	0	7080.5	0	0	80	90	19.5	0	76.5	54.75	0	6	3700	300.5	0	0	0	0	0	0	0	0	93	23	0	0	127	0	0	0	0	0		0.78
511 ELLEKFYEMKFNNNF	0	3067.25	0	850.5	148.5	0	46	0	0	155	0	0	468	364	80	0	0	0	0	0	0	0	256.25	0	0	0	33.5	0	5	0	0	0		2.06
512 LLEKFYEMKFNNNFN	0	0	0	560.5	0	31	0	0	0	0	0	0	0	0	0	0	0	0	0	0	0	0	0	0	0	0	49	0	0	0	0	0		1.32
513 LEKFYEMKFNNNFNK	0	0	0	0	0	0	0	0	0	0	0	0	0	0	0	47	0	0	0	0	0	0	0	0	0	594.5	11	0	0	9	0	0		-0.39
514 EKFYEMKFNNNFNKD	0	0	151.75	0	0	0	7.5	0	35.25	41	0	0	236.5	51	0	0	38.75	0	0	0	0	0	0	0	0	311.5	59.5	0	0	34	0	0		-0.54
515 KFYEMKFNNNFNKDV	0	0	0	181.25	0	0	13.25	0	0	0	0	0	0	0	0	0	0	0	0	0	0	0	0	0	0	313.75	44	0	0	0	0	157		-0.31
516 FYEMKFNNNFNKDVV	0	0	0	0	0	3	0	0	626.25	0	0	0	0	0	0	0	0	0	0	0	0	0	0	0	0	168	38	25.75	0	0	0	0		-0.35
517 YEMKFNNNFNKDVVD	0	0	332.25	523.25	0	3	0	0	0	0	0	7.75	0	0	0	0	0	0	0	74.5	0	0	0	0	0	0	21.25	8	0	0	0	0		3.15
518 EMKFNNNFNKDVVDK	0	0	146.5	0	0	0	0	0	0	89	0	0	531.5	0	0	0	0	0	0	0	0	0	0	0	0	0	0	0	0	0	0	0		-0.50
519 MKFNNNFNKDVVDKI	0	0	0	0	0	0	0	220	0	0	0	0	0	0	0	0	0	0	0	0	0	0	0	0	0	0	0	0	0	0	0	424.5		-0.41
520 KFNNNFNKDVVDKIF	0	0	0	0	0	0	0	0	0	0	0	0	0	0	0	0	36.75	0	0	0	0	0	0	0	0	287.75	44	0	0	0	0	0		-0.36
521 FNNNFNKDVVDKIFS	0	0	0	0	0	0	34.5	0	0	0	0	0	0	0	0	0	0	0	0	0	0	0	0	0	0	0	0	0	0	0	0	0		
522 NNNFNKDVVDKIFSA	0	0	0	565.25	0	0	0	0	0	0	0	99	0	0	0	0	213	0	0	0	0	0	0	0	0	0	40	0	0	0	0	0		0.88
523 NNFNKDVVDKIFSAR	0	0	0	0	0	0	14.5	0	0	0	0	42.5	0	0	0	0	208	0	0	0	7.5	0	0	0	0	0	15.5	0	0	131.5	0	0		-0.28
524 NFNKDVVDKIFSARY	0	858	0	0	0	0	0	0	0	0	0	0	0	0	0	0	169.5	0	0	0	0	0	0	0	0	0	0	0	0	0	0	0		1.22
525 FNKDVVDKIFSARYT	71.5	1036.25	0	0	0	0	0	0	0	0	0	0	0	0	0	45.5	245	0	0	0	0	0	0	0	0	0	0	0	0	0	0	0		1.39
526 NKDVVDKIFSARYTY	0	409.5	0	0	0	0	0	0	0	0	0	0	0	0	0	15	166.5	0	0	0	0	0	0	0	0	0	0	0	0	0	0	0		0.95

527 KDVVDKIFSARYTYN	0	429.5	0	0	0	0	0	0	0	0	0	0	0	0	0	0	0	0	0	0	16.25	0	0	0	0	478	0	0	0	0	0	0		0.17
528 DVVDKIFSARYTYNV	0	772	0	0	0	0	0	0	0	0	0	0	0	0	0	0	0	0	0	0	0	0	0	0	0	0	0	0	0	0	0	0		1.37
529 VVDKIFSARYTYNVE	2819	155	57.5	214	0	0	18	0	0	0	7.5	0	2	15	0	0	0	0	0	0	0	0	0	0	0	0	0	33.5	48	0	0	135.25		1.93
530 VDKIFSARYTYNVEK	679.25	0	0	0	0	0	0	0	0	0	0	0	0	0	420.25	0	0	0	0	0	0	0	0	0	0	0	0	0	0	0	0	0		0.68
531 DKIFSARYTYNVEKQ	171	0	0	0	0	0	0	0	0	0	0	0	0	0	0	0	0	0	0	0	0	0	0	0	0	0	0	0	0	0	0	100.75		0.72
532 KIFSARYTYNVEKQR	0	0	1352	0	0	0	0	0	0	0	0	0	0	0	0	0	0	0	0	0	0	0	0	0	0	587.25	0	0	0	0	0	0		0.93
533 IFSARYTYNVEKQRY	0	0	181	0	0	0	0	0	0	0	0	0	0	0	0	0	0	0	0	0	0	0	0	0	0	0	0	0	0	0	0	0		1.37
534 FSARYTYNVEKQRYN	0	0	0	381.5	0	0	0	0	0	0	0	0	0	0	0	0	0	0	0	0	12.25	0	0	0	0	0	0	0	0	0	0	0		1.36
535 SARYTYNVEKQRYNN	0	0	0	768.25	0	0	0	0	0	0	0	0	0	0	0	0	0	0	0	0	0	0	0	0	0	0	27	0	0	0	0	0		1.35
536 ARYTYNVEKQRYNNK	0	0	0	0	0	0	0	0	0	0	0	0	0	0	0	0	0	0	0	0	0	0	0	0	0	0	0	0	0	32.5	0	0		-0.41
537 RYTYNVEKQRYNNKF	0	0	0	0	0	0	0	0	0	0	0	0	0	0	0	0	0	0	0	0	0	0	0	0	0	0	0	0	0	0	0	25.5		-0.41
538 YTYNVEKQRYNNKFS	0	0	0	0	0	0	0	0	0	0	0	0	0	0	0	0	0	0	0	0	0	0	0	0	0	0	0	0	0	786.5	0	0		-0.41
539 TYNVEKQRYNNKFSS	0	0	0	0	0	0	0	0	0	0	0	0	0	0	0	0	0	0	0	0	0	0	0	0	0	0	0	0	0	728.5	79.5	0		-0.39
540 YNVEKQRYNNKFSSS	0	0	0	0	0	0	0	0	0	0	0	0	0	0	0	0	0	0	0	0	0	0	0	0	0	0	0	0	0	1861.25	0	0		-0.41
541 NVEKQRYNNKFSSSN	17.5	0	253.5	548.25	0	0	0	0	0	0	0	0	0	0	0	0	0	0	288.5	0	0	0	0	0	0	0	9.5	0	0	2133	0	0		-0.39
542 VEKQRYNNKFSSSNN	0	0	670	0	0	0	0	0	0	17.5	0	0	0	0	0	0	0	0	28.5	0	0	0	0	0	0	0	37	0	0	1509.5	0	0		-0.31
543 EKQRYNNKFSSSNNS	0	0	149.5	1201	0	0	0	0	0	29	0	0	0	0	0	0	0	0	298	0	0	0	0	0	0	0	0	0	0	860.75	0	0		0.71
544 KQRYNNKFSSSNNS V	0	0	239.5	3127.25	0	0	0	0	0	0	0	0	0	0	0	0	0	0	143	0	0	0	0	0	0	0	17.5	0	0	343.5	0	0		1.55
545 QRYNNKFSSSNNS VY	0	0	0	954.5	0	0	0	0	0	21.5	7.5	0	0	0	0	0	0	0	13	0	141.5	0	0	0	0	0	0	0	0	0	0	0		1.25
546 RYNNKFSSSNNS VYN	0	0	57	3870.5	0	0	0	0	0	0	0	0	0	0	0	0	0	0	1206.25	0	59.25	0	0	0	0	0	17	0	0	0	0	0		1.13
547 YNNKFSSSNNS VYNV	0	0	0	3822	0	0	0	0	0	0	15.5	0	0	0	0	0	0	0	0	0	0	0	0	0	0	0	21	0	0	0	0	0		1.37
548 NNKFSSSNNS VYNVQ	0	0	0	1172.5	0	0	0	6	0	0	0	34.5	0	0	0	0	0	0	0	0	0	0	0	0	0	0	49.25	0	0	0	0	0		1.34
549 NKFSSSNNSVYNVQK	0	0	0	321	0	0	0	0	0	0	0	0	0	0	0	0	0	0	0	0	0	0	0	0	0	0	0	0	0	0	0	0		1.37
550 KFSSSNNSVYNVQKL	0	0	0	0	0	0	0	0	0	0	0	0	0	0	0	0	0	0	0	0	0	0	0	0	0	687.5	0	0	0	0	0	48.5		-0.40
551 FSSSNNSVYNVQKLK	0	0	41.5	0	0	0	0	0	0	0	0	0	0	0	0	38	0	0	0	0	0	0	0	7.5	0	0	0	0	0	0	0	0		0.29
552 SSSNNSVYNVQKLKK	0	0	706	1312.25	0	0	0	413	0	0	29	0	0	0	0	286	0	165	0	0	28	0	0	472.75	0	0	68	0	0	104	0	0		2.22
553 SSNNSVYNVQKLKKA	0	0	0	1148.5	0	0	0	190.5	0	0	0	0	0	0	0	739	0	0	0	655.75	3	0	0	0	0	0	0	0	0	38	0	0		0.31
554 SNNSVYNV QKLKKAL	0	0	2088.5	3061	0	0	0	56.5	0	0	0	0	0	0	0	468.5	0	91.5	0	0	0	0	0	0	0	0	0	0	0	0	0	0		3.23
555 NNSVYNV QKLKKALS	0	0	0	1875.5	0	0	0	6.5	0	0	0	0	0	0	0	390.5	0	0	0	0	0	0	0	71	0	0	0	0	0	0	0	0		1.19
556 NSVYNV QKLKKALSY	0	0	505	1744.25	0	0	0	0	0	0	0	0	0	0	0	307.5	0	0	0	0	0	0	0	0	0	0	0	0	0	0	0	0		2.26
557 SVYNV QKLKKALSYL	0	0	0	1212.25	0	0	0	0	0	0	0	0	0	0	0	117.5	0	0	0	0	0	0	0	0	0	0	0	0	0	0	0	0		1.31
558 VYNV QKLKKALSYLE	194.5	0	149	444	0	0	0	0	0	0	0	0	0	0	0	0	0	0	0	0	0	0	0	0	0	0	0	0	56.5	0	0	0		4.66
559 YNVQKLKKALSYLED	755	0	415.5	0	0	0	0	0	0	0	0	0	0	0	0	0	155	88.5	0	222	0	0	0	0	0	0	0	0	0	0	0	0		2.48
560 NVQKLKKALSYLEDY	630.75	0	533.5	382.5	0	0	0	0	71.5	0	0	0	0	0	0	0	215.25	199.5	43.5	415.75	18	0	0	0	0	0	0	0	0	0	0	0		3.71
561 VQKLKKALSYLEDYS	252	0	147	0	0	0	0	0	0	0	0	0	0	0	0	0	124.5	0	0	0	0	0	0	0	0	0	0	0	0	0	0	0		2.35
562 QKLKKALSYLEDYSL	148.75	0	318.5	235.5	0	0	0	0	0	0	0	0	0	0	0	0	0	97.5	0	0	0	0	0	0	0	0	0	0	0	0	0	0		5.45
563 KLKKALSYLEDYSLR	0	0	0	152.5	0	0	0	0	0	0	0	0	0	0	0	0	16	0	0	0	0	0	0	0	0	331.5	0	0	0	0	0	0		-0.30
564 LKKALSYLEDYSLRK	0	0	0	0	0	0	0	0	0	0	0	0	0	0	0	151.5	0	0	0	0	0	0	0	0	0	58.75	0	0	0	0	0	0		-0.35
565 KKALSYLEDYSLRKG	0	0	0	0	0	0	0	0	0	0	0	0	0	0	0	0	0	0	0	0	0	0	0	0	0	941	0	0	0	0	0	0		-0.41
566 KALSYLEDYSLRKGI	0	0	0	0	0	0	0	0	0	0	0	0	0	0	0	0	0	0	0	0	0	0	0	0	0	403.5	0	0	0	0	0	0		-0.41
567 ALSYLEDYSLRKGIS	0	0	0	0	0	0	0	0	0	0	0	0	0	0	0	0	0	0	0	0	0	0	0	0	0	0	0	0	0	0	0	0		
568 LSYLEDYSLRKGISE	0	0	369.25	0	13.5	16	0	0	0	0	0	0	0	0	0	0	0	0	0	0	31	0	0	0	40	0	0	0	0	0	0	0		1.25
569 SYLEDYSLRKGISEK	0	0	0	0	0	0	0	0	0	0	0	54.5	143	0	239.75	0	1624	238	266	1774.5	0	0	0	0	0	0	115.5	0	0	545	0	150.5		-0.16
570 YLEDYSLRKGISEK D	0	0	404	0	20.5	67	0	0	810	399.5	0	504	4877	594	235.75	926.75	20569.25	6073.75	4405	16361.5	2745	212	36	519	316	1481.75	2808.5	164.5	117.25	3077.25	3917.25	3483.5		0.02
571 LEDYSLRKGISEK DF	0	0	256	690.25	66.5	0	0	0	1503.25	0	133	933.5	4097.75	257	564.75	1920	34041	4888	1976	5801.5	0	46.5	27.5	0	0	1395.25	782.5	73.5	332.5	5527	829	1927		-0.25
572 EDYSLRKGISEK DFN	0	0	0	0	59.5	0	0	0	40.5	17	66	47.75	487.75	89	0	522	7769.5	3018.25	723	12213.5	440.25	0	0	6	0	0	155	0	45	0	210.25	762.25		-0.23
573 DYSLRKGISEK DFNH	0	0	0	4665.25	0	0	0	0	0	0	0	0	315.5	0	0	0	2032.5	639.5	0	2556.25	0	0	0	0	0	0	0	0	0	0	0	0		0.44
574 YSLRKGISEKDFNHY	0	0	0	0	0	0	0	0	0	0	0	0	0	0	0	0	624	220	0	0	0	0	0	0	0	0	0	0	0	0	0	0		-0.36
575 SLRKGISEKDFNHYY	0	93.5	0	0	0	0	0	0	0	0	0	0	0	0	0	0	0	0	0	0	0	0	0	0	0	0	0	0	0	0	0	0		1.37
576 LRKGISEKDFNHYYT	0	0	0	0	0	0	0	0	0	0	0	0	0	0	0	0	0	0	0	0	0	0	0	0	0	0	0	0	0	0	0	0		
577 RKGISEKDFNHYYTL	0	364	0	0	0	0	22	0	0	0	0	0	0	0	0	0	0	0	0	0	0	0	0	0	0	0	0	0	0	0	0	0		1.37
578 KGISEKDFNHYYTLK	0	200	0	0	0	0	0	0	0	0	0	0	0	0	0	0	457.25	0	0	0	0	0	0	0	0	536.75	0	0	0	0	0	0		-0.53
579 GISEKDFNHYYTLKT	0	387.25	0	0	0	0	0	0	0	0	0	0	0	0	0	0	0	0	0	0	0	0	0	0	0	0	0	0	0	0	0	0		1.37
580 ISEKDFNHYYTLKTG	0	481.5	0	0	0	0	0	0	0	0	0	0	0	0	0	0	0	0	0	0	0	0	0	0	0	0	0	0	0	0	0	0		1.37
581 SEKDFNHYYTLKTGL	0	655.25	0	0	0	0	0	0	0	0	0	0	0	0	0	0	0	0	0	0	0	0	0	0	0	0	0	0	0	0	0	0		1.37
582 EKDFNHYYTLKTGLE	770.25	68	0	2465.25	0	0	0	0	0	2036	0	0	176.5	131	0	0	0	0	0	0	0	0	0	0	0	0	0	0	0	7.25	0	0		1.09
583 KDFNHYYTLKTGLEA	203	0	0	0	0	0	0	0	0	0	0	0	0	0	0	0	0	0	0	0	0	0	0	0	0	0	0	0	0	0	0	0		1.37
584 DFNHYYTLKTGLEAD	138.75	0	59	0	0	0	0	0	17	0	0	0	0	0	0	178	122	15.5	0	0	0	39	0	7.75	0	0	0	53	0	0	0	0		0.08
585 FNHYYTLKTGLEADI	87	0	0	1631.25	0	0	0	0	0	0	0	0	0	0	0	0	541.5	30.5	0	665.5	0	0	0	0	0	0	0	0	0	0	0	0		0.87
586 NHYYTLKTGLEADIK	0	873.5	0	0	0	0	0	0	0	0	0	0	0	0	0	0	0	0	0	0	0	0	0	0	0	0	0	0	0	0	0	0		1.37
587 HYYTLKTGLEADIKK	0	0	0	0	0	0	0	0	0	0	0	0	0	0	0	0	0	0	0	0	0	0	0	0	48	0	0	0	0	0	0	0		-0.41
588 YYTLKTGLEADIKKL	0	0	144.75	0	0	0	0	0	0	0	0	0	0	0	0	0	0	0	0	0	0	0	0	0	58	0	0	0	0	0	0	0		0.97
589 YTLKTGLEADIKKLT	0	0	204.75	0	0	0	0	0	0	0	0	0	0	0	0	0	0	0	0	0	0	0	0	0	0	0	0	0	0	0	0	0		1.37
590 TLKTGLEADIKKLTE	96.5	0	646	0	0	0	0	0	0	0	0	0	0	0	0	0	0	0	0	0	0	0	0	0	30.75	0	0	49	0	0	0	0		1.86
591 LKTGLEADIKKLTEE	0	0	3357.75	366	22	0	0	0	28.5	33.5	0	0	244	20.75	8.5	0	0	0	0	19	0	0	23	0	35.5	0	0	159.5	0	0	0	7.5		1.68
592 KTGLEADIKKLTEEI	0	49	3755	2702	13.5	0	0	0	0	1072.5	0	0	38.25	21	10	35	0	0	0	0	0	2	0	30.5	413	222	0	60.5	0	0	0	31		2.99
593 TGLEADIKKLTEEIK	225.25	0	2008.5	199.5	8	0	0	0	0	6.5	0	21	27.5	0	0	0	0	0	0	0	0	10.5	40.5	0	0	0	0	0	0	0	0	0		2.20
594 GLEADIKKLTEEIKS	0	0	2757	0	14	6	0	0	0	0	0	0	0	0	0	0	0	0	0	0	0	0	5	0	49.5	0	0	0	0	0	0	0		1.36
595 LEADIKKLTEEIKSS	0	0	3430.75	0	0	0	0	0	0	0	0	0	346.25	0	0	0	0	0	0	48.5	0	0	0	0	13.5	0	0	0	0	0	0	0		1.30
596 EADIKKLTEEIKSSE	0	0	5012.75	841	19	0	0	0	0	196	16.5	0	560.5	166.5	17.5	0	0	4	54	233	0	3	0	0	0	0	23.5	61.5	0	0	0	0		1.83
597 ADIKKLTEEIKSSEN	0	0	185	1747.5	5	0	0	0	0	0	0	10.75	0	0	0	0	0	0	0	0	0	0	0	0	0	0	0	23	0	0	0	0		1.76
598 DIKKLTEEIKSSENK	0	0	191.75	517	0	0	0	0	0	0	0	0	0	0	0	35	0	0	0	0	0	0	0	0	0	0	0	0	0	0	0	0		2.66
599 IKKLTEEIKSSENKI	0	0	0	562.5	0	0	0	0	0	0	0	11.5	0	0	0	574.75	0	0	0	0	0	0	0	0	0	52.5	41.5	0	0	0	0	0		0.21
600 KKLTEEIKSSENKIL	0	0	0	0	0	0	0	0	0	0	0	0	0	0	0	435	0	0	0	0	26	0	0	0	0	672.25	0	0	0	0	0	0		-0.33
601 KLTEEIKSSENKILE	146	0	0	317.25	21.5	0	0	0	12	44.25	0	83	0	0	0	259	0	0	0	0	0	0	0	0	0	0	0	85.5	0	0	0	8		1.04
602 LTEEIKSSENKILEK	0	0	0	0	23	0	13.5	0	0	0	0	119	44.5	0	0	0	0	0	0	47	0	0	0	0	0	0	0	18.5	0	0	0	0		-0.26
603 TEEIKSSENKILEKN	0	0	0	1969.75	0	0	0	0	0	0	3	0	1938.5	0	0	0	0	0	0	0	0	0	0	0	0	0	0	0	0	0	0	0		0.28
604 EEIKSSENKILEKNF	0	0	0	7015.5	24	66.5	0	0	0	82	0	0	2524.25	209	0	0	0	0	0	0	0	0	50.5	0	0	0	0	0	0	0	0	0		1.01
605 EIKSSENKILEKNFK	0	0	0	0	0	0	0	0	0	0	0	0	198	0	0	0	0	0	0	0	0	0	294.5	0	0	0	0	0	0	0	0	0		-0.33
606 IKSSENKILEKNFKG	0	0	0	0	0	0	0	0	0	0	0	134.5	0	0	0	0	0	0	0	0	0	0	598	0	0	206	0	0	0	0	0	0		-0.32
607 KSSENKILEKNFKGL	0	0	0	0	0	0	0	0	0	0	0	120	0	0	0	0	0	0	0	0	0	0	0	0	0	936.5	0	0	0	0	0	0		-0.39
608 SSENKILEKNFKGLT	0	0	0	0	0	0	0	0	0	8	0	0	0	0	0	0	0	0	0	0	0	0	0	0	0	0	0	0	0	0	0	0		-0.41
609 SENKILEKNFKGLTH	0	0	0	787	0	0	0	0	0	0	0	0	0	0	0	0	8.5	0	0	169.5	0	0	0	0	0	0	0	0	0	261	17.5	0		0.91
610 ENKILEKNFKGLTHS	301.5	0	0	2105.75	0	0	0	0	0	0	0	0	0	0	0	0	11.5	0	0	206.5	0	0	0	0	0	0	0	0	0	289.5	50.5	0		1.72
611 NKILEKNFKGLTHSA	311.5	0	0	963	0	0	0	0	0	0	0	0	0	0	32.5	0	0	0	0	189.5	0	0	0	0	0	0	0	0	0	175.5	0	0		2.15
612 KILEKNFKGLTHSAN	0	0	0	0	0	0	0	0	0	0	0	0	0	0	0	0	0	0	0	0	0	0	0	0	0	702	0	0	0	284	0	0		-0.35
613 ILEKNFKGLTHSANG	0	0	0	0	0	0	0	0	0	0	0	0	0	0	0	0	0	0	0	69.5	0	0	0	0	0	0	0	0	0	0	0	0		-0.41
614 LEKNFKGLTHSANGS	0	0	0	680.5	0	0	0	0	0	0	0	0	0	0	0	0	0	0	59	94	0	0	0	0	0	0	0	0	0	6.75	0	0		1.22
615 EKNFKGLTHSANGSL	0	0	0	1652.5	0	0	0	0	0	0	0	0	0	75.5	0	0	0	0	0	0	0	0	0	0	0	0	0	0	0	0	0	0		1.35
616 KNFKGLTHSANGSLE	0	0	0	2078.75	0	0	0	0	0	0	0	0	0	0	0	0	0	0	58.5	0	0	0	0	0	0	146	0	0	0	0	0	0		1.32
617 NFKGLTHSANGSLEV	0	261.5	0	1027.75	0	0	0	0	0	0	0	11	0	0	0	0	0	0	0	0	0	0	0	0	0	0	0	0	0	0	0	0		2.32
618 FKGLTHSANGSLEVS	0	0	0	0	3	0	0	0	0	0	0	0	0	0	0	0	0	145.5	0	59	0	0	0	0	0	0	0	0	0	20	0	0		-0.32
619 KGLTHSANGSLEVSD	0	0	184.5	456	5	0	0	0	0	0	0	0	0	0	0	0	0	0	0	0	0	0	0	0	0	185	0	27	0	7	0	0		2.12
620 GLTHSANGSLEVSDI	23.25	0	73	3585	16	5	0	0	0	0	0	0	0	0	0	0	31.5	0	0	0	0	0	0	0	0	0	5	26	9	0	0	7		1.46
621 LTHSANGSLEVSDIV	55.5	0	188	4637.5	18	0	0	0	0	0	0	51.5	0	0	0	0	0	0	0	207.75	0	0	8.5	0	0	0	0	11	0	0	0	0		1.54
622 THSANGSLEVSDIVK	0	0	0	0	0	0	0	0	0	0	0	0	449.25	0	0	0	0	0	0	0	0	0	0	0	0	37	0	0	0	0	0	0		-0.40
623 HSANGSLEVSDIVKL	0	0	0	887.5	0	0	0	0	0	0	0	0	0	0	0	0	0	0	0	0	0	0	0	0	0	0	0	0	0	0	0	0		1.37
624 SANGSLEVSDIVKLQ	0	0	0	0	0	0	0	0	0	0	3.5	0	0	0	0	0	0	0	0	0	0	0	0	0	0	0	0	0	0	0	0	0		-0.41
625 ANGSLEVSDIVKLQV	0	0	0	0	0	0	0	0	0	0	0	0	0	0	0	85.5	0	0	0	0	0	0	0	0	0	0	0	0	0	0	0	0		-0.41
626 NGSLEVSDIVKLQVQ	0	0	0	0	6	0	0	0	0	0	0	0	0	0	0	0	0	0	0	0	0	0	0	0	0	0	0	0	0	0	0	0		
627 GSLEVSDIVKLQVQK	0	0	152.5	0	14.5	0	0	0	0	0	0	0	0	0	0	0	0	0	0	0	0	0	0	0	0	0	0	0	0	0	0	0		1.37
628 SLEVSDIVKLQVQKV	0	0	31.5	0	0	0	0	0	0	0	0	0	0	0	0	0	0	0	0	0	0	0	0	0	0	0	0	0	0	0	0	0		1.37
629 LEVSDIVKLQVQKVL	0	0	0	0	0	0	0	0	0	0	0	0	0	0	0	0	0	0	0	0	0	0	0	0	0	0	0	0	0	0	0	0	7	
630 EVSDIVKLQVQKVLL	0	0	0	0	0	0	0	0	0	7	0	0	0	56	0	0	0	0	0	0	0	0	0	0	0	0	0	0	0	0	0	0		-0.39
631 VSDIVKLQVQKVLLI	0	0	0	0	0	0	0	0	0	0	0	0	0	0	0	0	0	0	0	0	0	0	0	0	0	0	0	0	0	0	0	0		
632 SDIVKLQVQKVLLIK	0	0	0	0	0	0	0	0	0	0	0	0	0	0	0	0	0	0	0	0	0	0	0	0	0	0	0	0	0	0	0	0		
633 DIVKLQVQKVLLIKK	0	7.5	97.5	0	12.25	11.5	9.5	0	0	0	0	0	0	19	0	0	0	177	0	0	0	0	0	188	0	0	0	0	0	68.5	0	0		-0.47
634 IVKLQVQKVLLIKKI	0	0	0	1649.75	0	0	0	0	0	0	0	0	0	0	0	35	0	0	0	0	0	0	0	110.5	0	65	0	0	0	190	0	0		1.23
635 VKLQVQKVLLIKKIE	2037.25	0	0	0	0	0	0	0	0	0	0	0	0	0	0	0	0	0	0	0	0	0	0	0	0	52	0	0	0	0	0	0		1.36
636 KLQVQKVLLIKKIED	11087.75	0	0	0	0	0	0	0	0	0	0	0	0	0	0	18.75	0	0	0	0	0	0	0	0	0	69	0	0	0	0	0	0		1.37
637 LQVQKVLLIKKIEDL	10167.5	0	0	0	0	0	0	0	0	0	0	0	0	0	0	0	0	0	0	0	0	0	0	0	0	0	0	0	0	0	0	0		1.37
638 QVQKVLLIKKIEDLR	1951.5	0	217.25	0	0	0	0	0	0	0	0	0	0	0	0	0	0	0	0	0	0	0	0	0	0	0	0	0	0	0	0	0		1.79
639 VQKVLLIKKIEDLRK	0	0	0	0	0	0	0	0	0	0	0	0	0	0	0	0	0	0	0	0	0	0	0	0	0	0	0	0	0	0	0	0		
640 QKVLLIKKIEDLRKI	0	0	0	0	0	0	0	0	0	0	0	0	0	0	0	0	0	0	0	0	0	0	0	0	0	0	0	0	0	0	0	0		
641 KVLLIKKIEDLRKIE	5224.5	0	127.5	0	0	0	0	0	0	0	0	0	0	0	0	0	63	0	0	0	0	0	0	0	0	94.5	0	0	0	0	0	0		1.45
642 VLLIKKIEDLRKIEL	8284	0	165	0	7	0	0	0	0	0	0	0	0	0	15	4	0	0	0	0	0	77.5	0	0	0	0	0	0	0	0	0	0		1.44
643 LLIKKIEDLRKIELF	5992.25	0	130	0	23.5	0	10.5	7.5	20	0	0	0	0	0	0	9.5	0	18	0	0	25.5	0	10.5	0	0	0	0	0	0	0	17.5	17		1.44
644 LIKKIEDLRKIELFL	2907	0	129	0	0	0	0	7	0	0	0	0	0	0	5.5	0	0	6.25	11	0	10	0	0	0	0	0	0	4.5	91	0	7	0		1.51
645 IKKIEDLRKIELFLK	0	0	0	0	0	0	0	0	0	0	0	0	0	0	0	0	0	0	0	0	0	0	0	0	0	107.75	0	0	0	0	0	0		-0.41
646 KKIEDLRKIELFLKN	437.5	0	0	133	0	0	0	0	0	0	0	0	0	0	0	0	0	0	0	0	0	0	0	0	0	536	0	0	0	0	0	0		0.50
647 KIEDLRKIELFLKNA	252	0	0	0	0	0	0	0	0	0	0	0	0	0	0	0	0	0	0	0	0	0	0	0	0	117	0	0	0	0	0	0		0.89
648 IEDLRKIELFLKNAQ	355.5	0	0	0	0	0	0	0	0	0	0	0	0	0	0	0	0	0	0	0	0	0	0	0	0	0	0	0	0	0	0	0		1.37
649 EDLRKIELFLKNAQL	0	0	0	0	0	0	0	0	0	0	0	0	50	138.5	0	0	0	0	0	0	0	0	0	0	0	0	0	0	0	0	0	0		-0.36
650 DLRKIELFLKNAQLK	0	0	0	0	0	0	0	0	0	0	0	0	0	0	0	0	0	0	0	0	0	0	0	0	0	0	0	0	0	0	0	0		
651 LRKIELFLKNAQLKD	0	0	0	0	0	0	0	0	0	0	0	0	0	0	0	0	0	0	0	0	0	0	0	0	0	0	0	0	0	0	0	0		
652 RKIELFLKNAQLKDS	0	0	0	1012.5	0	0	0	0	0	0	0	0	0	0	0	0	0	0	0	0	0	0	0	0	0	0	0	0	0	0	0	0		1.37
653 KIELFLKNAQLKDSI	0	0	0	0	0	0	0	0	0	0	0	0	0	0	0	0	0	0	0	0	0	0	0	0	0	0	0	0	0	0	0	0		
654 IELFLKNAQLKDSIH	0	0	0	121	0	0	0	0	0	0	0	0	0	0	0	0	0	0	1.5	0	0	0	11.75	0	0	0	0	0	0	0	0	0		1.31
655 ELFLKNAQLKDSIHV	0	0	0	0	0	0	0	0	0	34	0	0	75	148	0	107.25	370.25	0	0	0	0	0	31	0	0	0	0	0	128.75	0	0	0		-0.16
656 LFLKNAQLKDSIHVP	0	0	0	0	0	0	0	0	0	0	0	0	62.5	0	0	74.25	324.5	0	17	0	0	0	0	0	0	0	0	0	0	0	0	0		-0.33
657 FLKNAQLKDSIHVPN	0	0	0	0	0	0	0	0	0	0	0	0	130	0	0	33.5	325	0	0	0	0	0	0	0	0	0	0	0	0	0	0	0		-0.33
658 LKNAQLKDSIHVPNI	0	0	0	0	0	0	0	0	0	0	0	0	26.5	0	0	113.5	220	0	0	0	582.25	0	0	0	0	25	0	0	0	0	0	0		-0.30
659 KNAQLKDSIH VPNIY	2540.25	0	0	0	0	0	0	0	10.5	0	0	0	10.5	0	0	0	468	0	0	0	0	0	0	0	0	1503.5	16	0	0	0	0	0		0.63

660	NAQLKDSIHVPNIYK	616	0	0	0	0	0	0	0	2	0	0	47	0	0	0	0	645	92.5	0	0	0	0	0	0	0	0	0	0	0	697.25	0	0		-0.19
661	AQLKDSIHVPNIYKP	3896	211.5	0	0	0	0	0	0	0	0	0	146	3	0	0	50	1347	318.5	0	670	193.75	0	0	46.5	0	75	0	0	0	1710.75	209.5	234		0.65
662	QLKDSIHVPNIYKPQ	1044.5	0	0	0	0	0	0	0	142.5	0	49	212	0	0	0	34	1521	257	0	438.5	200.5	0	0	113	0	0	0	0	0	2015.75	2439.5	278.75		-0.51
663	LKDSIHVPNIYKPQN	3449	337.5	0	0	0	0	0	0	63.5	0	0	102	79	0	0	0	596.5	181.5	0	325.5	185.5	0	9	0	0	43.5	0	0	0	3554.25	2093.5	177		0.07
664	KDSIHVPNIYKPQNK	5754	694.75	0	1118	0	0	0	0	0	0	0	173.75	0	68.5	0	0	567.25	309.5	0	0	0	0	0	114	0	1585.5	0	0	0	3723.5	1629.75	355.25		1.03
665	DSIHVPNIYKPQNKP	3914.5	1861	0	0	0	0	0	0	0	0	0	110	0	0	0	152	611	1035	0	109.5	133.5	0	0	0	0	0	0	0	0	671.75	2365	0		1.47
666	SIHVPNIYKPQNKPE	1219.5	580	0	0	0	0	0	0	0	0	0	0	0	0	0	476.75	298.25	579.5	0	3227.75	41	0	0	0	0	0	0	0	0	441	0	38.5		-0.28
667	IHVPNIYKPQNKPEP	1073.5	1540.5	0	0	0	0	0	0	0	0	0	64	0	0	0	1590.5	221.5	344.75	0	3929.5	0	0	7.25	0	0	0	0	0	0	760.5	962.5	0		-0.26
668	HVPNIYKPQNKPEPY	0	1127.75	0	430	0	0	0	0	29	0	0	45	0	0	0	1557.75	119.5	349	0	959.5	46.5	0	0	0	0	0	0	0	20	582.75	502	73		-0.10
669	VPNIYKPQNKPEPYY	0	507.25	0	486.75	0	0	0	0	24.5	0	0	0	0	0	0	1044	0	168.5	87	1558.75	38	0	0	0	0	0	0	0	38.5	0	185.5	0		-0.32
670	PNIYKPQNKPEPYYL	0	488	0	224.5	0	0	0	0	0	0	0	0	0	0	0	650.75	0	0	81.5	1079	0	0	0	0	0	0	0	0	68	0	0	0		-0.24
671	NIYKPQNKPEPYYLI	0	334.5	0	0	0	0	0	0	0	0	0	4	0	0	0	618.25	0	0	176	597	0	0	0	0	0	0	0	0	0	0	0	0		-0.47
672	IYKPQNKPEPYYLIV	0	232	0	0	0	0	0	0	0	0	0	0	0	0	0	339.5	0	0	19.5	298.5	0	0	0	0	0	0	0	0	0	0	0	0		-0.31
673	YKPQNKPEPYYLIVL	0	0	0	0	0	0	0	0	0	0	0	0	0	0	0	0	0	0	0	0	0	0	0	0	0	50	0	0	0	0	0	0		-0.41
674	KPQNKPEPYYLIVLK	0	0	0	0	0	0	0	0	0	0	0	0	0	0	0	215.5	0	0	0	0	0	0	0	0	0	1409.5	0	0	0	0	0	0		-0.38
675	PQNKPEPYYLIVLKK	1692.5	396	0	0	0	0	0	0	0	0	0	0	0	0	0	703.25	0	0	0	538	0	0	0	0	0	0	0	0	0	0	0	0		1.38
676	QNKPEPYYLIVLKKE	252.75	0	35.75	0	0	0	0	0	0	0	0	0	0	0	0	32.5	0	0	0	0	0	25	0	0	0	0	0	0	0	0	0	0		1.74
677	NKPEPYYLIVLKKEV	429	0	0	0	0	4	0	0	0	0	0	40	188	0	0	0	0	0	0	273	4	638	0	0	0	0	0	0	0	0	50.5	0		-0.26
678	KPEPYYLIVLKKEVD	0	28	0	0	0	0	0	0	0	0	0	19	21	0	0	800	14	1518.5	0	0	829.5	35.5	20.5	0	95.5	80	0	425.75	79.5	0	0	24		-0.19
679	PEPYYLIVLKKEVDK	0	0	0	0	0	31.25	0	0	0	0	0	0	0	0	0	0	0	0	0	0	0	0	0	0	0	0	0	0	0	0	0	0		
680	EPYYLIVLKKEVDKL	0	41.5	0	0	97.5	32	0	0	0	0	0	0	0	0	0	0	28	0	0	0	37	0	0	0	0	0	0	0	0	6.5	0	186		-0.57
681	PYYLIVLKKEVDKLK	0	0	0	0	0	0	0	0	0	0	0	0	0	0	0	0	0	0	0	0	0	0	0	0	0	0	0	0	0	0	0	0		
682	YYLIVLKKEVDKLKE	0	0	0	0	0	0	0	0	0	0	0	0	0	0	0	0	0	0	0	0	0	50.5	0	0	0	0	0	0	0	0	0	0		-0.41
683	YLIVLKKEVDKLKEF	0	0	0	0	0	0	0	0	0	0	0	0	0	0	0	0	0	0	0	0	0	433	0	0	0	0	0	0	0	0	0	0		-0.41
684	LIVLKKEVDKLKEFI	0	0	0	118	0	0	0	0	0	0	0	0	0	0	0	0	0	0	0	54.5	0	119.5	0	0	0	0	0	0	0	0	0	0		0.11
685	IVLKKEVDKLKEFIP	0	738.75	0	0	0	0	0	0	0	0	0	0	0	0	0	0	0	0	0	0	0	0	0	0	0	0	0	3.5	0	0	0	0		1.37
686	VLKKEVDKLKEFIPK	0	746.5	0	0	0	36.5	0	0	0	0	0	0	0	0	0	0	0	0	0	0	0	0	0	0	0	0	0	0	0	0	0	0		1.37
687	LKKEVDKLKEFIPKV	0	604.5	0	0	0	0	0	0	0	0	0	0	0	0	0	0	0	0	0	0	0	0	0	0	0	206	0	0	0	0	0	0		1.05
688	KKEVDKLKEFIPKVK	0	289	0	0	0	0	0	68.5	0	0	0	0	0	41.5	0	0	0	56.5	0	0	0	11.5	0	0	0	1791.75	0	0	0	39.5	0	0		-0.56
689	KEVDKLKEFIPKVKD	0	311	0	0	0	13.5	0	0	62.5	23	0	0	0	0	0	0	0	28.75	0	0	0	45.75	0	0	0	251	0	0	0	0	0	0		0.31
690	EVDKLKEFIPKVKDM	0	317.5	0	44.5	0	22.5	25	0	0	432.75	5	0	148.5	67	10	44.5	0	27	0	487.75	0	0	0	0	0	0	0	25.5	0	0	0	0		-0.35
691	VDKLKEFIPKVKDML	0	141.5	0	0	0	18	0	0	0	0	0	16.5	176.5	0	0	0	0	0	0	425	0	0	2	0	0	0	0	0	0	51.5	0	0		-0.52
692	DKLKEFIPKVKDMLK	0	46.5	0	0	0	32.25	0	0	0	0	0	0	0	0	0	14	0	0	0	652.75	8	0	0	0	0	13	0	0	0	21.25	0	0		-0.47
693	KLKEFIPKVKDMLKK	0	0	0	195.5	7	0	0	280.5	0	57.5	0	0	13.75	142	0	99	0	101	0	0	0	1.5	0	373	0	692.75	83.5	0	0	114.75	0	0		-0.47
694	LKEFIPKVKDMLKKE	101.5	0	0	0	0	5	0	0	0	0	0	0	0	0	0	0	0	14.5	0	71.5	0	214.5	0	0	0	41.5	0	0	0	20.5	0	0		-0.41
695	KEFIPKVKDMLKKEQ	0	0	0	0	0	0	0	0	0	0	0	0	0	0	0	0	0	0	0	46	0	321.25	0	0	0	133	0	0	0	0	0	0		-0.32
696	EFIPKVKDMLKKEQA	0	0	0	0	3.5	0	0	0	0	0	0	0	64.5	0	0	0	22.75	0	0	71	0	0	0	0	0	0	0	0	0	0	0	0		-0.28
697	FIPKVKDMLKKEQAV	0	0	0	0	0	0	0	0	0	0	0	0	0	0	0	0	0	0	0	0	0	0	0	0	0	0	0	0	0	0	0	0		
698	IPKVKDMLKKEQAVL	0	0	0	0	0	0	0	0	0	0	0	0	0	0	0	0	99	0	0	0	0	0	0	0	0	0	0	0	0	0	0	0		-0.41
699	PKVKDMLKKEQAVLS	0	0	0	0	0	0	0	0	0	0	0	0	0	0	0	0	92	0	0	0	0	0	0	0	0	0	0	0	0	0	0	0		-0.41
700	KVKDMLKKEQAVLSS	0	0	0	8569.75	0	0	0	0	0	0	0	0	0	231.25	0	0	0	0	0	101	0	0	0	326	0	420	0	0	0	0	0	0		1.31
701	VKDMLKKEQAVLSSI	352.25	0	0	0	0	0	0	0	0	0	0	0	0	1120.75	0	272.5	0	0	0	2905	0	0	0	604.5	0	0	0	0	0	0	0	0		-0.43
702	KDMLKKEQAVLSSIT	778.5	272	0	0	0	0	0	0	0	0	0	0	0	294	0	9871	0	0	0	32201.5	0	0	0	564.5	0	133	485.5	0	0	0	0	0	8	-0.39
703	DMLKKEQAVLSSITQ	933.5	501	0	0	0	0	91.75	0	0	0	0	0	0	0	0	8952	0	0	0	19230.25	0	0	0	342	0	0	132.25	0	0	0	21	0		-0.42
704	MLKKEQAVLSSITQP	4747	6552.25	0	0	0	0	0	0	0	0	0	0	0	0	0	7167.25	1	0	0	19723	0	10.5	175.5	130.75	0	0	77.5	0	0	0	0	0		-0.23
705	LKKEQAVLSSITQPL	4144	6782.5	0	0	0	0	0	0	0	0	0	0	0	0	0	6264.5	0	0	0	20078.75	0	0	278.5	89	0	0	0	0	0	0	0	0		-0.25
706	KKEQAVLSSITQPLV	2080.5	5697.75	0	0	0	0	0	0	0	0	0	0	0	0	0	3765	0	0	0	4176.25	0	0	92	132	0	595	0	0	0	0	0	0		0.77
707	KEQAVLSSITQPLVA	2366.5	5114.5	0	0	0	0	0	0	0	0	0	0	293	0	0	4088.75	0	0	0	3487.5	0	420	38.5	13.25	0	0	0	0	0	0	0	0		0.83
708	EQAVLSSITQPLVAA	1908.5	5109.75	0	0	0	0	0	0	0	0	0	0	289	39	0	0	0	0	0	1533.5	0	0	36	0	0	0	0	0	0	0	0	0		2.27
709	QAVLSSITQPLVAAS	1754	6695.75	0	661	0	0	0	0	0	0	0	0	0	0	0	0	0	0	0	1308	0	0	12	0	0	0	0	0	0	0	0	0		2.62
710	AVLSSITQPLVAASE	3762	10174.5	0	78.5	0	0	0	0	188.75	0	0	0	0	0	0	0	247.5	25	0	1588.5	0	95	62	0	0	0	492.5	0	0	0	10.5	248.5		2.51
711	VLSSITQPLVAASET	4287	11682.75	0	0	21	0	0	0	353.5	0	0	0	0	0	0	79	0	21	0	5189.75	0	131	241.5	0	0	0	556.5	0	0	0	23	299.25		1.91
712	LSSITQPLVAASETT	2203	6888.25	0	0	0	0	0	0	649.75	0	0	51.5	0	0	0	142.5	0	0	0	6485.25	0	159	238	0	0	0	693	0	0	0	48.5	366		0.79
713	SSITQPLVAASETTE	655.25	7399	0	0	77.25	0	68.5	50	1269.5	0	1017.5	0	97	0	0	267	0	39.5	0	5622.5	0	224	294	26.5	0	0	870.5	84.5	0	0	192.5	752		0.48
714	SITQPLVAASETTED	337.25	1566	475	748.5	0	7.5	309.5	35.5	1667.5	12.5	70.5	0	19.5	0	0	575.75	0	109.5	45.5	3464.25	0	261.5	118	0	0	0	3047	182	11	0	262	785		-0.27
715	ITQPLVAASETTEDG	0	786.5	0	0	0	0	0	0	0	0	0	0	0	0	0	209.25	0	26.5	11.5	0	0	66.5	0	0	0	0	91.5	63.5	0	0	67.5	222.5		0.76
716	TQPLVAASETTEDGG	0	4100.75	0	0	0	0	0	0	150	0	0	0	0	0	0	54	0	0	0	0	0	103	0	0	0	0	36	0	0	0	72	497.5		1.24
717	QPLVAASETTEDGGH	0	10011.5	0	586.5	0	0	0	0	0	0	0	0	0	0	0	114.5	0	0	15	8	0	71	5.5	0	0	0	410.5	0	0	0	62.5	368.5		1.54
718	PLVAASETTEDGGHS	0	7629.25	0	0	0	0	32.5	0	0	0	29	0	0	0	0	0	0	0	0	0	0	0	148.5	0	0	0	8.25	232.25	0	0	39	0		1.34
719	LVAASETTEDGGHST	115.5	11968.5	0	0	0	0	0	0	0	0	0	0	0	0	0	66.25	0	0	1.5	0	0	0	0	0	0	0	0	0	0	0	0	0		1.41
720	VAASETTEDGGHSTH	0	9807.75	0	0	0	8.5	0	0	77.5	0	0	0	0	0	0	179	0	0	0	46	0	0	0	0	6.75	0	144.5	0	0	0	0	0		1.35
721	AASETTEDGGHSTHT	0	16221.5	0	0	0	0	0	0	76	0	0	0	0	0	0	107	0	0	5	29.5	0	0	0	0	0	0	0	0	35	93	0	0		1.36
722	ASETTEDGGHSTHTL	423.25	12834.5	0	0	0	0	0	0	75	0	0	0	0	0	0	0	0	0	0	24	0	0	22	0	0	0	0	0	0	156.5	0	0		1.49
723	SETTEDGGHSTHTLS	336.25	6730.5	0	0	0	0	0	0	70.5	0	0	0	0	0	0	0	0	0	0	0	0	0	0	0	0	0	0	0	0	257.5	0	0		1.53
724	ETTEDGGHSTHTLSQ	0	2587.25	0	875	0	0	0	0	0	46.25	0	0	276.25	118.5	0	0	0	0	0	0	0	0	775.25	0	0	0	0	0	0	210	0	0		2.05
725	TTEDGGHSTHTLSQS	8659.75	18738.5	0	726.5	0	0	0	0	0	0	0	0	0	0	0	0	0	0	0	0	0	0	0	0	0	0	0	0	0	0	0	0		3.20
726	TEDGGHSTHTLSQSG	1140	5324.25	0	327.5	15.5	0	0	0	0	0	0	0	0	0	0	0	0	0	0	0	0	0	0	0	0	0	0	0	0	0	0	0		2.48
727	EDGGHSTHTLSQSGE	156	3725.75	0	3365	0	2.25	0	0	0	70.5	0	0	142.5	155	0	32	0	0	0	0	0	0	195	0	0	79	10.5	69.5	0	150	0	0		3.67
728	DGGHSTHTLSQSGET	63545	939.75	0	0	21	0	0	0	0	0	0	0	0	0	0	0	0	0	0	0	0	0	88	0	0	0	0	12.5	0	10.5	0	0		1.43
729	GGHSTHTLSQSGETE	63545	5176.5	0	457.25	25.5	0	0	0	319.5	17.5	0	0	0	0	0	0	0	132.5	0	0	0	0	178.75	0	0	0	0	60.75	0	0	0	0		1.70
730	GHSTHTLSQSGETEV	63545	11043.75	1037.5	0	89	0	0	7	0	18	401	0	0	0	0	0	85.5	0	0	0	0	0	231	0	0	0	0	17.75	0	0	0	6		2.10
731	HSTHTLSQSGETEVT	63545	11024.5	477.25	0	0	0	0	0	0	0	361	0	0	0	4046.5	0	0	252	0	0	0	0	186.5	0	0	91	0	0	0	0	0	0		2.01
732	STHTLSQSGETEVTE	63545	11672.5	1014.25	0	128	17	106.5	0	0	0	155.5	0	126.5	0	0	0	0	0	21	0	8	10.5	210	19	0	139	0	0	0	0	0	20		2.14
733	THTLSQSGETEVTEE	63545	10949.5	868	3390	58.5	105.5	11	8	0	43.5	536.75	21	814.5	0	0	0	16	0	0	0	0	25	381.25	44	0	154	80.5	64.5	250	18	61	55		2.32
734	HTLSQSGETEVTEET	63545	11728.25	775.5	16585.5	0	132	0	0	0	7	0	0	248.25	0	0	0	0	0	0	0	0	0	309.5	26.5	0	87.75	0	0	0	0	0	25		3.38
735	TLSQSGETEVTEETE	63545	10484.5	1112	2858.75	31.5	90.5	38.5	16	0	76.5	3991.75	0	427.5	0	0	0	0	0	36	0	0	84	549.5	58	110.5	176.5	108.25	102	51.5	0	77	634.5		2.23
736	LSQSGETEVTEETEE	63545	3676	1789	3677.25	44	113.5	42	48.5	78	139.5	25332.75	0	727.5	10.25	0	53	0	2745	102.5	0	0	198	661.5	84	582	0	663.25	255.5	87	0	81	3328.5		1.37
737	SQSGETEVTEETEET	10804.5	5940.5	0	10986.5	28.5	229.25	29.5	81	0	67	44250.5	0	372.25	0	0	0	81.5	7407.5	149	81	0	47.5	329.5	61	922.5	0	1314.5	0	60	0	0	2940.75		-0.11
738	QSGETEVTEETEETE	4053.75	0	2133.5	3162	43	166.5	33	142.5	63	110	45848	37.5	969	62.5	48	56	0	8804.75	5	100.5	0	69.75	133.5	215.5	608.75	0	1415.5	215.5	95.5	0	0	3373.5		-0.55
739	SGETEVTEETEETEE	0	0	1999	4384.5	97.25	225.5	67.5	278	105	185	55829	81.5	884.5	42.5	101	161.25	0	9232.75	19.5	273	0	77	238.5	72	911.25	6.5	1401.25	520.25	142	0	30	3931		-0.47
740	GETEVTEETEETEET	218	0	2578.5	11967.75	49.5	135.5	48	337	0	173	55938.75	12	846.5	0	74.5	57.5	0	9894.75	0	368	0	69	522.5	41.75	1280.25	0	2249.5	236	106	0	0	4864		-0.54
741	ETEVTEETEETEETV	4827	0	2316	14501.25	76	184.5	122	409	45	426	55910.75	0	2702.5	101	105	0	0	9906	0	0	0	63	183.5	63	1555	0	2314.75	210.75	84.5	75.5	32	5140.75		-0.43
742	TEVTEETEETEETVG	16376.25	0	1187.75	9509.5	44	139	0	268	89	88.5	56854.25	0	949	109	40	67.5	0	9043.25	0	0	0	56	154	39.25	937.5	0	1836.5	162	45	0	0	3520.5		-0.31
743	EVTEETEETEETVGH	27614.5	0	0	18129	59	123	2202	337.5	60.5	262.5	46139	0	2678.75	134.5	108	0	15.5	7265	0	0	12	83	296	46.5	710.5	0	1252.75	690	194.5	19.75	0	2394.25		0.37
744	VTEETEETEETVGHT	41795.5	0	0	6829.5	0	137.5	625	165.5	130	31	44797.5	28.5	597	0	24.5	0	0	8150.5	50.5	124.5	0	33.5	85.5	18	195.5	0	1135	0	142	0	0	966		0.38
745	TEETEETEETVGHTT	39561.5	0	1519.25	0	10	0	0	216.5	224	76	44613.5	0	2093.75	0	35	0	0	8156.5	0	25.5	0	25	50	0	134	0	1401.25	0	45	0	0	1198.5		0.14
746	EETEETEETVGHTTT	31615.75	0	1615.5	0	0	363	78	80.75	0	796	32860.5	0	5936.75	153	58.5	0	0	7180.75	0	0	0	28	48.5	19	110	0	684.75	0	0	39.5	0	319		0.19
747	ETEETEETVGHTTTV	39063	212.75	2112	0	0	23	160.25	18	50.5	183.5	21467.5	0	1997	41.5	0	0	0	7470.5	0	0	0	0	0	0	42	0	134.5	0	0	40.5	15	15.5		0.81
748	TEETEETVGHTTTVT	38082	0	10577.75	0	0	40.5	35.5	0	131.5	41.5	27773	0	1849.75	0	0	0	0	5294.5	0	0	0	0	0	0	14	0	407.5	0	0	0	0	91		1.08
749	EETEETVGHTTTVTI	32719.5	6216	41720.25	0	0	295.5	105.5	0	0	647	2798.75	0	4870.5	280	0	65	0	4580.25	0	0	0	11.5	26	9.5	13	0	0	51.75	0	25	0	0		4.13
750	ETEETVGHTTTVTIT	16642.5	0	24011.5	0	0	0	2.5	0	0	185	0	0	1126.75	54.5	0	3190.5	0	0	0	0	0	0	0	0	0	0	0	0	0	12.5	85	0		3.25
751	TEETVGHTTTVTITL	1577	0	32730.25	0	0	19.5	0	0	0	0	0	0	0	0	0	2915.75	0	0	0	0	18	0	0	0	0	0	0	0	16	0	0	0		1.49
752	EETVGHTTTVTITLP	0	0	56962.25	0	0	171.5	0	0	0	179	0	0	3295	231	0	2212.5	0	0	0	0	0	0	8.5	0	0	0	0	0	0	0	0	0		1.32
753	ETVGHTTTVTITLPP	192.5	0	41161.5	0	0	0	0	0	0	0	0	0	0	110	0	973	0	0	0	0	29.5	0	0	0	0	0	0	0	0	0	0	0		1.38
754	TVGHTTTVTITLPPT	0	0	35232	0	0	0	0	0	0	0	0	0	0	0	0	120	0	0	0	0	0	0	0	0	0	0	0	0	0	0	0	0		1.37
755	VGHTTTVTITLPPTQ	1785.5	4676.5	26080.5	0	0	0	0	0	0	0	0	0	0	0	0	0	0	0	0	0	0	0	0	0	0	56.5	0	0	0	0	0	0		2.37
756	GHTTTVTITLPPTQP	11886.75	9074.25	42315.25	0	0	0	0	0	0	0	0	0	0	0	0	0	0	0	0	0	0	0	0	0	0	61.5	0	0	0	0	0	0		3.55
757	HTTTVTITLPPTQPS	8642.5	13179.25	34305	0	0	0	0	0	0	0	0	0	0	0	0	0	0	0	4	0	0	0	0	0	0	72	0	0	0	0	1104.75	0		4.16
758	TTTVTITLPPTQPSP	6414	12875.75	0	0	0	0	0	0	0	0	0	0	0	0	0	0	0	0	0	0	0	0	0	0	0	0	0	0	0	0	1907.25	0		2.91
759	TTVTITLPPTQPSPP	14732.75	15218	0	0	0	0	0	0	0	0	0	0	0	0	0	0	0	0	0	0	0	0	0	0	0	0	0	0	0	0	2352	0		3.45
760	TVTITLPPTQPSPPK	9205.5	6784	558	0	0	0	0	0	451.75	0	0	561.25	0	0	0	0	0	230.5	0	0	2711.75	0	0	2976.75	0	0	0	0	0	480.5	0	240.5		2.87
761	VTITLPPTQPSPPKE	6691.25	7180.25	590.5	0	0	0	0	0	947.25	0	0	281	0	455.25	0	0	0	44.5	0	0	2571	0	0	1284.25	0	0	0	0	0	175.5	1842.75	28.5		2.99
762	TITLPPTQPSPPKEV	8280.5	9349.75	803.75	0	1.5	0	0	0	0	0	0	280	0	321	0	0	0	117.5	0	0	2506.25	546.5	0	1005	0	0	0	0	0	239.5	1226.5	0		3.45
763	ITLPPTQPSPPKEVK	3662	3980.25	730	0	0	0	0	0	915.25	0	0	341.5	0	0	0	117.5	3.5	137.5	0	0	4229	0	0	780	0	0	0	0	0	382.5	769	41.5		1.71
764	TLPPTQPSPPKEVKV	1306.5	0	663.25	0	0	0	0	0	1015.5	0	0	113	0	466.5	0	112.5	0	301.5	0	0	4417	0	0	1745.5	0	0	149	0	0	201.5	1069.75	117.75		-0.51
765	LPPTQPSPPKEVKVV	3068.75	0	2174	0	0	0	0	0	1422.25	0	0	51	43	464.5	0	180.5	0	1112	0	0	0	0	0	1338.5	0	0	405	0	0	109.25	406	0		1.65
766	PPTQPSPPKEVKVVE	6425.5	0	2403.25	0	5	0	0	0	1171.5	0	0	0	0	674	0	60.5	0	1397	0	0	867.25	0	0	140.25	0	0	238	6.5	0	0	150.5	107		2.08
767	PTQPSPPKEVKVVEN	1641.5	0	963.5	2814	1	0	0	0	266.25	0	0	0	0	0	0	0	0	1225.5	0	0	195.5	0	0	0	0	0	0	0	0	0	0	0		4.00
768	TQPSPPKEVKVVENS	1568.75	0	2895.75	3463.5	0	0	0	0	294.5	0	0	0	0	0	0	0	0	1060.75	0	0	0	0	0	0	0	0	0	0	0	0	0	0		5.40
769	QPSPPKEVKVVENSI	572.5	0	2914.5	5411.25	0	0	0	0	0	0	0	0	0	0	0	0	0	1046.5	0	0	0	0	0	0	0	0	0	0	0	38	0	0		3.53
770	PSPPKEVKVVENSIE	2837.5	943.5	0	1926.75	10	0	0	0	0	0	0	0	0	0	0	28	0	678.5	0	0	0	0	0	0	0	0	0	0	0	118	0	0		4.93
771	SPPKEVKVVENSIEH	3057.25	1321	317.5	2783.25	0	0	0	0	0	0	0	0	0	0	0	96.5	0	219	0	0	0	0	0	0	0	0	0	0	0	0	0	0		7.01
772	PPKEVKVVENSIEHK	1283.25	0	129	0	0	0	0	0	0	0	0	0	0	0	0	0	0	0	0	0	211	0	0	0	0	0	0	0	0	0	0	0		1.61
773	PKEVKVVENSIEHKS	238	0	0	0	0	0	0	0	0	0	0	0	0	0	0	0	0	0	0	0	406	0	0	0	0	0	0	0	0	0	0	0		-0.15
774	KEVKVVENSIEHKSN	0	0	0	0	0	0	0	0	920	0	0	0	0	0	0	0	0	0	0	0	328	112.5	0	0	0	298.5	0	0	0	0	0	0		-0.28
775	EVKVVENSIEHKSND	0	0	109.5	0	0	0	6	0	0	28	0	0	166.5	42	10	0	0	0	0	0	179.5	0	0	0	0	0	0	73.5	0	0	0	0		-0.48
776	VKVVENSIEHKSNDN	422.5	0	1132.5	1407.5	0	0	0	0	312.5	0	0	0	53.5	0	0	0	0	0	0	0	44	0	0	0	0	0	0	0	102	0	0	0		4.86
777	KVVENSIEHKSNDNS	0	1024.5	190.5	1042.5	0	0	8	0	162.5	0	0	0	0	0	0	0	0	0	47	0	0	9.5	0	0	0	148.5	0	0	0	0	0	0		4.29
778	VVENSIEHKSNDNSQ	0	482.5	0	1057.75	0	0	54	0	31	0	0	0	25	0	0	0	0	0	0	0	0	0	0	0	0	0	0	12	0	0	0	0		2.92
779	VENSIEHKSNDNSQA	0	270	0	4317.75	0	1822.25	0	0	0	0	0	0	205	0	0	0	0	0	0	0	0	0	0	0	0	0	0	0	0	0	0	0		1.58
780	ENSIEHKSNDNSQAL	0	766.75	0	6771.5	0	0	0	0	0	104.5	25.25	0	206	109	2	0	0	0	0	0	18	0	0	0	0	0	0	0	0	46.5	0	0		1.76
781	NSIEHKSNDNSQALT	0	564	0	0	0	0	0	0	0	0	0	53	0	0	0	0	0	0	0	0	0	0	0	0	0	0	15	0	0	0	0	0		1.30
782	SIEHKSNDNSQALTK	0	0	0	0	0	0	0	0	0	0	15	0	0	0	0	0	0	0	0	0	0	0	0	0	0	0	0	0	0	0	0	0		-0.41
783	IEHKSNDNSQALTKT	0	0	20.5	0	0	0	0	0	0	0	0	0	111.5	0	0	0	0	0	0	0	0	0	0	0	0	0	0	0	0	0	0	0		-0.56
784	EHKSNDNSQALTKTV	0	148	265	628.25	0	0	0	0	0	114	0	0	347.5	98.75	13	0	0	0	0	0	0	0	58.5	0	0	0	0	0	0	0	46	0		2.45
785	HKSNDNSQALTKTVY	0	0	0	2390.5	0	0	0	0	0	0	0	0	0	0	0	4.5	0	0	0	0	0	0	0	0	0	0	0	0	0	0	0	0		1.37
786	KSNDNSQALTKTVYL	0	294.5	0	446	0	0	0	0	0	0	0	0	0	0	0	0	0	0	0	0	0	0	0	0	0	1319	0	0	0	0	0	0		-0.14
787	SNDNSQALTKTVYLK	0	220	0	0	0	0	0	0	0	0	0	0	0	0	0	0	0	0	0	0	0	0	0	0	0	0	0	0	0	17.5	0	0		1.33
788	NDNSQALTKTVYLKK	0	0	0	742	0	0	0	20	0	0	0	361	0	0	0	337	0	40	0	106	59.5	0	0	33	0	65	0	0	0	456	0	0		0.13
789	DNSQALTKTVYLKKL	0	0	0	0	0	0	0	12.5	0	0	0	385.5	0	0	0	564.75	229.5	24.5	0	65.5	0	0	0	0	0	0	0	0	0	355.5	0	0		-0.17
790	NSQALTKTVYLKKLD	0	0	0	0	0	0	0	0	0	0	0	29.5	0	0	0	0	0	0	0	0	17	0	0	0	0	85	0	0	0	0	0	0		-0.32
791	SQALTKTVYLKKLDE	0	0	0	0	0	0	0	0	0	0	0	0	0	0	0	0	0	0	0	0	0	0	0	0	0	0	0	48	0	0	0	0		-0.38
792	QALTKTVYLKKLDEF	0	0	37.5	0	0	0	0	0	36	2.75	0	0	0	0	441	0	0	1.5	0	91.5	0	0	0	0	0	0	0	0	0	0	0	0		-0.45

793 ALTKTVYLKKLDEFL	0	0	0	0	0	0	0	0	0	0	0	0	0	0	0	0	127	0	0	138.5	0	0	0	0	0	0	0	0	0	0	0	0		-0.33
794 LTKTVYLKKLDEFLT	0	0	0	0	0	0	0	0	0	0	0	0	0	0	0	0	0	0	0	98.5	0	0	0	0	0	0	0	0	0	0	0	0		-0.41
795 TKTVYLKKLDEFLTK	0	0	0	0	0	0	0	0	0	0	0	26.5	0	0	0	3	971	0	0	126.5	0	0	0	0	0	117	0	0	0	70.5	0	0		-0.34
796 KTVYLKKLDEFLTKS	0	172.5	0	0	0	0	0	0	0	0	0	0	0	0	0	29	1170.5	0	0	196.5	0	0	0	0	0	2037.5	0	0	0	73	0	0		-0.40
797 TVYLKKLDEFLTKSY	0	0	0	0	0	0	0	0	0	0	0	0	0	0	0	0	0	0	0	0	0	0	0	0	0	0	0	0	0	0	27	0		-0.41
798 VYLKKLDEFLTKSYI	0	0	0	0	0	0	0	0	0	0	0	0	0	0	0	0	225.75	0	0	0	0	0	0	0	0	0	0	0	0	17	0	0		-0.40
799 YLKKLDEFLTKSYIC	0	0	0	0	0	5	0	0	0	0	0	0	0	0	0	0	0	0	0	0	0	0	0	0	0	0	0	0	0	0	0	0	9	
800 LKKLDEFLTKSYICH	0	0	0	0	0	0	0	0	0	0	0	0	0	0	0	0	0	0	0	0	0	0	0	0	0	67.5	0	0	0	0	0	0		-0.41
801 KKLDEFLTKSYICHK	0	0	0	0	0	0	0	0	0	0	0	0	0	0	0	0	0	0	0	0	0	0	0	0	0	1841.5	0	0	0	0	0	0		-0.41
802 KLDEFLTKSYICHKY	0	0	0	0	0	0	0	0	0	0	0	0	0	0	0	0	0	0	0	0	0	0	0	0	0	743.5	0	0	0	0	0	0		-0.41
803 LDEFLTKSYICHKYI	0	0	0	0	0	0	0	0	0	0	0	0	0	0	0	0	0	0	0	0	0	0	0	0	0	0	0	0	0	0	0	0		
804 DEFLTKSYICHKYIL	0	0	0	0	0	0	0	0	0	0	0	0	0	0	0	0	0	0	0	0	0	0	0	0	0	0	0	0	0	0	0	0		
805 EFLTKSYICHKYILV	0	0	0	115.5	0	0	0	0	0	0	0	0	0	0	0	0	0	0	0	0	0	0	0	0	0	0	0	0	0	0	0	0		1.37
806 FLTKSYICHKYILVS	0	0	0	0	0	0	0	0	0	0	0	0	0	0	0	0	0	0	0	0	0	0	0	0	0	0	0	0	0	0	0	0		
807 LTKSYICHKYILVSN	0	0	0	0	0	0	0	0	0	0	0	0	0	0	0	0	0	0	0	0	0	0	0	0	0	0	0	0	0	0	0	0		
808 TKSYICHKYILVSNS	0	0	0	0	0	0	0	0	0	0	0	0	0	0	0	0	0	0	0	0	0	0	0	0	0	12	0	0	0	0	0	0		-0.41
809 KSYICHKYILVSNSS	0	0	0	0	0	0	0	0	0	0	0	0	0	236	0	0	104	0	2576.25	0	0	0	0	0	0	1294	0	0	0	0	0	0		-0.32
810 SYICHKYILVSNSSM	0	0	0	733	0	0	0	0	0	0	0	0	0	0	0	0	0	0	1672.5	0	0	0	0	0	0	0	0	0	0	0	0	0		-0.31
811 YICHKYILVSNSSMD	0	0	0	1048.5	0	0	33	7	0	0	0	0	0	0	0	0	0	0	2330.25	0	13	0	0	0	0	0	0	0	0	0	0	0		-0.30
812 ICHKYILVSNSSMDQ	0	0	0	0	0	0	0	0	0	0	0	0	0	0	0	0	0	527.75	1126.5	0	0	0	0	0	0	0	0	0	0	0	0	0		-0.34
813 CHKYILVSNSSMDQK	0	116.5	0	842.5	0	0	0	0	0	0	0	0	0	0	0	0	0	198.5	186.75	0	0	0	0	0	0	0	0	0	0	0	0	0		1.47
814 HKYILVSNSSMDQKL	0	0	0	685.75	0	0	0	0	0	0	0	0	0	0	0	0	0	0	513.5	159	0	0	0	0	0	0	0	0	0	0	0	0		0.44
815 KYILVSNSSMDQKLL	0	0	0	0	0	0	0	0	0	0	0	0	0	0	0	0	0	0	24.5	0	0	0	0	0	0	936	0	0	0	0	0	0		-0.41
816 YILVSNSSMDQKLLE	0	0	0	452.75	11.5	0	0	0	0	0	0	0	0	0	0	0	0	0	175	0	0	0	0	0	0	0	0	0	0	0	0	0		0.99
817 ILVSNSSMDQKLLEV	11	0	0	874.5	30	0	158	0	0	0	308.5	179.5	992.5	0	0	0	0	0	566	0	0	0	59	0	0	83	0	0	22	0	0	0		-0.14
818 LVSNSSMDQKLLEVY	0	0	0	1402.75	75	0	69.5	0	40.5	0	103.5	36.5	0	0	0	0	52.5	0	689	89	0	0	0	0	0	54.5	0	0	0	0	0	0		0.75
819 VSNSSMDQKLLEVYN	0	0	0	3693	0	0	153	0	0	0	830.75	0	0	0	0	0	11.25	0	1094.5	140.5	0	0	0	0	0	71.5	75.5	0	4.5	0	0	0		0.92
820 SNSSMDQKLLEVYNL	0	0	0	7195.5	340.5	0	0	2067	0	0	35407.5	0	0	0	0	0	0	0	5330.75	191.75	0	17	0	0	0	798.5	0	0	494.5	0	0	0		-0.57
821 NSSMDQKLLEVYNLT	0	0	0	1857	0	0	0	179.5	0	0	297.5	13	0	0	0	0	0	0	253	0	0	0	0	10.5	0	74.5	0	0	38	0	0	0		1.14
822 SSMDQKLLEVYNLTP	0	0	0	0	0	0	0	0	0	0	0	0	0	0	0	0	0	0	0	0	0	0	0	0	0	0	0	0	0	0	0	0		
823 SMDQKLLEVYNLTPE	0	0	0	0	0	5	0	0	250	0	0	0	0	0	0	0	0	0	0	42	0	0	0	0	0	0	0	44	0	0	0	106		-0.28
824 MDQKLLEVYNLTPEE	2381	35490	0	0	1.5	34	0	0	4171.75	125.5	346	35.5	4328	132.5	67	182.5	483.5	0	201.5	331	0	47.75	98.5	46	136.5	173.75	514.5	194.5	48	337	4674.5	2951.5		1.28
825 DQKLLEVYNLTPEEE	7542	43943.5	274	418	37.5	68	43	13	4510.75	185.5	322	57	5441.5	446	124.5	396	449.75	230	462.5	558	196.5	49.25	327	277	243.5	183.5	574.5	380.5	69	495.5	6128	2767.5		1.71
826 QKLLEVYNLTPEEEN	7969.25	58534	0	15897	28.5	63	13.5	0	3183.25	80	155	48.5	5092.25	179.5	68.5	388.5	466	303	525	862	0	0	173.75	145.5	97.5	106.5	273	187	25	564.5	2953	2538		2.87
827 KLLEVYNLTPEEENE	10202.5	55302.5	596.5	26575	39	68	21	0	2524.75	160	211	67.75	4724.25	213.5	66.5	321.5	380	252.5	518	603	235.25	15.5	316.5	194	135.5	95	248.5	346	79.5	592.5	3730.75	1831.5		3.96
828 LLEVYNLTPEEENEL	11077.25	58534	1078.5	21884	64.5	132	58	0	3665	232	599.5	128.5	7842.75	740	139	1014	730	512.5	671.5	1121.5	499	56	438.25	419.5	259.5	236.5	621.5	306.75	109.5	967	5222.25	3497.75		3.51
829 LEVYNLTPEEENELK	6341.75	58534	209.5	14539	31.5	29	9.5	0	3307.25	82	501.5	145.5	5224	157	66	1491.75	7890.75	1612.25	648.75	825.5	312.5	0	178.5	85	158	201.75	777.5	89	34.5	1666.25	8023.75	2685.25		2.41
830 EVYNLTPEEENELKS	9550.75	58534	0	18271	93	9	16.5	0	3087.5	107.25	210.5	70	4027.25	340.75	44	921.5	4797	713.5	491.25	697.25	662	352.5	173.5	106.5	92	138	517.5	55.5	16.5	999	5003	2209.5		3.08
831 VYNLTPEEENELKSC	4109.5	52681	187.5	18861.5	8	16.5	0	0	890.5	0	23	36	1881	0	14	42.25	3476.25	712.5	267	653.75	492	0	0	0	44.5	0	0	96.5	25	541.75	0	630.25		3.01
832 YNLTPEEENELKSCD	5928.25	53949.75	44.5	16292	46	32	93	0	2229.75	83.5	240.5	61	5353	35.5	85.5	0	3286.5	528.25	199.5	584.5	451	157	0	38.5	107	86.5	295.5	313	56.5	550.75	3028.25	1768		2.82
833 NLTPEEENELKSCDP	2902.75	39084	0	14207.5	29.5	35	0	0	0	33.5	0	110	222	0	13	0	2692.25	350.5	122	294.5	0	70	0	0	43.5	0	122.5	240.5	25	143.5	447.5	133		3.02
834 LTPEEENELKSCDPL	0	5170	0	11040.5	34	25	67	0	0	0	0	12	111.5	0	15.5	0	3059.5	55.5	55.5	199.25	0	109	0	0	12.5	0	0	129	23.5	47	477.5	62		2.55
835 TPEEENELKSCDPLD	0	0	0	13578.75	66.5	65.5	35	0	38.5	37	48.25	16.5	123.5	0	44.5	32.5	1336.25	119	0	104.5	0	64	0	0	23	0	60	308	76	10	118.25	57		1.27
836 PEEENELKSCDPLDL	0	0	0	13853.5	37	44.5	262.5	44	33.5	45.5	108.5	40	660.5	35	62	0	0	0	21	180.5	0	172	91	50	22.5	0	26	199	55.5	23.5	0	48		1.31
837 EEENELKSCDPLDLL	0	0	0	12943.75	47.5	168.5	28	3.5	0	189	15	11.5	3051	166.5	50.5	38	0	0	31	128.5	0	0	0	0	0	0	0	172	39.5	42	0	25.75		1.15
838 EENELKSCDPLDLLF	0	4780.25	0	14998.5	38.5	107.5	28.5	4.5	79.5	131.5	0	0	3669	260.5	47	83	5158.25	69.5	57.5	407.5	28.5	0	0	0	0	7.5	0	129	37	39.5	37	65.5		1.79
839 ENELKSCDPLDLLFN	0	4070	0	4798.75	26.5	10	0	0	0	58.5	0	0	210.5	42.5	24.75	17.5	128.25	0	0	84.5	0	0	0	2	0	0	5.5	43	10.5	359.5	0	24.5		3.42
840 NELKSCDPLDLLFNI	0	0	0	0	0	0	0	0	0	0	0	32	0	0	15.5	13.5	0	0	0	39	0	0	0	0	0	0	26.25	0	0	0	0	0		-0.14
841 ELKSCDPLDLLFNIQ	0	425.75	0	0	14	0	0	0	0	46.25	0	0	47.5	209	57	0	0	0	23.5	0	0	0	93.5	0	0	0	0	0	0	0	0	0		0.58
842 LKSCDPLDLLFNIQN	0	351	0	0	0	0	0	0	0	0	0	0	0	0	0	0	0	0	0	0	0	0	0	0	0	0	0	0	0	0	18.5	0		1.34
843 KSCDPLDLLFNIQNN	0	0	0	0	0	0	0	0	0	0	0	0	0	0	0	0	0	0	0	0	0	0	0	0	0	0	60	0	0	0	0	0		-0.41
844 SCDPLDLLFNIQNNI	0	0	0	393.5	0	0	9.5	0	0	0	0	30.5	0	0	0	0	0	0	31.5	23.5	0	0	0	0	0	0	52	0	0	0	0	0		1.17
845 CDPLDLLFNIQNNIP	0	0	0	0	0	0	0	0	0	0	0	0	0	0	0	0	0	5.5	15.5	192	0	0	0	0	0	0	47.5	0	0	0	0	0		-0.35
846 DPLDLLFNIQNNIPA	0	0	0	0	0	0	0	0	0	0	0	0	0	0	0	0	0	0	9.5	0	0	0	0	0	0	0	88	0	0	0	0	0		-0.39
847 PLDLLFNIQNNIPAM	0	0	0	0	0	0	0	0	0	0	2	0	0	0	0	0	0	0	0	0	0	0	0	0	0	0	15	17.5	0	0	6	0		-0.26
848 LDLLFNIQNNIPAMY	0	0	0	0	0	0	0	0	0	0	0	0	0	0	0	5	0	0	35.5	0	0	0	0	0	0	0	0	0	0	0	4	0		-0.37
849 DLLFNIQNNIPAMYS	0	0	0	1213.5	0	0	0	0	0	0	0	0	0	0	0	0	0	0	7.5	122	0	0	0	0	0	0	0	0	0	0	0	0		1.31
850 LLFNIQNNIPAMYSL	0	0	0	0	0	0	0	0	0	0	0	0	0	0	0	0	0	0	0	0	0	0	0	0	0	0	0	0	0	0	27.5	0		-0.41
851 LFNIQNNIPAMYSLY	0	0	25.5	0	0	0	0	0	0	7	0	0	0	0	0	0	120.5	44	72	93	0	0	0	0	0	0	0	0	0	0	2.5	0		-0.39
852 FNIQNNIPAMYSLYD	0	59	221.5	1248.5	45	2.25	10.5	0	130	0	0	0	118.5	53	120	0	332.5	94.5	57	211	0	0	35.5	0	20	0	28.5	129.75	0	0	32	0		1.47
853 NIQNNIPAMYSLYDS	0	20.5	0	749.5	0	0	0	0	49.5	0	0	37	8	0	0	19.5	0	93.75	35	89	16.5	0	0	0	0	0	30	34	0	0	9	0		1.16
854 IQNNIPAMYSLYDSM	0	24	0	0	0	7	0	0	30	0	0	0	0	0	0	64	223.5	67.5	57.5	74.25	0	0	0	0	0	0	0	4.5	0	0	0	0		-0.31
855 QNNIPAMYSLYDSMN	0	0	0	0	0	0	0	0	0	0	0	0	110	0	0	0	96.5	0	0	56.5	37	0	0	0	0	0	0	0	0	0	240.75	147		-0.13
856 NNIPAMYSLYDSMNN	0	0	0	0	0	0	0	7	0	0	0	121	29.5	0	0	0	0	0	0	0	0	0	0	0	0	0	133.75	0	0	0	31.25	130.25		-0.18
857 NIPAMYSLYDSMNND	0	1656	0	362	0	0	3	0	33	0	0	38.5	73	0	4	0	56.25	0	0	0	0	0	15	0	0	0	48.5	49	0	0	44	137		2.01
858 IPAMYSLYDSMNNDL	0	194.25	0	674.5	0	10	0	0	0	11	0	8	192.5	49	0	0	57.5	0	0	38	0	0	0	0	0	0	27	60.25	0	0	92.5	177		1.56
859 PAMYSLYDSMNNDLQ	0	0	0	519.25	0	0	0	2.25	0	0	0	0	216.5	0	0	0	0	0	0	12.75	0	0	0	0	0	0	0	0	0	0	23.25	118		0.79
860 AMYSLYDSMNNDLQH	0	0	0	552.5	0	0	0	0	0	0	0	0	0	0	0	0	0	0	0	0	0	0	0	0	0	0	0	0	0	0	0	75		1.28
861 MYSLYDSMNNDLQHL	0	0	0	0	0	0	0	0	0	0	0	0	14.5	51.5	0	0	0	0	0	0	0	0	0	0	0	0	0	0	0	0	12.5	52		-0.25
862 YSLYDSMNNDLQHLF	0	0	0	330	0	0	0	0	0	0	0	0	0	0	0	0	0	0	14.5	0	17	0	0	0	0	0	0	0	0	0	0	0		1.32
863 SLYDSMNNDLQHLFF	0	0	62	0	0	0	21	0	0	0	0	0	28.5	0	0	0	0	0	24	0	28.5	0	0	2.5	0	0	0	0	0	0	0	62		-0.09
864 LYDSMNNDLQHLFFE	769.5	0	446.5	1462	25.5	28.5	45.5	2	99	19.25	54.5	34.5	28.25	127	0	3029.75	81.5	222.5	82	210.25	41	28	0	0	0	0	30	135.5	32.5	15.5	27	62.5		0.25
865 YDSMNNDLQHLFFEL	304.75	0	260.25	2275	23.5	20.75	46.5	0	0	21	174.75	0	45.5	167	6.75	95	74	89	77.5	0	14.5	33	7.5	0	0	0	23.5	80	26	24.5	34	102.5		2.10
866 DSMNNDLQHLFFELY	86	0	327	960	22.5	27.5	40	0	27.5	0	91	0	0	38	0	200	114.5	94.5	73.5	137.5	35	21.5	47	0	8.5	0	35.5	76.5	14.5	8	0	27.5		2.27
867 SMNNDLQHLFFELYQ	0	0	0	0	0	0	0	0	0	0	0	0	0	4	0	0	0	0	0	114.25	0	0	0	0	0	0	9.5	21.5	0	0	0	0		-0.36
868 MNNDLQHLFFELYQK	0	0	0	1289.5	0	0	0	0	0	0	0	0	0	0	0	0	0	0	0	0	0	0	0	0	0	0	0	0	0	0	0	0		1.37
869 NNDLQHLFFELYQKE	461	0	0	0	10.5	0	1.5	0	54	0	0	75	0	0	0	41	345.75	458.25	0	877	11	41	17	0	0	0	62.5	93	0	0	0	0		-0.47
870 NDLQHLFFELYQKEM	319.5	0	0	39	18	0	0	0	0	0	0	43	0	0	0	0	94	179	3	425	0	615.5	17.5	0	0	0	17	35	0	0	5.5	0		-0.43
871 DLQHLFFELYQKEMI	394	0	15.5	0	18.5	16.5	4	0	0	0	0	0	0	0	0	180	359.75	492	0	809	0	91	0	0	0	0	0	24.5	0	7.5	9.75	26.5		-0.51
872 LQHLFFELYQKEMIY	117	0	182.5	340	0	0	4	0	0	0	0	0	0	0	0	0	332	191.5	0	1398	50	58.5	13	0	11	0	0	0	0	9	0	0		-0.37
873 QHLFFELYQKEMIYY	47.5	16.5	254.25	851.5	20	14.5	0	0	26.75	0	0	0	0	0	0	74	371.25	190.5	26	951.75	74	43	0	0	7	0	0	32.25	0	13.5	9	18		0.44
874 HLFFELYQKEMIYYL	0	0	98.5	418.5	21.5	0	0	0	0	0	0	0	0	0	0	0	240	136	0	1301.5	37	51.5	0	13.5	0	0	0	25.5	0	3	0	0		-0.42
875 LFFELYQKEMIYYLH	0	0	0	0	24.75	12.5	6.5	0	24	0	0	0	0	0	0	0	0	0	0	522.5	26	0	0	0	8	0	0	14.5	0	5.5	7	0		-0.38
876 FFELYQKEMIYYLHK	0	0	0	0	0	0	0	0	0	0	0	0	0	0	0	0	0	0	0	0	0	0	0	0	0	0	0	0	0	0	0	0		
877 FELYQKEMIYYLHKL	209.75	0	0	0	0	7.5	0	0	0	0	0	0	0	0	0	0	0	0	0	0	0	0	0	0	0	0	0	0	0	0	0	0		1.37
878 ELYQKEMIYYLHKLK	0	0	0	0	25.5	0	0	0	0	0	0	0	0	0	0	0	0	0	0	0	0	0	0	0	0	0	0	0	0	0	0	0		
879 LYQKEMIYYLHKLKE	0	0	111	0	0	2.25	0	0	0	0	0	0	0	0	0	0	0	0	0	0	0	23	0	0	0	0	0	0	0	0	0	0		1.21
880 YQKEMIYYLHKLKEE	0	0	195.5	0	11.5	10.5	0	0	0	0	0	0	173	0	12.75	0	0	0	0	12.5	0	157.5	9.5	10	1.5	0	6	47.5	0	0	0	0		-0.06
881 QKEMIYYLHKLKEEN	0	0	0	3570	0	0	0	0	0	0	0	0	0	0	0	0	0	0	0	0	0	73.5	0	0	0	0	0	0	0	0	0	11.25		1.36
882 KEMIYYLHKLKEENH	0	0	0	2953	0	0	0	0	0	28	0	0	0	0	0	0	0	0	0	29	0	449	0	0	0	334.5	0	0	0	0	0	0		1.19
883 EMIYYLHKLKEENHI	0	0	0	1193.5	10	0	0	0	0	314	0	0	302.25	0	25.5	0	0	0	0	0	0	0	55.5	0	0	0	0	0	0	0	0	0		0.94
884 MIYYLHKLKEENHIK	0	0	0	0	0	0	0	12	0	0	0	4.5	0	0	0	0	0	0	0	0	0	0	0	0	0	0	0	0	0	0	0	0		-0.41
885 IYYLHKLKEENHIKK	0	0	0	0	0	0	0	112.5	0	0	0	0	0	0	0	0	0	0	0	0	0	0	0	0	0	0	0	0	0	0	0	0		
886 YYLHKLKEENHIKKL	0	0	0	0	0	0	0	49	0	0	0	0	0	0	0	0	0	0	0	61	0	0	0	0	0	0	0	0	0	0	0	0		-0.41
887 YLHKLKEENHIKKLL	0	0	0	0	0	0	0	10.5	0	0	0	0	0	0	0	191.5	0	0	0	0	0	0	0	0	0	0	0	0	0	0	0	0		-0.41
888 LHKLKEENHIKKLLE	0	0	0	0	0	0	0	17.5	0	0	0	0	0	0	0	55.75	124.5	0	0	53.5	0	0	0	0	0	0	0	0	0	0	0	0		-0.28
889 HKLKEENHIKKLLEE	0	0	0	0	0	0	0	0	0	0	0	0	100	0	0	0	0	88.5	0	313	23	24.75	89.5	4	0	0	0	0	14	0	0	0		-0.21
890 KLKEENHIKKLLEEQ	0	0	0	0	1.5	17	0	0	1523	0	0	0	0	0	0	87.5	0	0	0	72	0	0	310	0	0	0	1155	0	0	0	0	0		-0.28
891 LKEENHIKKLLEEQK	0	0	0	0	0	0	0	0	896.25	0	0	0	0	0	0	49.5	176	0	0	0	0	0	0	0	0	0	500.5	0	0	0	0	0		-0.29
892 KEENHIKKLLEEQKQ	0	0	0	0	0	0	0	0	199	0	0	0	819	0	0	71	71	0	0	0	0	705	0	0	0	192.5	0	0	0	0	0	0		-0.22
893 EENHIKKLLEEQKQI	0	0	0	3788	0	42	0	0	1080.5	0	0	0	1489.75	34	0	92	254.25	206.5	0	0	0	0	0	0	0	0	349	0	0	0	0	0		0.71
894 ENHIKKLLEEQKQIT	0	0	0	0	0	0	0	0	534.5	0	0	0	0	0	0	118	130.5	0	0	0	0	0	0	0	0	0	219.5	0	0	0	0	0		-0.27
895 NHIKKLLEEQKQITG	0	0	0	0	0	0	0	0	0	0	0	0	0	0	0	28.5	0	202.25	0	0	0	0	0	0	0	0	0	0	0	0	0	0		-0.39
896 HIKKLLEEQKQITGT	28735.25	58534	0	0	0	0	0	0	1274.25	0	0	0	0	0	0	80.75	0	150	0	0	0	0	0	0	0	0	223.5	0	0	0	0	0		3.03
897 IKKLLEEQKQITGTS	19303.5	15268	0	0	0	0	0	0	1472	0	0	0	0	0	0	45	0	100.5	0	0	0	0	0	0	0	137.5	257.5	0	137.5	19.5	0	0		3.43
898 KKLLEEQKQITGTSS	27723.5	19777	0	0	0	0	0	0	2602	0	0	0	0	98.5	861.5	71.5	206	0	0	0	0	0	0	0	0	699.25	1012	0	1136.25	202	0	161.5		3.27
899 KLLEEQKQITGTSST	33649	29525.75	0	0	0	0	0	0	1194	0	0	0	0	0	0	50.5	0	327.5	0	0	0	0	330	0	0	140	481.75	0	0	0	0	0		3.52
900 LLEEQKQITGTSSTS	26236	23863	0	0	0	41	13.5	0	0	0	0	84.5	0	0	0	0	0	0	0	0	0	0	0	0	0	0	0	0	0	0	0	0		3.57
901 LEEQKQITGTSSTSS	31127	28225.5	834	0	0	0	0	0	0	0	0	0	0	0	0	0	0	59	0	0	0	0	0	0	17.5	0	0	0	0	0	0	0		3.72
902 EEQKQITGTSSTSSP	20227	14188.75	116.5	0	0	96.5	0	0	0	20	15.5	0	2961.25	102.5	0	0	0	0	0	0	0	0	0	0	0	0	0	0	0	0	0	0		3.31
903 EQKQITGTSSTSSPG	4987	3026.75	0	0	0	0	0	0	0	0	0	0	0	0	0	0	0	0	0	0	0	0	0	0	0	0	0	0	0	0	0	0		3.28
904 QKQITGTSSTSSPGN	0	1816.5	0	0	0	0	0	25.5	0	0	0	0	0	0	0	0	0	0	0	0	0	0	0	0	0	0	0	0	0	0	0	0		1.37
905 KQITGTSSTSSPGNT	190	1700.5	2262.75	0	0	0	0	0	0	0	0	0	0	0	0	258	0	0	0	0	0	0	0	0	0	201	0	0	0	0	0	0		3.80
906 QITGTSSTSSPGNTT	988.5	4699.5	2638.25	0	0	0	0	0	0	0	0	0	0	0	0	1652.75	0	0	0	0	0	0	0	0	22.25	0	0	0	0	0	0	0	10	3.73
907 ITGTSSTSSPGNTTV	160	4916.25	1537	1262	0	0	0	0	0	0	0	0	0	0	0	1394.75	0	0	0	0	0	0	0	0	0	0	0	0	0	0	0	0		3.60
908 TGTSSTSS PGNTTVN	0	3881.5	1341	232	0	0	512.5	440.5	0	0	22	0	3058.75	0	1186.25	2180	9919.5	5649	0	990.75	116	163.25	73	10.5	11.5	0	189.5	0	322.5	0	0	1929		-0.48
909 GTSSTSSPG NTTVNT	29693	5997	1229	0	0	0	539.75	322.5	0	0	0	0	3737	0	6763	2038.75	11386.5	6294	0	1115	0	447.5	324.5	0	13.5	0	66.75	0	1121.5	0	0	4159		1.25
910 TSSTSSPG NTTVNTA	33086.75	5575.25	237.5	0	0	0	500.5	309	0	0	134	0	4005.25	4049	5433.25	1476	9679.25	5244	0	0	122	577.5	364.5	0	0	0	0	0	1001.25	0	6.5	5158.25		1.21
911 SSTSSPG NTTVNTAQ	20664	4369.5	0	319.25	0	0	388.5	160.5	0	0	3384.5	0	2680.5	0	3694	1150.25	7401.5	4230.75	0	308	82.5	331.5	185	0	0	0	0	0	723	0	0	4683		1.14
912 STSSPG NTTVNTAQS	23989.75	15380.5	0	0	0	0	173	99	0	0	6472	0	1890	0	3657.5	864.5	7202	4040.75	0	252.25	71	182	102.5	0	0	0	41	0	550.5	0	0	4157.5		2.22
913 TSSPG NTTVNTAQSA	12760.5	3105.75	0	722.5	0	0	0	23	0	0	0	0	0	0	1899	566.5	6017	2847.25	0	0	23	12	0	0	0	0	0	0	328.5	0	0	1598.5		1.44
914 SSPG NTTVNTAQSAT	15183.25	3260.25	0	0	0	0	64	54	0	0	0	0	0	0	2090.5	736.25	6381	2601.75	0	0	0	78.5	35	0	0	0	0	0	404	0	0	2054.5		1.30
915 SPGNTTVNTAQSATH	7286.25	1663.5	46.5	0	0	0	15	39	0	0	0	0	0	0	1511.75	372.5	4927	1608.75	0	29.5	0	16	27	0	0	0	0	0	180	0	0	752		0.89
916 PGNTTVNTAQSATHS	12325	1878.5	0	0	0	0	0	20.5	0	0	0	0	0	0	1317	107	4520.5	1689.5	0	0	0	0	25	0	0	0	0	0	78.5	0	0	322.5		1.34
917 GNTTVNTAQSATHSN	8475	1516.75	0	0	0	0	0	0	0	0	0	0	0	0	288	34.5	2192	884	0	0	0	0	0	0	0	0	0	0	0	0	0	0		1.68
918 NTTVNTAQSATHSNS	3704.5	1397.5	0	0	0	0	0	0	0	0	0	0	0	0	162.5	93	4582.25	1230	10	204.75	0	0	0	0	0	0	0	0	35.5	0	0	0		0.46
919 TTVNTAQSATHSNSQ	1488.5	0	0	0	0	0	0	0	0	0	0	0	0	0	0	0	0	82.5	0	0	0	0	0	0	0	0	0	0	0	0	0	0		1.34
920 TVNTAQSATHSNSQN	0	0	2.75	2325.25	0	0	0	0	0	0	0	0	0	0	0	0	0	71.5	0	42	0	0	0	0	0	0	0	0	0	0	0	0		1.35
921 VNTAQSATHSNSQNQ	0	0	0	2817	0	0	0	0	0	0	0	0	0	0	0	0	0	0	30.5	0	0	0	0	0	0	0	0	0	0	0	0	0		1.37
922 NTAQSATHSNSQNQQ	1272	0	0	0	0	0	0	0	0	0	0	0	0	0	0	0	0	0	0	0	0	0	0	0	0	0	0	0	0	0	0	0		1.37
923 TAQSATHSNSQNQQS	22860.25	30032	0	216.75	0	0	0	0	0	0	0	0	0	0	0	0	0	0	0	0	0	0	0	0	0	0	24.5	0	0	0	0	0		3.53
924 AQSATHSNSQNQQSN	131.5	0	1359.25	0	0	0	0	0	0	0	0	0	0	0	0	0	0	0	0	0	381.25	0	0	0	0	0	42	0	0	0	0	239.25		1.31
925 QSATHSNSQNQQSNA	298.25	0	0	0	0	0	0	0	0	0	0	0	0	0	0	0	0	0	0	0	0	0	0	0	0	0	13	0	0	0	0	0		1.35

926 SATHSNSQNQQSNAS	0	0	0	3290	0	0	0	0	0	0	4.75	0	0	0	0	0	97.75	0	1941.25	0	0	0	0	0	0	0	0	0	0	0	0	0		0.71
927 ATHSNSQNQQSNASS	0	0	0	867.5	0	0	0	0	0	0	210	0	0	0	0	0	417.25	0	0	0	0	0	0	0	0	0	0	0	47.5	0	0	0		0.71
928 THSNSQNQQSNASST	0	0	0	2477.75	0	0	4.5	0	0	0	0	0	115.5	0	0	0	54.5	0	45.5	0	0	0	0	0	0	0	0	0	0	0	0	0		1.33
929 HSNSQNQQSNASSTN	0	0	1162.5	4742	0	0	0	0	1016.5	0	0	0	0	0	0	2652	0	0	0	0	0	0	0	0	0	0	0	93	0	0	0	0		1.27
930 SNSQNQQSNASSTNT	0	521.5	0	7870	0	0	20	91	2104	0	276	0	0	0	0	2731.75	58.5	43.75	241	3279.5	0	0	0	0	0	0	0	109.5	30.5	0	0	0		0.71
931 NSQNQQSNASSTNTQ	0	400	0	2062	0	0	0	0	2333.25	0	0	0	0	0	0	2139.25	0	0	0	4471.25	0	0	0	0	0	0	0	0	0	0	0	0		-0.40
932 SQNQQSNASSTNTQN	4564.5	0	1725	1033	0	0	977	124.5	1158.5	0	0	0	6955.5	0	874.5	2369.5	4535	2743	0	7426.75	168	6.25	442.5	0	0	0	0	0	157.25	0	0	1137.5		-0.33
933 QNQQSNASSTNTQNG	0	0	0	0	0	0	112.5	0	0	0	0	0	322	0	0	925.25	699.75	1278	0	3665.25	0	0	164.5	0	0	0	0	0	0	0	0	460		-0.22
934 NQQSNASSTNTQNGV	0	0	0	0	0	0	79.5	0	131	0	0	0	616.25	0	32.5	1035.75	532	355.5	0	2840.5	0	0	135	0	0	0	0	0	0	0	0	274		-0.21
935 QQSNASSTNTQNGVA	0	0	0	92	0	0	20	0	0	0	0	0	232	0	97.5	494.5	373.5	0	0	0	0	57	0	0	0	0	0	0	0	0	0	0		-0.39
936 QSNASSTNTQNGVAV	0	0	0	0	0	0	119.5	38.5	0	0	0	0	899	0	161.5	271	173	0	0	1121.5	0	155.5	35.5	0	0	0	0	0	0	0	0	483.5		-0.14
937 SNASSTNTQNGVAVS	0	0	0	0	0	0	124	77	0	0	0	0	1398	0	242.5	55.5	0	0	0	0	0	116.5	0	0	0	0	0	0	0	0	0	1671.25		-0.28
938 NASSTNTQNGVAVSS	0	0	0	5049.5	0	0	46.5	20	0	0	0	0	283.5	0	44.5	0	0	0	0	0	0	0	0	0	0	0	0	0	0	0	0	335.5		1.30
939 ASSTNTQNGVAVSSG	0	0	0	0	0	0	234	0	0	0	0	0	112	0	0	0	0	0	0	0	0	0	0	0	0	0	0	0	14	0	0	53.5		-0.32
940 SSTNTQNGVAVSSGP	0	0	0	0	0	0	212.25	0	0	0	206.5	0	232.75	0	55.5	0	0	0	0	0	0	20.5	0	0	0	0	0	0	0	0	0	306.75		-0.21
941 STNTQNGVAVSSGPA	0	0	0	0	0	0	17	0	0	0	0	0	27.5	0	0	0	0	0	0	0	0	0	0	0	0	0	0	0	0	0	0	0		-0.41
942 TNTQNGVAVSSGPAV	0	111	0	0	0	0	17.5	0	0	0	842	0	0	0	0	0	0	0	0	0	0	226	0	0	0	0	0	0	0	0	0	0		-0.50
943 NTQNGVAVSSGPAVV	0	113.5	0	0	0	0	0	0	0	0	459.5	0	0	0	0	0	0	0	0	0	0	36.5	0	0	0	0	0	0	0	0	0	0		-0.51
944 TQNGVAVSSGPAVVE	2015.5	280.5	0	0	0	0	226	0	0	0	2311	0	0	0	0	0	67.25	0	0	0	0	48	0	0	0	0	0	0	0	11.75	20.5	56.25		0.32
945 QNGVAVSSGPAVVEE	7899.25	234	69	4775	0	13.5	678	0	215.5	0	3009.75	0	119	2494.75	4	0	20.5	0	0	0	0	24.5	7.5	0	0	0	35.5	106	0	0	66.5	86		2.46
946 NGVAVSSGPAVVEES	10847.25	405	0	3668.5	0	10	74	0	28	0	2596.75	25.5	0	8283.25	0	0	0	0	0	0	0	0	0	26	0	0	16.75	17.5	0	0	66.5	5.5		1.20
947 GVAVSSGPAVVEESH	5395	347	0	3050.5	6.5	0	234	0	2552	0	1630.25	0	841.5	9199	0	0	0	3158	0	0	0	150	0	9.5	0	0	0	0	0	0	0	394.5		0.12
948 VAVSSGPAVVEESHD	2716.25	312	0	2193	0	0	437.75	0	3353	12	2220.5	0	1058	9918.5	0	0	0	1653	0	0	0	0	0	35.75	9.5	0	76.5	94	65	0	89	340		-0.38
949 AVSSGPAVVEESHDP	3144.25	232.5	0	1203.75	0	10	406.5	0	3771.25	0	2303.5	30.5	3609	11741	7.5	76	0	36136.75	28	0	0	0	0	43.5	26.5	504	20	79.5	116	784	577.5	1116		-0.43
950 VSSGPAVVEESHDPL	3324.75	87	0	663	23.5	0	7450.25	0	2710.75	0	1287.5	0	1402	11383.25	0	0	0	14470	0	0	0	15.5	0	24	0	39.5	0	38	401.5	357.5	177.5	450		-0.50
951 SSGPAVVEESHDPLT	4013	78.25	0	351.25	0	0	9.5	0	1802.5	0	591.5	0	1160	9114	0	0	0	15738.5	4	0	0	0	0	19.5	0	111.5	0	7.5	5.5	321.5	41.5	390.5		-0.55
952 SGPAVVEESHDPLTV	9509.75	4323	0	449.5	0	0	0	0	1619	0	0	0	796.5	10826	0	0	0	11069.5	0	0	0	0	0	15.5	0	36.5	0	0	0	138.5	0	224.5		0.22
953 GPAVVEESHDPLTVL	19697.25	13623	0	0	33	0	87.5	0	1374	0	0	0	355.5	11190.75	0	0	0	13030.5	12	0	0	117.5	0	0	0	41.5	0	0	0	47.5	0	157		1.53
954 PAVVEESHDPLTVLS	20834.5	15442	0	0	0	0	0	0	2396	0	172.5	0	726.5	11905.25	0	0	0	13062.5	0	0	0	0	0	0	0	238.5	0	0	57	164.5	0	305.75		1.62
955 AVVEESHDPLTVLSI	35222.75	18756	0	0	0	0	59.5	9	0	0	205.5	0	1333.75	0	0	20.5	80.5	19251.75	0	0	43.5	0	47	15.5	46	293.5	0	0	94.25	279	86.25	485		2.09
956 VVEESHDPLTVLSIS	31024.25	19852.5	0	0	0	33	481.75	0	0	0	46.25	0	535.5	22	8	0	0	6228.75	0	50.5	32.5	0	56.5	0	62	385	39.5	0	73.75	249.5	76.75	568		3.07
957 VEESHDPLTVLSISN	10959.75	15206.5	62.5	0	0	0	0	0	0	0	0	0	1152	100.75	0	0	0	11068	15	43.5	9.75	0	0	0	0	159.5	0	0	10	51.25	124	196.5		1.98
958 EESHDPLTVLSISND	4385.5	14101.5	203	948.5	0	236.5	0	0	0	2004.25	0	0	3615.25	173.5	48	0	0	4256	0	31.25	28.5	0	60	0	2	0	0	116	17.25	3.5	47.5	205.5		2.10
959 ESHDPLTVLSISNDL	1725	2374.75	122.5	0	0	5	0	0	0	582	0	0	168.75	193	760.75	0	0	0	0	0	0	0	0	0	0	0	0	0	0	0	0	70.5		2.91
960 SHDPLTVLSISNDLK	11828.75	9712.75	0	0	0	0	0	0	0	0	0	0	0	0	169.5	0	268.5	0	0	0	0	0	0	0	0	0	0	0	0	0	0	0		3.51
961 HDPLTVLSISNDLKG	594.75	0	0	0	27	0	0	0	0	0	0	0	0	0	0	0	16.5	0	0	0	0	0	0	0	0	0	0	0	0	0	0	0		1.36
962 DPLTVLSISNDLKGI	1534.75	0	0	0	0	0	0	0	0	0	0	0	0	0	0	81	57	0	0	0	0	0	0	0	0	0	0	0	0	0	3	0		1.32
963 PLTVLSISNDLKGIV	3527	390	0	0	0	0	0	0	0	0	0	0	0	0	0	0	0	0	0	0	0	0	0	0	0	0	0	59	0	0	0	0		1.78
964 LTVLSISNDLKGIVS	2850.75	341.5	0	0	0	0	0	0	0	0	0	0	0	0	0	0	0	0	0	0	0	0	0	0	0	0	0	0	0	0	0	0		1.82
965 TVLSISNDLKGIVSL	1851	0	0	0	0	0	0	0	0	0	0	0	0	0	0	0	7.5	0	0	0	0	5	0	0	0	0	5.75	0	0	0	0	187		1.30
966 VLSISNDLKGIVSLL	1080.5	0	0	0	0	0	0	0	0	0	0	0	0	0	0	0	67.5	0	29.25	15.5	0	7	0	0	0	0	0	0	23	0	0	0		1.30
967 LSISNDLKGIVSLLN	513	0	0	0	0	0	0	0	0	0	0	0	0	0	0	0	0	79.5	0	0	0	0	0	0	0	0	0	0	0	0	0	0		1.26
968 SISNDLKGIVSLLNL	294	0	0	0	0	0	0	0	0	0	0	0	0	0	0	0	0	0	0	0	0	0	0	0	0	0	0	0	0	0	0	0		1.37
969 ISNDLKGIVSLLNLG	0	0	0	1389.5	0	0	0	0	0	0	0	0	0	0	0	0	0	0	0	0	0	0	0	0	0	0	0	0	0	0	0	0		1.37
970 SNDLKGIVSLLNLGN	0	0	0	1309.5	0	0	0	0	0	0	0	0	0	0	0	0	0	0	0	68.5	0	0	0	0	0	0	0	0	0	0	0	0		1.34
971 NDLKGIVSLLNLGNK	0	0	0	260	0	0	0	0	0	0	0	0	0	0	0	0	0	0	0	0	0	0	0	0	0	0	0	0	0	0	0	0		1.37
972 DLKGIVSLLNLGNKT	0	0	0	296	0	0	0	0	0	0	0	0	0	0	0	0	0	0	0	0	0	0	0	0	0	0	0	0	0	9	0	0		1.36
973 LKGIVSLLNLGNKTK	0	0	0	268.5	0	0	0	114	0	0	0	0	0	0	0	0	0	0	0	1343.75	0	0	0	0	0	1089.5	0	0	0	0	0	0		-0.50
974 KGIVSLLNLGNKTKV	0	0	0	1905	0	0	0	23	0	0	0	0	0	0	0	0	636.5	0	17.5	439.5	0	0	0	11.5	0	1946	0	0	0	0	0	0		0.10
975 GIVSLLNLGNKTKVP	0	0	0	837.75	0	0	0	0	0	0	0	0	0	0	0	0	0	0	0	1208	48.75	0	0	0	0	275.75	0	43.5	0	61.75	0	0		-0.12
976 IVSLLNLGNKTKVPN	0	604.5	120.5	686.5	0	0	0	0	0	0	0	0	0	0	0	0	0	0	0	0	0	0	0	0	0	238.5	0	0	0	0	0	0		3.98
977 VSLLNLGNKTKVPNP	0	0	0	248.5	0	0	0	0	0	0	0	0	25490	0	0	0	0	0	0	0	0	0	0	0	0	340	0	0	13	0	99	0		-0.42
978 SLLNLGNKTKVPNPL	0	0	0	0	0	0	0	0	0	0	0	0	22170.25	0	0	0	0	0	0	0	0	0	0	0	0	483	0	0	0	65.5	56	0		-0.41
979 LLNLGNKTKVPNPLT	0	0	0	462.5	0	0	0	0	0	0	0	35	16051.25	68.5	0	130	226.5	0	0	0	0	0	3	104	0	511	0	0	0	675.5	945.5	230.5		-0.41
980 LNLGNKTKVPNPLTI	0	0	0	583.5	0	0	0	0	0	0	0	55.5	11250	92.25	0	87	145	0	0	0	27	0	0	264	0	365.5	0	0	0	749.25	224.5	137		-0.43
981 NLGNKTKVPNPLTIS	0	0	0	351.5	0	0	0	0	0	0	0	139	9005.25	0	0	64	0	0	0	0	42	0	0	70	0	5282.5	0	0	0	469.75	297.5	203.25		-0.35
982 LGNKTKVPNPLTIST	0	0	0	419	0	0	0	0	0	0	0	0	13456	53	0	13	0	0	0	0	0	1.5	0	154.5	0	222	0	0	0	482.5	319.5	192		-0.43
983 GNKTKVPNPLTISTT	0	0	0	0	0	0	0	0	0	0	0	0	13355.25	59	0	49	62	0	0	81	0	0	0	135.5	0	153	0	0	0	258.5	269.75	0		-0.40
984 NKTKVPNPLTISTTE	0	0	0	212.75	0	0	2.5	0	52	0	0	0	16895.25	73.5	0	93	136.25	0	0	328	0	219	15.5	239.25	0	3528.5	0	0	1021.75	1400.5	367.5	509.5		-0.34
985 KTKVPNPLTISTTEM	0	0	0	0	0	0	0	0	0	0	0	0	22775.75	185	0	131	250	0	0	0	26	57	0	219.5	0	1015	0	0	552	963.5	221	321.75		-0.38
986 TKVPNPLTISTTEME	738.5	0	142.5	0	0	0	0	0	43.5	41	5	0	233.5	31.5	0	154.5	176.75	82.5	0	0	41.5	206	13.5	0	0	253	0	47	1206.75	1509.5	448.5	709.5		-0.57
987 KVPNPLTISTTEMEK	199	59	0	0	0	0	0	0	0	10	0	0	407	0	0	111.5	316.75	710.5	445.5	0	48.5	64	0	173	0	1008.75	65	0	602	1636.5	0	0		-0.20
988 VPNPLTISTTEMEKF	246	616.25	0	0	0	0	0	0	178.5	0	0	0	0	0	0	0	121.5	540	558.5	84	0	0	0	0	0	0	28	0	803.5	0	0	14		-0.11
989 PNPLTISTTEMEKFY	173.25	3409.25	0	0	0	0	0	0	10.5	0	8	0	0	0	0	0	0	109.5	31	549	11	42	0	0	0	0	0	51	263	0	0	0		1.36
990 NPLTISTTEMEKFYE	228	63774	125.5	1413	22	7	25.75	35	53.5	26	28.5	104	69	98.5	13	45.5	285	278.5	62.5	1100.25	7.5	135	33.5	41.5	0	0	43.5	233.5	548.5	0	0	15.5		1.45
991 PLTISTTEMEKFYEN	211.75	31922.5	0	0	9.5	4	60	37.75	0	0	39	34.75	161.25	52.5	0	0	0	37.75	17	319	0	39.5	64	0	0	0	0	311.5	854.5	0	0	0		1.37
992 LTISTTEMEKFYENI	455.5	51507.75	0	1456.5	4.5	7	21	4	0	0	0	11.75	49	53	0	115	526.5	16	32	525	0	0	0	0	0	0	8	77.5	649.5	0	0	5.5		1.49
993 TISTTEMEKFYENIL	0	36073	0	439.5	0	4	38	11	0	0	0	0	22	0	0	0	0	0	0	37.25	0	0	147.25	0	0	0	0	22.5	365	0	2.5	0		1.41
994 ISTTEMEKFYENILK	0	17091.75	0	0	0	0	0	0	0	0	0	6	543.5	0	27	0	0	0	0	0	0	0	349.5	0	0	0	0	0	4425	0	0	0		1.13
995 STTEMEKFYENILKN	39	8887.5	0	0	0	0	0	0	0	0	0	0	0	0	0	0	0	0	0	0	0	0	0	0	0	0	0	0	48.5	0	0	0		1.39
996 TTEMEKFYENILKNN	0	1096.5	452	633.5	5	0	0	0	0	0	0	0	0	0	0	0	0	0	0	0	0	0	0	0	0	0	18.5	0	0	0	0	0		5.64
997 TEMEKFYENILKNND	43	0	138	0	21	0	0	0	0	50.5	0	0	1042.5	0	0	0	0	0	0	0	0	0	21.5	0	0	0	26	0	0	0	0	0		-0.57
998 EMEKFYENILKNNDT	0	0	0	0	0	0	0	0	6513	1626.75	0	0	2003.25	101.5	0	0	0	0	0	0	0	0	59	0	0	0	0	0	10	0	0	0		-0.31
999 MEKFYENILKNNDTY	202	2189.25	112.5	0	0	0	10	0	1706.5	12.75	0	0	0	0	0	0	0	2.5	0	0	0	0	0	0	0	0	4.5	0	0	0	0	0		0.79
1000 EKFYENILKNNDTYF	0	0	0	554.75	0	0	20	0	562.5	41	0	0	140	24	0	109.25	178.5	35	0	0	26.5	0	23.5	0	0	0	0	0	0	0	0	0		0.01
1001 KFYENILKNNDTYFN	0	378.5	0	0	0	0	0	0	715	0	0	0	0	0	25	206.75	0	0	0	0	0	0	0	0	0	142.5	0	0	0	0	63	0		-0.34
1002 FYENILKNNDTYFND	0	5223.25	401.5	2008.5	0	15	54.5	5.75	489.5	6	11	38	0	40	62	593.5	921.5	233.5	70	0	30	0	31	0	0	0	62	202.5	49	0	155	8.25		2.71
1003 YENILKNNDTYFNDD	0	3986	854	2363.5	63.5	48	80.5	25.5	441.5	43	0	46	87	136.5	33	685.5	597.75	348.5	193	76.75	0	46	82.5	0	0	0	87	454	129.75	130	350.5	28.5		3.72
1004 ENILKNNDTYFNDDI	0	2412	342.75	0	36.5	20.5	42.25	11	0	32	0	130	121	211.5	11	504.75	290.5	137	0	0	10.25	0	83	0	0	0	45	216	59.5	0	243.5	41		1.35
1005 NILKNNDTYFNDDIK	22213.75	4369	0	0	0	0	0	0	0	0	9	69.5	0	0	0	0	222.5	0	0	0	0	0	0	0	0	0	9.5	0	13.5	0	59.5	932.75		2.08
1006 ILKNNDTYFNDDIKQ	11601.75	1483.5	0	0	0	0	0	0	0	0	0	0	0	0	0	283.5	0	0	0	0	0	0	0	0	0	0	0	0	0	0	76	461.25		1.81
1007 LKNNDTYFNDDIKQF	9019.25	1524.75	0	0	0	7	0	0	36.5	0	0	0	0	0	0	295	0	25	0	0	0	0	0	22	0	0	0	0	0	0	0	423.5		1.96
1008 KNNDTYFNDDIKQFV	0	970.75	0	0	0	0	0	0	0	0	0	0	0	0	14	138.75	88	0	58	0	0	0	0	28.5	0	261.5	0	0	0	0	0	0		0.97
1009 NNDTYFNDDIKQFVK	3545.25	1271	0	0	0	0	0	0	0	0	0	72	0	0	0	0	0	0	0	0	0	0	0	0	0	0	61	0	0	0	0	0		2.65
1010 NDTYFNDDIKQFVKS	4038.25	950.25	0	0	0	0	0	0	0	0	0	0	0	0	0	0	0	0	0	0	0	0	0	0	0	0	0	0	0	0	0	0		2.26
1011 DTYFNDDIKQFVKSN	4501.5	0	0	377	0	0	0	0	1333	0	0	0	0	0	0	0	0	0	0	15.5	0	0	0	0	0	0	0	0	0	0	0	88.5		1.37
1012 TYFNDDIKQFVKSNS	2154.5	0	0	0	0	0	6	0	0	0	0	0	0	0	0	0	45.5	0	0	0	0	0	0	0	0	0	0	0	0	0	0	0		1.36
1013 YFNDDIKQFVKSNSK	4463.25	439.5	0	0	0	0	0	0	295.5	284.25	263	83.5	1748.5	6780.5	0	12527.75	14626.5	10199.75	0	1154.5	276.5	35.75	0	0	148	0	59.5	0	0	1718.5	768	2294.25		-0.39
1014 FNDDIKQFVKSNSKV	8333.75	1248.5	0	1402.5	0	0	0	0	1436	678	1677.5	304.5	3940.75	13822	320.75	19278	27320	16433.5	3089	4236	724	0	0	1470.5	0	764	202	6	357.5	4447.25	0	5242.75		-0.39
1015 NDDIKQFVKSNSKVI	4481.25	1704.5	0	397.5	6	14	11	0	0	2851.5	2137.5	472.5	6553.75	23968.25	0	19911.25	25004.75	16626.75	7014.75	4430.75	1163.5	0	38.5	2970	1205.75	1919.5	507.25	0	682.75	5852.5	1801	6914		-0.10
1016 DDIKQFVKSNSKVIT	334.5	1378.5	0	1051	0	0	0	0	1620.75	0	985	332	4696.5	12522.5	387	15489	23246	15341.25	5283.5	3531.25	1328.5	0	0	1566.5	671.5	680	0	4.25	1082	4632	0	3294.5		-0.05
1017 DIKQFVKSNSKVITG	0	0	0	0	0	0	0	0	0	136.5	140.5	0	947	3015	0	12669.5	9011.25	10653.25	1901.5	0	378.75	0	0	231.75	125.5	0	0	0	0	1643.5	0	0		-0.13
1018 IKQFVKSNSKVITGL	0	0	0	0	0	0	0	0	0	53.75	0	0	0	273.5	0	3634	982	3086.5	1334.25	273	63.75	0	0	11	73	258	0	0	0	1094	416	0		-0.10
1019 KQFVKSNSKVITGLT	0	0	0	0	0	0	0	0	409.75	0	0	145	0	0	0	3675.5	2014	2787.5	1717	431	0	506	0	25.25	264	1225.25	0	71	68.25	2739.5	0	0		0.05
1020 QFVKSNSKVITGLTE	121.5	0	0	0	0	0	0	0	0	491	0	0	0	0	0	2417.75	674.25	711.25	0	0	0	0	0	0	42.5	0	0	0	0	0	0	86.5		-0.33
1021 FVKSNSKVITGLTET	0	0	0	0	0	0	0	0	0	960.25	0	19	0	0	0	248.5	453.5	0	0	0	0	0	0	0	0	0	0	0	0	113.5	14	36.5		-0.26
1022 VKSNSKVITGLTETQ	0	0	0	0	36	0	60.5	0	0	1109.5	0	0	0	0	0	160	85	0	0	0	0	0	0	24.75	0	0	0	0	0	12.25	0	0		-0.36
1023 KSNSKVITGLTETQK	0	0	0	0	0	0	0	0	0	1077.75	0	0	0	0	0	0	2549.5	2354	14.5	0	0	0	0	0	0	824.5	0	0	0	0	0	0		-0.22
1024 SNSKVITGLTETQKN	0	0	0	0	0	23.5	0	71	0	0	1032.5	0	0	0	0	248.5	439	2194.5	0	0	0	0	0	91	0	0	0	0	0	0	0	0		-0.28
1025 NSKVITGLTETQKNA	0	0	0	0	257	887.25	103	0	0	0	0	0	0	0	0	120	114.5	2339.5	0	0	0	0	16.5	1300.5	0	24	0	0	0	0	10.5	0		-0.31
1026 SKVITGLTETQKNAL	0	0	0	0	0	0	0	0	0	0	0	0	0	34	0	233	149.5	2081.25	0	0	0	0	0	0	0	851	0	0	294.5	0	0	0		-0.28
1027 KVITGLTETQKNALN	0	0	0	0	0	0	0	0	0	0	0	0	0	0	0	0	0	851.5	0	0	0	0	0	0	0	329.5	0	0	0	0	0	0		-0.35
1028 VITGLTETQKNALND	0	0	7829	705.25	0	0	0	0	27	0	0	0	0	109.5	0	0	52.5	1704	0	0	0	0	16.5	0	32.5	0	0	0	16.5	0	0	0		1.50
1029 ITGLTETQKNALNDE	0	0	20382.5	2458	0	0	0	0	128	28.5	0	0	90.5	169.5	0	28.5	31	1190	23	76.5	0	9	54.5	19	0	0	0	135.5	37	0	0	27.5		1.77
1030 TGLTETQKNALNDEI	0	0	1331.5	0	31.5	0	25	8	99	28	0	217.5	36.25	285.75	4.5	844	32	956.5	17	898	0	8	68.75	23	18.5	0	13	99	114	0	58	68.5		-0.16
1031 GLTETQKNALNDEIK	0	0	3789.5	0	12	0	0	0	11	0	0	2	0	59	0	0	0	697	0	487.5	0	0	11	0	0	0	813.5	0	1867.5	0	30	13.5		0.60
1032 LTETQKNALNDEIKK	811.25	0	92.5	0	0	0	0	0	22	0	0	0	0	19	0	367	0	0	0	0	0	0	0	0	0	0	354	0	0	0	140.75	0		0.78
1033 TETQKNALNDEIKKL	0	0	1123.5	0	0	0	0	0	391	0	14.5	0	79.5	0	1.5	305	0	0	0	0	0	0	0	0	0	0	136	0	321.5	0	0	0		0.62
1034 ETQKNALNDEIKKLK	2376.75	0	831.25	276.5	0	0	0	0	0	0	0	0	100.5	0	0	221	0	0	0	0	0	0	0	0	0	0	0	0	147.5	0	0	0		3.13
1035 TQKNALNDEIKKLKD	691.75	0	563.5	0	0	0	0	0	0	0	0	0	0	0	0	0	0	0	0	0	0	0	0	0	0	0	61.5	14	38	0	0	0		3.41
1036 QKNALNDEIKKLKDT	0	0	435.5	267.5	0	0	0	0	0	0	0	0	0	0	0	247.5	0	0	37	0	0	0	0	0	0	0	105.5	0	398.5	0	112	0		0.87
1037 KNALNDEIKKLKDTL	1756.5	0	202	619	0	0	10.5	0	0	0	0	0	0	0	0	0	0	0	0	0	0	0	0	0	0	210.75	106.75	0	0	0	0	0		3.13
1038 NALNDEIKKLKDTLQ	0	0	16.5	0	16.5	0	0	0	0	0	14	47	0	0	0	0	0	0	0	0	0	0	0	0	0	0	31.5	0	14.5	0	91	0		-0.42
1039 ALNDEIKKLKDTLQL	0	0	12	0	10.5	0	0	0	41	0	0	0	0	34.5	0	0	0	50	0	0	0	0	23	0	0	0	0	0	0	0	10.5	0		-0.37
1040 LNDEIKKLKDTLQLS	0	0	0	0	0	0	0	0	0	0	0	0	0	62	0	0	0	128.5	0	189	0	0	0	0	0	0	0	0	0	0	90	0		-0.21
1041 NDEIKKLKDTLQLSF	0	0	0	0	0	0	0	0	0	0	0	0	0	0	0	0	0	160.75	0	0	0	0	0	0	0	0	0	0	0	0	0	0		-0.41
1042 DEIKKLKDTLQLSFD	0	6	0	0	5	0	0	0	57.5	0	0	0	0	19.5	64.5	31	0	36.5	7.5	99.5	0	0	8.5	0	0	0	0	72	0	0	0	0		-0.07
1043 EIKKLKDTLQLSFDL	0	0	0	0	0	0	0	0	33.75	19	0	0	0	89	0	0	0	0	44.5	95.5	0	0	9	0	0	0	0	17.5	0	0	0	8		-0.11
1044 IKKLKDTLQLSFDLY	0	0	0	0	9.5	0	0	0	53.5	0	0	0	0	0	0	0	0	0	15	80	0	0	0	0	0	75.5	14.75	0	0	0	0	0	11	-0.19
1045 KKLKDTLQLSFDLYN	161	0	0	0	0	0	0	0	0	0	0	0	0	0	0	0	0	0	0	0	0	0	0	0	0	206.25	0	0	0	0	0	0		0.06
1046 KLKDTLQLSFDLYNK	0	0	0	0	0	0	0	0	0	0	0	0	0	0	0	0	0	0	0	0	0	0	0	0	0	180.5	0	0	0	0	0	0		-0.41
1047 LKDTLQLSFDLYNKY	0	0	0	0	0	0	0	0	0	0	0	0	0	0	0	0	0	0	13.5	0	0	0	0	0	0	0	0	0	0	0	0	0		-0.41
1048 KDTLQLSFDLYNKYK	0	0	0	0	0	0	0	0	0	0	0	0	0	0	0	0	0	0	0	0	0	0	0	0	0	315.5	0	0	0	0	0	0		-0.41
1049 DTLQLSFDLYNKYKL	0	0	0	0	0	0	0	0	0	0	0	0	0	0	0	0	0	0	0	0	0	0	0	0	0	0	0	0	0	0	0	0		
1050 TLQLSFDLYNKYKLK	0	0	0	0	0	0	0	0	0	0	0	0	0	0	0	172	0	0	0	0	0	0	0	0	0	0	0	0	0	81.5	0	0		-0.34
1051 LQLSFDLYNKYKLKL	0	0	0	0	5.75	0	0	0	0	0	0	0	0	0	0	200.5	0	12.75	0	0	0	0	0	0	0	0	0	0	0	0	0	0		-0.40
1052 QLSFDLYNKYKLKLD	0	0	0	0	0	0	0	0	0	0	0	0	0	0	0	0	0	0	0	0	0	0	0	0	0	0	0	0	0	0	0	0		
1053 LSFDLYNKYKLKLDR	0	0	52	0	0	0	0	0	0	0	0	0	0	0	0	0	0	0	0	0	0	0	0	0	0	0	0	0	0	0	0	0		1.37
1054 SFDLYNKYKLKLDRL	0	0	0	0	0	0	0	0	0	0	0	0	0	0	0	0	65.5	0	0	0	0	0	0	0	0	0	0	0	0	0	0	0		-0.41
1055 FDLYNKYKLKLDRLF	0	0	0	0	0	0	0	0	0	0	0	0	0	0	0	6	0	0	0	23.5	0	0	0	0	0	0	4	0	0	0	0	0		-0.34
1056 DLYNKYKLKLDRLFN	0	0	0	0	0	0	0	0	0	0	0	0	0	0	0	0	0	0	0	0	0	0	0	0	0	0	0	0	0	0	0	0		
1057 LYNKYKLKLDRLFNK	0	0	0	0	0	0	0	32	0	0	0	0	0	0	0	173	560.5	0	0	152.25	0	0	0	0	0	0	0	0	0	0	29	69.5		-0.28
1058 YNKYKLKLDRLFNKK	34.5	7126	0	0	16.5	12.5	21.5	666.25	0	417.5	0	63.5	272	545	0	538	1184.25	1209.25	0	399.75	41.5	122.5	99.5	1012.75	56	697.5	577.25	142.5	0	825.25	1323	848.5		0.65

1059 NKYKLKLDRLFNKKK	0	3517.25	63.5	277	113.5	81.5	67.5	997.25	0	688.5	0	174	394.5	862.5	0	416.5	711.25	679.5	3.5	35	0	292	182.5	1200	114.5	853.5	983.5	0	40.75	494	572	690		0.32
1060 KYKLKLDRLFNKKKE	428	5882.25	60	1276	0	0	0	353.5	0	218.5	0	0	199.5	393.5	0	849.5	0	840.25	0	733.5	0	118.5	0	765.25	0	1305.5	0	0	0	0	0	442.5		1.81
1061 YKLKLDRLFNKKKEL	4468	6413.5	0	1065	0	0	0	0	0	0	0	0	0	278.5	0	894.5	418.5	1091	0	0	0	767.75	0	0	0	0	0	0	0	233.75	78.75	187.5		3.86
1062 KLKLDRLFNKKKELG	1644.5	1267	0	0	0	0	0	0	0	17	0	0	0	0	0	551.75	444.5	449	0	215	0	137.5	0	0	0	446	0	0	0	0	0	0		2.18
1063 LKLDRLFNKKKELGQ	460	0	0	0	0	0	0	0	0	0	0	0	0	0	0	564.75	0	0	0	0	0	98.5	0	48.5	0	0	0	0	0	0	29	25.5		0.01
1064 KLDRLFNKKKELGQD	298.5	379.5	0	118.75	0	0	0	0	0	44.5	0	0	0	5.5	0	0	0	11	0	0	0	47	0	77.5	0	0	0	92.25	0	0	44.5	0		4.37
1065 LDRLFNKKKELGQDK	0	0	0	0	0	0	0	0	5618	0	0	23.5	0	818	0	2381	211.5	0	694.25	114	0	102.5	94.25	0	13	268.5	784.5	0	0	1329.25	89	0		-0.18
1066 DRLFNKKKELGQDKM	922.5	679.5	0	0	0	0	0	0	5085	0	0	29.5	0	694.5	0	2544	470.5	0	883	365.25	8.5	116.5	67	112.5	11	679.75	383	0	0	0	168.5	107.25		-0.52
1067 RLFNKKKELGQDKMQ	429.25	0	0	0	0	0	0	0	3205.5	0	0	42.25	0	362	0	2129.25	591.5	0	534.5	134.75	20.5	74.5	0	82.5	0	397	176.25	0	0	976	68.75	53		-0.28
1068 LFNKKKELGQDKMQI	298.5	0	0	0	0	0	0	0	3071.5	0	0	24	0	137	0	2205.5	672.5	0	577.75	552	0	64	0	60	0	343.5	126.25	0	0	1109.75	101.5	5.25		-0.22
1069 FNKKKELGQDKMQIK	181	0	0	274.25	0	0	0	0	1523	0	0	15.5	0	272	0	2466.5	711.25	8.5	826.5	541.5	0	51.5	0	100	0	0	436.25	0	0	985.75	45.5	0		-0.26
1070 NKKKELGQDKMQIKK	0	0	0	1528.5	0	0	0	269.5	1797.5	0	0	46.5	25.5	726	0	1749	745	180	668.75	254.5	0	129.75	42.5	0	29	295.75	812.5	0	0	913.25	0	0		-0.54
1071 KKKELGQDKMQIKKL	0	0	144.5	2422.75	0	0	0	349.5	0	25	0	33.75	86.25	398.75	0	1799.75	615.75	140.5	502.25	442.5	0	0	0	892.5	17	1930.5	1361.25	0	0	1226	0	0		-0.29
1072 KKELGQDKMQIKKLT	0	0	0	2350.5	0	0	0	0	2923	0	0	37	0	984	0	2465.5	1578.5	140	643	714.5	0	143.25	25	0	21.5	1183	1020.5	0	0	1763.5	0	0		-0.56
1073 KELGQDKMQIKKLTL	0	0	0	0	0	0	0	0	583	32	0	0	0	41	0	569	0	0	0	89.5	0	501.5	0	0	0	0	0	0	0	0	0	0		-0.20
1074 ELGQDKMQIKKLTLL	0	0	0	0	0	0	0	0	0	75.5	0	0	70.5	194.5	9.75	0	0	0	0	0	240	0	30	0	0	0	0	0	0	0	0	0		-0.20
1075 LGQDKMQIKKLTLLK	0	0	0	0	0	0	0	86	0	0	0	30	0	0	0	20.25	3	0	0	0	0	0	0	101	0	0	0	0	0	10.5	24.5	0		-0.25
1076 GQDKMQIKKLTLLKE	0	0	0	391	7.5	0	0	0	0	0	0	0	0	0	0	0	0	0	0	0	0	0	0	0	0	0	0	0	0	0	0	261		0.62
1077 QDKMQIKKLTLLKEQ	0	0	0	0	0	0	0	0	0	0	0	0	0	0	0	0	0	0	0	0	0	0	0	0	0	0	0	0	0	0	0	0		
1078 DKMQIKKLTLLKEQL	0	0	0	0	0	0	0	0	0	0	0	0	0	0	0	0	0	0	0	0	0	0	0	0	0	0	0	0	0	0	0	0		
1079 KMQIKKLTLLKEQLE	89.5	0	0	875.75	0	0	0	0	0	0	0	0	0	0	0	0	0	0	0	0	0	0	0	0	0	64	0	0	0	0	0	0	12	1.71
1080 MQIKKLTLLKEQLES	79	0	0	245.5	0	0	0	0	0	0	0	0	0	0	0	0	0	0	0	0	0	0	0	0	0	0	0	0	0	0	0	0		2.56
1081 QIKKLTLLKEQLESK	0	0	0	1343.5	0	0	0	0	0	0	0	0	0	0	0	0	0	0	0	0	0	0	0	0	0	0	0	0	0	0	0	0		1.37
1082 IKKLTLLKEQLESKL	0	0	0	0	0	0	0	0	0	0	0	0	0	0	0	0	0	0	0	0	0	0	0	0	0	77	6	0	0	0	0	0		-0.40
1083 KKLTLLKEQLESKLN	0	0	0	0	0	0	0	0	0	0	0	0	0	0	0	0	0	0	0	0	0	0	0	0	0	419.75	0	0	0	0	0	0		-0.41
1084 KLTLLKEQLESKLNS	0	0	0	0	0	0	0	0	0	0	0	0	0	227.5	0	0	0	0	0	0	0	0	0	0	0	65.5	0	0	0	0	0	0		-0.36
1085 LTLLKEQLESKLNSL	0	12.5	0	0	0	0	0	0	0	0	0	0	0	0	0	0	0	0	0	0	0	0	0	0	0	0	0	0	0	0	0	0		1.37
1086 TLLKEQLESKLNSLN	17591.75	0	0	328.5	0	0	0	0	0	0	3.5	0	378	0	0	16.5	781	166	0	80.5	0	0	0	0	0	0	0	0	0	133.75	4	155.5		1.39
1087 LLKEQLESKLNSLNN	14869.25	0	212.5	246.75	0	0	0	0	0	0	0	0	14	0	0	0	362	18	0	0	0	0	0	0	0	0	0	0	0	27.5	0	66		1.47
1088 LKEQLESKLNSLNNP	10286	0	0	0	0	0	0	0	0	0	0	0	0	0	0	0	0	122.5	39.5	0	5	0	0	0	0	0	2	0	0	375.75	0	0		1.35
1089 KEQLESKLNSLNNPH	3633.75	0	0	0	0	0	0	0	0	0	0	0	602	0	0	0	0	0	0	0	0	34.5	0	0	0	0	0	0	0	0	0	0		1.25
1090 EQLESKLNSLNNPHN	3066.5	0	0	0	7.5	0	0	0	0	0	0	0	625.5	163	0	0	288.5	0	0	0	0	0	0	0	0	0	0	0	0	0	0	0		1.14
1091 QLESKLNSLNNPHNV	2620	219	0	0	0	0	0	0	0	0	0	0	0	0	0	0	0	0	0	0	0	0	0	0	0	0	0	0	0	0	0	0		1.69
1092 LESKLNSLNNPHNVL	3824.5	969	0	0	0	0	0	0	0	0	0	0	0	0	0	0	0	0	0	0	0	0	0	0	0	0	0	0	0	0	0	0		2.32
1093 ESKLNSLNNPHNVLQ	1084.5	392.5	0	237	0	0	0	0	0	0	0	0	46.5	100.5	0	0	0	0	0	0	0	0	0	0	0	0	0	0	0	0	0	0		3.78
1094 SKLNSLNNPHNVLQN	0	324.75	0	2204	0	0	0	0	0	0	0	0	0	0	0	0	0	0	0	0	0	0	0	0	0	0	33	0	0	0	0	0		1.92
1095 KLNSLNNPHNVLQNF	0	282.5	0	3172.75	0	0	0	0	0	0	0	0	0	0	0	0	0	0	0	0	0	0	0	0	0	144	0	0	0	0	0	0		1.68
1096 LNSLNNPHNVLQNFS	248	137.5	0	2314.5	0	0	0	0	0	0	0	0	0	578	0	0	0	0	706	0	0	0	0	0	0	0	0	0	0	0	0	0		1.45
1097 NSLNNPHNVLQNFSV	0	68	0	995.25	0	0	0	0	0	0	0	22.25	0	0	0	0	0	0	0	68	0	0	0	0	0	0	28.5	0	0	0	0	0		1.56
1098 SLNNPHNVLQNFSVF	0	0	0	667.25	0	0	0	0	0	0	0	0	0	0	0	0	0	0	14	3	0	0	0	0	0	0	0	0	0	0	0	0		1.36
1099 LNNPHNVLQNFSVFF	0	86.5	0	0	0	0	0	0	7.5	0	0	0	0	0	0	0	0	0	0	20.75	0	0	0	0	0	0	0	0	0	0	0	0		1.13
1100 NNPHNVLQNFSVFFN	0	0	0	0	0	0	0	0	0	0	0	50	0	0	0	0	0	0	0	0	0	0	0	0	0	0	80	0	0	0	0	0		-0.33
1101 NPHNVLQNFSVFFNK	0	0	0	0	0	0	0	0	0	0	0	0	0	0	0	0	0	0	0	195	0	0	0	0	0	0	0	0	0	0	0	0		-0.41
1102 PHNVLQNFSVFFNKK	122	0	0	0	0	0	0	0	0	0	0	0	0	0	0	0	0	0	0	86.75	0	0	0	0	0	0	0	0	0	0	0	0		0.57
1103 HNVLQNFSVFFNKKK	0	105.5	105.5	382	0	0	23	750	64	58.5	0	0	0	136.5	0	0	0	175.5	0	383	0	0	0	1009.25	0	0	0	0	0	236.5	33.5	0		-0.35
1104 NVLQNFSVFFNKKKE	442	0	0	826.25	0	0	0	0	0	0	0	0	31.5	0	0	0	0	0	0	72.5	0	0	0	0	0	0	0	0	0	0	0	0		3.05
1105 VLQNFSVFFNKKKEA	428.5	0	0	0	0	0	0	0	0	0	0	0	0	0	0	0	0	0	4.5	0	0	335.5	0	0	0	0	0	0	0	0	0	0		0.48
1106 LQNFSVFFNKKKEAE	710	0	0	140.75	3	0	0	0	0	0	0	0	141.5	0	0	0	0	29.5	16	78	0	0	0	0	0	0	0	16.25	0	0	0	0		1.80
1107 QNFSVFFNKKKEAEI	1031.5	0	0	0	0	0	0	0	0	0	0	0	20	0	0	0	0	56	0	220	0	0	0	0	0	0	0	0	0	0	0	0		1.17
1108 NFSVFFNKKKEAEIA	509.5	0	0	0	0	0	18	0	0	0	0	0	0	0	31.5	0	0	0	0	0	0	0	0	0	0	0	0	0	0	0	0	0		1.34
1109 FSVFFNKKKEAEIAE	1172.75	0	0	0	0	0	0	0	0	0	0	0	0	0	0	0	0	107.75	0	0	0	0	0	0	0	0	0	43	0	19.75	0	8		1.29
1110 SVFFNKKKEAEIAET	6787	143	0	0	0	0	46	0	0	1.5	0	0	9.75	0	0	0	0	0	0	0	0	0	0	0	0	0	0	0	0	36	0	202		1.43
1111 VFFNKKKEAEIAETE	1533.75	567.75	190.25	0	0	0	152	0	35.75	3	126.25	0	94.5	10	0	31	0	0	0	0	0	30.5	7	0	0	0	687.25	65.25	88	70.5	6.5	88.5		2.30
1112 FFNKKKEAEIAETEN	352.5	321.5	647	0	0	0	87	0	0	0	20	0	103	11	0	11	0	44.5	0	0	0	25	0	21	0	0	22	55	0	15	0	157.25		4.97
1113 FNKKKEAEIAETENT	428	569	0	4527.25	0	0	54.5	0	0	0	0	0	27.5	0	0	0	14	117.5	0	69	0	32	0	0	0	0	42.25	0	10.5	28.5	0	324		2.18
1114 NKKKEAEIAETENTL	166.5	203.5	0	872.5	0	0	5	0	0	0	0	52.5	0	0	0	0	0	64.5	0	0	0	0	0	0	6.5	0	14.5	0	0	0	0	200.5		2.77
1115 KKKEAEIAETENTLE	187.5	83.5	0	1207.25	7.75	0	27	0	0	33.5	0	0	42	0	0	0	0	241.5	0	0	0	0	0	0	0	0	59.5	78.5	0	0	0	219.5		1.81
1116 KKEAEIAETENTLEN	262.5	182	262	1726	0	0	57.5	0	47.25	0	0	0	330	15.5	0	44.5	216.5	170.25	0	0	0	203.5	0	0	23	116.5	0	119.5	0	0	0	246		2.46
1117 KEAEIAETENTLENT	166	259.5	717	2104	0	3.5	94.5	7	0	146.75	0	0	749.5	0	613	46.5	250.5	398	0	0	0	84.5	0	0	0	0	12.5	61	135.5	0	0	124.5		2.34
1118 EAEIAETENTLENTK	17.5	0	80	1538.75	0	0	0	0	0	328.5	0	0	781.75	80	137.5	25	0	147.75	0	0	0	0	0	0	0	0	0	30	42.5	0	0	0		0.77
1119 AEIAETENTLENTKI	0	17.5	183.5	950.5	0	0	0	0	0	20	0	0	254.25	0	187.5	0	0	0	0	0	0	0	10.25	0	0	0	0	28	0	0	0	5.5		1.68
1120 EIAETENTLENTKIL	41	0	0	1044.75	0	0	76	0	0	326.25	0	0	546.5	192	136	0	0	0	0	0	0	0	217	0	0	0	0	0	44.5	0	0	32		0.50
1121 IAETENTLENTKILL	28	0	0	457.75	0	0	0	0	0	0	0	0	36.5	0	0	0	0	0	0	0	0	0	92	0	0	0	0	0	688.5	0	0	0		-0.05
1122 AETENTLENTKILLK	20	0	0	0	0	0	0	0	0	0	0	0	244	0	0	0	0	0	0	0	0	0	0	0	0	0	0	0	0	0	0	0		-0.50
1123 ETENTLENTKILLKH	9	20	0	0	0	0	0	0	0	74	0	0	584	43.5	27.5	0	0	0	0	0	0	0	25.5	0	0	0	0	0	0	0	0	0		-0.41
1124 TENTLENTKILLKHY	20.5	0	0	617.5	0	0	0	0	10.5	0	0	0	0	0	0	0	0	0	0	0	0	0	0	0	0	0	0	0	0	0	0	0		1.49
1125 ENTLENTKILLKHYK	0	0	0	0	0	0	0	0	0	0	0	0	0	63	0	0	0	0	0	0	0	0	0	0	0	0	0	0	0	0	0	0		-0.41
1126 NTLENTKILLKHYKG	0	0	0	0	0	0	0	0	0	0	0	0	0	0	0	0	0	0	0	0	0	0	0	0	0	0	0	0	0	0	0	0		
1127 TLENTKILLKHYKGL	0	0	0	0	0	0	0	0	0	0	0	0	0	0	0	0	0	0	0	0	0	0	0	0	0	0	0	0	0	0	0	0		
1128 LENTKILLKHYKGLV	0	0	0	0	0	0	0	0	0	0	0	0	0	0	0	0	0	0	0	0	0	196.5	0	0	0	0	0	0	0	0	0	0		-0.41
1129 ENTKILLKHYKGLVK	0	0	0	0	0	0	0	35	0	0	0	2.25	0	0	0	0	0	0	0	0	0	0	0	0	0	0	22.5	0	0	235.5	0	0		-0.39
1130 NTKILLKHYKGLVKY	0	0	0	0	0	0	0	0	0	0	0	45	0	0	0	46	283.75	0	0	0	0	0	0	0	0	947.75	0	0	0	185	0	0		-0.31
1131 TKILLKHYKGLVKYY	0	0	0	0	0	0	0	0	0	0	0	0	0	0	0	71	969.5	0	0	0	0	0	0	0	0	0	0	0	0	126.5	0	0		-0.37
1132 KILLKHYKGLVKYYN	0	0	0	0	0	0	0	0	0	0	0	0	0	0	0	0	0	0	0	0	0	0	0	0	0	1841	0	0	0	0	0	0		-0.41
1133 ILLKHYKGLVKYYNG	0	0	284.75	0	0	0	0	0	0	0	0	0	0	0	0	0	0	0	0	0	0	0	0	0	0	0	0	0	0	0	0	0		1.37
1134 LLKHYKGLVKYYNGE	0	0	404	0	0	0	0	0	0	0	0	0	0	0	0	0	0	20.5	0	0	0	0	0	0	0	0	0	0	0	0	0	0		1.35
1135 LKHYKGLVKYYNGES	0	0	597.5	123	0	0	0	0	0	0	0	0	0	0	0	0	0	0	0	0	0	0	0	0	0	0	0	0	0	0	0	0		2.15
1136 KHYKGLVKYYNGESS	0	0	0	0	0	0	0	0	0	0	0	0	0	0	0	0	0	0	0	0	0	0	0	0	0	618.5	0	0	0	0	0	0		-0.41
1137 HYKGLVKYYNGESSP	0	0	94	289.5	0	0	0	0	0	0	0	0	0	0	0	0	0	0	0	0	0	0	0	0	0	0	0	0	0	0	0	0		2.57
1138 YKGLVKYYNGESSPL	0	0	92.5	860	0	0	0	0	0	0	0	0	0	0	0	0	0	0	0	0	0	0	0	0	0	0	0	0	0	0	0	0		1.78
1139 KGLVKYYNGESSPLK	0	0	0	0	0	0	0	0	0	0	0	0	0	0	0	0	0	0	0	0	0	0	0	0	0	0	0	0	0	0	0	0		
1140 GLVKYYNGESSPLKT	0	0	0	0	0	0	0	0	0	0	0	0	0	0	0	0	0	0	0	0	0	0	0	0	0	0	0	0	0	0	0	0		
1141 LVKYYNGESSPLKTL	0	0	0	0	0	0	0	0	0	0	0	0	0	0	0	0	0	0	0	0	0	0	0	0	0	0	0	0	0	0	0	0		
1142 VKYYNGESSPLKTLS	0	0	911.75	0	0	0	0	0	0	0	0	0	0	0	0	0	0	0	0	0	0	0	0	0	0	0	0	0	0	0	0	0		1.37
1143 KYYNGESSPLKTLSE	0	0	0	0	0	0	0	0	0	0	0	0	0	0	0	0	0	0	0	0	0	0	0	0	0	131.5	0	0	0	0	0	0		-0.41
1144 YYNGESSPLKTLSEV	0	0	134.75	0	0	0	0	0	11	53	0	0	0	0	0	0	0	0	0	0	0	0	0	0	0	0	0	0	0	0	0	0		0.94
1145 YNGESSPLKTLSEVS	0	0	4606.5	0	0	0	0	0	0	0	0	0	0	0	0	0	0	0	0	0	0	0	0	0	0	0	0	15	0	0	0	0		1.37
1146 NGESSPLKTLSEVSI	0	0	1477.5	0	0	0	0	30	0	0	0	0	0	435	0	0	0	0	0	0	0	0	0	0	0	0	37.5	0	0	0	0	0		1.10
1147 GESSPLKTLSEVSIQ	0	0	1174.25	0	16	0	0	0	0	59	0	0	0	0	0	0	0	0	0	0	0	0	0	0	0	0	0	0	0	0	0	0		1.35
1148 ESSPLKTLSEVSIQT	0	0	276.5	738	0	0	0	0	0	64.75	0	0	333.5	172.5	36	0	0	0	0	0	0	0	0	0	0	0	0	0	0	0	0	0		1.73
1149 SSPLKTLSEVSIQTE	53	0	1172.25	0	30.5	65	0	22.5	3126.75	0	103.25	44	0	22.5	0	132.5	0	0	0	0	0	0	128.5	0	0	0	0	0	143.25	0	0	0		-0.39
1150 SPLKTLSEVSIQTED	113	484	889.5	312.75	21	185.25	0	23.5	60	0	0	233	22.5	62	0	175.5	31.5	0	0	0	0	0	175	0	0	0	438	228	98	0	59	0		3.52
1151 PLKTLSEVSIQTEDN	0	0	0	0	95.5	69	0	0	0	0	0	41.75	0	3	0	0	0	0	0	0	0	0	64.5	41.5	0	0	127	77.5	17	0	0	0		-0.12
1152 LKTLSEVSIQTEDNY	0	0	425.75	512.25	61.5	34	0	0	43.5	0	0	0	0	0	0	0	40.5	91.5	0	142.75	0	0	40	29	0	0	129.5	0	39	0	0	0		2.63
1153 KTLSEVSIQTEDNYA	0	0	0	0	0	83	0	0	0	0	0	0	0	0	0	52.5	0	0	0	0	0	0	0	0	0	75.5	0	0	0	0	0	0		-0.33
1154 TLSEVSIQTEDNYAN	0	0	107	0	0	63.75	0	0	0	0	3	0	0	0	0	97	0	0	0	0	0	0	69	22	0	0	0	0	0	0	0	0		0.05
1155 LSEVSIQTEDNYANL	0	0	0	0	0	36.5	0	0	0	0	0	0	0	0	0	126.75	0	0	0	73	0	0	0	0	0	0	0	0	0	0	190	0		-0.27
1156 SEVSIQTEDNYANLE	172	0	2409.25	584.25	15.25	49.5	0	13.5	38.5	0	12.75	14	0	0	0	91	35.75	0	10	0	0	9.5	109	0	0	0	343.25	100	46.5	0	0	0		2.39
1157 EVSIQTEDNYANLEK	0	0	0	0	0	19.25	0	0	0	35.5	0	0	188	37.5	28	0	0	0	0	0	0	0	0	0	0	0	0	0	0	0	0	0		-0.31
1158 VSIQTEDNYANLEKF	0	0	0	0	3.5	0	179	0	0	0	0	0	0	0	0	0	0	0	0	0	0	0	275	0	0	0	91.5	21.5	0	0	0	0	13	-0.34
1159 SIQTEDNYANLEKFR	0	0	0	0	5.5	46.5	40	129	0	88.5	0	526.5	862	0	0	0	0	0	0	0	0	0	7688.5	0	0	0	0	0	0	0	0	0		-0.38
1160 IQTEDNYANLEKFRV	0	0	0	0	0	9.5	0	0	0	0	0	0	214	0	0	0	0	0	0	0	0	0	0	0	0	0	0	0	1400	0	0	0		-0.38
1161 QTEDNYANLEKFRVL	0	0	0	0	0	0	0	0	0	0	0	0	0	0	0	0	0	0	0	0	0	0	0	0	0	0	0	0	0	0	0	0		
1162 TEDNYANLEKFRVLS	0	0	0	0	0	0	0	0	0	0	0	0	0	0	0	0	0	0	0	0	0	0	0	0	0	0	0	0	0	0	0	0		
1163 EDNYANLEKFRVLSK	0	0	0	396.75	0	0	0	0	0	0	0	0	0	0	0	0	18.5	0	0	0	0	0	0	0	0	0	0	0	0	40.5	0	0		1.28
1164 DNYANLEKFRVLSKI	522.5	0	0	0	0	0	0	0	0	0	0	0	0	0	0	0	187	0	0	0	0	0	0	0	0	0	0	0	0	93	0	5		0.92
1165 NYANLEKFRVLSKID	0	0	762.75	0	0	0	0	0	0	0	0	0	0	0	0	0	0	0	0	0	0	0	0	0	0	0	0	27.5	0	50.5	3.75	0		1.31
1166 YANLEKFRVLSKIDG	0	0	0	0	0	0	0	0	0	0	0	0	0	0	0	0	0	0	0	0	0	0	0	0	0	0	0	0	0	0	0	0		
1167 ANLEKFRVLSKIDGK	0	0	0	0	0	0	0	0	0	0	0	0	0	0	0	0	0	0	0	0	0	0	0	0	0	0	0	0	0	0	0	0		
1168 NLEKFRVLSKIDGKL	0	3172	0	0	0	0	0	0	0	0	0	0	0	0	0	0	0	0	0	0	0	0	0	0	0	0	0	0	0	0	0	0		1.37
1169 LEKFRVLSKIDGKLN	0	632.25	8	0	0	0	0	0	0	0	0	0	0	0	0	0	0	0	0	0	0	0	0	0	0	0	0	0	0	12.5	0	0		1.41
1170 EKFRVLSKIDGKLND	0	0	180.5	76.5	0	0	59	0	0	0	0	0	0	0	13	0	0	98	0	0	0	0	0	0	0	0	0	0	0	0	0	0		1.86
1171 KFRVLSKIDGKLNDN	0	127.25	100	350.5	0	0	0	0	0	0	0	0	0	0	0	0	0	124.5	0	22.5	0	0	0	0	0	0	0	0	0	0	0	0		3.44
1172 FRVLSKIDGKLNDNL	0	528.5	0	617.5	0	0	0	0	0	0	0	0	0	0	0	0	0	0	0	0	0	0	0	26.5	0	0	0	0	0	0	0	0		3.52
1173 RVLSKIDGKLNDNLH	0	0	0	214.25	0	0	0	0	0	0	0	0	0	0	0	0	0	183	0	0	0	0	0	0	0	0	0	0	0	0	0	0		0.41
1174 VLSKIDGKLNDNLHL	0	508.5	0	110.5	0	0	0	0	0	0	0	0	0	0	0	0	0	0	0	0	0	0	0	0	0	0	0	0	0	0	0	0		2.19
1175 LSKIDGKLNDNLHLG	0	242.5	0	170	0	0	0	0	0	0	0	0	0	0	0	0	0	0	0	0	0	0	0	0	0	0	0	0	0	0	0	0		3.42
1176 SKIDGKLNDNLHLGK	0	558.25	0	0	0	0	0	0	0	0	0	0	0	0	0	0	0	0	0	0	0	0	0	0	0	0	0	0	0	0	0	0		1.37
1177 KIDGKLNDNLHLGKK	0	443	0	0	0	0	0	0	0	0	0	0	0	0	0	0	0	0	0	1362.75	0	0	0	0	0	712	0	0	0	0	0	0		-0.52
1178 IDGKLNDNLHLGKKK	0	120.5	505.75	0	0	0	0	323.5	0	0	0	0	46.5	21.25	0	0	0	0	0	0	0	0	0	406.5	0	0	0	42	0	0	0	0		0.88
1179 DGKLNDNLHLGKKKL	0	0	2285.75	1956.25	0	0	0	185	0	0	0	90	0	0	0	0	0	0	0	4898	0	0	0	120	0	0	0	0	0	0	23	0		0.28
1180 GKLNDNLHLGKKKLS	0	444.75	1460.5	2390.5	18	0	0	225.5	0	0	0	117	0	0	0	0	0	74.5	0	4591.25	0	0	0	121	0	0	0	0	0	0	0	0		0.42
1181 KLNDNLHLGKKKLSF	0	317.25	1028.5	2803.75	86.5	0	0	0	0	0	0	162	0	72.5	0	351.5	0	0	0	0	0	0	0	0	0	314.5	0	0	0	0	0	0		3.04
1182 LNDNLHLGKKKLSFL	0	0	960.5	0	0	0	0	0	0	0	0	0	0	0	0	0	0	0	0	342.75	0	0	0	8.5	0	0	0	0	0	0	0	0		1.02
1183 NDNLHLGKKKLSFLS	0	0	329.5	0	0	0	0	0	0	0	0	735.5	0	0	0	37.5	61.5	0	0	0	0	0	0	0	0	0	0	0	0	3132.25	0	0		-0.48
1184 DNLHLGKKKLSFLSS	0	0	0	0	0	0	0	0	0	0	0	2910	0	71.5	0	0	96.5	0	0	0	0	0	0	19	0	52.5	0	0	0	3215.5	17	256		-0.30
1185 NLHLGKKKLSFLSSG	0	0	0	0	0	0	0	0	0	0	0	3314.75	0	155	0	0	87.5	0	0	0	0	0	0	0	0	56.5	0	0	0	2243.75	0	229		-0.30
1186 LHLGKKKLSFLSSGL	0	0	0	1107.5	0	0	0	0	0	0	0	2527.5	0	0	0	0	0	0	0	0	0	0	0	27.5	0	78.5	0	0	0	2736.25	277.5	293.75		-0.55
1187 HLGKKKLSFLSSGLH	0	0	568.5	1858.75	0	0	0	10	0	0	0	1668	0	0	0	0	0	0	301.25	72	0	0	0	27	0	67.5	0	0	0	1831	0	148.5		0.24
1188 LGKKKLSFLSSGLHH	0	0	1105	2251.75	0	0	0	0	0	0	0	331	0	0	0	0	0	0	0	0	0	0	0	31	0	166.5	0	0	0	542.5	0	20.5		2.55
1189 GKKKLSFLSSGLHHL	0	0	2399	1790.75	27.75	0	0	0	0	104	0	316	0	0	0	0	0	0	0	0	0	0	0	30	5	268.5	0	0	0	307.5	0	0		3.13
1190 KKKLSFLSSGLHHLI	0	0	0	763	0	0	0	0	0	0	0	403.75	0	0	0	0	0	0	0	0	0	0	0	61.5	0	5582	0	0	0	0	0	0		-0.53
1191 KKLSFLSSGLHHLIT	0	0	0	0	0	0	0	0	0	0	0	340	0	0	0	0	0	0	0	0	0	0	0	0	0	3373.25	0	0	0	165	33.5	10		-0.38

1192	KLSFLSSGLHHLITE	0	0	0	0	0	0	0	0	0	0	0	0	0	0	0	33.5	40.5	0	0	0	0	0	0	0	0	383	0	0	0	0	54.5	15		-0.34
1193	LSFLSSGLHHLITEL	93.25	0	101	709	0	0	7	0	0	0	0	0	0	63.5	0	0	0	0	11.75	97.5	0	0	0	0	0	0	112.5	0	0	0	0	11.5		2.15
1194	SFLSSGLHHLITELK	0	0	0	0	0	0	0	0	0	0	0	160	0	0	0	0	0	0	0	0	0	0	0	0	0	0	20.5	0	0	0	0	0		-0.39
1195	FLSSGLHHLITELKE	334.5	0	0	0	0	0	0	0	0	0	0	0	0	0	0	22.5	0	0	0	0	0	11.5	16	0	0	0	0	0	0	0	17	0		1.27
1196	LSSGLHHLITELKEV	0	0	0	0	0	5.5	0	0	0	0	0	0	0	0	0	0	0	0	0	0	0	1036.25	0	0	15	0	24.5	0	0	0	0	6		-0.40
1197	SSGLHHLITELKEVI	0	0	0	0	0	5	0	0	182	0	45.5	0	0	0	0	36.5	0	0	0	0	0	150	0	0	0	0	196.5	0	0	0	12	9.25		-0.16
1198	SGLHHLITELKEVIK	0	0	0	0	0	0	0	0	0	0	0	0	679.25	0	0	0	0	0	0	0	0	0	0	0	0	0	0	0	0	0	0	0		-0.41
1199	GLHHLITELKEVIKN	0	0	0	0	0	0	0	0	0	0	0	0	0	0	0	0	0	0	0	0	0	0	0	0	0	0	0	0	0	0	0	0		
1200	LHHLITELKEVIKNK	0	0	0	0	0	4.25	0	0	0	0	0	83.5	693	0	0	0	0	0	0	97.25	0	54.5	0	0	0	0	0	0	0	1391.5	0	0		-0.31
1201	HHLITELKEVIKNKN	0	0	0	201.5	0	0	0	0	0	0	0	60	361.5	0	0	0	171.5	0	0	0	0	28	0	0	0	0	0	0	0	0	0	0		-0.34
1202	HLITELKEVIKNKNY	0	0	0	0	0	0	0	0	0	0	0	504.75	0	0	0	0	0	0	0	0	0	0	0	0	0	0	0	0	0	179.5	0	0		-0.36
1203	LITELKEVIKNKNYT	0	0	0	0	0	0	0	0	0	0	0	0	464.5	0	0	0	0	197	0	0	0	6.5	0	0	0	0	0	0	0	228	0	0		-0.28
1204	ITELKEVIKNKNYTG	0	0	0	0	0	0	0	0	0	0	0	0	0	0	0	0	0	0	0	0	0	0	0	0	0	0	0	0	0	194.5	0	426.5		-0.35
1205	TELKEVIKNKNYTGN	0	0	0	0	0	0	0	9.5	0	0	267	869	936.5	2558.5	79	2276	5012	7349.25	10066	0	650.25	1185	0	2701.25	0	977.75	57.5	115.5	59.5	6772.5	1983	16204.5		0.07
1206	ELKEVIKNKNYTGNS	0	0	0	0	0	0	0	0	0	68.5	0	567.5	409	1658.25	136	0	1585.5	6187.75	8237	0	552	1350.25	813.5	876.75	0	649.25	17	129.5	173	4972	1616.5	7911.5		0.07
1207	LKEVIKNKNYTGNSP	3737	0	180.5	280.5	0	0	42.5	0	0	427.75	529.5	1794.5	1797	6149.75	297	1186.5	4641.75	7452	9199	0	882	2686.5	1537.25	2819.5	174.5	1559.75	83.5	344.5	554	13938	6944	15445.5		-0.07
1208	KEVIKNKNYTGNSPS	2344	95.5	0	0	0	0	0	0	0	160	385.25	1467.5	1735.5	4097.25	199.5	1048.5	4789	6590.25	9438.25	0	975.5	2586.75	1076	2755.75	0	1189	31.5	224.75	665.5	10191	5670.5	10644.75		0.07
1209	EVIKNKNYTGNSPSE	12589.5	83	0	883	0	0	70.5	0	0	249.5	0	941	1296.5	1898.25	142	1502.5	2909.75	5624.25	7507	222.5	425.5	1210.75	562.5	1482	0	0	45	171.5	343.5	9479.75	2305	8986.5		-0.23
1210	VIKNKNYTGNSPSEN	7116	86.5	0	3942	0	0	0	0	0	0	101.75	551.5	0	858.75	133	1992.5	1578.75	4813.25	5972.5	0	111	0	481.5	549.5	0	527.5	42	0	94	6361.5	1817.5	5601		0.04
1211	IKNKNYTGNSPSENN	1791.5	110	0	11103.5	0	0	0	0	0	0	0	1016.25	705.75	1196.75	16.5	1541	1631.5	6394.25	4021.75	0	196	0	0	950.5	0	355.5	49	0	116	5684.25	1516.75	5046.5		0.21
1212	KNKNYTGNSPSENNK	0	6	0	3071.25	0	0	0	0	0	0	248.5	1221.5	1963.25	4008	32	2135	3548.75	7496	4999.25	32.5	457	2979.75	985.5	2233.5	30.5	1612	99.5	297.5	359.5	9648.25	4288	9302		-0.03
1213	NKNYTGNSPSENNKK	0	0	0	0	0	0	0	0	0	0	0	0	0	0	0	655.25	0	1243	0	0	0	2429.5	0	0	0	0	0	0	0	1078.75	0	388		-0.20
1214	KNYTGNSPSENNKKV	0	0	0	0	0	0	0	0	0	0	0	0	20	289.25	0	1034.5	483.25	1218	371.5	0	88.5	0	0	65	0	684	0	0	0	2277.75	220.5	936.25		0.00
1215	NYTGNSPSENNKKVN	247.5	0	0	499.5	0	0	0	0	0	0	0	0	0	0	0	182.5	0	0	0	0	0	406.5	0	0	0	0	0	0	0	0	0	0		1.22
1216	YTGNSPSENNKKVNE	322.5	694.5	0	1836.75	0	0	0	0	0	0	0	0	0	0	0	0	0	0	0	111	8	463.5	0	0	0	0	0	0	0	0	0	0		3.27
1217	TGNSPSENNKKVNEA	0	0	0	213	0	0	0	0	0	0	0	0	0	0	0	0	0	0	0	0	0	0	0	0	0	0	0	0	0	0	0	0		1.37
1218	GNSPSENNKKVNEAL	0	0	0	0	0	0	0	0	0	0	0	0	0	0	0	0	0	0	0	0	0	0	0	0	0	0	0	0	0	0	0	0		-0.41
1219	NSPSENNKKVNEALK	0	0	0	1742	0	0	0	0	0	0	0	0	0	0	0	0	0	0	0	0	47	390	0	0	0	0	0	0	0	0	0	0		1.18
1220	SPSENNKKVNEALKS	0	0	0	6920	0	0	0	0	0	0	0	0	0	0	0	0	0	0	0	0	0	0	0	0	0	0	0	0	0	0	0	0		1.37
1221	PSENNKKVNEALKSY	0	0	0	5095.5	0	0	0	0	0	0	0	0	0	0	0	0	0	352.75	0	0	0	0	0	0	0	0	0	0	0	0	0	0		1.33
1222	SENNKKVNEALKSYE	0	0	0	3914.75	0	0	0	29	0	0	0	0	0	0	0	0	0	1605.5	0	0	0	0	0	0	0	0	0	0	0	0	0	0		0.96
1223	ENNKKVNEALKSYEN	0	0	0	2942.5	0	0	0	17	0	0	0	0	0	0	0	0	0	448	0	0	0	0	0	0	0	0	0	0	0	0	0	0		1.26
1224	NNKKVNEALKSYENF	0	748.75	0	0	0	0	0	0	0	0	0	18.5	0	0	0	0	91.5	0	1507.75	0	0	0	0	0	0	0	0	0	0	0	0	0		-0.26
1225	NKKVNEALKSYENFL	0	3188.5	0	0	0	0	0	0	460	0	0	0	0	0	0	471	135.5	0	1625.25	0	0	0	0	0	0	0	0	0	0	0	0	719.5		0.58
1226	KKVNEALKSYENFLP	0	14033.25	0	0	0	0	0	0	3012.75	146.5	71.5	0	289.5	541	124	1435	1105.5	980.25	2285.25	2888	1065	224.5	173	911	0	634.5	91.5	0	0	255.5	330.5	4584.75		0.55
1227	KVNEALKSYENFLPE	17.5	16253	0	0	0	0	0	0	5163	344	225.5	72	607.75	1839	327	2597.5	2910.25	2265.75	4100.75	10840	1973	347	346	1703	82	1591	286	0	371.5	1919.25	1677	9296.75		-0.12
1228	VNEALKSYENFLPEA	0	8157	0	0	0	0	0	0	7938.25	469.5	388	36.5	1067.25	1756	518.5	2650	1913	1782.5	3330.5	8755	895.75	823	695.75	1437.5	24	1059.75	207.5	0	741	1688.25	2011.25	10632		-0.56
1229	NEALKSYENFLPEAK	289.5	6920.25	0	0	0	0	0	0	2174	28	0	0	0	590.25	139	4189	1342.25	3233.25	802.25	4623.25	541.5	112.5	0	566.25	0	390	0	0	110	560.75	277.25	3315.25		-0.16
1230	EALKSYENFLPEAKV	270	17392	0	0	0	0	0	0	1258.5	77	0	0	471	299	52	5677	1858	4778	2432.25	5617.5	641.25	0	0	278.25	0	231.25	9.5	0	142	451.25	432	2296.5		0.50
1231	ALKSYENFLPEAKVT	826	46677	1719	0	0	0	0	0	1174	0	0	0	197	356.75	20.5	7298	1882.75	6566.75	2931	3648.5	281.75	0	96.25	569.75	0	309.5	0	0	104.5	515	817.5	1492.5		1.22
1232	LKSYENFLPEAKVTT	845	43921	0	0	0	0	0	0	1893	0	0	0	0	375	131.5	10813	2785	10357.75	3021.25	8363.5	1774.75	0	147.5	458	20	471.5	0	0	190.25	815.25	1793.75	1666		0.82
1233	KSYENFLPEAKVTTV	2249.5	57978.5	0	0	0	0	0	0	1500.5	0	0	0	847.5	1025	138.5	13204.25	4184	8080.5	4110.75	10128.75	2476.5	0	109.5	1342.5	77	1162	0	0	1113.5	523.25	528.5	2888.25		0.97
1234	SYENFLPEAKVTTVV	5974.25	64074	0	1553.5	0	0	0	8.5	2920.5	0	167	136.5	299.25	1493	225	8318.75	5078.75	6972.25	2080.25	13279.5	2677.25	626.75	0	1686	205	1463	567.25	0	878.5	475.75	2082.5	7339.5		1.26
1235	YENFLPEAKVTTVVT	5891	51558.25	223.5	0	0	0	0	17	910.5	168.5	1.5	19.75	465.75	888.5	142.5	10542.5	4065	6498.25	659.5	6126	1608.25	248.75	0	698.75	99.5	1072	322.75	0	424.5	427.5	1303.75	4485.5		1.31
1236	ENFLPEAKVTTVVTP	6935.25	44963.25	0	0	218.5	0	0	0	0	0	0	0	103.5	0	0	3128.5	500.5	1660.75	0	0	1794	0	0	0	0	313	70.5	0	0	0	490	0		1.85
1237	NFLPEAKVTTVVTPP	4783	38436.25	0	0	0	0	0	61.75	0	0	0	0	0	0	0	788	0	897.25	582	0	1607.5	0	0	0	0	0	0	0	0	0	0	0		1.79
1238	FLPEAKVTTVVTPPQ	3379.5	26107.75	0	0	0	0	0	18	0	0	0	0	0	0	0	513	0	0	1003.75	0	1492	0	0	0	0	19	0	0	0	0	0	0		1.79
1239	LPEAKVTTVVTPPQP	1456	0	0	2782.25	0	0	0	0	0	0	0	0	0	0	0	123.5	0	0	298	0	1024.5	0	59	0	0	0	0	0	0	0	0	0		2.43
1240	PEAKVTTVVTPPQPD	2125	0	52.5	0	0	0	0	51.5	0	0	5.75	0	0	0	0	362.5	0	0	1518.25	186.5	1602.5	0	0	0	0	0	0	0	0	0	45	0		0.13
1241	EAKVTTVVTPPQPDV	1192.25	3653.5	0	0	0	0	0	40.5	0	0	0	0	0	0	0	316	0	0	1437.5	443.5	1393.5	0	0	0	0	0	0	0	0	0	100.25	0		1.46
1242	AKVTTVVTPPQPDVT	965	4468	0	0	241	0	0	0	0	0	0	0	0	0	0	770	0	0	928	218	3509.5	0	933	1536.5	0	0	0	0	0	0	728	0	14	0.49
1243	KVTTVVTPPQPDVTP	1069.5	6461	0	0	0	0	0	0	0	0	0	0	0	744.5	88.5	4994.75	388.5	0	1642.5	11727.75	3768.75	430.75	14819.25	2835.25	0	217.5	0	0	79	503.25	898	0		-0.56
1244	VTTVVTPPQPDVTPS	367.25	17490.5	0	1837.5	0	0	0	0	0	484.5	0	567.5	0	1070.5	74.5	2708	0	0	958	7647.25	930.75	905	15678.75	3177.5	0	0	0	0	1035	778.5	767.75	0		0.23
1245	TTVVTPPQPDVTPSP	32050.75	8800.25	0	0	0	0	0	0	0	833	0	439	0	1388	79	3706	239.5	1407.25	911	11813.5	2033.25	411.5	10924.5	3209	0	0	0	0	448	250	1077.75	0		1.32
1246	TVVTPPQPDVTPSPL	35902.75	13386	0	0	0	0	0	0	0	2378.5	0	647	0	1079.5	0	3196.75	196.5	1040	850.5	8064.75	1994.5	576.5	10697	2060	0	0	23	0	161	0	759.75	0		1.90
1247	VVTPPQPDVTPSPLS	34060	30525.75	0	0	0	0	0	0	0	2107.75	0	453	0	579.5	8	2743.25	148.25	946	0	9327.25	2257.75	381	6556	1317.5	0	0	0	0	222.5	309	746.75	112.5		2.91
1248	VTPPQPDVTPSPLSV	45338.75	25931	0	349.75	0	0	0	0	9988.5	847.5	0	967.25	0	529	31	2841.75	918	23041.25	1049.5	10102	1811.75	660.5	3274.75	1382.5	0	63.5	22	0	138	7924	603.5	310.5		1.79
1249	TPPQPDVTPSPLSVR	33494.75	17117.25	0	0	0	17.75	0	0	19813	1422	0	485.5	0	361.5	3208.75	1350	5961.5	57106	22600.25	8863.5	1165.5	922.5	2164.75	670.25	21.5	1613.5	30	56	40.5	16058.75	1213	154.25		-0.13
1250	PPQPDVTPSPLSVRV	31817	24016.75	0	125.5	0	0	0	0	24137.75	4382.5	0	489.5	5	258.5	1798	1581	5314	52085	11863	11284.75	955.75	314	3622.5	623.25	52	1938.5	49	0	70	13599.5	1386.5	1345		0.09
1251	PQPDVTPSPLSVRVS	23661	19024	0	0	0	0	0	0	16998	1188.5	0	618.5	2	313	865	447.75	5175.5	52473.5	17552	9393.75	576.5	324.5	1281.5	861	0	995.75	0	0	55	11243.5	673.5	820.75		-0.15
1252	QPDVTPSPLSVRVSG	10156.5	8378.5	0	0	0	0	0	0	4819	658.5	0	312.5	0	0	40.5	272.75	3861.5	28084.5	6913.5	7746.5	824	134	0	555.5	0	574	2	0	0	6290.25	303.5	228.5		-0.29
1253	PDVTPSPLSVRVSGS	15072.75	17914	0	0	0	0	0	0	6028.25	1477	23	0	0	0	196.25	0	4961	40770	13865.25	5595.75	0	0	157.5	32	33	972.5	32.5	9	0	8114.25	48.5	874.5		-0.08
1254	DVTPSPLSVRVSGSS	12789.5	16582	0	507	0	0	0	0	0	0	59	0	0	0	0	0	0	0	363.25	1494	0	0	0	0	17	18.5	0	0	0	1121.5	0	260.5		3.53
1255	VTPSPLSVRVSGSSG	475	235.25	0	13.5	0	0	0	0	0	0	4	0	0	0	0	0	0	0	0	0	0	0	0	0	0	0	0	0	0	0	0	0		3.22
1256	TPSPLSVRVSGSSGS	275.5	1382.5	0	130	0	0	0	0	0	0	6.5	12	0	0	0	0	0	0	0	644	0	0	0	0	4	0	0	0	0	976	0	0		0.93
1257	PSPLSVRVSGSSGST	0	0	0	0	0	0	0	0	0	0	85.5	36.5	0	0	0	0	0	0	0	0	0	0	0	0	0	0	0	0	0	463	0	296.75		-0.29
1258	SPLSVRVSGSSGSTK	0	359	0	0	0	0	0	0	0	0	0	0	0	0	0	0	0	0	0	0	0	0	0	0	0	0	0	0	0	0	0	0		1.37
1259	PLSVRVSGSSGSTKE	89	0	0	721.5	0	0	0	0	0	0	0	0	0	0	0	7.5	117	34.5	0	0	0	0	0	0	0	0	0	214	0	0	0	0		1.38
1260	LSVRVSGSSGSTKEE	0	5273.5	768.5	503.75	0	0	0	0	0	0	0	0	174.5	0	0	0	179	0	0	0	0	375	0	0	0	0	0	0	0	0	0	0		2.27
1261	SVRVSGSSGSTKEET	0	8398.5	979	459	0	0	0	4.5	0	0	0	0	0	0	0	0	0	0	0	0	0	0	0	0	0	0	0	0	0	0	0	50.5		2.05
1262	VRVSGSSGSTKEETQ	3217.5	9709	393	0	0	0	0	0	0	0	0	0	15.5	0	0	0	0	0	0	0	0	0	0	0	0	0	0	0	0	0	0	0		2.81
1263	RVSGSSGSTKEETQI	4011.5	9171	868.5	0	0	0	600	0	0	0	0	0	0	0	0	0	11444	0	0	0	0	0	4718.25	3336	0	0	0	0	0	0	0	0		0.51
1264	VSGSSGSTKEETQIP	6549	31530.25	446	1718	0	0	2310.5	0	0	32.5	126.5	123.5	0	0	0	0	23512	1818.75	0	12445	259	0	60769.5	7076.25	0	5802	0	0	0	136	995.75	1516.5		-0.24
1265	SGSSGSTKEETQIPT	15076.25	26777.75	0	0	0	0	0	0	0	0	453.5	236	0	2108.25	0	0	23425.5	4355	0	6941.25	0	0	55208	10209.75	0	6345.25	0	0	35	85	0	1692.75		-0.15
1266	GSSGSTKEETQIPTS	16914.75	45375.5	125.5	0	0	0	0	0	0	21.25	99.5	382.5	0	999.5	19.5	6924	22690	5518.5	0	12254.5	124	0	58432	8570.5	0	5213.5	0	0	0	67.5	1326.25	1284		0.18
1267	SSGSTKEETQIPTSG	11574.75	29007.5	0	4338.25	0	0	0	0	0	0	0	193	0	559	0	7230	19685.25	5817.5	0	12414	59	0	25923	6419.75	0	3814.5	0	0	0	0	819	752		0.55
1268	SGSTKEETQIPTSGS	16567.25	38322.25	85	9884.5	0	0	0	0	0	0	184.25	171.5	0	651	0	7183.25	20715	5993.5	0	9839	83.5	0	23491.5	5312	0	4123	16	0	0	50	1039.25	1100.25		1.57
1269	GSTKEETQIPTSGSL	13800.5	33407.5	44.75	6029.25	0	0	0	0	0	0	0	127.5	0	391.25	0	6635.5	20492.25	4616	0	11332	126.5	0	19445	4514.25	122.5	2531.5	0	0	0	50	0	876		1.22
1270	STKEETQIPTSGSLL	9323.5	23590.5	0	0	0	0	0	0	0	0	143.5	167.25	0	345.5	41.5	4686	16141.75	4460	0	9291.75	23	0	10890.5	3476	2506.5	2573	0	0	0	0	584.5	660.25		0.68
1271	TKEETQIPTSGSLLT	12421	33374.75	151	0	0	11.5	0	0	0	0	0	808.5	76	609.5	0	6039.5	18609.5	4558.5	0	14799.25	0	0	14750.75	6502.5	2579	3042	0	0	39.25	32	881.5	830.75		0.78
1272	KEETQIPTSGSLLTE	12674	33387.5	3005.5	4139.25	308.5	249.5	213	638	919.5	685.75	927.25	9151.75	3309	1901	121.5	6480.5	22865	9201	264.5	23825	158.5	2363	59081.25	8975.25	4907.25	6180.5	22.5	527	255.5	144	2087.25	1705		-0.14
1273	EETQIPTSGSLLTEL	*5378.25*	*31099.5*	*5861.75*	*4373*	*680.75*	*622*	*3276.5*	*579*	*496.75*	*624*	*393*	*7567*	*6942*	*110.25*	*121.5*	*11713*	*8913.5*	*4142*	*3599.75*	*0*	*315.5*	*24537*	*14355.5*	*1896.5*	*7762*	*0*	*64.5*	*23677.5*	*79*	*125.5*	*47715*	*0*		-0.22
1274	ETQIPTSGSLLTELQ	*0*	*10925*	*2215*	*2015*	*186*	*57.25*	*2047*	*311.5*	*648.5*	*102.5*	*306.75*	*2820.5*	*1714.5*	*0*	*0*	*10824.25*	*2199.5*	*1917.5*	*1113*	*0*	*228.75*	*13859.5*	*8166*	*1208*	*3627.25*	*0*	*0*	*13360.5*	*0*	*0*	*29272.25*	*0*		-0.57
1275	TQIPTSGSLLTELQQ	*373.5*	*4645.25*	*1896.75*	*0*	*116.5*	*36*	*636.5*	*143.5*	*95*	*3*	*133*	*1813.5*	*470.5*	*0*	*0*	*5762.5*	*1442.5*	*1741*	*849*	*1205.25*	*0*	*4335.5*	*2704*	*218.25*	*307.5*	*0*	*0*	*5212*	*0*	*0*	*11805*	*0*		-0.54
1276	QIPTSGSLLTELQQV	*0*	*4783.5*	*828.5*	*0*	*127.5*	*23*	*811.5*	*163*	*0*	*0*	*0*	*1886*	*701.25*	*0*	*0*	*6037.25*	*1696.25*	*1995.75*	*1339.75*	*934.5*	*73.25*	*2759.25*	*2422.25*	*128*	*561.25*	*0*	*0*	*2053.75*	*0*	*0*	*2739.75*	*0*		-0.41
1277	IPTSGSLLTELQQVV	*0*	*5779.25*	*333*	*0*	*127.5*	*49*	*543*	*48*	*252.25*	*5.5*	*0*	*1808.25*	*322.75*	*0*	*0*	*6167.25*	*1160.5*	*1141*	*720.5*	*505.5*	*107*	*1392*	*1718.5*	*56*	*0*	*0*	*0*	*2984.5*	*0*	*0*	*5045.5*	*0*		-0.33
1278	PTSGSLLTELQQVVQ	*0*	*0*	*433*	*0*	*43.5*	*14*	*0*	*7*	*0*	*0*	*0*	*588.5*	*0*	*0*	*0*	*3991.5*	*416*	*749*	*227.75*	*434*	*0*	*0*	*589.5*	*0*	*0*	*0*	*0*	*644.5*	*0*	*0*	*1192*	*0*		-0.31
1279	TSGSLLTELQQVVQL	*0*	*1335*	*512.75*	*0*	*247.75*	*35*	*87.5*	*76.5*	*0*	*12.25*	*2*	*2932*	*575.5*	*0*	*0*	*4063*	*1125.75*	*771.25*	*358.75*	*0*	*0*	*0*	*2375.75*	*132.5*	*69.5*	*0*	*0*	*784.5*	*0*	*0*	*1226*	*0*		-0.48
1280	SGSLLTELQQVVQLQ	*0*	*0*	*111.25*	*0*	*56*	*10.5*	*0*	*33.5*	*0*	*0*	*0*	*606*	*70.5*	*0*	*0*	*1381.5*	*380.5*	*304.75*	*73*	*349.25*	*0*	*136.75*	*576*	*0*	*0*	*0*	*0*	*500.25*	*0*	*0*	*462.5*	*0*		-0.06
1281	GSLLTELQQVVQLQN	*0*	*553.75*	*0*	*0*	*20.5*	*25*	*13.5*	*52*	*0*	*0*	*0*	*396*	*1796.75*	*0*	*0*	*869.25*	*281.75*	*371.75*	*0*	*166.25*	*0*	*202*	*1456*	*0*	*40.75*	*0*	*0*	*0*	*0*	*0*	*0*	*0*		-0.42
1282	SLLTELQQVVQLQNY	*0*	*0*	*1137.5*	*0*	*6631*	*1608.5*	*2249*	*2069*	*2090*	*1090*	*1466.5*	*12919.5*	*7998.5*	*0*	*0*	*0*	*979.25*	*1087.75*	*170.5*	*0*	*0*	*957*	*27635.5*	*2899.5*	*508.25*	*0*	*0*	*893.25*	*355.5*	*5.5*	*0*	*0*		-0.25
1283	LLTELQQVVQLQNYD	*14884.75*	*0*	*0*	*0*	*47.5*	*38.5*	*32.5*	*22.5*	*99*	*0*	*0*	*0*	*0*	*452.5*	*244.5*	*0*	*0*	*34*	*0*	*205.5*	*3*	*0*	*0*	*0*	*0*	*0*	*0*	*0*	*0*	*0*	*177.5*	*0*		1.33
1284	LTELQQVVQLQNYDE	*5884*	*695*	*701*	*0*	*48*	*44*	*21*	*279*	*5540.75*	*0*	*35*	*0*	*0*	*618.5*	*0*	*0*	*107.5*	*0*	*0*	*524.5*	*0*	*0*	*0*	*0*	*12.5*	*0*	*0*	*0*	*0*	*0*	*268.25*	*0*		0.68
1285	TELQQVVQLQNYDEE	6174	11334.5	944.5	0	62	59.5	41.5	109.5	540	89	190	0	485	1095.75	21.5	81	510.5	117	33.5	1663.5	0	104.5	101.5	0	20	0	24	181.5	0	816	140.75	0		3.24
1286	ELQQVVQLQNYDEED	5228	18770.25	891.5	290.5	129.5	76.25	71.5	159.5	387	232.5	77	54	805.25	1456.5	85.75	162.5	469.5	268.75	152	4786	11	188.5	657	102	44.5	20	139.5	481.5	60.5	1055	366	105		2.20
1287	LQQVVQLQNYDEEDD	3012.5	17728.5	1049.5	509	208.5	106	84	243	459.5	200	123.5	106.5	229.5	811.75	86	137	521	391	542.25	14586.25	16.5	196	1295.25	41.5	64.5	0	290.5	796	121	1830.75	531.5	334.75		0.83
1288	QQVVQLQNYDEEDDS	5919.75	15451	200.5	0	88.5	53	38	54.5	234.25	87	0	24	105.5	570	31.5	138	537.5	153	165.5	6351	0	105.75	433	0	40	0	69.5	366.75	0	1422.5	203.5	0		2.01
1289	QVVQLQNYDEEDDSL	5792.75	24453.75	440.5	588	113.5	60	73	120	409	83	0	0	67.5	801	32.5	348	467	257	258	8589.25	0	143.5	409.5	245	47	0	127	466	0	1930.25	203.5	0		1.85
1290	VVQLQNYDEEDDSLV	4800.5	20106.5	0	566	78	60	73.5	265.5	300	77	76.5	27.5	136.5	1443.5	20	350	448.5	149.5	122.5	5416.75	0	103	613	509.5	48.5	0	284.5	399	0	2145	377.75	0		1.87
1291	VQLQNYDEEDDSLVV	8229.25	24720	0	1019	72.5	59.5	55	140.5	447.5	64	82	0	168	1207.5	13	171.5	478	150.5	78.5	6904.25	0	128	1240.25	1487.5	39	0	364.5	331	76	2493	465.25	0		2.21
1292	QLQNYDEEDDSLVVL	18368	27379	397.75	631.5	67.5	77.5	49	155	352	48.5	17	0	84	340.25	0	0	502	111.5	107.5	7420.25	0	122	198.5	1418.5	53.5	0	68.5	370	29	2648.5	178	17		3.09
1293	LQNYDEEDDSLVVLP	2675	19816.75	217.5	0	74	75.5	40	1388.5	136.5	0	0	38.5	0	545	9.5	0	486.5	0	0	7279.75	53	119	1221.25	534.5	55.5	748.25	70.5	367.5	0	3383.25	178	0		1.27
1294	QNYDEEDDSLVVLPI	0	6334	0	0	73.5	65.5	37.5	0	0	0	0	31	0	141.5	0	0	429.5	1014.5	0	4647.5	0	70	0	0	35.5	730.5	88.5	324.5	0	1134.75	46	0		0.34
1295	NYDEEDDSLVVLPIF	0	5841.75	478.5	993	83	93	59.5	42.5	3747.5	24	326.25	222.75	158.5	399.25	24.5	1573	493.5	14523.75	46	1776.75	448.25	138	52.5	2627	33	1613	210	443	91	2656.5	55.5	2598.75		-0.48
1296	YDEEDDSLVVLPIFG	0	1026.25	0	0	90	76.5	56	55.5	4849.5	0	692.5	99	0	233.5	21.5	838.5	414.5	11150	0	789	180.5	13	91.5	1775.5	16.5	2037.5	0	289	0	1465.5	28.5	2629.5		-0.25
1297	DEEDDSLVVLPIFGE	0	3308	0	0	73.5	128.5	33	24	8906.5	80	1541.5	86.5	455.5	474.5	97.5	2682.5	814.5	16225.75	0	434.75	0	32	3697.75	5468.25	33	1536.75	98	444	151	1882.75	87.75	5333.25		-0.28
1298	EEDDSLVVLPIFGES	0	5808	0	0	32	186	0	14	6294.25	296.5	695.5	589	3623.75	290.25	81	3098.5	674	14072.5	0	276.75	257.5	0	1436	2706.5	0	1075.5	0	116	0	1143	32.5	3889.75		-0.52
1299	EDDSLVVLPIFGESE	0	5640.25	275.25	0	64	54	19	21.5	12919.5	84	5295	1248	664.5	704	238.75	4510	1173.5	16500	501.75	530.25	371.25	0	2180	2425	28	4722.5	223	220	292.5	1634.25	115	10245.5		-0.33
1300	DDSLVVLPIFGESED	2768.75	9091.75	485	0	60.5	43.5	0	0	11707.5	60	4237.5	1030	356.5	166	139	454.75	1168	17885.25	0	0	571.25	0	2552.75	5921.75	30.5	3043	134.5	579.5	251.5	1599.75	0	12543.25		-0.51
1301	DSLVVLPIFGESEDN	0	9043.75	182	516	31	20.5	0	0	6758	0	1523	246.75	309.5	0	28	3969.25	842.25	17922.5	0	1109.5	605.25	0	1383.5	2446.25	0	1236.25	0	127	0	540	0	6108.5		-0.47
1302	SLVVLPIFGESEDND	6156.25	4095.25	384.75	1040	70	43.5	18	11	6392.5	0	1728	372	60.5	15	59.5	2264.5	641	12399.75	0	768.5	0	0	1835.75	0	4.5	1825.75	48	423	108	433	32	3762.5		-0.07
1303	LVVLPIFGESEDNDE	3765.5	3427.25	542	1599	161	62.5	42.5	25.5	2634	96	662	379.25	225.5	401.25	88.5	1721	584.75	10182.5	0	818	337.25	0	1397.75	1891.5	39.75	1350	153.5	864	0	1346	104.5	3238.75		-0.06
1304	VVLPIFGESEDNDEY	984	907.25	815.5	1664.25	174.5	102	111.5	39.5	4589.25	105.5	437.5	319.5	194.5	509	393	1912.75	868.5	10118	0	650	172.5	0	1870	2298.5	55	895.5	96.5	838.75	100	776.25	115	1934.75		-0.53
1305	VLPIFGESEDNDEYL	1385	5862.5	578.75	1564.75	155	81	65.5	43	2752.75	51	509.25	384.25	198.75	433	46	1249.25	1079.5	9942	82	895.75	190.75	0	2854	1727	29.5	499.5	0	630	103.5	413.5	49.5	2031		-0.02
1306	LPIFGESEDNDEYLD	1683.25	4219.25	709	2251.5	223.5	131.5	101.5	36	388.5	144.5	92	119.5	0	187.5	136	635.5	1638	4174.5	149	1332	30	51	1891	0	43.5	52.5	53.5	1138	52	0	77	0		1.71
1307	PIFGESEDNDEYLDQ	1025.5	2456	304	2277.25	120	66	188.5	103.5	0	83	24.5	0	0	52	35	0	721.5	277.25	29.5	1014.75	0	0	236.5	0	18	0	3	837	0	0	0	0	15	4.76
1308	IFGESEDNDEYLDQV	483	2482.25	0	0	103	95.5	43	91	0	0	0	0	0	48	26.5	0	579.5	101.5	0	430.75	0	0	0	0	14.5	0	65	1032.25	0	0	20.5	0		1.22
1309	FGESEDNDEYLDQVV	242.5	2869.5	0	2129	80	53	41.5	27	0	18.5	44.5	0	0	81	24.5	0	813.25	1421.25	0	276.5	0	272	0	0	3	0	0	1045.5	25	0	88.5	63.5		2.24
1310	GESEDNDEYLDQVVT	0	2162	0	985	56	19.5	0	0	0	0	0	0	0	0	10	0	677.5	0	0	0	0	1175.5	0	0	0	0	0	473.5	71.5	0	332.5	0		1.48
1311	ESEDNDEYLDQVVTG	0	2409.25	0	0	0	17.5	0	0	0	780.25	646.5	0	1050.25	204.5	73	0	0	145.75	0	0	0	371.5	0	0	0	0	0	449.5	75.25	0	0	20.5		0.40
1312	SEDNDEYLDQVVTGE	0	2671	251.25	4684	70.5	31	0	12.5	60	0	0	0	205.5	52	8	0	972	192.5	0	0	0	1305	0	0	0	0	0	766	159.5	0	47	0		2.70
1313	EDNDEYLDQVVTGEA	0	2179	0	9351.75	0	0	0	0	0	0	0	0	534.5	201.5	0	0	0	0	0	0	0	1884.5	0	0	0	0	0	747.5	0	0	0	0		1.94
1314	DNDEYLDQVVTGEAI	0	2384.5	0	2414.5	16.5	32	9	21.75	110	0	0	0	248.5	60	11	0	1353	0	0	0	0	925.25	33	20	0	0	0	1853.5	0	0	127.5	86.5		1.49
1315	NDEYLDQVVTGEAIS	0	2054.75	0	1631.25	38	15	10.5	10	0	0	0	60	296	0	0	24.5	0	0	0	0	0	811.5	12	0	0	0	34	100	20.5	0	214.5	0		2.70
1316	DEYLDQVVTGEAISV	27	2911.25	0	2767	22.5	19	0	0	48.5	0	0	0	409.25	0	0	56	0	0	0	0	0	310	0	0	0	0	0	0	0	0	179	0		3.39
1317	EYLDQVVTGEAISVT	0	1301.5	0	1070.25	104.5	0	0	0	0	30.5	0	0	523.5	249	21.5	0	0	0	0	0	0	0	0	0	0	0	0	0	29.75	0	0	0		2.74
1318	YLDQVVTGEAISVTM	0	0	0	392.5	0	0	0	0	0	0	0	0	197	0	0	0	0	0	0	0	0	0	0	0	0	0	0	0	0	0	0	0		0.83
1319	LDQVVTGEAISVTMD	0	0	0	0	0	0	0	0	0	0	0	0	286.5	0	0	0	0	0	0	0	0	0	0	0	0	0	0	0	0	0	0	0		-0.41
1320	DQVVTGEAISVTMDN	0	443	0	239.5	0	0	0	0	0	0	0	0	154	0	0	0	0	0	0	0	0	0	0	0	0	0	0	0	0	0	0	0		2.62
1321	QVVTGEAISVTMDNI	0	259.75	0	262.25	0	0	11	0	0	0	0	0	0	0	0	0	0	0	0	0	0	0	0	0	0	0	0	0	0	0	0	0		3.58
1322	VVTGEAISVTMDNIL	143.5	0	0	0	0	0	0	0	0	0	0	0	0	0	0	0	0	0	0	0	0	0	546	0	0	0	5.5	0	54.5	0	0	0		-0.51
1323	VTGEAISVTMDNILS	42	0	0	0	0	0	0	0	0	0	0	0	0	0	0	0	0	0	0	0	0	0	183.25	0	0	0	0	0	0	0	0	0		-0.52
1324	TGEAISVTMDNILSG	40.5	0	0	0	0	0	0	0	0	0	0	0	0	0	0	0	0	0	0	0	0	0	0	0	0	0	0	0	0	0	0	0		1.37

1325 GEAISVTMDNILSGF	0	0	0	0	0	0	0	0	0	0	0	0	0	0	0	0	0	0	0	0	0	0	0	0	0	0	0	0	0	0	0	0		
1326 EAISVTMDNILSGFE	108	0	105	1374.5	108	0	0	2	46	102.5	0	0	103	286	17.25	0	0	84.5	0	0	0	0	0	0	0	0	0	69.5	0	0	0	3		1.61
1327 AISVTMDNILSGFEN	0	0	0	0	0	0	0	0	0	0	0	0	0	0	0	1180.5	0	0	0	0	0	0	0	0	0	0	0	0	71	0	0	0		-0.40
1328 ISVTMDNILSGFENE	0	4687	0	1815.75	6	0	0	0	0	49	0	0	157.5	0	20	380.5	0	0	27	0	0	9	0	0	0	0	7.5	46.5	1417	0	0	0		2.26
1329 SVTMDNILSGFENEY	9501.25	3527	198	2484.5	41.5	33.5	25.5	15	0	43	0	0	0	0	39	86.5	0	0	77	0	15	75	0	0	0	0	12.5	211.25	34.5	11	0	0		4.31
1330 VTMDNILSGFENEY D	26819.5	3678.5	867.25	3963	71.5	51	62.5	29.5	90.5	95.25	0	0	0	142.5	409	198	128.25	0	150	3035.25	0	131.5	0	0	21.5	0	44.5	318	113.5	35.5	0	0		2.60
1331 TMDNILSGFENEY DV	23923	3833.5	393.75	2068	61.5	39.5	40	20.5	0	87	0	0	0	85.5	46.5	53.5	0	0	43.5	0	0	95	0	0	37	0	34	242	91	14	0	0		2.45
1332 MDNILSGFENEY DVI	27471	4841.5	471.5	1460.5	85	60.5	64.5	31	531.25	180.75	0	0	3156.25	201	116.5	0	302.25	269.25	68.5	32732.25	375	134	0	1488	1524.25	590	59.5	476.5	158	79.5	0	301.75		0.39
1333 DNILSGFENEY DVIY	11918	3532.5	668.5	29560.5	87	94.5	95.5	42	337	37	0	0	1361	235.5	79.5	113.5	545.5	402.5	117	36683.5	489.5	116	0	1255.75	1256.5	286.5	47	452	99	285.25	25.25	186.5		0.81
1334 NILSGFENEY DVIYL	10637	2194.5	0	7645	68	89	120	30	604.5	58	0	59	2078	222	46.5	183	321.5	0	0	17197	221.25	79.5	0	1052.25	1039	0	57	357.5	50.25	268.5	0	136		0.75
1335 ILSGFENEYDVIYLK	0	1830	0	2594.25	0	0	0	0	0	0	0	0	492.5	0	0	0	545.25	0	0	7492.25	92	0	258	245	0	0	0	0	0	202.5	0	0		-0.16
1336 LSGFENEYDVIYLKP	5886.25	2147	0	13349	0	19	0	0	187.5	0	0	0	1195.5	139.5	42.5	27.5	635.5	0	0	10139	46.5	0	0	551	313	77	0	0	0	319.5	442.5	257		1.84
1337 SGFENEYDVIYLKP L	2936.25	1141.25	0	4289.25	0	25.5	0	0	0	0	0	0	583	0	0	0	331.75	0	0	17907.75	42.5	0	0	197	0	0	0	0	0	181	101	107.5		-0.27
1338 GFENEYDVIYLKP LA	2803.75	989.5	0	2545	0	0	0	0	0	17	0	0	337	0	0	0	0	0	0	3689.25	0	0	0	0	94.25	0	0	0	0	137	0	23		1.77
1339 FENEYDVIYLKP LAG	1925.75	201.5	0	596.5	0	18	0	0	0	0	0	0	0	0	0	0	0	0	0	9149.5	0	0	0	0	39	0	0	0	0	42.5	0	0		-0.45
1340 ENEYDVIYLKP LAGV	3643	0	0	5558.75	0	9	0	0	0	52.25	0	0	61.5	0	0	0	83	0	0	3716.75	0	0	0	0	0	0	0	0	0	379	0	0		2.01
1341 NEYDVIYLKPLAGVY	0	0	0	0	0	9.5	0	0	0	0	0	0	0	0	0	0	0	0	0	4950.75	0	0	0	0	0	0	0	0	0	0	0	0		-0.41
1342 EYDVIYLKPLAGVYR	0	0	21	0	0	4.5	0	0	0	112	0	0	0	69.5	0	0	0	0	0	0	0	0	0	0	0	0	0	0	0	0	0	0		-0.51
1343 YDVIYLKPLAGVYRS	0	0	0	0	0	0	0	0	0	0	0	0	0	0	0	0	0	0	0	0	0	0	0	0	0	0	0	0	0	0	0	0		
1344 DVIYLKPLAGVYRSL	0	0	0	0	0	0	0	0	0	0	0	0	0	0	0	0	0	0	0	0	0	0	0	0	0	0	0	0	0	0	0	0		
1345 VIYLKPLAGVYRSLK	0	0	0	0	0	0	0	0	0	0	0	0	0	0	0	24	0	0	0	0	0	0	0	0	0	0	0	0	0	0	0	0		-0.41
1346 IYLKPLAGVYRSLKK	0	0	0	0	0	26	0	0	0	0	0	0	0	0	0	236.5	0	28	0	0	0	5.75	0	0	0	0	0	164	0	45.5	0	0		-0.27
1347 YLKPLAGVYRSLKKQ	0	0	0	93	0	0	0	0	0	0	0	0	0	0	0	120.5	0	0	0	0	0	0	0	0	0	0	0	0	0	0	0	0		0.05
1348 LKPLAGVYRSLKKQI	0	0	0	31.25	0	0	0	0	0	0	0	0	0	0	0	0	0	0	0	0	0	0	0	0	0	53.5	0	0	0	0	0	0		-0.15
1349 KPLAGVYRSLKKQIE	1526.5	0	0	0	0	0	0	0	0	0	0	0	0	0	0	0	0	0	0	0	0	0	0	0	0	255	0	0	0	0	0	0		1.25
1350 PLAGVYRSLKKQIEK	807	0	0	0	0	0	0	0	0	0	0	0	0	0	35	0	0	0	0	0	0	0	0	0	0	0	0	0	0	0	0	0		1.35
1351 LAGVYRSLKKQIEKN	0	0	0	0	0	0	0	0	0	0	0	0	0	0	0	0	0	0	0	0	0	0	0	0	0	0	0	0	0	0	0	0		
1352 AGVYRSLKKQIEKNI	0	0	0	1595	0	0	0	0	0	0	0	0	0	0	0	0	0	0	0	235	0	0	0	0	0	0	0	0	0	0	0	0		1.27
1353 GVYRSLKKQIEKNIF	0	0	0	0	0	0	0	0	0	0	0	0	0	0	0	0	0	0	0	0	0	0	0	0	0	0	0	0	0	0	0	0		
1354 VYRSLKKQIEKNIFT	0	0	0	0	0	0	0	0	0	0	0	0	0	0	0	0	0	0	0	0	0	0	0	0	0	0	0	0	0	0	0	0		
1355 YRSLKKQIEKNIFTF	0	0	0	0	0	0	0	0	0	0	0	0	0	0	0	0	0	0	0	0	0	0	0	0	0	0	0	0	0	0	0	0		
1356 RSLKKQIEKNIFTFN	0	0	0	0	0	0	0	0	0	0	0	0	0	0	0	0	0	0	0	0	0	0	0	0	0	0	0	0	0	0	0	0		
1357 SLKKQIEKNIFTFNL	0	0	0	0	0	0	0	0	0	0	0	0	0	0	0	0	0	0	0	0	0	0	0	0	0	0	0	0	0	0	0	0		
1358 LKKQIEKNIFTFNLN	0	0	0	0	0	0	0	0	0	0	0	0	0	0	0	0	0	0	0	0	0	0	0	0	0	0	0	0	0	0	0	0		
1359 KKQIEKNIFTFNLNL	0	0	0	0	0	0	0	0	0	0	0	0	0	0	0	0	0	0	0	0	0	0	0	0	0	173.5	0	0	0	0	0	0		-0.41
1360 KQIEKNIFTFNLNLN	2165.5	0	0	873	0	0	0	0	0	0	0	0	0	0	0	0	0	0	0	0	0	0	0	0	0	0	0	0	0	0	0	0		2.81
1361 QIEKNIFTFNLNLND	2000.25	0	0	709.5	0	0	0	0	0	0	0	0	0	0	0	0	0	0	0	0	0	0	0	0	0	0	0	0	0	12.5	0	0		2.66
1362 IEKNIFTFNLNLNDI	327	0	453.75	650.5	0	0	0	0	0	0	0	0	0	584	0	0	0	0	0	0	0	0	0	0	0	0	0	0	0	0	0	0		3.03
1363 EKNIFTFNLNLNDIL	0	0	0	2906.5	0	0	0	0	0	0	0	0	0	147	0	0	0	0	0	2348.5	0	0	0	0	0	0	0	0	0	0	0	0		0.44
1364 KNIFTFNLNLNDILN	0	617.5	0	198.5	0	0	0	0	0	0	0	0	0	0	0	0	0	0	0	641.75	0	0	0	0	0	75	0	0	0	0	0	0		0.71
1365 NIFTFNLNLNDILNS	510.25	154.75	0	0	0	0	0	0	0	0	0	0	0	2826	0	0	0	0	0	0	0	0	0	0	0	0	0	0	0	0	117.5	0		-0.52
1366 IFTFNLNLNDILNSR	119.5	0	0	392.5	0	0	0	0	0	0	0	0	0	776.5	0	0	0	0	0	0	0	0	0	0	0	0	0	0	0	0	342	0		-0.16
1367 FTFNLNLNDILNSRL	0	0	0	0	0	0	0	0	0	0	0	0	0	0	0	0	0	0	0	0	0	0	0	0	0	0	0	0	0	0	0	0		
1368 TFNLNLNDILNSRLK	0	0	0	0	0	0	0	0	0	0	0	0	0	0	0	56.5	0	0	0	0	0	0	0	0	0	0	0	0	0	0	0	0		-0.41
1369 FNLNLNDILNSRLKK	0	0	0	0	0	0	0	0	0	0	0	0	0	0	0	289	0	0	0	0	0	0	0	0	0	0	0	0	0	0	0	0		-0.41
1370 NLNLNDILNSRLKKR	305.5	0	989.25	416.25	0	0	0	0	0	0	0	0	0	0	0	150.5	0	0	0	0	0	0	0	31	0	0	0	0	0	0	0	0		4.38
1371 LNLNDILNSRLKKRK	0	0	731.5	707	31	22.5	0	663	0	0	0	20.5	135.5	98.25	0	61.5	0	241.5	0	0	0	0	0	722	0	0	229.5	0	0	15.25	0	0		1.33
1372 NLNDILNSRLKKRKY	0	0	1859	593	0	14.5	0	390	0	0	0	17	0	0	0	0	0	250.75	0	0	0	0	0	0	0	0	0	0	0	0	6	0		2.41
1373 LNDILNSRLKKRKYF	0	0	0	547.75	0	0	0	0	0	0	0	0	0	0	0	157.5	0	0	0	0	0	0	0	0	0	0	0	0	0	0	0	0		1.12
1374 NDILNSRLKKRKYFL	0	0	0	0	0	0	0	0	0	0	0	0	0	0	0	45	0	0	0	0	0	0	0	0	0	0	0	0	0	0	0	0		-0.41
1375 DILNSRLKKRKYFLD	0	0	0	0	0	0	0	0	0	0	0	0	0	6	0	0	214.75	0	0	0	0	0	0	0	0	0	0	0	0	0	0	0		-0.41
1376 ILNSRLKKRKYFLDV	0	13	0	0	0	0	0	0	0	0	0	0	0	0	0	0	0	0	0	0	0	0	0	0	0	0	0	0	0	0	0	0		1.37
1377 LNSRLKKRKYFLDVL	0	0	0	0	0	0	0	0	0	0	0	0	0	0	0	0	0	0	0	0	0	0	0	0	0	0	0	0	0	0	0	0		
1378 NSRLKKRKYFLDVLE	119	0	30	3000	0	0	0	0	0	0	0	3	0	0	0	0	0	142.5	0	0	0	0	0	0	0	28.75	0	0	0	0	0	0		1.53
1379 SRLKKRKYFLDVLES	224	0	0	0	0	0	0	0	0	0	0	0	0	0	0	0	0	14.25	0	0	0	0	0	0	0	0	0	0	0	0	0	0		1.34
1380 RLKKRKYFLDVLESD	192.5	0	0	0	0	0	0	0	0	0	0	0	0	0	0	0	0	0	0	0	0	0	0	0	29	0	0	0	0	0	0	0		1.27
1381 LKKRKYFLDVLESDL	192	0	169.5	0	0	0	0	0	0	0	0	0	0	259	10	0	0	0	2	0	0	0	0	15	0	0	0	0	0	358.75	0	0		0.12
1382 KKRKYFLDVLESDLM	0	0	0	0	0	2.75	0	0	0	0	0	0	0	143	0	0	0	0	0	0	10.5	0	0	0	0	474.5	7	0	0	0	0	0		-0.35
1383 KRKYFLDVLESDLMQ	0	0	0	0	3.5	0	0	0	0	0	0	0	0	0	0	0	0	0	0	0	0	0	0	0	0	195	0	0	0	0	0	0		-0.41
1384 RKYFLDVLESDLMQF	176	0	0	398.5	9.25	0	26.5	0	39.5	0	6.5	8	0	99	6.5	30	131.5	0	9.5	81.5	65	6	0	0	9	12.75	0	0	0	10.5	0	20.5		1.76
1385 KYFLDVLESDLMQFK	92.25	0	0	0	0	0	0	0	0	0	0	0	0	0	0	0	0	0	0	0	0	0	0	0	0	745	0	0	0	0	0	0		-0.54
1386 YFLDVLESDLMQFKH	103	0	0	88.5	0	3	0	0	0	0	0	0	0	23.5	0	0	0	0	0	22.5	0	0	6	0	0	0	0	0	0	0	0	0		3.06
1387 FLDVLESDLMQFKHI	0	0	0	106.5	0	6.5	0	0	0	0	0	0	0	0	6	0	0	0	12	140.5	5	0	0	0	0	0	0	0	0	0	0	0		0.00
1388 LDVLESDLMQFKHIS	0	0	0	0	0	0	0	0	0	0	0	0	0	0	0	36.5	0	0	0	23.5	0	0	0	0	0	0	0	0	0	0	0	0		-0.33
1389 DVLESDLMQFKHISS	45.5	0	0	0	0	0	0	0	0	0	0	0	0	0	0	0	0	0	0	0	0	0	0	0	0	0	0	0	0	0	0	0		1.37
1390 VLESDLMQFKHISSN	0	0	0	0	0	26	0	0	0	0	0	0	0	0	0	75.5	0	0	0	691.25	0	0	0	0	0	0	0	0	0	0	0	0		-0.39
1391 LESDLMQFKHISSNE	161.5	792	0	1653	23.5	0	0	0	0	0	0	0	0	0	0	0	0	0	0	632	23	0	0	0	0	0	22.25	0	0	0	0	0		2.86
1392 ESDLMQFKHISSNEY	0	34	0	1635.5	43.25	0	0	0	17.5	84	4.5	0	583	144.5	30.5	84	62.5	79	62	523.5	439	0	22	0	0	0	24.5	0	0	25	3.5	2		0.59
1393 SDLMQFKHISSNEYI	0	0	0	0	18	0	0	0	0	0	0	0	0	0	0	0	0	0	0	0	1508.75	0	0	0	0	0	0	0	0	0	0	0		-0.41
1394 DLMQFKHISSNEYII	0	431.5	0	0	5.5	0	0	0	0	0	0	0	0	0	0	192.5	0	0	0	0	501.5	0	0	0	0	0	0	0	0	0	0	0		0.02
1395 LMQFKHISSNEYIIE	0	468	1646.5	0	31.5	3.5	0	0	0	0	0	16.5	0	0	0	381.5	0	0	0	0	338.5	0	0	0	0	0	0	0	0	0	0	0		1.96
1396 MQFKHISSNEYIIED	9210	429.75	530	0	0	6	16	0	0	0	0	0	0	0	20.5	165	0	0	35	0	0	12	0	0	0	0	92.5	55	22.5	0	0	0	16	1.76
1397 QFKHISSNEYIIED S	5891	10059.5	0	0	0	0	0	0	0	0	0	0	0	0	0	122	0	0	0	0	0	0	0	0	0	0	148	0	0	0	0	0		3.23
1398 FKHISSNEYIIED SF	0	22370.25	286	1282.5	26	0	29	0	141.5	0	0	2	0	0	0	345.75	142.5	190	67.25	0	17.5	0	6.5	0	0	0	81.25	0	0	0	0	69		1.62
1399 KHISSNEYIIED SFK	0	11337	64.25	0	0	0	0	0	0	0	0	0	0	0	0	0	0	0	0	0	0	0	0	0	0	143.5	0	0	0	0	0	0		1.39
1400 HISSNEYIIED SFKL	0	11934.75	0	0	19.5	0	0	7.5	0	0	0	0	0	0	0	109	87.5	0	0	0	0	0	129	10	0	0	0	0	0	0	0	0		1.36
1401 ISSNEYIIED SFKLL	0	11075	0	290	198	0	0	0	0	0	0	0	0	0	0	51	0	0	0	248.5	0	0	587	0	0	0	0	0	1047.5	0	0	0		1.37
1402 SSNEYIIEDSFKLLN	0	2015.5	0	0	0	7.5	0	0	0	0	0	0	0	0	0	0	0	0	0	967.25	0	0	0	0	0	0	0	0	0	0	0	0		0.86
1403 SNEYIIEDSFKLLNS	0	1873.5	0	0	47.5	0	0	0	0	0	0	0	0	307	0	0	0	0	0	0	0	0	0	0	0	0	0	0	0	0	0	0		1.25
1404 NEYIIEDSFKLLNSE	191.5	4896.5	0	0	0	19	0	0	0	0	0	0	0	0	0	0	0	0	0	0	0	0	0	0	0	0	33	27	0	0	0	0		1.51
1405 EYIIEDSFKLLNSEQ	0	0	536	0	29	6	0	0	0	0	0	0	0	0	0	0	0	0	0	0	0	0	0	0	0	0	0	0	0	0	0	0		1.37
1406 YIIEDSFKLLNSEQK	0	456.5	0	0	0	0	0	0	0	0	0	0	0	0	0	0	0	0	0	0	0	0	0	0	0	0	0	913.25	0	0	0	0		-0.24
1407 IIEDSFKLLNSEQKN	0	0	0	0	0	0	0	0	0	0	0	0	0	0	0	0	0	0	0	0	0	0	0	0	0	0	0	0	0	0	0	0		
1408 IEDSFKLLNSEQKNT	0	0	0	0	0	0	0	0	0	0	0	0	0	0	0	0	0	0	0	0	0	0	0	0	0	0	0	0	0	0	0	0		
1409 EDSFKLLNSEQKNTL	0	0	0	0	0	0	0	0	0	0	0	0	0	0	0	0	0	0	0	0	0	0	0	0	0	0	0	0	0	0	0	0		
1410 DSFKLLNSEQKNTLL	0	0	0	0	0	0	0	0	0	0	0	0	0	0	0	0	0	0	0	0	0	0	0	0	0	0	0	0	0	0	0	0		
1411 SFKLLNSEQKNTLLK	0	0	0	0	0	0	0	0	0	0	0	0	0	0	0	0	0	0	0	234	0	0	0	0	0	0	0	0	0	0	0	0		-0.41
1412 FKLLNSEQKNTLLKS	0	0	0	157.5	0	0	0	0	0	0	0	0	0	0	0	0	0	0	0	1045.25	0	0	0	0	0	0	0	0	0	0	0	0		-0.56
1413 KLLNSEQKNTLLKSY	0	0	0	0	0	0	0	0	0	0	0	0	0	11.5	0	0	0	0	0	201.25	0	0	0	0	0	194.5	0	0	0	0	0	433.5		-0.27
1414 LLNSEQKNTLLKSYK	0	0	0	0	0	0	0	0	0	0	0	32	0	0	0	20.5	0	0	0	2275	0	0	79	0	0	0	31.5	15	0	0	0	132		-0.39
1415 LNSEQKNTLLKSYKY	0	0	0	0	0	0	0	0	0	0	0	43.5	0	0	0	0	0	0	0	288.5	0	0	0	0	0	0	0	0	0	0	0	0		-0.38
1416 NSEQKNTLLKSYKYI	0	0	0	161	0	0	0	0	0	0	0	17	0	0	0	0	0	0	0	0	0	0	0	0	0	152.5	0	0	0	0	0	0		0.29
1417 SEQKNTLLKSYKYIK	0	0	0	0	0	0	0	0	0	0	0	0	0	0	0	0	0	0	0	271	0	0	0	0	0	0	0	0	0	0	0	0		-0.41
1418 EQKNTLLKSYKYIKE	359	0	0	0	0	0	0	0	0	8	0	0	146.5	67.5	0	0	0	0	0	154	0	0	0	0	0	0	0	0	0	0	0	0		0.56
1419 QKNTLLKSYKYIKES	0	0	0	0	0	0	0	0	0	0	0	0	0	0	0	0	0	0	0	122.75	0	45	0	0	0	0	0	0	0	0	0	0		-0.35
1420 KNTLLKSYKYIKESV	0	0	241.5	868.75	0	0	0	0	0	0	0	0	0	0	0	0	0	0	0	533.5	0	0	0	0	0	402.75	0	0	0	0	0	0		1.01
1421 NTLLKSYKYIKESVE	1111.5	0	0	0	0	0	0	0	0	0	0	30.5	135	0	0	0	0	0	0	0	0	0	0	0	0	1854.5	0	0	162	0	0	36.5		-0.18
1422 TLLKSYKYIKESVEN	1037	50.5	405	1330.25	0	0	0	0	0	0	0	0	0	0	0	0	0	0	0	0	0	0	0	0	0	0	0	0	0	0	0	0		5.70
1423 LLKSYKYIKESVEN D	591.5	0	276	3738	0	0	0	0	0	0	0	0	0	0	0	31	294.5	34.5	0	171	0	5.5	0	0	0	0	0	30	0	0	0	0		2.21
1424 LKSYKYIKESVEN DI	237	0	77	4615	0	0	0	0	14	0	0	0	12.5	0	0	0	193	1680.5	0	0	0	12.5	16.25	6	0	0	0	0	0	0	0	4		1.21
1425 KSYKYIKESVENDIK	0	472	0	0	26	0	0	0	0	0	0	0	0	0	0	0	0	480.25	0	0	172	0	0	0	0	333.5	0	0	301.5	0	0	0		-0.20
1426 SYKYIKESVENDIKF	811	0	0	725.5	17.5	0	0	0	0	0	0	0	0	0	0	30	757	80	0	479.5	256.5	13.5	0	34.5	0	0	0	0	0	0	0	0		1.20
1427 YKYIKESVENDIKFA	0	0	0	0	0	0	0	0	0	0	0	0	0	0	493.5	131.5	0	246.5	1805.5	426.5	0	0	0	24.5	0	0	0	0	130.25	0	0	0		-0.26
1428 KYIKESVENDIKFAQ	0	0	0	0	0	0	0	0	0	0	0	0	0	0	49	0	91.5	0	332.75	58.75	0	0	0	0	0	181	0	0	30	0	0	0		-0.21
1429 YIKESVENDIKFAQE	655	0	0	523.25	24	11.5	13.5	0	35.5	0	0	0	70.5	0	1494.25	77	446.25	403.5	2185.25	54	1224.25	62	0	27.75	0	0	100.5	31	187.5	0	0	7.5		-0.54
1430 IKESVENDIKFAQEG	323	0	0	364.5	0	9.5	0	0	0	11	40	0	300.25	0	91.75	0	187.5	160	298.75	0	189	45	0	0	0	0	7	0	199	0	0	0		0.45
1431 KESVENDIKFAQEGI	478.5	0	0	0	1.5	0	0	0	0	61	0	0	923.5	0	114	0	0	278.5	289	0	0	173.5	0	0	0	0	0	0	0	0	0	0		-0.42
1432 ESVENDIKFAQEGIS	0	0	0	0	7	0	0	0	0	195	0	0	698	248.5	49.5	56.5	0	247.25	5020	0	1703.5	0	30.5	5.5	0	0	0	15.5	0	11.5	0	0		-0.29
1433 SVENDIKFAQEGISY	256	160.5	47.5	1672.5	0	0	0	0	0	0	0	0	0	0	309	34	135	241.5	2648	125.5	923	0	0	0	0	0	0	0	0	0	0	0		-0.02
1434 VENDIKFAQEGISY Y	271	0	147.75	655	0	10	33.5	0	0	0	0	0	0	0	60.25	19	37	157.5	1824	215.5	553.75	0	0	0	0	0	0	0	0	0	0	0		-0.24
1435 ENDIKFAQEGI SYYE	355.5	231.25	463	2567.25	54	30	79	12	114	152.5	73.5	10.5	295.5	285.5	111	105.5	187	356	0	246	501.5	29.5	41	22.5	0	0	36	127	0	157.5	29.5	41.5		2.36
1436 NDIKFAQEGISYYEK	0	0	0	0	10	0	0	0	0	0	0	42.5	0	0	52.75	0	0	0	0	0	0	0	0	0	0	269.5	0	0	0	0	0	0		-0.35
1437 DIKFAQEGISYYEKV	1484.25	5390	0	0	0	0	0	12	0	0	0	0	0	0	401	0	0	0	0	0	0	0	0	0	0	0	0	0	0	0	0	28		2.34
1438 IKFAQEGISYYEKV L	1096	5490	0	0	0	0	0	0	0	0	0	0	0	0	0	0	0	0	0	0	0	0	0	0	0	0	0	0	0	0	0	0		2.13
1439 KFAQEGISYYEKV LA	269.25	2640	0	0	0	0	0	0	0	0	0	0	0	0	0	0	0	0	0	0	0	0	0	0	0	172	0	0	0	0	0	0		1.72
1440 FAQEGISYYEKV LAK	218.75	2570	0	0	0	0	0	0	0	0	0	0	0	0	0	0	0	0	0	0	0	0	0	0	0	0	0	0	0	0	0	0		1.69
1441 AQEGISYYEKVLAKY	0	181.5	0	0	0	0	0	0	0	0	0	0	0	0	0	0	0	0	0	0	0	0	0	0	0	0	0	0	0	0	0	0		1.37
1442 QEGISYYEKVLAKYK	0	0	0	0	0	0	0	0	0	7	0	0	0	0	0	0	0	0	0	0	0	0	0	0	0	0	0	0	0	0	0	0		-0.41
1443 EGISYYEKVLAKYKD	0	0	0	0	0	0	0	0	0	386.75	0	0	106.5	169.5	55	0	0	145.5	137.5	0	0	0	28.5	0	0	0	0	0	0	0	0	15		-0.12
1444 GISYYEKVLAKYKDD	0	0	0	0	0	0	0	0	129.5	0	0	0	0	0	0	0	0	0	372.75	0	0	0	25.25	0	0	0	9.5	31	0	66.5	0	18.5		-0.28
1445 ISYYEKVLAKYKDDL	0	0	0	0	0	0	0	0	0	0	0	0	0	0	0	0	845	0	0	0	0	0	0	0	0	0	0	0	0	0	0	0		-0.41
1446 SYYEKVLAKYKDDLE	0	8	0	7114.25	0	8.5	0	0	0	14.75	42.75	0	0	0	0	0	89	0	0	0	0	0	14	0	5.5	0	15	79.5	0	0	0	0		1.36
1447 YYEKVLAKYKDDLES	0	0	0	91.5	0	4.5	0	0	0	0	0	0	0	0	0	0	0	0	0	0	0	15	0	0	0	0	0	0	27	0	0	0		1.01
1448 YEKVLAKYKDDLESI	204.5	122.5	0	0	0	0	0	0	0	0	0	37	0	0	0	0	324.75	0	0	33.5	18	0	0	0	0	0	0	0	19	0	0	0		0.39
1449 EKVLAKYKDDLESIK	0	0	0	0	0	0	0	0	0	5.5	0	0	0	86.5	0	0	1006	0	0	73.25	0	0	0	0	0	0	0	0	0	785	0	0		-0.30
1450 KVLAKYKDDLESIKK	0	0	0	0	0	0	0	0	0	0	0	0	0	0	0	0	1558	0	0	0	0	0	0	16	0	1148.75	0	0	0	985.5	0	0		-0.25
1451 VLAKYKDDLESIKKV	0	0	0	0	0	0	0	0	0	0	0	0	0	0	0	0	2074.75	0	0	0	0	0	0	2	0	0	0	0	0	900.5	0	0		-0.35
1452 LAKYKDDLESIKKVI	642.5	60.5	0	0	0	0	0	16	0	0	0	8.25	0	0	0	0	1772.75	0	0	0	29	0	0	0	0	0	0	0	0	868	0	0		-0.45
1453 AKYKDDLESIKKVIK	748.75	237.5	0	0	0	0	0	0	0	0	0	0	0	0	0	24.5	1317.5	0	0	0	56.5	0	0	0	0	326	0	0	0	645.75	0	0		-0.15
1454 KYKDDLESIKKVIKE	1126.75	53	0	157.5	0	0	0	0	0	0	0	0	0	0	0	0	1200	0	0	0	77.75	91.5	0	0	0	830.5	0	0	0	250.5	0	0		0.12
1455 YKDDLESIKKVIKEE	348.25	0	70.5	431.25	0	10	0	0	0	0	195.5	0	268.5	18	53	0	0	0	0	0	0	399	0	0	0	0	0	0	0	183	7	0		1.17
1456 KDDLESIKKVIKEEK	0	0	0	0	0	0	0	0	0	0	0	0	0	0	6.5	0	0	0	0	0	0	0	0	0	0	111.5	0	0	0	48.5	0	0		-0.34
1457 DDLESIKKVIKEEKE	90.5	0	0	0	0	16	0	0	14	0	0	0	171.75	0	0	0	0	0	22	0	0	57	0	8	0	0	0	166.5	0	59.5	0	0		-0.55

1458	DLESIKKVIKEEEKEK	0	0	0	0	0	9.5	0	0	0	0	0	0	0	0	0	0	0	0	0	0	0	197.5	0	0	0	0	0	0	0	0	0	0	-0.41
1459	LESIKKVIKEEEKEKF	0	990.75	0	0	0	0	0	0	0	0	0	15	397.5	43.5	0	0	0	0	0	0	0	0	0	0	0	0	0	0	0	0	0	0	0.95
1460	ESIKKVIKEEEKEKFP	0	7203	0	0	0	0	0	0	0	0	30	0	409	114.5	0	29	0	0	0	412	0	0	0	0	0	0	0	0	0	0	0	0	1.30
1461	SIKKVIKEEEKEKFPS	0	11072.5	0	0	0	0	0	0	0	0	12.75	0	296.5	0	0	0	114	0	0	370	0	0	0	0	0	0	0	0	0	0	0	272.5	1.32
1462	IKKVIKEEEKEKFPSS	0	14071	0	407.75	0	0	0	0	0	0	0	0	349.5	0	0	0	89	0	0	341.75	0	0	0	0	0	195.5	68.5	0	0	0	0	0	1.44
1463	KKVIKEEEKEKFPSSP	0	19184.5	0	0	0	0	0	0	0	0	0	0	876.5	0	0	0	142.5	0	0	386.5	0	0	0	31	0	965	0	0	0	0	0	392	1.30
1464	KVIKEEEKEKFPSSPP	0	16998.5	42.5	0	0	0	0	0	96.5	0	50	0	527.5	0	0	0	220.5	0	0	0	0	0	0	0	0	371	0	0	0	195.5	0	0	1.34
1465	VIKEEEKEKFPSSPPT	0	15873.5	0	306.5	0	0	0	0	5155.25	0	671	1367.5	3621	2097.75	2212	787.5	0	10651.5	699.5	9042.75	872.5	497.25	0	1087	0	0	291.5	158.5	1187.5	3713.5	5967.25	1645.75	-0.14
1466	IKEEEKEKFPSSPPTT	0	25997.5	0	8166	0	0	0	0	7366	966.5	1245	3318.25	6935.25	3576.5	4150.5	1272.5	1809.5	15143.5	895	11881.25	1241.5	1337	726.75	2647.75	151.5	1732	634.5	111.25	1561	9048	10660.5	3754	0.28
1467	KEEEKEKFPSSPPTTP	1025.5	21856.5	0	2643.75	0	57	0	0	22195.5	3745.5	8888.75	9167.25	16140.5	13207.75	11970	2553.25	3115.5	21400.75	4114.75	21782.25	2967.5	1291.5	1107	6855.75	1466.75	4276.25	1683.5	520.5	5368.5	14384.5	21341	9694.5	-0.34
1468	EEKEKFPSSPPTTPP	1309.25	15525.75	0	885.5	0	191	0	0	63558.25	11063.25	15172.25	13820.5	37213.25	28321.5	11675	3100	3040.25	24249.25	9039.75	33109.25	4481.5	3990.5	3807	13219	3594	9415	2895.5	1626.5	7659.25	23975.5	33570	22656	0.26
1469	EKEKFPSSPPTTPPS	1289.5	9728.75	0	25034.5	0	0	0	0	42103	10355.75	19081	16868.5	30068.25	25515	12099	3646.5	4075.75	25224	9582	33700	4798	3364	3384.5	11806.5	3302.75	8884.25	2381.5	1543	9042	32937.5	40646.25	18857.5	-0.11
1470	KEKFPSSPPTTPPSP	6071	8539.75	0	3367.5	0	6.5	0	7	20774	5230.75	13434.5	19066	20856.75	18592	8577.25	3901	4463.75	26167	10576.5	33433.75	4699	2767.25	2310.5	8864.5	2804	5654	2153.75	963.25	7244	34772.5	31495	9336.5	0.23
1471	EKFPSSPPTTPPSPA	2964	6364	0	5219.75	0	0	0	0	19634.5	3243.5	9458.5	17077.5	9636.25	6128.75	7538.25	3599.75	3584.5	22082.5	7461.25	30773.5	4390.75	2305.5	1405	3641.5	1210.25	3868.5	1398.5	662	4167.25	27323.75	20231.5	7886.5	0.06
1472	KFPSSPPTTPPSPAK	10025.5	4009.25	63977	3445.25	0	0	0	0	26411	4109	16148	18451.75	34640	22493.25	16840.75	4750	5344.5	28485	9059.5	36348.75	5423.25	4111.75	1815	12851	3914	4685	1380.5	692.5	6986.5	37776.75	23527	11919.5	-0.22
1473	FPSSPPTTPPSPAKT	8906.5	3624.75	63977	1416.75	0	0	0	0	539	0	366.5	382	606.25	0	189	85.5	160.75	4784.5	197.25	2798.5	606.5	45.5	0	310.5	0	0	0	0	0	797	950	360	2.13
1474	PSSPPTTPPSPAKTD	8724	4373	63977	2429.5	0	0	0	0	143.5	0	0	0	175	0	43	277.25	0	5031.75	295	2006.25	518	0	0	0	0	0	0	0	0	198	166.5	159	2.29
1475	SSPPTTPPSPAKTDE	4914	2849	63977	6852.25	0	0	0	0	0	0	0	0	0	169.5	0	0	0	0	0	0	0	672.25	0	0	0	0	0	14.5	0	0	0	16.75	2.32
1476	SPPTTPPSPAKTDEQ	3916.5	3801.5	63977	6062.75	0	0	0	0	0	0	0	0	0	0	0	10256	0	0	0	612.75	646.5	520.5	0	0	0	0	0	0	0	0	0	50	2.10
1477	PPTTPPSPAKTDEQK	2199.25	2519.5	63977	697.5	0	0	0	0	0	0	0	0	0	0	0	11404	0	12090.25	0	333.5	487	376.5	0	0	25	0	0	0	0	0	0	0	1.39
1478	PTTPPSPAKTDEQKK	425.75	1167.25	63977	0	0	0	0	0	0	0	0	0	0	0	0	11860	4056	6112	0	0	98.25	180.5	0	0	0	0	0	0	0	0	0	0	1.23
1479	TTPPSPAKTDEQKKE	283.5	0	32978.75	976	0	0	0	0	0	0	0	0	0	0	0	11346.5	7876	7069.25	0	0	389.5	88.5	0	0	0	0	0	0	0	0	0	0	0.87
1480	TPPSPAKTDEQKKES	0	0	63977	0	0	0	0	0	0	0	0	0	0	0	0	9772.5	7881.25	5994	0	0	292.5	283.5	0	0	0	0	0	0	0	0	0	0	1.13
1481	PPSPAKTDEQKKESK	303.25	0	28840	0	0	0	0	0	0	0	0	0	0	0	0	12950	6319	7060.25	0	0	0	41.5	0	0	0	0	0	0	0	0	0	0	0.68
1482	PSPAKTDEQKKESKF	0	0	24834.75	0	1.5	0	0	0	0	0	0	0	0	0	0	11748.5	8287.25	8547	0	168.25	322	35.5	0	0	0	0	0	0	0	0	0	0	0.48
1483	SPAKTDEQKKESKFL	174.25	226	19184.5	956	0	0	0	0	0	0	0	0	0	0	0	11353	7896.5	5305.25	0	0	352.5	0	0	0	0	0	0	0	0	0	0	0	0.51
1484	PAKTDEQKKESKFLP	0	0	4563.25	0	0	0	0	0	0	0	0	202.5	0	0	0	13757	5702	5050	0	0	96.5	19	0	0	0	0	0	0	0	0	0	0	-0.55
1485	AKTDEQKKESKFLPF	0	0	0	97.5	0	0	0	0	0	0	0	0	0	0	0	9169.5	1861.75	854.75	0	0	60.5	0	0	16	0	0	0	27	0	155.5	0	2	-0.36
1486	KTDEQKKESKFLPFL	0	53	0	42	0	0	0	0	0	7	107	407.5	127	0	63	10115	1501.5	314	0	431.5	187.5	92.5	7	72	0	240	0	0	240.5	151	329	71.25	-0.34
1487	TDEQKKESKFLPFLT	0	0	0	0	0	0	0	0	0	0	28	286.75	137	0	24.5	0	0	358.5	248.5	0	0	39.5	37	0	0	0	0	0	144.75	149.5	194	0	0.09
1488	DEQKKESKFLPFLTN	0	0	0	0	0	0	0	0	0	0	0	189	134.5	0	22	0	0	321.25	190	487	77	22	0	0	0	139.5	8	0	146.25	94.5	147.5	66	0.17
1489	EQKKESKFLPFLTNI	0	0	0	445.75	0	0	0	0	0	0	0	134	201.5	0	23	0	0	438.75	254.5	0	0	0	0	0	0	0	0	0	66	104	127.75	0	-0.20
1490	QKKESKFLPFLTNIE	2562	199	0	0	0	0	0	0	0	0	12	185.5	154	0	0	0	689	505.5	223.5	552	88.5	29.5	0	0	0	0	0	0	156.5	134.5	0	74	0.95
1491	KKESKFLPFLTNIET	12527	0	324	0	0	0	0	0	63.5	0	73.5	196.5	175.5	0	18	0	4014.5	356.5	320.5	466.25	0	277.75	0	0	34.5	328	150.5	0	357.75	76.5	93	87.25	1.06
1492	KESKFLPFLTNIETL	11492.75	180	0	0	0	0	0	0	0	20	60.5	88	248	0	29	0	0	167.5	264.5	554.75	48	209.5	0	0	0	158.5	168	0	184.75	38.5	208.5	44.5	1.32
1493	ESKFLPFLTNIETLY	3724.5	0	0	349.5	0	4	0	0	719	37	0	143.75	353.5	122.5	13.75	0	439	92	112.5	313.5	0	0	20	75	0	0	79.75	0	136	98.25	86.25	30	1.26
1494	SKFLPFLTNIETLYN	0	0	0	0	0	0	0	0	0	0	0	0	0	0	0	0	0	78.25	0	76.25	0	0	0	0	0	269.5	0	0	353	0	0	0	-0.26
1495	KFLPFLTNIETLYNN	198	0	0	0	0	0	0	0	0	0	0	0	0	0	0	0	0	0	0	0	0	0	0	0	0	74	17.25	0	21.5	0	0	0	0.91
1496	FLPFLTNIETLYNNL	0	0	0	0	0	0	0	0	0	0	0	0	0	0	0	0	0	0	0	0	0	0	0	0	0	0	24.5	0	0	0	25.5	0	-0.33
1497	LPFLTNIETLYNNLV	0	0	0	217	0	0	0	0	0	0	0	0	0	0	0	0	0	0	0	0	0	0	0	0	0	0	0	0	0	0	0	0	1.37
1498	PFLTNIETLYNNLVN	0	0	0	324	0	0	0	0	0	0	0	0	0	0	0	0	0	0	0	0	0	0	0	0	0	0	0	227.5	90	0	0	0	0.46
1499	FLTNIETLYNNLVNK	0	0	0	0	0	0	0	0	0	0	0	0	0	0	0	0	0	0	0	0	0	0	0	0	0	0	0	0	0	0	0	0	
1500	LTNIETLYNNLVNKI	0	0	0	0	0	0	0	0	0	0	0	0	0	0	0	0	0	0	0	65	0	0	0	0	0	0	0	0	0	0	0	221	-0.36
1501	TNIETLYNNLVNKID	0	0	0	578.75	0	0	0	0	0	0	0	0	0	0	0	0	0	0	0	0	0	0	0	0	0	0	0	0	0	0	0	0	1.37
1502	NIETLYNNLVNKIDD	308	221.5	246.5	261.25	0	0	19	0	48	0	0	108	0	0	0	0	0	0	0	0	0	0	100	0	0	0	46.25	137.5	0	0	0	0	10.19
1503	IETLYNNLVNKIDDY	487	0	269.5	786.75	9	26.5	19.75	0	255	0	38	34.5	2342.25	0	0	0	202	91.5	0	194	0	0	193.75	0	0	0	0	0	1759	0	0	0	-0.33
1504	ETLYNNLVNKIDDYL	0	0	235	1323.25	0	0	0	0	180.5	19	4.5	0	255.5	134	0	0	220	0	0	112.25	0	0	0	0	0	0	0	0	0	0	0	0	1.51
1505	TLYNNLVNKIDDYLI	0	0	108	0	0	0	0	0	112.5	0	0	0	0	0	0	0	228.5	0	0	104.5	0	0	0	0	0	0	0	0	0	0	0	0	-0.46
1506	LYNNLVNKIDDYLIN	0	0	88	160.75	0	0	0	0	0	0	0	0	0	0	0	0	107	0	0	0	0	0	0	0	0	0	0	0	66.75	0	0	0	1.52
1507	YNNLVNKIDDYLINL	0	0	0	1122.5	0	0	0	0	0	0	8	0	0	0	0	0	504.5	0	0	0	0	0	0	0	0	0	0	0	0	0	0	0	0.90
1508	NNLVNKIDDYLINLK	0	0	0	0	0	0	0	0	0	0	0	73	0	0	0	0	355	0	0	0	0	0	0	0	0	0	30.5	0	0	0	0	0	-0.36
1509	NLVNKIDDYLINLKA	0	0	0	0	0	0	0	0	0	0	0	9	0	0	0	0	0	0	0	0	0	0	0	0	0	0	0	0	0	0	0	0	-0.41
1510	LVNKIDDYLINLKAK	0	0	0	0	0	0	0	0	0	0	0	0	0	0	0	138.5	0	0	0	0	0	0	0	0	0	0	0	0	0	0	0	0	-0.41
1511	VNKIDDYLINLKAKI	0	0	0	330	0	0	0	0	0	0	0	0	0	0	0	224	0	0	0	6	0	0	0	0	0	0	0	0	0	0	0	0	0.60
1512	NKIDDYLINLKAKIN	0	0	0	183	0	0	0	0	0	0	0	0	0	0	0	21.75	0	0	0	0	0	0	0	0	0	0	0	0	0	0	0	0	1.29
1513	KIDDYLINLKAKIND	0	33.5	0	0	0	0	0	13	6.5	19	0	0	0	0	12.5	150.5	101.5	0	94.25	1698	19	0	0	0	0	108	0	85	0	0	0	0	-0.37
1514	IDDYLINLKAKINDC	0	19	8.5	0	0	0	0	8	0	0	0	0	0	16.5	0	0	0	0	78	1635.75	26.5	3.5	0	20	0	0	0	0	80.5	0	0	0	-0.40
1515	DDYLINLKAKINDCN	0	0	0	265.25	0	0	0	0	0	0	0	0	0	0	0	0	0	0	0	1851.75	0	0	0	0	0	0	0	0	0	0	0	0	-0.55
1516	DYLINLKAKINDCNV	0	86.25	0	195.5	0	0	0	0	0	0	0	0	0	0	0	0	0	0	0	1771.5	0	0	0	0	0	0	0	0	0	0	0	0	-0.57
1517	YLINLKAKINDCNVE	1025	0	0	170.5	0	0	0	0	0	0	0	0	15.5	0	0	0	90	8.75	0	888	0	0	3.5	63.5	0	0	0	47.75	57.5	0	0	82	0.61
1518	LINLKAKINDCNVEK	318.5	0	0	0	0	0	0	0	0	0	0	0	0	0	931	0	0	0	0	479.5	0	0	0	0	0	0	0	0	0	0	0	0	-0.50
1519	INLKAKINDCNVEKD	149.5	0	530	311.5	0	0	0	0	0	0	0	0	0	0	89.5	0	0	0	46	1239.5	0	0	18	0	0	0	0	132	31.25	0	0	0	0.16
1520	NLKAKINDCNVEKDE	0	0	1021	836.5	0	10.5	2	0	7	4	0	39.25	0	0	54	35.5	0	0	95.5	1992.75	0	0	29	11.5	2.5	0	34.25	169.75	21	0	0	0	0.33
1521	LKAKINDCNVEKDEA	0	0	770.5	0	0	3.5	0	0	0	0	0	0	0	0	0	0	0	0	0	987.5	0	0	0	0	0	0	0	0	0	0	0	0	0.06
1522	KAKINDCNVEKDEAH	0	0	0	0	0	0	9	0	0	0	0	0	8.5	0	35.25	0	0	0	0	645.5	0	0	0	0	0	489.5	0	0	0	0	0	0	-0.32
1523	AKINDCNVEKDEAHV	153.5	0	0	0	0	0	0	0	0	0	0	0	0	0	0	0	0	0	0	0	0	0	0	0	0	21.25	0	0	0	0	0	0	1.28
1524	KINDCNVEKDEAHVK	0	0	0	194.5	0	0	0	0	0	0	0	0	0	0	0	0	0	210.75	714	0	0	0	0	0	0	593	0	0	0	0	0	0	-0.52
1525	INDCNVEKDEAHVKI	0	1416.25	0	776.25	0	0	0	0	0	0	0	0	0	0	14.5	0	0	236	1081.5	296.5	0	0	0	0	0	0	0	0	289	0	0	0	1.36
1526	NDCNVEKDEAHVKIT	1638	3249.75	1037.5	197.5	0	0	16	0	0	0	0	84	0	0	10	11	0	208.5	1706.5	394.25	8.75	0	0	0	0	0	0	0	0	0	0	0	3.69
1527	DCNVEKDEAHVKITK	10470	3134	0	0	0	0	0	0	0	0	0	0	0	0	0	162.75	0	232	3453	0	0	0	92.5	68.5	26	0	0	0	0	260.5	97	0	1.99
1528	CNVEKDEAHVKITKL	19341	4883	0	1875.5	0	0	0	0	0	0	0	177.5	0	0	0	256	0	362.75	3094	536	361.25	0	252.75	186.5	77.25	0	0	0	0	345.5	372.25	0	2.56
1529	NVEKDEAHVKITKLS	18215.75	3722	33.5	0	0	0	0	0	0	0	0	140.5	0	0	0	249.5	472	419.5	2926.5	870.25	217.5	0	0	174.5	128	0	0	0	0	303.5	789.75	0	1.89
1530	VEKDEAHVKITKLSD	19814.5	2362.25	248.75	0	0	0	0	0	0	59	8.5	87	358.75	0	0	236.5	0	583	3268.5	768.5	210.75	0	0	53.25	13	0	0	0	0	388.25	118	0	1.66
1531	EKDEAHVKITKLSDL	11692.5	3009.25	33.25	0	0	0	0	0	0	769.5	0	84.25	261.5	211.5	0	238	658.5	375	2966.5	1207.5	246	0	0	43.5	0	0	0	0	0	0	147.5	0	1.85
1532	KDEAHVKITKLSDLK	5639.75	12488	0	0	0	0	0	0	0	0	0	38.5	0	182	0	162	1079.25	271.5	2113.5	2440.5	0	0	0	70.5	123	551.5	0	0	0	0	553.75	320	2.41
1533	DEAHVKITKLSDLKA	4020.5	0	0	0	0	0	0	0	0	0	0	0	0	0	0	0	589	0	0	1605.5	0	0	0	0	0	0	0	0	0	0	0	0	0.90
1534	EAHVKITKLSDLKAI	4678.25	1570.5	0	0	0	0	0	0	0	0	0	0	0	288.5	11	0	484.5	0	0	4222.25	0	0	0	0	0	0	0	0	0	0	0	852	0.86
1535	AHVKITKLSDLKAID	2462.5	2877.25	0	0	0	0	0	44.5	0	0	0	0	0	0	0	277.75	608	0	1259	3669.25	78.5	0	0	0	0	0	0	0	0	0	0	0	0.89
1536	HVKITKLSDLKAIDD	882	8473	170.5	0	0	0	0	0	0	0	0	0	0	0	0	80	0	0	508	0	0	0	225.25	0	0	0	0	0	0	0	0	0	1.79
1537	VKITKLSDLKAIDDK	136.5	7894.25	0	0	2	0	0	0	0	0	0	0	234.75	0	0	408	0	762.75	980.25	558	0	0	207	0	0	0	0	0	0	4360	0	0	0.67
1538	KITKLSDLKAIDDKI	1422	9350.5	0	111.5	44.25	0	0	113.5	0	0	0	148.5	0	0	0	936.25	192	0	563	1144.5	0	259.75	445	88	127.5	188.5	0	0	977.5	7376	517	0	0.64
1539	ITKLSDLKAIDDKID	0	9056	0	175.5	0	0	0	0	42	0	0	93	484.5	0	0	297	117.75	1156	512	662	0	0	337.5	41	0	0	0	67	228.25	3890.75	0	0	0.81
1540	TKLSDLKAIDDKIDL	546	7964.5	0	0	0	0	0	0	0	0	0	153.5	246	0	0	259.5	83.75	1791	517.5	471.75	0	0	0	0	0	0	0	0	0	2980.5	0	0	0.98
1541	KLSDLKAIDDKIDLF	559.5	6722.25	0	0	29	0	15	0	0	6.25	0	0	173.5	91.5	0	210.25	0	1143.75	223	1356.5	0	0	100.5	0	0	129	0	18.25	0	1306.5	12	11	1.19
1542	LSDLKAIDDKIDLFK	0	4847.5	0	0	0	0	0	0	0	0	0	143.5	0	1159	0	645.25	1507.5	999.25	0	882	0	12.5	0	0	0	18.5	0	0	0	2421.5	0	26.5	0.36
1543	SDLKAIDDKIDLFKN	0	3783	0	0	0	0	0	0	0	0	0	14	255.5	368.5	0	0	0	0	0	290	0	0	0	0	6	0	0	0	0	1125.5	74.5	0	0.98
1544	DLKAIDDKIDLFKNP	0	1864.25	0	0	0	0	0	0	843	0	0	278	1706	1026.5	0	3181.25	25603.75	9913.5	391	3994.5	0	44.5	0	553.25	0	268	10.75	4.25	0	1796.25	118.5	384.25	-0.32
1545	LKAIDDKIDLFKNPY	6074.5	0	0	0	0	0	0	0	106.5	0	0	0	357.5	59.5	0	707.75	1302	513.25	67.75	1188	0	0	0	0	22.5	0	0	0	0	548.5	0	0	0.89
1546	KAIDDKIDLFKNPYD	10378.25	2068	0	0	0	0	0	0	3310.75	46.5	28	30	412.5	583.5	0	2485.75	23245.5	9463.5	1022.5	4065.25	382.5	40.5	198	464.5	401.5	581	0	0	83.5	162.25	102.5	95.5	-0.40
1547	AIDDKIDLFKNPYDF	4914.75	668.5	226.75	679.5	13.5	14.5	80.5	33	1489	72	45	0	162.5	331	28.5	1135.75	8205	10014.5	499.5	3037.5	317.75	28.5	89	101.5	98	183.75	13	0	159.5	0	16.25	101.5	-0.42
1548	IDDKIDLFKNPYDFE	7696	1440.25	415.75	8417	52	27	71.5	17.5	2841	171	72	23.5	347	549.25	2165.5	3233.75	16465.25	10821	690	4832	394	99.5	91.5	244.75	255.5	359.5	69	220.5	119.5	624.5	72	202.5	0.07
1549	DDKIDLFKNPYDFEA	11650.5	3136	0	766.25	0	10	48.75	0	2225.25	103.25	10	18.25	476	568	2428.25	1656.75	15478	16197.25	1128.25	4346.75	466	75.5	86	279	402	160.5	0	36	52.25	207	60.5	111.5	-0.21
1550	DKIDLFKNPYDFEAI	13283.25	1398	0	0	0	20.5	32	0	2205.25	68.75	8	10.25	470.5	353.5	3411.75	2540	12547.5	12981	371	2519.75	617	19.5	38	546	184.25	392	62.5	0	46.5	32.5	27.5	141.5	-0.13
1551	KIDLFKNPYDFEAIK	0	1590	0	0	0	0	0	0	0	26	0	0	261	0	747.25	1254	0	15954.5	1467.5	2141.75	479	0	0	0	0	623.5	0	0	0	113.5	0	102.5	-0.44
1552	IDLFKNPYDFEAIKK	4804.25	2687.25	0	0	0	0	0	0	587	0	0	0	313	0	480.25	3701.25	0	18905.5	4358.25	3146.5	740	0	0	31	86.25	1077	232.25	0	37	691.5	0	404	-0.50
1553	DLFKNPYDFEAIKKL	3849	2211	0	0	0	0	0	0	1182.5	0	0	0	587.25	93	444	5568.25	29710.25	15079.5	3760	2523.75	0	0	0	31	38.5	0	152	0	0	1178	0	55	-0.46
1554	LFKNPYDFEAIKKLI	3153.5	1497.75	0	163.5	0	0	0	0	947.5	35	0	0	731	0	203.5	4777.25	9666	12400	4263.5	2945.75	626.5	0	0	111.5	0	431	112	0	0	881.25	6	67	-0.48
1555	FKNPYDFEAIKKLIN	4451.75	630.75	0	0	0	0	0	0	187	0	0	17	51	0	180	1438.25	0	3863	1448.75	1495	739.25	0	0	406.5	0	177	0	0	0	201.5	178.5	104.5	0.19
1556	KNPYDFEAIKKLIND	448.5	0	69.75	303	0	0	0	0	73	0	0	0	0	0	447	1669.25	3005	2699.25	910.25	1149.5	491.25	0	10.75	71.5	4.25	233	0	0	16	0	31	51.5	-0.33
1557	NPYDFEAIKKLINDD	0	0	276.25	304.5	15.5	15	21.5	0	0	48	31.5	0	360.5	108	472	397.25	1864.25	2235.75	0	977	486	15.5	99	625.5	18	0	59	168	82	358.5	0	152	-0.26
1558	PYDFEAIKKLINDDT	0	852.5	41.5	0	0	0	15	0	114.5	31	64.5	46	262.25	71.5	0	376.75	0	1824.25	0	422.5	301.5	0	108.5	413	0	45.5	37	85.5	51.5	437.25	111	168.5	-0.54
1559	YDFEAIKKLINDDTK	14375.25	4436	0	0	0	0	0	0	448.5	0	0	0	0	513.25	0	0	0	1005	0	144.25	46	0	20	107.5	0	0	0	0	0	140	24.25	0	2.40
1560	DFEAIKKLINDDTKK	26873.25	2036.75	0	0	0	0	0	0	0	15.25	0	0	0	0	0	211	0	960.5	0	0	0	0	78.5	0	52.5	0	0	0	0	0	0	0	1.63
1561	FEAIKKLINDDTKKD	64614	64990	0	0	0	0	0	0	0	0	0	0	29.75	364	0	0	0	316.75	0	0	0	0	15	0	91.5	10.5	0	0	0	0	0	0	3.58
1562	EAIKKLINDDTKKDM	58776.25	32882.25	94.5	0	0	0	0	0	0	21	0	0	0	106	0	0	0	0	0	667.75	0	0	0	0	0	0	0	0	0	0	0	0	3.20
1563	AIKKLINDDTKKDML	60551	64990	85.5	0	0	0	0	0	0	0	0	0	497	0	0	36.5	0	0	0	1694	0	0	16.5	0	11.75	0	0	0	0	0	31	0	3.56
1564	IKKLINDDTKKDMLG	22554.5	39739.75	0	0	0	0	0	0	0	0	0	0	255.25	0	0	0	0	0	0	3416.75	0	0	6	0	0	0	0	0	0	0	28	0	3.14
1565	KKLINDDTKKDMLGK	20493.25	28881.5	0	0	0	0	0	0	0	0	0	0	84	0	0	0	0	0	0	0	0	0	0	0	16	349.25	0	0	0	1118	15.5	0	3.40
1566	KLINDDTKKDMLGKL	9740.75	17708	0	0	0	0	0	0	0	0	0	0	0	0	0	0	0	623.5	0	169.75	0	0	0	0	0	128.5	0	0	0	5230.75	12	0	2.73
1567	LINDDTKKDMLGKLL	28811.5	28666.5	0	0	0	0	0	0	0	0	60.25	0	30.5	0	0	0	0	4330	0	0	0	0	0	0	0	0	355	187.5	0	4367.25	107	428	3.32
1568	INDDTKKDMLGKLLS	24730.5	35918.5	0	0	0	0	0	0	257.5	0	174	0	0	0	0	0	293	7276.5	0	0	0	0	6546.25	0	29.5	0	375.25	628.5	196.75	3040	654.75	253	2.91
1569	NDDTKKDMLGKLLST	26295.5	45589	0	0	0	0	16	0	367.75	0	430.5	51.5	0	0	0	269	343.5	0	0	145	0	0	17078.5	38	137.5	0	896.75	1454.5	445.5	6943.5	2260.25	826	2.44
1570	DDTKKDMLGKLLSTG	10714	42677.25	0	0	0	0	0	0	503	0	389	0	0	0	0	305.5	224.25	10719.5	0	290	0	0	8919.5	0	0	0	433	646.5	526.5	4653	0	824	1.72
1571	DTKKDMLGKLLSTGL	0	6494.5	0	0	0	0	0	0	340.5	0	434	0	151.5	0	0	323	1420	11600	0	0	0	0	1356.75	421.5	0	412.75	432.25	1300.5	675.5	3204	2451.25	1725.5	-0.42
1572	TKKDMLGKLLSTGLV	0	0	0	0	0	0	0	0	637	0	440.25	0	124	0	0	1095	2098.25	12528.25	0	0	0	0	2007.5	1112.5	119.5	0	745.5	853.75	885.5	5968	2910	2380.75	-0.08
1573	KKDMLGKLLSTGLVQ	0	0	0	0	0	0	0	0	458.5	0	220.25	0	0	0	0	564.5	833.75	6995.5	0	0	0	0	173.5	269	0	617.5	375	0	421	3255.25	918	922.5	-0.17
1574	KDMLGKLLSTGLVQN	0	0	0	0	0	0	0	0	0	0	0	0	0	0	0	0	0	669.75	0	0	0	0	0	27.5	0	91	0	0	0	0	0	0	-0.38
1575	DMLGKLLSTGLVQNF	0	0	0	334.5	0	0	0	0	0	0	0	10.5	0	0	0	0	0	0	44.25	44	0	0	0	0	0	0	0	0	0	0	0	0	1.18
1576	MLGKLLSTGLVQNFP	0	0	0	80.5	0	0	0	0	0	0	0	0	0	0	0	150	0	81.5	266	25	0	0	0	0	1388.75	0	0	0	0	0	0	0	-0.41
1577	LGKLLSTGLVQNFPN	436.25	0	0	0	0	0	0	0	0	0	0	0	0	0	0	87	0	0	0	0	0	0	0	0	1380.25	0	0	0	0	0	0	0	-0.45
1578	GKLLSTGLVQNFPNT	146.5	0	0	0	0	0	0	0	0	0	0	0	0	0	0	231	0	0	3.5	0	0	0	0	0	282	0	0	0	0	0	0	0	-0.42
1579	KLLSTGLVQNFPNTI	0	0	0	0	0	0	0	0	0	0	0	0	0	0	0	290	0	0	0	0	0	0	0	0	0	80	0	0	0	0	0	0	-0.37
1580	LLSTGLVQNFPNTII	211	0	0	0	0	0	0	0	0	0	0	0	0	0	4.75	16197	0	0	0	0	0	0	0	0	75	0	0	0	0	0	0	0	-0.43
1581	LSTGLVQNFPNTIIS	249.5	0	0	679.5	0	0	0	0	0	0	0	13	0	0	0	1847.5	0	2712	160.5	0	0	0	0	0	0	0	0	96.5	0	2903.5	0	18.25	-0.50
1582	STGLVQNFPNTIISK	435.25	0	0	0	0	0	0	0	0	0	0	179.5	0	0	0	133	0	3013.75	66.5	177	13.5	0	0	0	41.5	168.75	0	0	0	3685.75	46.5	367	-0.39
1583	TGLVQNFPNTIISKL	436	809.25	0	0	0	0	0	0	287.5	0	0	143	14	0	0	256.75	179	1920.75	0	223	7	0	0	0	0	78.5	23	26	0	3998.5	0	201	-0.57
1584	GLVQNFPNTIISKLI	0	28707	0	0	0	0	0	0	542	0	0	332	0	0	0	2390.5	1096.5	1515	620.75	0	0	0	0	0	0	128.5	0	0	0	3332.5	16.5	52.5	1.18
1585	LVQNFPNTIISKLIE	1003.5	60429	0	0	0	0	0	0	1987.5	18.5	56.5	760.5	20.5	0	34	1462.5	697.5	3537.25	1137.75	1151.5	127.5	0	134.5	0	136	548.75	174.25	0	30.5	4366.5	139.5	441.5	1.29
1586	VQNFPNTIISKLIEG	1420.25	54862.25	0	0	0	0	0	0	1236.75	0	0	334	0	0	0	1318	424.5	3075.5	471.75	0	32.5	0	0	0	0	60	12	0	0	1910.75	67.25	128.5	1.38
1587	QNFPNTIISKLIEGK	442.75	42582.5	0	0	0	0	0	0	467.75	0	0	148	0	0	0	569.5	0	2441	315	745.5	0	0	0	102	0	0	0	0	0	970.75	0	0	1.34
1588	NFPNTIISKLIEGKF	0	22025	0	0	0	0	0	0	48.5	0	0	834.75	0	0	0	434.5	290	431.5	0	206.75	39.5	0	0	387.5	0	0	0	0	0	601.75	0	0	1.30
1589	FPNTIISKLIEGKFQ	0	3294	0	0	0	0	0	0	0	0	0	0	0	0	0	30.5	0	0	0	0	0	0	0	43	0	0	0	0	0	398.5	0	0	1.28
1590	PNTIISKLIEGKFQD	1464	5134.5	0	0	4	0	0	0	0	0	0	0	0	0	0	0	0	0	0	0	0	0	0	0	0	0	0	0	0	0	0	0	2.43

1591 NTIISKLIEGKFQDM	1959	4257	0	0	3.5	0	0	0	0	0	0	72.75	0	0	0	0	0	0	0	0	0	0	0	0	0	0	0	0	0	0	0	0		2.96
1592 TIISKLIEGKFQDML	1249	3893.5	0	0	6	0	0	0	0	0	0	423.25	43.75	0	0	122	0	1333.25	0	46	0	0	0	0	0	107	0	0	0	174.75	0	0		1.94
1593 IISKLIEGKFQDMLN	677	0	12.5	0	0	0	0	0	0	0	51.75	251.5	37	0	0	480.5	187	2252	0	5250.5	169.5	98.75	0	0	0	0	1722.5	0	12	2150	0	146.5		-0.32
1594 ISKLIEGKFQDMLNI	743	0	0	0	0	0	0	0	0	0	184	377.75	0	0	0	720.5	0	2212.25	0	234	138	0	959.5	0	0	0	5374.5	0	0	2227.5	0	0		-0.35
1595 SKLIEGKFQDMLNIS	704	0	0	0	0	0	0	0	0	0	0	0	0	0	0	634.75	0	2131.5	0	270.5	39	0	1680.75	8	0	941.5	1715.75	0	390.5	800.5	0	0		-0.34
1596 KLIEGKFQDMLNISQ	157	0	0	0	11.5	0	0	0	0	0	0	0	0	48	0	122	0	1307.75	0	157	0	0	821	0	0	109.5	1491.25	0	0	276	0	0		-0.28
1597 LIEGKFQDMLNISQH	165.5	0	0	0	18.5	3	0	4	0	0	7	0	0	0	0	0	0	1149.5	0	295.5	0	0	0	0	0	0	908.75	0	0	0	0	0		-0.42
1598 IEGKFQDMLNISQHQ	235	0	0	0	0	0	0	0	0	0	0	0	24.5	0	0	0	0	297.25	0	0	0	0	0	0	0	0	752	0	0	0	0	0		-0.51
1599 EGKFQDMLNISQHQC	0	0	0	127	11.5	0	3.5	0	0	115	0	0	218	92	0	18.5	0	598.75	0	0	0	0	0	0	0	0	397.75	0	0	0	0	0		-0.43
1600 GKFQDMLNISQHQCV	0	0	0	0	0	0	0	0	0	0	0	0	0	0	0	0	0	357	0	0	0	0	0	0	0	0	323.5	0	0	0	0	0		-0.33
1601 KFQDMLNISQHQCVK	0	0	0	0	0	0	0	0	0	0	0	0	0	0	0	0	0	0	0	247	0	0	0	0	0	381.5	733.25	0	0	0	0	0		-0.29
1602 FQDMLNISQHQCVKK	0	0	0	0	0	0	0	0	0	0	0	0	0	0	0	0	0	0	0	0	0	0	0	0	0	0	0	0	0	0	0	0		
1603 QDMLNISQHQCVKKQ	0	0	0	272	0	0	0	0	0	0	0	0	0	0	0	0	0	0	0	0	0	0	0	0	0	0	0	0	0	0	0	0		1.37
1604 DMLNISQHQCVKKQC	0	0	0	0	0	0	0	0	0	0	0	0	0	0	0	0	0	0	0	0	0	0	0	0	0	0	0	0	0	0	0	0		
1605 MLNISQHQCVKKQCP	0	0	0	0	0	0	0	0	0	0	0	0	0	0	0	0	0	0	0	0	0	0	0	0	0	0	0	0	0	0	0	0	17	
1606 LNISQHQCVKKQCPE	0	0	0	0	0	0	0	0	0	0	0	0	0	0	0	0	0	0	0	0	0	0	0	0	0	0	0	0	0	0	0	0		
1607 NISQHQCVKKQCPEN	0	0	0	10132	0	0	0	0	0	0	0	37	0	0	0	0	0	0	0	0	0	0	0	0	0	0	0	0	0	0	0	0		1.37
1608 ISQHQCVKKQCPENS	0	0	0	20405.25	0	0	0	0	0	0	0	0	0	0	0	0	0	0	0	0	0	0	0	0	0	0	0	0	0	0	0	0		1.37
1609 SQHQCVKKQCPENSG	0	0	0	8844.5	0	0	0	0	0	0	0	0	0	0	0	0	0	0	0	0	0	0	0	0	0	0	0	0	0	0	0	0		1.37
1610 QHQCVKKQCPENSGC	0	0	0	11773	0	0	0	0	0	0	0	0	0	0	0	0	0	0	0	0	0	0	0	0	0	0	0	0	0	0	0	0		1.37
1611 HQCVKKQCPENSGCF	0	0	0	0	0	0	0	0	0	0	0	0	0	0	0	0	0	0	0	0	0	0	0	0	0	0	0	0	0	0	0	0		
1612 QCVKKQCPENSGCFR	0	0	0	668.5	0	0	0	0	0	0	0	0	0	0	0	0	0	0	0	0	0	0	0	0	0	0	0	0	0	0	0	0		1.37
1613 CVKKQCPENSGCFRH	0	0	0	0	0	0	0	0	0	0	0	0	0	0	0	0	0	0	0	0	0	0	0	0	0	0	0	0	0	0	0	0		
1614 VKKQCPENSGCFRHL	0	0	0	167.5	0	0	0	0	0	0	0	0	0	0	0	0	0	0	0	0	0	0	0	0	0	266	0	0	0	0	0	0		-0.10
1615 KKQCPENSGCFRHLD	0	0	0	451.5	0	0	0	0	0	0	0	0	0	0	0	0	0	0	0	0	0	0	0	0	0	503.5	0	0	0	0	0	0		0.17
1616 KQCPENSGCFRHLDE	0	0	286	1312	0	0	8.25	0	0	24.5	0	0	0	53	0	0	0	0	0	0	0	0	31.5	0	0	39.5	10	0	0	0	0	10.25		2.12
1617 QCPENSGCFRHLDER	0	0	0	1559	20	13.5	14	0	0	24	0	0	0	48.5	0	0	0	0	0	0	0	0	27.5	0	0	0	0	0	0	0	0	23		1.33
1618 CPENSGCFRHLDERE	0	0	575.75	5007.25	31.5	26	47	7	0	49.5	50	0	0	308	0	0	0	0	0	0	4.25	39.5	56.5	37	35	0	57.5	99.5	62	0	20	40.75		1.71
1619 PENSGCFRHLDEREE	0	0	633.5	5773	62	50	94.5	11.5	58.75	141	94	16.5	518	229	40	0	0	0	0	16.5	1.5	56	117.5	60.5	47.5	6	113	248.5	86.5	0	45	62		1.60
1620 ENSGCFRHLDEREEC	0	0	793.5	1272.75	82	50	90.5	19	57	167.75	68	24	416	454.5	47	66	232	0	0	61.5	19.5	38.5	118.5	28	37.5	0	76.5	254	150.5	28.5	47	84.5		1.82
1621 NSGCFRHLDEREECK	0	0	0	0	0	11	0	0	0	0	0	0	0	0	0	0	0	0	0	0	0	0	17	0	0	0	0	0	0	0	0	0		-0.41
1622 SGCFRHLDEREECKC	0	0	0	0	0	12	16.5	0	0	0	14	0	0	0	0	0	0	0	0	0	0	16.5	33	0	0	0	7	24	18.5	1.5	20.5	65		-0.05
1623 GCFRHLDEREECKCL	0	0	0	0	28	12	6.5	0	0	13.5	0	0	0	0	0	0	0	0	0	0	0	0	39	0	0	0	0	0	9.5	0	0	43		-0.25
1624 CFRHLDEREECKCLL	0	0	0	0	14.25	0	0	3	0	6.5	0	0	0	0	0	0	0	0	0	0	0	6	35.5	0	0	0	6	0	0	0	0	0		-0.32
1625 FRHLDEREECKCLLN	0	0	92.75	0	0	0	0	0	0	0	0	0	0	0	0	0	0	0	0	0	0	0	14	0	0	0	0	0	0	0	0	0		1.27
1626 RHLDEREECKCLLNY	0	0	0	0	0	0	0	0	0	0	0	0	0	0	0	0	0	0	0	0	0	0	25.5	0	0	0	0	0	0	0	0	0		-0.41
1627 HLDEREECKCLLNYK	0	0	0	0	0	0	0	0	0	0	0	0	0	0	0	0	0	0	0	0	0	0	0	0	0	0	0	0	0	0	0	0		
1628 LDEREECKCLLNYKQ	0	0	0	0	0	0	0	0	0	0	0	0	0	0	0	0	0	0	0	0	0	0	0	0	0	0	0	0	0	0	0	0		
1629 DEREECKCLLNYKQE	0	0	0	38.5	0	3.75	0	0	48.5	376	0	0	374.5	115.5	7.5	0	0	19	0	0	0	0	0	0	0	0	0	54.5	0	0	0	0		-0.32
1630 EREECKCLLNYKQEG	0	0	0	0	13.5	8	0	0	0	1683.5	29.5	0	1093.75	90.5	33.5	0	0	0	0	0	0	0	0	0	0	0	11.75	0	0	0	0	0		-0.31
1631 REECKCLLNYKQEGD	0	0	205	91.5	18.5	9	11	0	45	22	0	0	1274	34.5	14.75	0	92.5	0	27.5	0	0	10	0	0	0	0	8	67.25	0	0	0	0		-0.55
1632 EECKCLLNYKQEGDK	0	0	298	1482.5	14.5	25.5	0	0	0	36.5	52	33.5	2003.25	434.25	0	1910.5	920.75	573.5	2007.5	1066	13	163.25	691.5	127	0	0	0	162	0	2768.75	75.5	0		-0.49
1633 ECKCLLNYKQEGDKC	0	0	0	527.75	0	0	0	0	0	0	18.5	0	0	351.5	0	1739.75	490.5	466.5	1411.75	851.25	16	127	299	0	0	281.75	0	127.25	0	2858.75	40.5	0		-0.27
1634 CKCLLNYKQEGDKCV	0	190.5	0	627.25	0	0	0	0	0	0	0	0	0	180	0	1508	441.75	731	0	910.25	0	145.5	0	63	0	0	0	0	0	2137	46.5	0		-0.50
1635 KCLLNYKQEGDKCVE	1080	1028.5	75.5	443.5	14.5	0	16	0	0	0	59	24.5	151	132.5	0	1711	283.5	861.75	0	931.25	0	83.5	2137	64	0	241.5	0	25	0	2996	22	114.5		-0.31
1636 CLLNYKQEGDKCVEN	989.5	0	0	1015	0	0	12	0	0	0	0	0	20.5	38	0	1194.5	122.5	277.5	1320.5	676	0	21.5	1583.25	0	0	0	0	0	0	1146	0	0		-0.18
1637 LLNYKQEGDKCVENP	701	228.25	0	5491	0	0	13	0	0	0	0	0	22.5	94.5	0	1505.75	0	720.5	989	795	0	42.5	0	0	0	0	0	0	0	2783.5	0	0		0.90
1638 LNYKQEGDKCVENPN	334	0	0	4311	0	0	0	3.5	0	0	0	0	0	43	0	1219.5	60.75	521.5	0	937	0	63.5	0	0	0	0	0	0	0	1493.25	0	35.5		0.88
1639 NYKQEGDKCVENPNP	0	210	0	1212.75	0	0	4	0	0	0	26.5	0	0	6.5	0	958.5	58	191	213	434	0	26.5	0	0	0	0	0	0	160.5	1145	0	0		0.04
1640 YKQEGDKCVENPNPT	0	205	0	0	0	0	0	0	0	0	0	0	0	0	0	609.5	0	115	18.5	321.5	0	0	0	0	0	0	0	0	0	135.5	0	0		-0.57
1641 KQEGDKCVENPNPTC	0	0	0	508	0	0	0	0	0	0	0	0	0	0	0	262	0	163	0	171.5	0	0	0	0	0	0	0	0	0	0	0	0		0.47
1642 QEGDKCVENPNPTCN	0	386	420	13421	0	0	0	0	0	56.75	0	0	252.5	0	0	0	127.5	0	0	0	0	0	0	0	0	0	18	0	0	0	0	0		1.58
1643 EGDKCVENPNPTCNE	0	775.5	957.75	12295	0	0	30	0	22	744	209.5	0	564.5	209	30	0	0	0	0	0	0	0	63.5	17.5	0	0	24.5	69	0	0	0	0		1.84
1644 GDKCVENPNPTCNEN	63	1354.25	355.75	19061.25	0	0	0	0	0	0	0	0	30.75	0	0	0	260	51	63	0	0	0	0	0	19.5	0	0	0	0	0	0	0		1.72
1645 DKCVENPNPTCNENN	0	665.5	112	30018.5	0	0	0	0	0	0	0	0	0	0	0	0	308	0	0	0	0	0	0	0	52.5	0	31.75	0	0	0	0	0		1.46
1646 KCVENPNPTCNENNG	25	350.75	0	18044.25	0	0	0	0	0	0	0	0	0	0	0	0	93	0	0	0	0	0	0	0	0	133.5	0	0	0	0	0	0		1.44
1647 CVENPNPTCNENNGG	0	63.5	0	11093	0	0	0	0	0	0	0	0	0	0	0	0	0	0	0	0	0	0	0	0	0	0	0	0	0	0	0	12.5		1.39
1648 VENPNPTCNENNGGC	0	0	83.5	13098	0	8	0	0	0	0	0	0	97.25	27	0	0	0	3	0	0	0	0	0	0	3	0	0	0	0	0	0	0		1.39
1649 ENPNPTCNENNGGCD	0	11.25	247.75	8929.75	0	0	0	6	33	24	12	0	87.5	68.25	25.5	0	73	18	0	0	27	21.5	83	0	28	0	43.5	80.5	18	0	7	46.5		1.44
1650 NPNPTCNENNGGCDA	0	0	723	7053.25	0	0	0	3	0	0	0	0	0	0	9	25	0	0	0	0	13	0	8	0	16	0	54	0	0	0	2.5	0		1.75
1651 PNPTCNENNGGCDAD	0	0	752.75	8389.75	0	0	14	17	1421.75	0	8	16	0	79.5	18.5	2.5	119.75	0	0	17	27.5	35	89.5	65	34	0	65.5	288.25	41	0	0	20		1.52
1652 NPTCNENNGGCDADA	0	0	110.5	4562.5	0	15	9	13	101	0	0	16	0	20.5	0	0	24	45.75	0	0	26	14	44	0	10.5	0	52	82	49	0	17.5	0		1.41
1653 PTCNENNGGCDADAT	0	0	0	4578.5	0	0	3	0	156.5	0	0	0	53.75	0	0	0	0	0	0	0	0	0	23.5	0	0	0	0	0	0	0	0	0		1.35
1654 TCNENNGGCDADATC	0	0	0	1713.5	0	0	0	0	0	0	0	0	0	9.25	0	0	9.5	0	0	0	0	51.5	0	0	0	0	0	0	0	0	0	0		1.35
1655 CNENNGGCDADATCT	0	0	0	1401.25	0	0	27.5	0	0	0	0	0	0	0	0	0	0	0	0	0	0	1456	0	0	0	0	0	0	0	0	0	0		0.23
1656 NENNGGCDADATCTE	0	0	97	2635	0	0	68	0	0	10.5	0	0	234.25	49	0	0	0	0	0	0	0	62.5	0	0	0	0	45	68	19.5	0	0	0		1.41
1657 ENNGGCDADATCTEE	0	0	240.5	10390	13.25	53.5	201	0	45.5	83.75	27	0	521	164	21.5	24.5	46	0	0	0	0	53	0	0	0	0	80	292	62	0	20	30		1.39
1658 NNGGCDADATCTEED	0	0	456.5	8223	60.5	48.5	385.5	7.25	72	26	42	128.5	115.5	131	17	60.5	42.5	0	0	0	0	53	0	0	0	0	140.5	591.5	94	0	46.5	15.25		1.48
1659 NGGCDADATCTEEDS	0	92	195	1244.25	18	26.5	330	0	40.5	17.5	15.5	21	84.5	50.5	0	0	0	0	0	0	0	39	0	0	0	0	20	209.5	52.5	0	0	0		1.98
1660 GGCDADATCTEEDSG	0	69.5	0	543.75	9.75	0	205.75	0	131	0	0	0	0	0	0	0	9	0	0	0	0	26.5	0	0	0	0	0	0	15	0	0	0		1.57
1661 GCDADATCTEEDSGS	0	116.5	0	1396	0	49	91.5	0	0	0	41.5	0	0	0	0	0	0	0	0	0	0	11	125	0	0	0	0	0	0	0	0	0		1.61
1662 CDADATCTEEDSGSS	0	0	0	0	0	59.5	86	0	0	0	906.25	0	18	0	0	0	0	0	0	0	0	21.5	96.25	0	0	0	0	97.25	0	0	0	0		-0.36
1663 DADATCTEEDSGSSR	0	0	0	0	0	17	18	0	0	0	0	0	0	0	0	0	0	0	0	0	0	15	0	0	0	0	51.5	0	11	0	0	0		-0.33
1664 ADATCTEEDSGSSRK	721	0	0	0	0	0	15	0	0	0	0	0	0	0	0	0	0	0	0	0	0	0	0	0	0	254	0	0	0	0	0	0		1.03
1665 DATCTEEDSGSSRKK	480	0	0	0	0	0	0	0	0	0	0	0	0	0	0	0	0	0	0	0	0	0	0	0	0	271.25	0	0	0	0	0	0		0.75
1666 ATCTEEDSGSSRKKI	1128.5	0	0	40.25	0	0	0	0	0	0	0	0	0	0	0	50	0	0	0	0	0	0	0	0	0	301.5	0	0	0	0	0	0		1.24
1667 TCTEEDSGSSRKKIT	2324.5	21.5	108.25	0	0	0	0	0	0	0	12.5	0	20.5	0	0	0	0	0	0	0	0	0	0	0	0	315	0	0	0	0	0	0		1.48
1668 CTEEDSGSSRKKITC	677	66.5	48.5	0	0	0	0	0	38	0	0	0	28	0	0	0	0	0	0	0	0	0	0	0	0	341	0	0	0	0	0	0		1.28
1669 TEEDSGSSRKKITCE	841.5	21.5	103.5	143.5	35.5	49	0	0	0	43.5	48	2	2257.25	96.5	0	34	0	37	16.5	10	20	32	37	0	7	392.5	12	99.5	89.5	0	0	98		-0.33
1670 EEDSGSSRKKITCEC	545	2	120	0	21.5	149	26	0	0	76.5	28	0	2844.75	161	24	26	21.5	29	57	0	6	23.25	36.5	6	0	0	0	69.5	34	35	9	30.5		-0.55
1671 EDSGSSRKKITCECT	0	0	0	0	0	0	0	0	0	0	0	0	43	99	0	0	0	0	0	0	0	0	0	0	0	170.5	0	0	0	57	0	0		-0.24
1672 DSGSSRKKITCECTK	0	0	0	0	0	0	0	0	0	0	0	0	0	0	0	0	0	0	0	0	0	0	0	0	0	0	0	0	0	0	0	0		
1673 SGSSRKKITCECTKP	0	0	0	0	0	0	0	0	0	0	15.5	0	0	0	0	0	0	0	0	0	0	0	0	0	0	0	0	0	0	0	0	0		-0.41
1674 GSSRKKITCECTKPD	0	0	0	0	0	0	0	6.75	0	0	0	0	0	0	0	0	0	0	0	0	0	0	0	0	0	0	0	3	0	0	0	0		-0.41
1675 SSRKKITCECTKPDS	20.25	0	0	558.5	0	0	0	1423	0	0	68.5	0	118	0	0	0	0	91	0	0	0	0	36.5	0	0	0	0	0	0	0	65	0		1.06
1676 SRKKITCECTKPDSY	0	0	0	0	0	0	0	0	0	0	6	0	0	0	0	0	0	0	0	0	0	0	0	0	0	0	0	0	0	0	0	0		-0.41
1677 RKKITCECTKPDSYP	0	0	0	0	0	0	56.5	0	0	0	0	0	0	0	0	0	81	221.5	0	0	0	0	0	0	0	84	0	0	0	0	0	0		-0.29
1678 KKITCECTKPDSYPL	0	0	0	193	0	0	0	0	0	0	0	0	0	0	0	0	0	0	0	0	0	0	0	0	0	539.25	0	0	0	0	0	0		-0.39
1679 KITCECTKPDSYPLF	0	0	0	846.25	0	0	0	0	0	0	0	0	0	0	0	0	331.5	0	0	0	0	0	0	0	0	240.25	0	0	0	0	0	0		0.80
1680 ITCECTKPDSYPLFD	0	345.5	240.5	1023.25	0	0	61	0	77	0	179.75	0	0	0	260.5	53.5	194.75	745.25	13.5	128.25	13.5	0	42	0	0	0	10	52.5	1092.75	0	9	0		0.52
1681 TCECTKPDSYPLFDG	0	121.5	0	210.5	0	0	0	0	0	0	21.25	0	0	0	0	0	0	0	0	0	0	0	0	0	0	0	0	0	38	0	0	0		2.95
1682 CECTKPDSYPLFDGI	0	131.5	0	835.5	0	7.5	2.5	0	0	0	69	0	0	0	0	12.5	0	955	0	0	0	0	4.5	0	0	0	0	0	0	0	0	0		0.35
1683 ECTKPDSYPLFDGIF	0	0	9.5	314.75	0	4.5	0	0	0	32.5	38.25	0	12.5	154.5	22	7.75	2961.25	2196	0	0	0	0	51	0	0	0	0	0	0	0	0	0		-0.42
1684 CTKPDSYPLFDGIFC	0	0	0	0	0	9	8	0	0	3	2	0	0	0	0	0	0	0	0	0	0	0	4.5	0	0	0	0	0	2	0	9.5	0		-0.21
1685 TKPDSYPLFDGIFCS	0	0	0	0	0	0	0	0	0	0	0	0	0	0	0	0	0	2023.5	0	0	0	0	0	0	0	0	0	0	0	0	0	0		-0.41
1686 KPDSYPLFDGIFCSS	0	0	0	0	0	0	0	0	0	0	0	0	0	0	0	0	0	2507	0	0	0	0	2	0	0	0	0	0	0	0	0	0		-0.41
1687 PDSYPLFDGIFCSSS	0	0	0	775	0	2.5	0	0	0	0	188	0	0	0	0	0	1704.25	3159	0	0	0	0	0	0	0	0	0	0	0	0	0	0		-0.56
1688 DSYPLFDGIFCSSSN	0	20.5	0	857.5	0	0	0	0	0	0	0	0	0	0	0	0	2863.25	2706.5	0	20	0	0	0	0	0	0	0	0	0	0	0	0		-0.56
1689 SYPLFDGIFCSSSNF	0	0	261	483.5	0	0	0	0	0	0	4.5	0	0	0	0	0	377	143	0	77.5	0	0	0	0	0	0	0	0	0	0	0	0		1.35
1690 YPLFDGIFCSSSNFL	0	0	0	0	0	6.5	0	0	0	0	0	0	0	0	0	0	0	0	0	0	0	0	0	0	0	0	0	0	0	0	0	0		
1691 PLFDGIFCSSSNFLG	0	0	0	251	11.75	0	13.5	0	0	0	0	9.5	0	0	0	0	56	14.5	0	0	0	0	21	0	0	0	0	0	20	0	0	0		1.06
1692 LFDGIFCSSSNFLGI	0	0	340	0	0	0	0	0	0	0	0	0	0	0	0	0	0	0	0	0	0	0	0	0	0	0	0	0	0	0	0	0		1.37
1693 FDGIFCSSSNFLGIS	0	0	191	0	0	0	0	0	0	0	0	0	0	0	0	0	0	0	0	0	0	0	0	0	0	0	0	0	0	0	0	0		1.37
1694 DGIFCSSSNFLGISF	0	0	0	0	7	0	0	0	0	0	0	0	0	0	0	0	0	0	0	0	0	0	0	0	0	0	0	0	0	0	0	0		
1695 GIFCSSSNFLGISFL	0	0	0	0	0	0	0	0	0	0	0	0	0	0	0	0	41	0	23	0	0	0	0	0	0	0	0	0	0	0	0	0		-0.34
1696 IFCSSSNFLGISFLL	0	0	0	0	0	0	0	0	0	0	0	39.5	0	0	0	0	0	0	0	0	0	0	0	0	0	0	0	0	0	0	0	0		-0.41
1697 FCSSSNFLGISFLLI	0	0	0	0	0	0	0	0	0	0	0	0	0	0	0	0	0	0	0	0	0	0	0	0	0	0	0	0	0	0	0	0		
1698 CSSSNFLGISFLLIL	0	0	0	0	2	0	0	0	0	0	0	0	0	0	0	0	0	0	0	0	0	0	0	0	0	0	0	0	0	0	0	0		
1699 SSSNFLGISFLLILM	0	0	0	0	7	0	0	0	0	0	0	0	0	0	0	0	0	0	0	0	0	0	0	0	0	0	0	0	0	0	0	0		
1700 SSNFLGISFLLILML	0	0	0	0	0	0	0	0	0	0	0	0	0	0	0	0	0	0	0	0	0	0	0	0	0	0	0	0	0	0	0	0		
1701 SNFLGISFLLILMLI	0	0	0	0	0	0	0	0	0	0	0	0	0	0	0	0	0	0	0	0	0	0	0	0	0	0	0	0	0	0	0	0		
1702 NFLGISFLLILMLIL	0	0	0	0	0	0	0	0	0	0	0	0	0	0	0	0	0	0	0	0	0	0	0	0	0	0	0	0	0	0	0	0		
1703 FLGISFLLILMLILY	0	0	0	0	0	0	0	0	0	0	0	0	0	0	0	0	0	0	0	0	15.5	0	0	0	0	0	0	0	0	0	0	0		-0.41
1704 LGISFLLILMLILYS	0	0	0	0	0	0	0	0	0	0	0	0	0	0	0	0	49.5	0	0	0	6.5	0	0	0	0	0	0	0	0	0	0	0		-0.39
1705 GISFLLILMLILYSF	0	0	0	0	0	0	0	0	0	0	0	0	0	0	0	0	0	0	0	0	16	0	0	0	0	0	0	0	0	0	0	0		-0.41
1706 ISFLLILMLILYSFI	0	0	0	0	0	0	0	0	0	0	0	0	0	0	0	0	0	0	0	0	0	0	0	0	0	0	0	0	0	0	0	0		

Supplementary Table 3: Anamnestic data and all solicited and unsolicited adverse reactions grouped by participants. These are the original data underlying Table 1 in the article.

Obs	ethnic	USUBJID	BRTHDTC	AGE	SEX	armcd	AESEQ	AESPID	AETERM	AESER	AEREL	AESEV	AEACN	AEOUT	aeendt	Split_No_	LLT_Coded_Term	PT_Coded_Term	SOC_Coded_Term	aedosdur	aestdy	aeendy
1	Caucasian/White	1	1992	25	Female	Placebo	1	1	Pain at injection site	No	(Definitely) related	Mild	Dose not changed	Recovered/ resolved	11/04/2017	.	Injection site pain	Injection site pain	General disorders and administration site conditions	0	1	1
2	Caucasian/White	1	1992	25	Female	Placebo	2	2	Fatigue	No	Unlikely	Mild	Dose not changed	Recovered/ resolved	21/04/2017	.	Fatigue	Fatigue	General disorders and administration site conditions	8	9	11
3	Caucasian/White	1	1992	25	Female	Placebo	4	4	induration at injection side	No	(Definitely) related	Mild	Dose not changed	Recovered/ resolved	12/05/2017	.	Injection site induration	Injection site induration	General disorders and administration site conditions	1	31	32
4	Caucasian/White	1	1992	25	Female	Placebo	5	5	Increase of lactat dehydrogenasis	No	Unlikely	Mild	Dose not changed	Recovered/ resolved	09/06/2017	.	Lactate dehydrogenase increased	Blood lactate dehydrogenase increased	Investigations	27	57	60
5	Caucasian/White	1	1992	25	Female	Placebo	6	6	Increase of Transaminase	No	Unlikely	Mild	Dose not changed	Recovered/ resolved	09/06/2017	.	Transaminases increased	Transaminases increased	Investigations	27	57	60
6	Caucasian/White	1	1992	25	Female	Placebo	11	11	warmth at injection site	No	(Definitely) related	Mild	Dose not changed	Recovered/ resolved	07/06/2017	.	Injection site warmth	Injection site warmth	General disorders and administration site conditions	0	58	58
7	Caucasian/White	1	1992	25	Female	Placebo	12	12	pain at injection site	No	(Definitely) related	Mild	Dose not changed	Recovered/ resolved	07/06/2017	.	Injection site pain	Injection site pain	General disorders and administration site conditions	0	58	58
8	Caucasian/White	1	1992	25	Female	Placebo	7	7	upper respiratory infektion	No	Possible	Mild	Dose not changed	Recovered/ resolved	14/06/2017	.	Upper respiratory infection	Upper respiratory tract infection	Infections and infestations	1	59	65
9	Caucasian/White	1	1992	25	Female	Placebo	8	8	Arthralgia	No	Possible	Mild	Dose not changed	Recovered/ resolved	10/06/2017	.	Arthralgia	Arthralgia	Musculoskeletal and connective tissue disorders	1	59	61
10	Caucasian/White	1	1992	25	Female	Placebo	9	9	sweating	No	Possible	Mild	Dose not changed	Recovered/ resolved	10/06/2017	.	Sweating	Hyperhidrosis	Skin and subcutaneous tissue disorders	1	59	61
11	Caucasian/White	1	1992	25	Female	Placebo	10	10	chills	No	Possible	Mild	Dose not changed	Recovered/ resolved	10/06/2017	.	Chills	Chills	General disorders and administration site conditions	1	59	61
12	Caucasian/White	1	1992	25	Female	Placebo	13	13	Decreased apetite	No	(Definitely) related	Mild	Dose not changed	Recovered/ resolved	09/06/2017	.	Decreased appetite	Decreased appetite	Metabolism and nutrition disorders	2	60	60
13	Caucasian/White	2	1964	53	Female	Adjuvant	1	1	Pain at injection site	No	(Definitely) related	Mild	Dose not changed	Recovered/ resolved	16/04/2017	.	Injection site pain	Injection site pain	General disorders and administration site conditions	0	1	6
14	Caucasian/White	2	1964	53	Female	Adjuvant	4	4	Warmth at injection site	No	(Definitely) related	Mild	Dose not changed	Recovered/ resolved	16/04/2017	.	Injection site warmth	Injection site warmth	General disorders and administration site conditions	2	3	6
15	Caucasian/White	2	1964	53	Female	Adjuvant	2	2	Hematom at right antecubital fossa	No	Not related	Mild	Dose not changed	Recovered/ resolved	13/05/2017	.	Hematoma	Haematoma	Vascular disorders	15	16	33
16	Caucasian/White	2	1964	53	Female	Adjuvant	3	3	Pain at injection site	No	(Definitely) related	Mild	Dose not changed	Recovered/ resolved	11/05/2017	.	Injection site pain	Injection site pain	General disorders and administration site conditions	1	30	31
17	Caucasian/White	2	1964	53	Female	Adjuvant	5	5	Hematoma at injection site	No	(Definitely) related	Mild	Dose not changed	Recovered/ resolved	23/05/2017	.	Hematoma injection site	Injection site haematoma	General disorders and administration site conditions	7	36	43
18	Caucasian/White	2	1964	53	Female	Adjuvant	6	6	Pain at injection site	No	(Definitely) related	Mild	Dose not changed	Recovered/ resolved	13/06/2017	.	Injection site pain	Injection site pain	General disorders and administration site conditions	2	59	64
19	Caucasian/White	2	1964	53	Female	Adjuvant	7	7	Pruritus	No	Not related	Moderate	Dose not changed	Recovered/ resolved	21/06/2017	.	Pruritus	Pruritus	Skin and subcutaneous tissue disorders	5	62	72
20	Caucasian/White	2	1964	53	Female	Adjuvant	9	9	Accidental fall	No	Not related	Mild	Dose not changed	Recovered/ resolved	12/06/2017	.	Falling down	Fall	Injury, poisoning and procedural complications	6	63	63
21	Caucasian/White	2	1964	53	Female	Adjuvant	8	8	GOT increased	No	Possible	Mild	Dose not changed	Recovered/ resolved	20/06/2017	.	GOT increased	Aspartate aminotransferase increased	Investigations	7	64	71
22	Caucasian/White	3	1960	57	Male	50 ug MSP-1	1	1	Pain at injection site	No	(Definitely) related	Mild	Dose not changed	Recovered/ resolved	13/04/2017	.	Injection site pain	Injection site pain	General disorders and administration site conditions	1	2	3
23	Caucasian/White	3	1960	57	Male	50 ug MSP-1	5	5	Contact dermatitis at right antecubital fossa (due to adhesive bandage)	No	Not related	Mild	Dose not changed	Recovered/ resolved	13/05/2017	.	Contact dermatitis	Dermatitis contact	Skin and subcutaneous tissue disorders	0	29	33
24	Caucasian/White	3	1960	57	Male	50 ug MSP-1	2	2	Pain at injection site	No	(Definitely) related	Mild	Dose not changed	Recovered/ resolved	16/05/2017	.	Injection site pain	Injection site pain	General disorders and administration site conditions	1	30	36
25	Caucasian/White	3	1960	57	Male	50 ug MSP-1	3	3	Induration at injection site	No	(Definitely) related	Mild	Dose not changed	Recovered/ resolved	16/05/2017	.	Injection site induration	Injection site induration	General disorders and administration site conditions	1	30	36
26	Caucasian/White	3	1960	57	Male	50 ug MSP-1	4	4	Warmth at injection site	No	(Definitely) related	Mild	Dose not changed	Recovered/ resolved	16/05/2017	.	Injection site warmth	Injection site warmth	General disorders and administration site conditions	1	30	36
27	Caucasian/White	3	1960	57	Male	50 ug MSP-1	6	6	Pain at injection site	No	(Definitely) related	Mild	Dose not changed	Recovered/ resolved	08/06/2017	.	Injection site pain	Injection site pain	General disorders and administration site conditions	1	58	59
28	Caucasian/White	3	1960	57	Male	50 ug MSP-1	10	10	populae in the axilla (both sides)	No	Not related	Mild	Dose not changed	Recovered/ resolved	15/06/2017	.	Papular rash	Rash papular	Skin and subcutaneous tissue disorders	4	61	66
29	Caucasian/White	3	1960	57	Male	50 ug MSP-1	7	7	cold symptoms	No	Possible	Mild	Dose not changed	Recovered/ resolved	16/06/2017	.	Cold symptoms	Nasopharyngitis	Infections and infestations	5	62	67
30	Caucasian/White	3	1960	57	Male	50 ug MSP-1	8	8	Dizziness	No	Possible	Mild	Dose not changed	Recovered/ resolved	16/06/2017	.	Dizziness	Dizziness	Nervous system disorders	5	62	67
31	Caucasian/White	3	1960	57	Male	50 ug MSP-1	9	9	Diarrhea	No	Possible	Mild	Dose not changed	Recovered/ resolved	14/06/2017	.	Diarrhea	Diarrhoea	Gastrointestinal disorders	5	62	65
32	Caucasian/White	3	1960	57	Male	50 ug MSP-1	11	11	increased sweating	No	Possible	Mild	Dose not changed	Recovered/ resolved	16/06/2017	.	Sweating increased	Hyperhidrosis	Skin and subcutaneous tissue disorders	5	62	67
33	Caucasian/White	3	1960	57	Male	50 ug MSP-1	12	12	Fatigue	No	Possible	Mild	Dose not changed	Recovered/ resolved	16/06/2017	.	Fatigue	Fatigue	General disorders and administration site conditions	5	62	67
34	Caucasian/White	3	1960	57	Male	50 ug MSP-1	13	13	Dizziness	No	Unlikely	Mild	Dose not changed	Recovered/ resolved	07/07/2017	.	Dizziness	Dizziness	Nervous system disorders	29	86	88
35	Caucasian/White	3	1960	57	Male	50 ug MSP-1	15	15	CRP increase	No	Unlikely	Mild	Dose not changed	Recovered/ resolved	28/09/2017	.	C-reactive protein increased	C-reactive protein increased	Investigations	1	170	171
36	Caucasian/White	3	1960	57	Male	50 ug MSP-1	14	14	pain at left hip	No	Not related	Mild	Dose not changed	Recovered/ resolved	30/09/2017	.	Pain in hip	Arthralgia	Musculoskeletal and connective tissue disorders	2	171	173
37	Caucasian/White	4	1987	30	Male	50 ug MSP-1	1	1	warmth at injection site	No	(Definitely) related	Mild	Dose not changed	Recovered/ resolved	19/04/2017	.	Injection site warmth	Injection site warmth	General disorders and administration site conditions	0	1	1
38	Caucasian/White	4	1987	30	Male	50 ug MSP-1	5	5	Diarrhea	No	Unlikely	Mild	Dose not changed	Recovered/ resolved	26/04/2017	.	Diarrhea	Diarrhoea	Gastrointestinal disorders	6	7	8
39	Caucasian/White	4	1987	30	Male	50 ug MSP-1	3	3	Induration at injection site	No	(Definitely) related	Mild	Dose not changed	Recovered/ resolved	24/05/2017	.	Injection site induration	Injection site induration	General disorders and administration site conditions	1	30	36
40	Caucasian/White	4	1987	30	Male	50 ug MSP-1	6	6	Diarrhea	No	Unlikely	Mild	Dose not changed	Recovered/ resolved	22/05/2017	.	Diarrhea	Diarrhoea	Gastrointestinal disorders	5	34	34
41	Caucasian/White	4	1987	30	Male	50 ug MSP-1	4	4	Headache	No	Unlikely	Mild	Dose not changed	Recovered/ resolved	31/05/2017	.	Headache	Headache	Nervous system disorders	14	43	43
42	Caucasian/White	4	1987	30	Male	50 ug MSP-1	7	7	warmth at injection site	No	(Definitely) related	Mild	Dose not changed	Recovered/ resolved	12/06/2017	.	Injection site warmth	Injection site warmth	General disorders and administration site conditions	0	55	55
43	Caucasian/White	4	1987	30	Male	50 ug MSP-1	8	8	common cold	No	Not related	Mild	Dose not changed	Recovered/ resolved	01/07/2017	.	Common cold	Nasopharyngitis	Infections and infestations	19	74	74
44	Caucasian/White	4	1987	30	Male	50 ug MSP-1	9	9	common cold	No	Possible	Mild	Dose not changed	Recovered/ resolved	10/10/2017	.	Common cold	Nasopharyngitis	Infections and infestations	5	166	175
45	Caucasian/White	4	1987	30	Male	50 ug MSP-1	10	10	common cold	No	Unlikely	Moderate	Dose not changed	Recovered/ resolved	27/10/2017	.	Common cold	Nasopharyngitis	Infections and infestations	24	185	192
46	Caucasian/White	5	1990	27	Female	Adjuvant	2	2	Erythema at injection site	No	(Definitely) related	Mild	Dose not changed	Recovered/ resolved	19/04/2017	.	Injection site erythema	Injection site erythema	General disorders and administration site conditions	0	1	1
47	Caucasian/White	5	1990	27	Female	Adjuvant	3	3	warmth at injection site	No	(Definitely) related	Mild	Dose not changed	Recovered/ resolved	19/04/2017	.	Injection site warmth	Injection site warmth	General disorders and administration site conditions	0	1	1
48	Caucasian/White	5	1990	27	Female	Adjuvant	4	4	sternal pain	No	Unlikely	Mild	Dose not changed	Recovered/ resolved	20/04/2017	.	Sternal pain	Chest pain	General disorders and administration site conditions	1	2	2
49	Caucasian/White	5	1990	27	Female	Adjuvant	6	6	sore throat	No	Unlikely	Mild	Dose not changed	Recovered/ resolved	01/05/2017	.	Sore throat	Oropharyngeal pain	Respiratory, thoracic and mediastinal disorders	12	13	13
50	Caucasian/White	5	1990	27	Female	Adjuvant	13	13	menstrual cramps	No	Not related	Mild	Dose not changed	Recovered/ resolved	06/05/2017	.	Menstrual cramps	Dysmenorrhoea	Reproductive system and breast disorders	17	18	18
51	Caucasian/White	5	1990	27	Female	Adjuvant	9	9	Hematoma at left antecubital fossa	No	Not related	Mild	Dose not changed	Recovered/ resolved	31/05/2017	.	Hematoma	Haematoma	Vascular disorders	0	29	43
52	Caucasian/White	5	1990	27	Female	Adjuvant	7	7	Nausea	No	Possible	Mild	Dose not changed	Recovered/ resolved	18/05/2017	.	Nausea	Nausea	Gastrointestinal disorders	1	30	30
53	Caucasian/White	5	1990	27	Female	Adjuvant	8	8	Pain at injection site	No	(Definitely) related	Mild	Dose not changed	Recovered/ resolved	19/05/2017	.	Injection site pain	Injection site pain	General disorders and administration site conditions	1	30	31
54	Caucasian/White	5	1990	27	Female	Adjuvant	10	10	Flatulence	No	Not related	Mild	Dose not changed	Recovered/ resolved	22/05/2017	.	Flatulence	Flatulence	Gastrointestinal disorders	4	33	34
55	Caucasian/White	5	1990	27	Female	Adjuvant	11	11	soft stool	No	Not related	Mild	Dose not changed	Recovered/ resolved	21/05/2017	.	Soft stools	Faeces soft	Gastrointestinal disorders	4	33	33
56	Caucasian/White	5	1990	27	Female	Adjuvant	12	12	Hematoma at lower left leg	No	Not related	Mild	Dose not changed	Recovered/ resolved	12/06/2017	.	Hematoma	Haematoma	Vascular disorders	9	38	55
57	Caucasian/White	5	1990	27	Female	Adjuvant	15	15	Tinea pedis (right foot)	No	Not related	Mild	Dose not changed	Recovered/ resolved	20/06/2017	.	Tinea pedis	Tinea pedis	Infections and infestations	16	45	63
58	Caucasian/White	5	1990	27	Female	Adjuvant	14	14	rash at right lower arm	No	Not related	Mild	Dose not changed	Recovered/ resolved	13/06/2017	.	Rash	Rash	Skin and subcutaneous tissue disorders	18	47	56
59	Caucasian/White	5	1990	27	Female	Adjuvant	16	16	pain at injection site	No	(Definitely) related	Mild	Dose not changed	Recovered/ resolved	14/06/2017	.	Injection site pain	Injection site pain	General disorders and administration site conditions	0	55	57
60	Caucasian/White	5	1990	27	Female	Adjuvant	18	18	fatigue	No	Possible	Mild	Dose not changed	Recovered/ resolved	12/06/2017	.	Fatigue	Fatigue	General disorders and administration site conditions	0	55	55
61	Caucasian/White	5	1990	27	Female	Adjuvant	17	17	sternal pain	No	Possible	Mild	Dose not changed	Recovered/ resolved	13/06/2017	.	Sternal pain	Chest pain	General disorders and administration site conditions	1	56	56
62	Caucasian/White	5	1990	27	Female	Adjuvant	26	26	hematoma at lower right leg	No	Possible	Mild	Dose not changed	Recovered/ resolved	23/06/2017	.	Hematoma	Haematoma	Vascular disorders	1	56	66
63	Caucasian/White	5	1990	27	Female	Adjuvant	22	22	rash maculo-papular-axillary (both sides)	No	Unlikely	Mild	Dose not changed	Recovered/ resolved	10/07/2017	.	Maculo-papular rash	Rash maculo-papular	Skin and subcutaneous tissue disorders	6	61	83
64	Caucasian/White	5	1990	27	Female	Adjuvant	19	19	Nausea	No	Possible	Mild	Dose not changed	Recovered/ resolved	21/06/2017	.	Nausea	Nausea	Gastrointestinal disorders	7	62	64
65	Caucasian/White	5	1990	27	Female	Adjuvant	20	20	Dizziness	No	Possible	Mild	Dose not changed	Recovered/ resolved	21/06/2017	.	Dizziness	Dizziness	Nervous system disorders	7	62	64
66	Caucasian/White	5	1990	27	Female	Adjuvant	21	21	Headache	No	Possible	Mild	Dose not changed	Recovered/ resolved	21/06/2017	.	Headache	Headache	Nervous system disorders	7	62	64
67	Caucasian/White	5	1990	27	Female	Adjuvant	24	24	headache	No	Possible	Mild	Dose not changed	Recovered/ resolved	04/07/2017	.	Headache	Headache	Nervous system disorders	22	77	77
68	Caucasian/White	5	1990	27	Female	Adjuvant	25	25	mild nausea	No	Possible	Mild	Dose not changed	Recovered/ resolved	04/07/2017	.	Nausea	Nausea	Gastrointestinal disorders	22	77	77
69	Caucasian/White	5	1990	27	Female	Adjuvant	23	23	dysmenorrhea	No	Unlikely	Mild	Dose not changed	Recovered/ resolved	06/07/2017	.	Dysmenorrhea	Dysmenorrhoea	Reproductive system and breast disorders	24	79	79
70	Caucasian/White	5	1990	27	Female	Adjuvant	27	27	hematoma left upper leg	No	Possible	Mild	Dose not changed	Recovered/ resolved	20/07/2017	.	Hematoma	Haematoma	Vascular disorders	28	83	93
71	Hispanic	6	1989	28	Female	50 ug MSP-1	1	1	Warmth at injection site	No	(Definitely) related	Mild	Dose not changed	Recovered/ resolved	19/04/2017	.	Injection site warmth	Injection site warmth	General disorders and administration site conditions	0	1	1
72	Hispanic	6	1989	28	Female	50 ug MSP-1	2	2	Pain at injection site	No	(Definitely) related	Mild	Dose not changed	Recovered/ resolved	21/04/2017	.	Injection site pain	Injection site pain	General disorders and administration site conditions	1	2	3
73	Hispanic	6	1989	28	Female	50 ug MSP-1	3	3	Fatigue	No	Possible	Mild	Dose not changed	Recovered/ resolved	21/04/2017	.	Fatigue	Fatigue	General disorders and administration site conditions	1	2	3

74	Hispanic	6	1989	28	Female	50 ug MSP-1	4.01	4	Sleep disturbance (due to pain at injection site)	No	(Definitely) related	Mild	Dose not changed	Recovered/ resolved	21/04/2017	1	Sleep disturbance	Sleep disorder	Psychiatric disorders	1	2	3
75	Hispanic	6	1989	28	Female	50 ug MSP-1	4.02	4	Sleep disturbance (due to pain at injection site)	No	(Definitely) related	Mild	Dose not changed	Recovered/ resolved	21/04/2017	2	Injection site pain	Injection site pain	General disorders and administration site conditions	1	2	3
76	Hispanic	6	1989	28	Female	50 ug MSP-1	5	5	Blood glucose decreased	No	Unlikely	Mild	Dose not changed	Recovered/ resolved	21/04/2017	.	Blood glucose decreased	Blood glucose decreased	Investigations	1	2	3
77	Hispanic	6	1989	28	Female	50 ug MSP-1	7	7	Sweating	No	Possible	Mild	Dose not changed	Recovered/ resolved	21/04/2017	.	Sweating	Hyperhidrosis	Skin and subcutaneous tissue disorders	1	2	3
78	Hispanic	6	1989	28	Female	50 ug MSP-1	9	9	Pruritus under the breast	No	Not related	Mild	Dose not changed	Recovered/ resolved	23/04/2017	.	Pruritus breast	Pruritus	Skin and subcutaneous tissue disorders	1	2	5
79	Hispanic	6	1989	28	Female	50 ug MSP-1	6	6	Diarrhea	No	Possible	Mild	Dose not changed	Recovered/ resolved	21/04/2017	.	Diarrhea	Diarrhoea	Gastrointestinal disorders	2	3	3
80	Hispanic	6	1989	28	Female	50 ug MSP-1	8	8	Fatigue	No	Possible	Mild	Dose not changed	Recovered/ resolved	25/04/2017	.	Fatigue	Fatigue	General disorders and administration site conditions	4	5	7
81	Hispanic	6	1989	28	Female	50 ug MSP-1	10	10	Chills	No	Possible	Mild	Dose not changed	Recovered/ resolved	24/04/2017	.	Chills	Chills	General disorders and administration site conditions	4	5	6
82	Hispanic	6	1989	28	Female	50 ug MSP-1	11	11	Itching at injection site	No	Possible	Mild	Dose not changed	Recovered/ resolved	24/04/2017	.	Injection site itching	Injection site pruritus	General disorders and administration site conditions	5	6	6
83	Hispanic	6	1989	28	Female	50 ug MSP-1	12	12	localized edema at injection site	No	Possible	Mild	Dose not changed	Recovered/ resolved	24/04/2017	.	Edema injection site	Injection site oedema	General disorders and administration site conditions	5	6	6
84	Hispanic	6	1989	28	Female	50 ug MSP-1	13	13	Erythema at injection site	No	Possible	Mild	Dose not changed	Recovered/ resolved	24/04/2017	.	Injection site erythema	Injection site erythema	General disorders and administration site conditions	5	6	6
85	Hispanic	6	1989	28	Female	50 ug MSP-1	14.01	14	Insect bite with surrounding erythema at right upperarm (contralateral to injection site)	No	Not related	Mild	Dose not changed	Recovered/ resolved	10/05/2017	1	Insect bite NOS	Arthropod bite	Injury, poisoning and procedural complications	5	6	22
86	Hispanic	6	1989	28	Female	50 ug MSP-1	14.02	14	Insect bite with surrounding erythema at right upperarm (contralateral to injection site)	No	Not related	Mild	Dose not changed	Recovered/ resolved	10/05/2017	2	Exanthema	Rash	Skin and subcutaneous tissue disorders	5	6	22
87	Hispanic	6	1989	28	Female	50 ug MSP-1	15	15	Pain at injection site	No	(Definitely) related	Moderate	Dose not changed	Recovered/ resolved	19/05/2017	.	Injection site pain	Injection site pain	General disorders and administration site conditions	1	30	31
88	Hispanic	6	1989	28	Female	50 ug MSP-1	17	17	Edema deltoid muscle	No	(Definitely) related	Mild	Dose not changed	Recovered/ resolved	25/05/2017	.	Muscle edema	Muscle oedema	Musculoskeletal and connective tissue disorders	1	30	37
89	Hispanic	6	1989	28	Female	50 ug MSP-1	18	18	local hyperthermia deltoid muscle	No	(Definitely) related	Mild	Dose not changed	Recovered/ resolved	19/05/2017	.	Hyperthermia	Hyperthermia	General disorders and administration site conditions	1	30	31
90	Hispanic	6	1989	28	Female	50 ug MSP-1	21	21	Erythema at injection site	No	(Definitely) related	Mild	Dose not changed	Recovered/ resolved	19/05/2017	.	Injection site erythema	Injection site erythema	General disorders and administration site conditions	1	30	31
91	Hispanic	6	1989	28	Female	50 ug MSP-1	23	23	Induration at injection site	No	(Definitely) related	Mild	Dose not changed	Recovered/ resolved	25/05/2017	.	Injection site induration	Injection site induration	General disorders and administration site conditions	1	30	37
92	Hispanic	6	1989	28	Female	50 ug MSP-1	41	41	Warmth at injection site	No	(Definitely) related	Mild	Dose not changed	Recovered/ resolved	19/05/2017	.	Injection site warmth	Injection site warmth	General disorders and administration site conditions	1	30	31
93	Hispanic	6	1989	28	Female	50 ug MSP-1	19	19	Hematoma forearm left	No	Unlikely	Mild	Dose not changed	Recovered/ resolved	28/05/2017	.	Hematoma	Haematoma	Vascular disorders	2	31	40
94	Hispanic	6	1989	28	Female	50 ug MSP-1	20	20	Pruritus at injection site	No	(Definitely) related	Mild	Dose not changed	Recovered/ resolved	23/05/2017	.	Injection site pruritus	Injection site pruritus	General disorders and administration site conditions	3	32	35
95	Hispanic	6	1989	28	Female	50 ug MSP-1	24	24	Headache	No	Possible	Mild	Dose not changed	Recovered/ resolved	20/05/2017	.	Headache	Headache	Nervous system disorders	3	32	32
96	Hispanic	6	1989	28	Female	50 ug MSP-1	22	22	Hematoma at injection site	No	(Definitely) related	Mild	Dose not changed	Recovered/ resolved	25/05/2017	.	Hematoma injection site	Injection site haematoma	General disorders and administration site conditions	6	35	37
97	Hispanic	6	1989	28	Female	50 ug MSP-1	25	25	breast pain	No	Unlikely	Mild	Dose not changed	Recovered/ resolved	13/06/2017	.	Breast pain	Breast pain	Reproductive system and breast disorders	25	54	56
98	Hispanic	6	1989	28	Female	50 ug MSP-1	26	26	pain at injection site	No	(Definitely) related	Mild	Dose not changed	Recovered/ resolved	13/06/2017	.	Injection site pain	Injection site pain	General disorders and administration site conditions	0	55	56
99	Hispanic	6	1989	28	Female	50 ug MSP-1	27	27	induration at injection site	No	(Definitely) related	Mild	Dose not changed	Recovered/ resolved	22/06/2017	.	Injection site induration	Injection site induration	General disorders and administration site conditions	0	55	65
100	Hispanic	6	1989	28	Female	50 ug MSP-1	28	28	localized edema at injection site	No	(Definitely) related	Mild	Dose not changed	Recovered/ resolved	19/06/2017	.	Edema injection site	Injection site oedema	General disorders and administration site conditions	0	55	62
101	Hispanic	6	1989	28	Female	50 ug MSP-1	29	29	Warmth at injection site	No	(Definitely) related	Mild	Dose not changed	Recovered/ resolved	22/06/2017	.	Injection site warmth	Injection site warmth	General disorders and administration site conditions	0	55	65
102	Hispanic	6	1989	28	Female	50 ug MSP-1	30	30	Erythema at injection site	No	(Definitely) related	Mild	Dose not changed	Recovered/ resolved	19/06/2017	.	Injection site erythema	Injection site erythema	General disorders and administration site conditions	1	56	62
103	Hispanic	6	1989	28	Female	50 ug MSP-1	31	31	Hematoma at injection site	No	(Definitely) related	Mild	Dose not changed	Recovered/ resolved	22/06/2017	.	Hematoma injection site	Injection site haematoma	General disorders and administration site conditions	1	56	65
104	Hispanic	6	1989	28	Female	50 ug MSP-1	32	32	pruritus at injection site	No	(Definitely) related	Mild	Dose not changed	Recovered/ resolved	16/06/2017	.	Injection site pruritus	Injection site pruritus	General disorders and administration site conditions	1	56	59
105	Hispanic	6	1989	28	Female	50 ug MSP-1	33	33	GOT increased	No	Possible	Mild	Dose not changed	Recovered/ resolved	19/06/2017	.	GOT increased	Aspartate aminotransferase increased	Investigations	2	57	62
106	Hispanic	6	1989	28	Female	50 ug MSP-1	35	35	headache	No	Possible	Mild	Dose not changed	Recovered/ resolved	17/06/2017	.	Headache	Headache	Nervous system disorders	4	59	60
107	Hispanic	6	1989	28	Female	50 ug MSP-1	34	34	rash maculo-papular	No	Possible	Mild	Dose not changed	Recovered/ resolved	10/07/2017	.	Rash maculo-papular	Rash maculo-papular	Skin and subcutaneous tissue disorders	5	60	83
108	Hispanic	6	1989	28	Female	50 ug MSP-1	36	36	stomach pain	No	Unlikely	Moderate	Dose not changed	Recovered/ resolved	22/06/2017	.	Stomach pain	Abdominal pain upper	Gastrointestinal disorders	9	64	65
109	Hispanic	6	1989	28	Female	50 ug MSP-1	37	37	Diarrhea	No	Unlikely	Mild	Dose not changed	Recovered/ resolved	22/06/2017	.	Diarrhea	Diarrhoea	Gastrointestinal disorders	9	64	65
110	Hispanic	6	1989	28	Female	50 ug MSP-1	38	38	Nausea	No	Unlikely	Mild	Dose not changed	Recovered/ resolved	22/06/2017	.	Nausea	Nausea	Gastrointestinal disorders	9	64	65
111	Hispanic	6	1989	28	Female	50 ug MSP-1	40	40	upper respiratory tract infection	No	Possible	Mild	Dose not changed	Recovered/ resolved	02/07/2017	.	Upper respiratory tract infection	Upper respiratory tract infection	Infections and infestations	18	73	75
112	Hispanic	6	1989	28	Female	50 ug MSP-1	39	39	hematoma right knee	No	Not related	Mild	Dose not changed	Recovered/ resolved	02/08/2017	.	Hematoma	Haematoma	Vascular disorders	23	78	106
113	Hispanic	6	1989	28	Female	50 ug MSP-1	42	42	Hematoma at upper and lower extremity	No	Not related	Mild	Dose not changed	Recovered/ resolved	28/09/2017	.	Hematoma	Haematoma	Vascular disorders	102	157	163
114	Hispanic	6	1989	28	Female	50 ug MSP-1	43	43	Pain at injection site	No	(Definitely) related	Mild	Dose not changed	Recovered/ resolved	26/09/2017	.	Injection site pain	Injection site pain	General disorders and administration site conditions	0	161	161
115	Hispanic	6	1989	28	Female	50 ug MSP-1	44	44	warmth at injection site	No	(Definitely) related	Mild	Dose not changed	Recovered/ resolved	26/09/2017	.	Injection site warmth	Injection site warmth	General disorders and administration site conditions	0	161	161
116	Hispanic	6	1989	28	Female	50 ug MSP-1	45	45	itching at injection site	No	(Definitely) related	Mild	Dose not changed	Recovered/ resolved	27/09/2017	.	Injection site itching	Injection site pruritus	General disorders and administration site conditions	0	161	162
117	Hispanic	6	1989	28	Female	50 ug MSP-1	48	48	Headache	No	Unlikely	Mild	Dose not changed	Recovered/ resolved	29/09/2017	.	Headache	Headache	Nervous system disorders	3	164	164
118	Hispanic	6	1989	28	Female	50 ug MSP-1	47.01	47	ache of head and stomach	No	Unlikely	Mild	Dose not changed	Recovered/ resolved	03/10/2017	1	Headache	Headache	Nervous system disorders	7	168	168
119	Hispanic	6	1989	28	Female	50 ug MSP-1	47.02	47	ache of head and stomach	No	Unlikely	Mild	Dose not changed	Recovered/ resolved	03/10/2017	2	Stomach ache	Abdominal pain upper	Gastrointestinal disorders	7	168	168
120	Hispanic	6	1989	28	Female	50 ug MSP-1	46	46	upper respiratory infection	No	Unlikely	Mild	Dose not changed	Recovered/ resolved	14/10/2017	.	Upper respiratory infection	Upper respiratory tract infection	Infections and infestations	13	174	179
121	Hispanic	6	1989	28	Female	50 ug MSP-1	50	50	Vitamin D defiency	No	Not related	Moderate	Not applicable	Unknown	.	.	Vitamin D deficiency	Vitamin D deficiency	Metabolism and nutrition disorders	36	197	.
122	Hispanic	6	1989	28	Female	50 ug MSP-1	49	49	Depression	No	Not related	Moderate	Not applicable	Recovering/ resolving	.	.	Depression	Depression	Psychiatric disorders	97	258	.
123	Caucasian/White	7	1989	28	Male	50 ug MSP-1	3	3	warmth at injection site	No	(Definitely) related	Mild	Dose not changed	Recovered/ resolved	20/04/2017	.	Injection site warmth	Injection site warmth	General disorders and administration site conditions	0	1	2
124	Caucasian/White	7	1989	28	Male	50 ug MSP-1	4	4	Erythema at injection site	No	(Definitely) related	Mild	Dose not changed	Recovered/ resolved	20/04/2017	.	Injection site erythema	Injection site erythema	General disorders and administration site conditions	0	1	2
125	Caucasian/White	7	1989	28	Male	50 ug MSP-1	5	5	Pain at injection site	No	(Definitely) related	Mild	Dose not changed	Recovered/ resolved	22/04/2017	.	Injection site pain	Injection site pain	General disorders and administration site conditions	0	1	4
126	Caucasian/White	7	1989	28	Male	50 ug MSP-1	6	6	Induration at injection site	No	(Definitely) related	Mild	Dose not changed	Recovered/ resolved	20/04/2017	.	Injection site induration	Injection site induration	General disorders and administration site conditions	0	1	2
127	Caucasian/White	7	1989	28	Male	50 ug MSP-1	7	7	Contact dermatitis at antecubital fossa (due to adhesive bandage)	No	Not related	Mild	Dose not changed	Recovered/ resolved	24/04/2017	.	Contact dermatitis	Dermatitis contact	Skin and subcutaneous tissue disorders	1	2	6
128	Caucasian/White	7	1989	28	Male	50 ug MSP-1	9	9	abdominal pain, intermittent	No	Possible	Mild	Dose not changed	Recovered/ resolved	21/05/2017	.	Abdominal pain	Abdominal pain	Gastrointestinal disorders	2	3	33
129	Caucasian/White	7	1989	28	Male	50 ug MSP-1	10	10	diarrhea, intermittent	No	Possible	Mild	Dose not changed	Recovered/ resolved	05/07/2017	.	Diarrhea	Diarrhoea	Gastrointestinal disorders	2	3	78
130	Caucasian/White	7	1989	28	Male	50 ug MSP-1	12	12	nausea	No	Unlikely	Mild	Dose not changed	Recovered/ resolved	22/04/2017	.	Nausea	Nausea	Gastrointestinal disorders	2	3	4
131	Caucasian/White	7	1989	28	Male	50 ug MSP-1	29	29	Fatigue	No	Possible	Mild	Dose not changed	Recovered/ resolved	24/04/2017	.	Fatigue	Fatigue	General disorders and administration site conditions	4	5	6
132	Caucasian/White	7	1989	28	Male	50 ug MSP-1	30	30	Headache	No	Possible	Mild	Dose not changed	Recovered/ resolved	26/04/2017	.	Headache	Headache	Nervous system disorders	6	7	8
133	Caucasian/White	7	1989	28	Male	50 ug MSP-1	8	8	hematona at injection site	No	(Definitely) related	Mild	Dose not changed	Recovered/ resolved	03/05/2017	.	Hematoma injection site	Injection site haematoma	General disorders and administration site conditions	7	8	15
134	Caucasian/White	7	1989	28	Male	50 ug MSP-1	11	11	fatigue	No	Unlikely	Mild	Dose not changed	Recovered/ resolved	02/05/2017	.	Fatigue	Fatigue	General disorders and administration site conditions	12	13	14
135	Caucasian/White	7	1989	28	Male	50 ug MSP-1	16	16	papulae at right lumbar region	No	Not related	Mild	Dose not changed	Recovered/ resolved	03/06/2017	.	Papular rash	Rash papular	Skin and subcutaneous tissue disorders	17	18	46
136	Caucasian/White	7	1989	28	Male	50 ug MSP-1	13	13	cold symptoms	No	Unlikely	Mild	Dose not changed	Recovered/ resolved	14/05/2017	.	Cold symptoms	Nasopharyngitis	Infections and infestations	24	25	26
137	Caucasian/White	7	1989	28	Male	50 ug MSP-1	14	14	CRP increased	No	Unlikely	Mild	Dose not changed	Recovered/ resolved	29/05/2017	.	CRP increased	C-reactive protein increased	Investigations	27	28	41
138	Caucasian/White	7	1989	28	Male	50 ug MSP-1	15	15	nasal congestion	No	Unlikely	Mild	Dose not changed	Recovered/ resolved	20/05/2017	.	Nasal congestion	Nasal congestion	Respiratory, thoracic and mediastinal disorders	28	29	32
139	Caucasian/White	7	1989	28	Male	50 ug MSP-1	19	19	Pain at injection site	No	(Definitely) related	Mild	Dose not changed	Recovered/ resolved	07/06/2017	.	Injection site pain	Injection site pain	General disorders and administration site conditions	0	43	50
140	Caucasian/White	7	1989	28	Male	50 ug MSP-1	17	17	induration at injection site	No	(Definitely) related	Mild	Dose not changed	Recovered/ resolved	07/06/2017	.	Injection site induration	Injection site induration	General disorders and administration site conditions	1	44	50
141	Caucasian/White	7	1989	28	Male	50 ug MSP-1	18	18	Warmth at injection site	No	(Definitely) related	Mild	Dose not changed	Recovered/ resolved	07/06/2017	.	Injection site warmth	Injection site warmth	General disorders and administration site conditions	1	44	50
142	Caucasian/White	7	1989	28	Male	50 ug MSP-1	23	23	Pruritus at injection site	No	(Definitely) related	Mild	Dose not changed	Recovered/ resolved	02/06/2017	.	Injection site pruritus	Injection site pruritus	General disorders and administration site conditions	1	44	45
143	Caucasian/White	7	1989	28	Male	50 ug MSP-1	20	20	Fatigue intermittent	No	Possible	Mild	Dose not changed	Recovered/ resolved	24/07/2017	.	Fatigue	Fatigue	General disorders and administration site conditions	2	45	97
144	Caucasian/White	7	1989	28	Male	50 ug MSP-1	21	21	Headache	No	Possible	Mild	Dose not changed	Recovered/ resolved	03/06/2017	.	Headache	Headache	Nervous system disorders	2	45	46
145	Caucasian/White	7	1989	28	Male	50 ug MSP-1	22	22	Pyuria	No	Possible	Moderate	Dose not changed	Recovered/ resolved	16/06/2017	.	Pyuria	Pyuria	Infections and infestations	14	57	59
146	Caucasian/White	7	1989	28	Male	50 ug MSP-1	24	24	CRP increased	No	Unlikely	Mild	Dose not changed	Recovered/ resolved	05/07/2017	.	CRP increased	C-reactive protein increased	Investigations	26	69	78
147	Caucasian/White	7	1989	28	Male	50 ug MSP-1	25	25	Warmth at injection site	No	(Definitely) related	Mild	Dose not changed	Recovered/ resolved	29/06/2017	.	Injection site warmth	Injection site warmth	General disorders and administration site conditions	0	71	72
148	Caucasian/White	7	1989	28	Male	50 ug MSP-1	26	26	induration at injection site	No	(Definitely) related	Mild	Dose not changed	Recovered/ resolved	29/06/2017	.	Injection site induration	Injection site induration	General disorders and administration site conditions	0	71	72
149	Caucasian/White	7	1989	28	Male	50 ug MSP-1	27	27	increase in serum creatinine	No	Unlikely	Mild	Dose not changed	Recovered/ resolved	24/07/2017	.	Increased serum creatinine	Blood creatinine increased	Investigations	21	92	97

150	Caucasian/White	7	1989	28	Male	50 ug MSP-1	28	28	upper respiratory infection	No	Unlikely	Mild	Dose not changed	Recovered/ resolved	24/07/2017	.	Upper respiratory infection	Upper respiratory tract infection	Infections and infestations	24	95	97
151	Caucasian/White	8	1976	41	Male	Placebo	2	2	Warmth at injection site	No	(Definitely) related	Mild	Dose not changed	Recovered/ resolved	25/04/2017	.	Injection site warmth	Injection site warmth	General disorders and administration site conditions	0	1	1
152	Caucasian/White	8	1976	41	Male	Placebo	3	3	Erythema at injection site	No	(Definitely) related	Mild	Dose not changed	Recovered/ resolved	25/04/2017	.	Injection site erythema	Injection site erythema	General disorders and administration site conditions	0	1	1
153	Caucasian/White	8	1976	41	Male	Placebo	4	4	Herpes labialis	No	Possible	Mild	Dose not changed	Recovered/ resolved	13/05/2017	.	Herpes labialis	Oral herpes	Infections and infestations	7	8	19
154	Caucasian/White	8	1976	41	Male	Placebo	5	5	Induration at injection site	No	(Definitely) related	Mild	Dose not changed	Recovered/ resolved	23/05/2017	.	Injection site induration	Injection site induration	General disorders and administration site conditions	0	28	29
155	Caucasian/White	8	1976	41	Male	Placebo	6	6	Warmth at injection site	No	(Definitely) related	Mild	Dose not changed	Recovered/ resolved	30/05/2017	.	Injection site warmth	Injection site warmth	General disorders and administration site conditions	2	30	36
156	Caucasian/White	8	1976	41	Male	Placebo	7	7	Erythematous macula (left axillary)	No	Not related	Mild	Dose not changed	Recovered/ resolved	04/07/2017	.	Erythematous skin rash	Rash erythematous	Skin and subcutaneous tissue disorders	28	56	71
157	Caucasian/White	9	1963	54	Female	50 ug MSP-1	1	1	Erythema at injection site	No	(Definitely) related	Mild	Dose not changed	Recovered/ resolved	25/04/2017	.	Injection site erythema	Injection site erythema	General disorders and administration site conditions	0	1	1
158	Caucasian/White	9	1963	54	Female	50 ug MSP-1	2	2	Erythema at injection site	No	(Definitely) related	Mild	Dose not changed	Recovered/ resolved	29/05/2017	.	Injection site erythema	Injection site erythema	General disorders and administration site conditions	0	28	35
159	Caucasian/White	9	1963	54	Female	50 ug MSP-1	3	3	Induration at injection site	No	(Definitely) related	Mild	Dose not changed	Recovered/ resolved	29/05/2017	.	Injection site induration	Injection site induration	General disorders and administration site conditions	1	29	35
160	Caucasian/White	9	1963	54	Female	50 ug MSP-1	4	4	localized edema at injection site	No	(Definitely) related	Mild	Dose not changed	Recovered/ resolved	24/05/2017	.	Edema injection site	Injection site oedema	General disorders and administration site conditions	1	29	30
161	Caucasian/White	9	1963	54	Female	50 ug MSP-1	5	5	Warmth at injection site	No	(Definitely) related	Mild	Dose not changed	Recovered/ resolved	29/05/2017	.	Injection site warmth	Injection site warmth	General disorders and administration site conditions	1	29	35
162	Caucasian/White	9	1963	54	Female	50 ug MSP-1	6	6	Warmth at injection site	No	(Definitely) related	Mild	Dose not changed	Recovered/ resolved	22/06/2017	.	Injection site warmth	Injection site warmth	General disorders and administration site conditions	1	58	59
163	Caucasian/White	9	1963	54	Female	50 ug MSP-1	7	7	Warmth at injection site	No	(Definitely) related	Mild	Dose not changed	Recovered/ resolved	30/10/2017	.	Injection site warmth	Injection site warmth	General disorders and administration site conditions	1	183	189
164	Caucasian/White	9	1963	54	Female	50 ug MSP-1	8	8	Erythema at inejtion site	No	(Definitely) related	Mild	Dose not changed	Recovered/ resolved	30/10/2017	.	Injection site erythema	Injection site erythema	General disorders and administration site conditions	1	183	189
165	Caucasian/White	9	1963	54	Female	50 ug MSP-1	9	9	Induration at injection site	No	(Definitely) related	Mild	Dose not changed	Recovered/ resolved	25/10/2017	.	Injection site induration	Injection site induration	General disorders and administration site conditions	1	183	184
166	Caucasian/White	9	1963	54	Female	50 ug MSP-1	10	10	Back Pain	No	Not related	Moderate	Not applicable	Recovered/ resolved	01/01/2018	.	Back pain	Back pain	Musculoskeletal and connective tissue disorders	70	252	252
167	Caucasian/White	10	1994	23	Male	50 ug MSP-1	1	1	Hematoma at injection site	No	(Definitely) related	Mild	Dose not changed	Recovered/ resolved	11/05/2017	.	Hematoma injection site	Injection site haematoma	General disorders and administration site conditions	7	8	15
168	Caucasian/White	10	1994	23	Male	50 ug MSP-1	2	2	Headache	No	Unlikely	Mild	Dose not changed	Recovered/ resolved	20/05/2017	.	Headache	Headache	Nervous system disorders	23	24	24
169	Caucasian/White	10	1994	23	Male	50 ug MSP-1	3	3	Agitation	No	Unlikely	Mild	Dose not changed	Recovered/ resolved	22/05/2017	.	Agitation	Agitation	Psychiatric disorders	0	26	26
170	Caucasian/White	10	1994	23	Male	50 ug MSP-1	4	4	Hypertension	No	Unlikely	Severe	Dose not changed	Recovered/ resolved	22/05/2017	.	Hypertension	Hypertension	Vascular disorders	0	26	26
171	Caucasian/White	10	1994	23	Male	50 ug MSP-1	5	5	lymph node pain left axilla	No	Possible	Mild	Dose not changed	Recovered/ resolved	26/05/2017	.	Lymph node pain	Lymph node pain	Blood and lymphatic system disorders	2	28	30
172	Caucasian/White	10	1994	23	Male	50 ug MSP-1	6	6	GPT increased	No	Possible	Mild	Dose not changed	Recovered/ resolved	06/06/2017	.	GPT increased	Alanine aminotransferase increased	Investigations	2	28	41
173	Caucasian/White	10	1994	23	Male	50 ug MSP-1	7	7	lymph node induration	No	Possible	Mild	Dose not changed	Recovered/ resolved	06/06/2017	.	Lymph nodes enlarged	Lymphadenopathy	Blood and lymphatic system disorders	7	33	41
174	Caucasian/White	10	1994	23	Male	50 ug MSP-1	8	8	blood bilirubin increased, intermittent	No	Possible	Moderate	Dose not changed	Not recovered/ not resolved	.	.	Blood bilirubin increased	Blood bilirubin increased	Investigations	15	41	.
175	Caucasian/White	10	1994	23	Male	50 ug MSP-1	11	11	Induration at injection site	No	(Definitely) related	Mild	Dose not changed	Recovered/ resolved	22/06/2017	.	Injection site induration	Injection site induration	General disorders and administration site conditions	0	55	57
176	Caucasian/White	10	1994	23	Male	50 ug MSP-1	9	9	lymph node induration	No	Probable	Mild	Dose not changed	Recovered/ resolved	27/06/2017	.	Lymph nodes enlarged	Lymphadenopathy	Blood and lymphatic system disorders	1	56	62
177	Caucasian/White	10	1994	23	Male	50 ug MSP-1	10	10	warmth at injection site	No	(Definitely) related	Mild	Dose not changed	Recovered/ resolved	22/06/2017	.	Injection site warmth	Injection site warmth	General disorders and administration site conditions	1	56	57
178	Caucasian/White	10	1994	23	Male	50 ug MSP-1	12	12	Erythema at injection site	No	(Definitely) related	Mild	Dose not changed	Recovered/ resolved	22/06/2017	.	Injection site erythema	Injection site erythema	General disorders and administration site conditions	1	56	57
179	Caucasian/White	10	1994	23	Male	50 ug MSP-1	13	13	pectoral papulae at both sides	No	Unlikely	Mild	Dose not changed	Recovered/ resolved	29/06/2017	.	Papular rash	Rash papular	Skin and subcutaneous tissue disorders	7	62	64
180	Caucasian/White	10	1994	23	Male	50 ug MSP-1	14	14	wasp sting left hand	No	Not related	Mild	Dose not changed	Recovered/ resolved	20/07/2017	.	Wasp sting	Arthropod sting	Injury, poisoning and procedural complications	26	81	85
181	Caucasian/White	11	1996	21	Female	50 ug MSP-1	1	1	Headache	No	Possible	Mild	Dose not changed	Recovered/ resolved	02/05/2017	.	Headache	Headache	Nervous system disorders	0	1	1
182	Caucasian/White	11	1996	21	Female	50 ug MSP-1	4	4	contact dermatitis at left antecubital fossa (due to adhesire bandage)	No	Not related	Mild	Dose not changed	Recovered/ resolved	09/05/2017	.	Contact dermatitis	Dermatitis contact	Skin and subcutaneous tissue disorders	0	1	8
183	Caucasian/White	11	1996	21	Female	50 ug MSP-1	2	2	Pain at injection site	No	(Definitely) related	Mild	Dose not changed	Recovered/ resolved	04/05/2017	.	Injection site pain	Injection site pain	General disorders and administration site conditions	1	2	3
184	Caucasian/White	11	1996	21	Female	50 ug MSP-1	3	3	Fatigue	No	Possible	Mild	Dose not changed	Recovered/ resolved	05/05/2017	.	Fatigue	Fatigue	General disorders and administration site conditions	1	2	4
185	Caucasian/White	11	1996	21	Female	50 ug MSP-1	5	5	Hematoma at left antecubital fossa	No	Not related	Mild	Dose not changed	Recovered/ resolved	19/05/2017	.	Hematoma	Haematoma	Vascular disorders	7	8	18
186	Caucasian/White	11	1996	21	Female	50 ug MSP-1	6	6	GPT increased	No	Possible	Mild	Dose not changed	Recovered/ resolved	29/05/2017	.	GPT increased	Alanine aminotransferase increased	Investigations	14	15	28
187	Caucasian/White	11	1996	21	Female	50 ug MSP-1	7	7	Itching at injection site	No	(Definitely) related	Mild	Dose not changed	Recovered/ resolved	30/05/2017	.	Injection site itching	Injection site pruritus	General disorders and administration site conditions	0	29	29
188	Caucasian/White	11	1996	21	Female	50 ug MSP-1	8	8	Induration at injection site	No	(Definitely) related	Mild	Dose not changed	Recovered/ resolved	01/06/2017	.	Injection site induration	Injection site induration	General disorders and administration site conditions	0	29	31
189	Caucasian/White	11	1996	21	Female	50 ug MSP-1	9	9	Pain at injection site	No	(Definitely) related	Mild	Dose not changed	Recovered/ resolved	06/06/2017	.	Injection site pain	Injection site pain	General disorders and administration site conditions	0	29	36
190	Caucasian/White	11	1996	21	Female	50 ug MSP-1	12	12	contact dermatitis at right antecubital fossa (due to adhesive bandage)	No	Not related	Mild	Dose not changed	Recovered/ resolved	08/06/2017	.	Contact dermatitis	Dermatitis contact	Skin and subcutaneous tissue disorders	0	29	38
191	Caucasian/White	11	1996	21	Female	50 ug MSP-1	10	10	Hematoma at injection site	No	(Definitely) related	Mild	Dose not changed	Recovered/ resolved	10/06/2017	.	Hematoma injection site	Injection site haematoma	General disorders and administration site conditions	1	30	40
192	Caucasian/White	11	1996	21	Female	50 ug MSP-1	11	11	Warmth at injection site	No	(Definitely) related	Mild	Dose not changed	Recovered/ resolved	01/06/2017	.	Injection site warmth	Injection site warmth	General disorders and administration site conditions	1	30	31
193	Caucasian/White	11	1996	21	Female	50 ug MSP-1	13	13	Fatigue	No	Possible	Mild	Dose not changed	Recovered/ resolved	01/06/2017	.	Fatigue	Fatigue	General disorders and administration site conditions	1	30	31
194	Caucasian/White	11	1996	21	Female	50 ug MSP-1	14	14	Headache	No	Possible	Moderate	Dose not changed	Recovered/ resolved	31/05/2017	.	Headache	Headache	Nervous system disorders	1	30	30
195	Caucasian/White	11	1996	21	Female	50 ug MSP-1	15	15	Nausea	No	Possible	Mild	Dose not changed	Recovered/ resolved	02/06/2017	.	Nausea	Nausea	Gastrointestinal disorders	2	31	32
196	Caucasian/White	11	1996	21	Female	50 ug MSP-1	16	16	Headache	No	Possible	Mild	Dose not changed	Recovered/ resolved	05/06/2017	.	Headache	Headache	Nervous system disorders	6	35	35
197	Caucasian/White	11	1996	21	Female	50 ug MSP-1	17	17	Hypotension	No	Possible	Moderate	Dose not changed	Recovered/ resolved	06/06/2017	.	Hypotension	Hypotension	Vascular disorders	7	36	36
198	Caucasian/White	11	1996	21	Female	50 ug MSP-1	18	18	Itching at injection site	No	(Definitely) related	Mild	Dose not changed	Recovered/ resolved	27/06/2017	.	Injection site itching	Injection site pruritus	General disorders and administration site conditions	0	57	57
199	Caucasian/White	11	1996	21	Female	50 ug MSP-1	19	19	Pain at injection site	No	(Definitely) related	Mild	Dose not changed	Recovered/ resolved	29/06/2017	.	Injection site pain	Injection site pain	General disorders and administration site conditions	0	57	59
200	Caucasian/White	11	1996	21	Female	50 ug MSP-1	22	22	Excoriation at right lumbar region	No	Not related	Mild	Dose not changed	Recovered/ resolved	04/07/2017	.	Excoriation	Excoriation	Injury, poisoning and procedural complications	0	57	64
201	Caucasian/White	11	1996	21	Female	50 ug MSP-1	20	20	warmth at injection site	No	(Definitely) related	Mild	Dose not changed	Recovered/ resolved	29/06/2017	.	Injection site warmth	Injection site warmth	General disorders and administration site conditions	1	58	59
202	Caucasian/White	11	1996	21	Female	50 ug MSP-1	21	21	Induration at injection site	No	(Definitely) related	Mild	Dose not changed	Recovered/ resolved	29/06/2017	.	Injection site induration	Injection site induration	General disorders and administration site conditions	1	58	59
203	Caucasian/White	11	1996	21	Female	50 ug MSP-1	23	23	wakulo papulous exanthema	No	Possible	Mild	Dose not changed	Recovered/ resolved	10/07/2017	.	Maculo-papular exanthema	Rash maculo-papular	Skin and subcutaneous tissue disorders	6	63	70
204	Caucasian/White	12	1980	37	Male	50 ug MSP-1	1	1	contact dermatitis at left antecubital (due to adhesive bandage)	No	Not related	Mild	Dose not changed	Recovered/ resolved	18/05/2017	.	Contact dermatitis	Dermatitis contact	Skin and subcutaneous tissue disorders	0	1	17
205	Caucasian/White	12	1980	37	Male	50 ug MSP-1	2	2	Pyrosis, intermittent	No	Not related	Mild	Dose not changed	Recovered/ resolved	21/10/2017	.	Pyrosis	Dyspepsia	Gastrointestinal disorders	1	2	173
206	Caucasian/White	12	1980	37	Male	50 ug MSP-1	4	4	cold symptoms	No	Unlikely	Mild	Dose not changed	Recovered/ resolved	06/06/2017	.	Cold symptoms	Nasopharyngitis	Infections and infestations	26	27	36
207	Caucasian/White	12	1980	37	Male	50 ug MSP-1	3	3	Platelet count decreased	No	Unlikely	Mild	Dose not changed	Recovered/ resolved	31/05/2017	.	Platelet count decreased	Platelet count decreased	Investigations	0	28	30
208	Caucasian/White	12	1980	37	Male	50 ug MSP-1	5	5	Fatigue	No	Possible	Mild	Dose not changed	Recovered/ resolved	29/05/2017	.	Fatigue	Fatigue	General disorders and administration site conditions	0	28	28
209	Caucasian/White	12	1980	37	Male	50 ug MSP-1	6	6	Induration at injection site	No	(Definitely) related	Mild	Dose not changed	Recovered/ resolved	01/06/2017	.	Injection site induration	Injection site induration	General disorders and administration site conditions	0	28	31
210	Caucasian/White	12	1980	37	Male	50 ug MSP-1	7	7	Pain at injection site	No	(Definitely) related	Mild	Dose not changed	Recovered/ resolved	30/05/2017	.	Injection site pain	Injection site pain	General disorders and administration site conditions	0	28	29
211	Caucasian/White	12	1980	37	Male	50 ug MSP-1	8	8	Erythema at injection site	No	(Definitely) related	Mild	Dose not changed	Recovered/ resolved	31/05/2017	.	Injection site erythema	Injection site erythema	General disorders and administration site conditions	1	29	30
212	Caucasian/White	12	1980	37	Male	50 ug MSP-1	9	9	Warmth at injection site	No	(Definitely) related	Mild	Dose not changed	Recovered/ resolved	06/06/2017	.	Injection site warmth	Injection site warmth	General disorders and administration site conditions	1	29	36
213	Caucasian/White	12	1980	37	Male	50 ug MSP-1	10	10	Lymph node enlargement	No	Unlikely	Mild	Dose not changed	Recovered/ resolved	06/06/2017	.	Lymph nodes enlarged	Lymphadenopathy	Blood and lymphatic system disorders	1	29	36
214	Caucasian/White	12	1980	37	Male	50 ug MSP-1	11	11	excessive sweating	No	Possible	Mild	Dose not changed	Recovered/ resolved	31/05/2017	.	Sweating increased	Hyperhidrosis	Skin and subcutaneous tissue disorders	1	29	30
215	Caucasian/White	12	1980	37	Male	50 ug MSP-1	12	12	CRP increased	No	Possible	Mild	Dose not changed	Recovered/ resolved	06/06/2017	.	CRP increased	C-reactive protein increased	Investigations	2	30	36
216	Caucasian/White	12	1980	37	Male	50 ug MSP-1	13	13	back pain	No	Unlikely	Mild	Dose not changed	Recovered/ resolved	23/06/2017	.	Back pain	Back pain	Musculoskeletal and connective tissue disorders	25	53	53
217	Caucasian/White	12	1980	37	Male	50 ug MSP-1	14	14	pain at injection site	No	(Definitely) related	Mild	Dose not changed	Recovered/ resolved	29/06/2017	.	Injection site pain	Injection site pain	General disorders and administration site conditions	0	57	59
218	Caucasian/White	12	1980	37	Male	50 ug MSP-1	17	17	Induration at injection site	No	(Definitely) related	Mild	Dose not changed	Recovered/ resolved	28/06/2017	.	Injection site induration	Injection site induration	General disorders and administration site conditions	0	57	58
219	Caucasian/White	12	1980	37	Male	50 ug MSP-1	15	15	warmth at injection site	No	(Definitely) related	Mild	Dose not changed	Recovered/ resolved	03/07/2017	.	Injection site warmth	Injection site warmth	General disorders and administration site conditions	1	58	63
220	Caucasian/White	12	1980	37	Male	50 ug MSP-1	16	16	Erythema at injection site	No	(Definitely) related	Mild	Dose not changed	Recovered/ resolved	29/06/2017	.	Injection site erythema	Injection site erythema	General disorders and administration site conditions	1	58	59
221	Caucasian/White	12	1980	37	Male	50 ug MSP-1	18	18	CRP increased	No	Possible	Mild	Dose not changed	Recovered/ resolved	03/07/2017	.	CRP increased	C-reactive protein increased	Investigations	2	59	63
222	Caucasian/White	12	1980	37	Male	50 ug MSP-1	19	19	Back pain	No	Unlikely	Mild	Dose not changed	Recovered/ resolved	20/07/2017	.	Back pain	Back pain	Musculoskeletal and connective tissue disorders	23	80	80
223	Caucasian/White	12	1980	37	Male	50 ug MSP-1	20	20	Pressure at injection site	No	(Definitely) related	Mild	Dose not changed	Recovered/ resolved	24/10/2017	.	Injection site pressure sensation	Injection site discomfort	General disorders and administration site conditions	0	175	176
224	Caucasian/White	12	1980	37	Male	50 ug MSP-1	21	21	Warmth at injection site	No	(Definitely) related	Mild	Dose not changed	Recovered/ resolved	26/10/2017	.	Injection site warmth	Injection site warmth	General disorders and administration site conditions	0	175	178
225	Caucasian/White	12	1980	37	Male	50 ug MSP-1	22	22	CRP increase	No	(Definitely) related	Mild	Dose not changed	Recovered/ resolved	30/10/2017	.	C-reactive protein increased	C-reactive protein increased	Investigations	1	176	182

226	Caucasian/White	12	1980	37	Male	50 ug MSP-1	24	24	Induration at injection site	No	(Definitely) related	Mild	Dose not changed	Recovered/ resolved	25/10/2017	.	Injection site induration	Injection site induration	General disorders and administration site conditions	1	176	177
227	Caucasian/White	12	1980	37	Male	50 ug MSP-1	23	23	CRP increase	No	Possible	Mild	Dose not changed	Recovered/ resolved	20/11/2017	.	C-reactive protein increased	C-reactive protein increased	Investigations	14	189	203
228	Asian	13	1971	46	Female	50 ug MSP-1	1	1	Headache	No	Possible	Mild	Dose not changed	Recovered/ resolved	02/05/2017	.	Headache	Headache	Nervous system disorders	0	1	1
229	Asian	13	1971	46	Female	50 ug MSP-1	2	2	Warmth at injection site, intermittent	No	(Definitely) related	Mild	Dose not changed	Recovered/ resolved	05/05/2017	.	Injection site warmth	Injection site warmth	General disorders and administration site conditions	0	1	4
230	Asian	13	1971	46	Female	50 ug MSP-1	3	3	Pain at injection site	No	(Definitely) related	Mild	Dose not changed	Recovered/ resolved	03/05/2017	.	Injection site pain	Injection site pain	General disorders and administration site conditions	0	1	2
231	Asian	13	1971	46	Female	50 ug MSP-1	7	5	Itching at injection site	No	(Definitely) related	Mild	Dose not changed	Recovered/ resolved	04/05/2017	.	Injection site itching	Injection site pruritus	General disorders and administration site conditions	1	2	3
232	Asian	13	1971	46	Female	50 ug MSP-1	4	4	Induration at injection site, intermittent	No	(Definitely) related	Mild	Dose not changed	Recovered/ resolved	15/05/2017	.	Injection site induration	Injection site induration	General disorders and administration site conditions	2	3	14
233	Asian	13	1971	46	Female	50 ug MSP-1	5	6	Headache	No	Unlikely	Mild	Dose not changed	Recovered/ resolved	08/05/2017	.	Headache	Headache	Nervous system disorders	6	7	7
234	Asian	13	1971	46	Female	50 ug MSP-1	6	7	Pain at injection site	No	(Definitely) related	Mild	Dose not changed	Recovered/ resolved	15/05/2017	.	Injection site pain	Injection site pain	General disorders and administration site conditions	7	8	14
235	Asian	13	1971	46	Female	50 ug MSP-1	8	8	Induration at injection site	No	(Definitely) related	Mild	Dose not changed	Recovered/ resolved	03/06/2017	.	Injection site induration	Injection site induration	General disorders and administration site conditions	0	28	33
236	Asian	13	1971	46	Female	50 ug MSP-1	9	9	Pain at injection site	No	(Definitely) related	Mild	Dose not changed	Recovered/ resolved	30/05/2017	.	Injection site pain	Injection site pain	General disorders and administration site conditions	0	28	29
237	Asian	13	1971	46	Female	50 ug MSP-1	10	10	Erythema at injection site	No	(Definitely) related	Mild	Dose not changed	Recovered/ resolved	03/06/2017	.	Injection site erythema	Injection site erythema	General disorders and administration site conditions	1	29	33
238	Asian	13	1971	46	Female	50 ug MSP-1	11	11	Itching at injection site	No	(Definitely) related	Mild	Dose not changed	Recovered/ resolved	03/06/2017	.	Injection site itching	Injection site pruritus	General disorders and administration site conditions	1	29	33
239	Asian	13	1971	46	Female	50 ug MSP-1	12	12	Edema at injection site	No	(Definitely) related	Mild	Dose not changed	Recovered/ resolved	02/06/2017	.	Edema injection site	Injection site oedema	General disorders and administration site conditions	1	29	32
240	Asian	13	1971	46	Female	50 ug MSP-1	13	13	Warmth at injection site	No	(Definitely) related	Mild	Dose not changed	Recovered/ resolved	02/06/2017	.	Injection site warmth	Injection site warmth	General disorders and administration site conditions	1	29	32
241	Asian	13	1971	46	Female	50 ug MSP-1	14	14	headache	No	Possible	Mild	Dose not changed	Recovered/ resolved	30/05/2017	.	Headache	Headache	Nervous system disorders	1	29	29
242	Asian	13	1971	46	Female	50 ug MSP-1	15	15	Hematoma at injection site	No	(Definitely) related	Mild	Dose not changed	Recovered/ resolved	13/06/2017	.	Hematoma injection site	Injection site haematoma	General disorders and administration site conditions	8	36	43
243	Asian	13	1971	46	Female	50 ug MSP-1	16	16	sore throat	No	Unlikely	Mild	Dose not changed	Recovered/ resolved	29/06/2017	.	Sore throat	Oropharyngeal pain	Respiratory, thoracic and mediastinal disorders	14	42	59
244	Asian	13	1971	46	Female	50 ug MSP-1	17	17	warmth at injection site	No	(Definitely) related	Mild	Dose not changed	Recovered/ resolved	29/06/2017	.	Injection site warmth	Injection site warmth	General disorders and administration site conditions	0	57	59
245	Asian	13	1971	46	Female	50 ug MSP-1	18	18	Pruritus at injection site	No	(Definitely) related	Mild	Dose not changed	Recovered/ resolved	30/06/2017	.	Injection site pruritus	Injection site pruritus	General disorders and administration site conditions	0	57	60
246	Asian	13	1971	46	Female	50 ug MSP-1	19	19	Pain at injection site	No	(Definitely) related	Moderate	Dose not changed	Recovered/ resolved	30/06/2017	.	Injection site pain	Injection site pain	General disorders and administration site conditions	0	57	60
247	Asian	13	1971	46	Female	50 ug MSP-1	20	20	Induration at injection site	No	(Definitely) related	Mild	Dose not changed	Recovered/ resolved	04/07/2017	.	Injection site induration	Injection site induration	General disorders and administration site conditions	0	57	64
248	Asian	13	1971	46	Female	50 ug MSP-1	21	21	Fatigue	No	Possible	Mild	Dose not changed	Recovered/ resolved	27/06/2017	.	Fatigue	Fatigue	General disorders and administration site conditions	0	57	57
249	Asian	13	1971	46	Female	50 ug MSP-1	29	29	sore throat	No	Unlikely	Mild	Dose not changed	Recovered/ resolved	28/10/2017	.	Sore throat	Oropharyngeal pain	Respiratory, thoracic and mediastinal disorders	114	171	180
250	Asian	13	1971	46	Female	50 ug MSP-1	22	22	itching at injection site	No	(Definitely) related	Mild	Dose not changed	Recovered/ resolved	25/10/2017	.	Injection site itching	Injection site pruritus	General disorders and administration site conditions	0	175	177
251	Asian	13	1971	46	Female	50 ug MSP-1	23	23	pain at injection site	No	(Definitely) related	Mild	Dose not changed	Recovered/ resolved	25/10/2017	.	Injection site pain	Injection site pain	General disorders and administration site conditions	1	176	177
252	Asian	13	1971	46	Female	50 ug MSP-1	24	24	warmth at injection site	No	(Definitely) related	Mild	Dose not changed	Recovered/ resolved	24/10/2017	.	Injection site warmth	Injection site warmth	General disorders and administration site conditions	1	176	176
253	Asian	13	1971	46	Female	50 ug MSP-1	25	25	fatigue	No	Probable	Mild	Dose not changed	Recovered/ resolved	25/10/2017	.	Fatigue	Fatigue	General disorders and administration site conditions	1	176	177
254	Asian	13	1971	46	Female	50 ug MSP-1	26	26	chills	No	Probable	Mild	Dose not changed	Recovered/ resolved	24/10/2017	.	Chills	Chills	General disorders and administration site conditions	1	176	176
255	Asian	13	1971	46	Female	50 ug MSP-1	27	27	headache	No	Probable	Mild	Dose not changed	Recovered/ resolved	24/10/2017	.	Headache	Headache	Nervous system disorders	1	176	176
256	Asian	13	1971	46	Female	50 ug MSP-1	28	28	joint ache	No	Probable	Mild	Dose not changed	Recovered/ resolved	24/10/2017	.	Joint ache	Arthralgia	Musculoskeletal and connective tissue disorders	1	176	176
257	Asian	13	1971	46	Female	50 ug MSP-1	30	30	common cold symptoms	No	Unlikely	Mild	Dose not changed	Recovered/ resolved	22/11/2017	.	Common cold	Nasopharyngitis	Infections and infestations	28	203	205
258	Asian	13	1971	46	Female	50 ug MSP-1	31	31	headache	No	Unlikely	Mild	Dose not changed	Recovered/ resolved	22/11/2017	.	Headache	Headache	Nervous system disorders	28	203	205
259	Asian	14	1979	38	Female	50 ug MSP-1	2	2	Hematoma left upper arm	No	Not related	Mild	Dose not changed	Recovered/ resolved	06/05/2017	.	Hematoma	Haematoma	Vascular disorders	0	1	4
260	Asian	14	1979	38	Female	50 ug MSP-1	3	3	Induration at injection site	No	(Definitely) related	Mild	Dose not changed	Recovered/ resolved	03/05/2017	.	Injection site induration	Injection site induration	General disorders and administration site conditions	0	1	1
261	Asian	14	1979	38	Female	50 ug MSP-1	8	8	Pain at injection site	No	(Definitely) related	Mild	Dose not changed	Recovered/ resolved	04/05/2017	.	Injection site pain	Injection site pain	General disorders and administration site conditions	0	1	2
262	Asian	14	1979	38	Female	50 ug MSP-1	6	6	sibilant rhonchi	No	Unlikely	Mild	Dose not changed	Recovered/ resolved	10/05/2017	.	Rhonchi	Rhonchi	Respiratory, thoracic and mediastinal disorders	1	2	8
263	Asian	14	1979	38	Female	50 ug MSP-1	7	7	Contact dermatitis at left antecubital fossa (due to adhesive bandage)	No	Not related	Mild	Dose not changed	Recovered/ resolved	31/05/2017	.	Contact dermatitis	Dermatitis contact	Skin and subcutaneous tissue disorders	1	2	29
264	Asian	14	1979	38	Female	50 ug MSP-1	10	10	Induration at injection site	No	(Definitely) related	Mild	Dose not changed	Recovered/ resolved	06/06/2017	.	Injection site induration	Injection site induration	General disorders and administration site conditions	0	29	35
265	Asian	14	1979	38	Female	50 ug MSP-1	11	11	pain at injection site	No	(Definitely) related	Mild	Dose not changed	Recovered/ resolved	02/06/2017	.	Injection site pain	Injection site pain	General disorders and administration site conditions	0	29	31
266	Asian	14	1979	38	Female	50 ug MSP-1	18	18	Blood bilirubin increased, intermittent	No	Unlikely	Moderate	Dose not changed	Recovered/ resolved	27/12/2017	.	Blood bilirubin increased	Blood bilirubin increased	Investigations	6	35	239
267	Asian	14	1979	38	Female	50 ug MSP-1	12	12	Rhinitis	No	Unlikely	Mild	Dose not changed	Recovered/ resolved	29/06/2017	.	Rhinitis	Rhinitis	Infections and infestations	21	50	58
268	Asian	14	1979	38	Female	50 ug MSP-1	13	13	Pain at injection site	No	(Definitely) related	Mild	Dose not changed	Recovered/ resolved	29/06/2017	.	Injection site pain	Injection site pain	General disorders and administration site conditions	0	57	58
269	Asian	14	1979	38	Female	50 ug MSP-1	14	14	Eosinophil count increased	No	Possible	Mild	Dose not changed	Recovered/ resolved	05/07/2017	.	Eosinophil count increased	Eosinophil count increased	Investigations	2	59	64
270	Asian	14	1979	38	Female	50 ug MSP-1	15	15	Fatigue	No	Possible	Mild	Dose not changed	Recovered/ resolved	07/07/2017	.	Fatigue	Fatigue	General disorders and administration site conditions	2	59	66
271	Asian	14	1979	38	Female	50 ug MSP-1	16.01	16	Papular erythema after wasp sting	No	Not related	Mild	Dose not changed	Recovered/ resolved	02/08/2017	1	Papular rash	Rash papular	Skin and subcutaneous tissue disorders	23	80	92
272	Asian	14	1979	38	Female	50 ug MSP-1	16.02	16	Papular erythema after wasp sting	No	Not related	Mild	Dose not changed	Recovered/ resolved	02/08/2017	2	Wasp sting	Arthropod sting	Injury, poisoning and procedural complications	23	80	92
273	Asian	14	1979	38	Female	50 ug MSP-1	19	19	Warmth at injection site	No	(Definitely) related	Mild	Dose not changed	Recovered/ resolved	15/11/2017	.	Injection site warmth	Injection site warmth	General disorders and administration site conditions	0	197	197
274	Asian	14	1979	38	Female	50 ug MSP-1	20	20	Induration at injection site	No	(Definitely) related	Mild	Dose not changed	Recovered/ resolved	21/11/2017	.	Injection site induration	Injection site induration	General disorders and administration site conditions	2	199	203
275	Asian	14	1979	38	Female	50 ug MSP-1	21	21	hematoma at injection site	No	(Definitely) related	Mild	Dose not changed	Recovered/ resolved	21/11/2017	.	Hematoma injection site	Injection site haematoma	General disorders and administration site conditions	2	199	203
276	Asian	14	1979	38	Female	50 ug MSP-1	22	22	Nausea	No	Unlikely	Mild	Not applicable	Recovered/ resolved	13/12/2017	.	Nausea	Nausea	Gastrointestinal disorders	28	225	225
277	Caucasian/White	15	1991	26	Male	50 ug MSP-1	1	1	Pain at injection site	No	(Definitely) related	Mild	Dose not changed	Recovered/ resolved	04/05/2017	.	Injection site pain	Injection site pain	General disorders and administration site conditions	0	1	2
278	Caucasian/White	15	1991	26	Male	50 ug MSP-1	2	2	Warmth at injection site	No	(Definitely) related	Mild	Dose not changed	Recovered/ resolved	05/05/2017	.	Injection site warmth	Injection site warmth	General disorders and administration site conditions	1	2	3
279	Caucasian/White	15	1991	26	Male	50 ug MSP-1	3	3	Induration at injection site	No	(Definitely) related	Mild	Dose not changed	Recovered/ resolved	17/05/2017	.	Injection site induration	Injection site induration	General disorders and administration site conditions	1	2	15
280	Caucasian/White	15	1991	26	Male	50 ug MSP-1	4	4	Hematoma at injection site	No	(Definitely) related	Mild	Dose not changed	Recovered/ resolved	17/05/2017	.	Hematoma injection site	Injection site haematoma	General disorders and administration site conditions	2	3	15
281	Caucasian/White	15	1991	26	Male	50 ug MSP-1	5	5	Anisocoria	No	Unlikely	Mild	Dose not changed	Recovered/ resolved	17/05/2017	.	Anisocoria	Pupils unequal	Eye disorders	7	8	15
282	Caucasian/White	15	1991	26	Male	50 ug MSP-1	6	6	Agitation	No	Unlikely	Moderate	Dose not changed	Recovered/ resolved	17/05/2017	.	Agitation	Agitation	Psychiatric disorders	7	8	15
283	Caucasian/White	15	1991	26	Male	50 ug MSP-1	7	7	tremor of the head and upper limbs	No	Unlikely	Mild	Dose not changed	Recovered/ resolved	17/05/2017	.	Tremor	Tremor	Nervous system disorders	7	8	15
284	Caucasian/White	15	1991	26	Male	50 ug MSP-1	9	9	Microhematurie	No	Possible	Mild	Dose not changed	Recovered/ resolved	30/05/2017	.	Microscopic hematuria	Haematuria	Renal and urinary disorders	14	15	28
285	Caucasian/White	15	1991	26	Male	50 ug MSP-1	13	13	Warmth at injection site	No	(Definitely) related	Mild	Dose not changed	Recovered/ resolved	02/06/2017	.	Injection site warmth	Injection site warmth	General disorders and administration site conditions	0	29	31
286	Caucasian/White	15	1991	26	Male	50 ug MSP-1	14	14	localised edema at injection site	No	(Definitely) related	Mild	Dose not changed	Recovered/ resolved	01/06/2017	.	Edema injection site	Injection site oedema	General disorders and administration site conditions	0	29	30
287	Caucasian/White	15	1991	26	Male	50 ug MSP-1	15	15	Pain at injection site	No	(Definitely) related	Mild	Dose not changed	Recovered/ resolved	02/06/2017	.	Injection site pain	Injection site pain	General disorders and administration site conditions	0	29	31
288	Caucasian/White	15	1991	26	Male	50 ug MSP-1	16	16	Induration at injection site	No	(Definitely) related	Mild	Dose not changed	Recovered/ resolved	02/06/2017	.	Injection site induration	Injection site induration	General disorders and administration site conditions	0	29	31
289	Caucasian/White	15	1991	26	Male	50 ug MSP-1	10	10	Protein in the urine intermittent	No	Possible	Mild	Dose not changed	Recovered/ resolved	30/11/2017	.	Protein urine	Protein urine	Investigations	2	31	212
290	Caucasian/White	15	1991	26	Male	50 ug MSP-1	12	12	Microhematurie	No	Possible	Mild	Dose not changed	Recovered/ resolved	08/06/2017	.	Microscopic hematuria	Haematuria	Renal and urinary disorders	2	31	37
291	Caucasian/White	15	1991	26	Male	50 ug MSP-1	8	8	upper respiratory infection	No	Probable	Mild	Dose not changed	Recovered/ resolved	14/06/2017	.	Upper respiratory infection	Upper respiratory tract infection	Infections and infestations	3	32	43
292	Caucasian/White	15	1991	26	Male	50 ug MSP-1	17	17	Headache	No	Possible	Mild	Dose not changed	Recovered/ resolved	05/06/2017	.	Headache	Headache	Nervous system disorders	4	33	34
293	Caucasian/White	15	1991	26	Male	50 ug MSP-1	18	18	Fever	No	Possible	Mild	Dose not changed	Recovered/ resolved	05/06/2017	.	Fever	Pyrexia	General disorders and administration site conditions	4	33	34
294	Caucasian/White	15	1991	26	Male	50 ug MSP-1	11	11	CRP increased	No	Possible	Mild	Dose not changed	Recovered/ resolved	14/06/2017	.	CRP increased	C-reactive protein increased	Investigations	7	36	43
295	Caucasian/White	15	1991	26	Male	50 ug MSP-1	19	20	Induration at injection site	No	(Definitely) related	Mild	Dose not changed	Recovered/ resolved	28/06/2017	.	Injection site induration	Injection site induration	General disorders and administration site conditions	0	57	57
296	Caucasian/White	15	1991	26	Male	50 ug MSP-1	20	21	hypotension	No	Possible	Moderate	Dose not changed	Recovered/ resolved	29/06/2017	.	Hypotension	Hypotension	Vascular disorders	0	57	58
297	Caucasian/White	15	1991	26	Male	50 ug MSP-1	21	22	warmth at injection site	No	(Definitely) related	Mild	Dose not changed	Recovered/ resolved	12/07/2017	.	Injection site warmth	Injection site warmth	General disorders and administration site conditions	1	58	71
298	Caucasian/White	15	1991	26	Male	50 ug MSP-1	22	23	common cold	No	Not related	Mild	Dose not changed	Recovered/ resolved	08/11/2017	.	Common cold	Nasopharyngitis	Infections and infestations	126	183	190
299	Caucasian/White	15	1991	26	Male	50 ug MSP-1	24	25	Pain at injection site	No	(Definitely) related	Mild	Dose not changed	Recovered/ resolved	16/11/2017	.	Injection site pain	Injection site pain	General disorders and administration site conditions	0	197	198
300	Caucasian/White	15	1991	26	Male	50 ug MSP-1	23	24	microhematuria	No	Possible	Mild	Dose not changed	Recovered/ resolved	30/11/2017	.	Microscopic hematuria	Haematuria	Renal and urinary disorders	7	204	212
301	Caucasian/White	15	1991	26	Male	50 ug MSP-1	25	26	Depression	No	Not related	Mild	Not applicable	Recovered/ resolved	01/02/2018	.	Depression	Depression	Psychiatric disorders	47	244	275
302	mother finish/father indian	16	1995	22	Male	50 ug MSP-1	3	3	Pain and resistance by deep palpation of the left lumbar region at the abdome	No	Not related	Mild	Dose not changed	Recovered/ resolved	05/05/2017	.	Painful nodule	Nodule	General disorders and administration site conditions	0	1	3

303	mother finish/father indian	16	1995	22	Male	50 ug MSP-1	4	4	Fatigue	No	Possible	Mild	Dose not changed	Recovered/ resolved	05/05/2017	.	Fatigue	Fatigue	General disorders and administration site conditions	0	1	3
304	mother finish/father indian	16	1995	22	Male	50 ug MSP-1	5	5	Induration at injection site	No	(Definitely) related	Mild	Dose not changed	Recovered/ resolved	05/05/2017	.	Injection site induration	Injection site induration	General disorders and administration site conditions	1	2	3
305	mother finish/father indian	16	1995	22	Male	50 ug MSP-1	6	6	Warmth at injection site	No	(Definitely) related	Mild	Dose not changed	Recovered/ resolved	05/05/2017	.	Injection site warmth	Injection site warmth	General disorders and administration site conditions	1	2	3
306	mother finish/father indian	16	1995	22	Male	50 ug MSP-1	7	7	pain at injection site	No	(Definitely) related	Mild	Dose not changed	Recovered/ resolved	05/05/2017	.	Injection site pain	Injection site pain	General disorders and administration site conditions	1	2	3
307	mother finish/father indian	16	1995	22	Male	50 ug MSP-1	8	8	Hematoma at right antecubital fossa	No	Not related	Mild	Dose not changed	Recovered/ resolved	12/05/2017	.	Hematoma	Haematoma	Vascular disorders	2	3	10
308	mother finish/father indian	16	1995	22	Male	50 ug MSP-1	9	9	Induration at injection site	No	(Definitely) related	Mild	Dose not changed	Recovered/ resolved	07/06/2017	.	Injection site induration	Injection site induration	General disorders and administration site conditions	1	30	36
309	mother finish/father indian	16	1995	22	Male	50 ug MSP-1	10	10	white blood cell count decreased	No	Possible	Mild	Dose not changed	Recovered/ resolved	14/06/2017	.	White blood cell count decreased	White blood cell count decreased	Investigations	7	36	43
310	mother finish/father indian	16	1995	22	Male	50 ug MSP-1	11	12	Lymph node enlargement (right axilla)	No	Possible	Mild	Dose not changed	Recovered/ resolved	12/07/2017	.	Lymph nodes enlarged	Lymphadenopathy	Blood and lymphatic system disorders	2	59	71
311	mother finish/father indian	16	1995	22	Male	50 ug MSP-1	12	13	Induration at injection site	No	(Definitely) related	Mild	Dose not changed	Recovered/ resolved	17/11/2017	.	Injection site induration	Injection site induration	General disorders and administration site conditions	1	198	199
312	mother finish/father indian	16	1995	22	Male	50 ug MSP-1	13	14	Fatigue	No	Possible	Mild	Not applicable	Recovered/ resolved	18/11/2017	.	Fatigue	Fatigue	General disorders and administration site conditions	2	199	200
313	mother finish/father indian	16	1995	22	Male	50 ug MSP-1	14	15	Headache	No	Possible	Mild	Not applicable	Recovered/ resolved	18/11/2017	.	Headache	Headache	Nervous system disorders	2	199	200
314	mother finish/father indian	16	1995	22	Male	50 ug MSP-1	15	16	Flu like symptoms	No	Unlikely	Mild	Dose not changed	Recovered/ resolved	26/11/2017	.	Flu like symptoms	Influenza like illness	General disorders and administration site conditions	8	205	208
315	Caucasian/White	17	1995	22	Female	Adjuvant	1	1	Warmth at injection site	No	(Definitely) related	Mild	Dose not changed	Recovered/ resolved	07/09/2017	.	Injection site warmth	Injection site warmth	General disorders and administration site conditions	0	1	2
316	Caucasian/White	17	1995	22	Female	Adjuvant	3	3	Erythema at injection site	No	(Definitely) related	Mild	Dose not changed	Recovered/ resolved	07/09/2017	.	Injection site erythema	Injection site erythema	General disorders and administration site conditions	0	1	2
317	Caucasian/White	17	1995	22	Female	Adjuvant	4	4	headache	No	Possible	Mild	Dose not changed	Recovered/ resolved	07/09/2017	.	Headache	Headache	Nervous system disorders	0	1	2
318	Caucasian/White	17	1995	22	Female	Adjuvant	2	2	increase of CRP	No	Possible	Mild	Dose not changed	Recovered/ resolved	20/09/2017	.	CRP increased	C-reactive protein increased	Investigations	1	2	15
319	Caucasian/White	17	1995	22	Female	Adjuvant	5	5	Common Cold	No	Unlikely	Mild	Dose not changed	Recovered/ resolved	16/10/2017	.	Common cold	Nasopharyngitis	Infections and infestations	20	21	41
320	Caucasian/White	17	1995	22	Female	Adjuvant	8	8	Headache	No	Unlikely	Mild	Dose not changed	Recovered/ resolved	28/09/2017	.	Headache	Headache	Nervous system disorders	22	23	23
321	Caucasian/White	17	1995	22	Female	Adjuvant	7	7	Fever	No	Not related	Moderate	Dose not changed	Recovered/ resolved	29/09/2017	.	Fever	Pyrexia	General disorders and administration site conditions	23	24	24
322	Caucasian/White	17	1995	22	Female	Adjuvant	10	10	increased frequency of acne	No	Possible	Mild	Dose not changed	Recovered/ resolved	08/12/2017	.	Acne aggravated	Acne	Skin and subcutaneous tissue disorders	24	25	94
323	Caucasian/White	17	1995	22	Female	Adjuvant	9	9	CRP increased	No	Unlikely	Mild	Dose not changed	Recovered/ resolved	09/10/2017	.	CRP increased	C-reactive protein increased	Investigations	28	29	34
324	Caucasian/White	17	1995	22	Female	Adjuvant	6	6	Hematoma at injection site	No	(Definitely) related	Mild	Dose not changed	Recovered/ resolved	16/10/2017	.	Hematoma injection site	Injection site haematoma	General disorders and administration site conditions	2	36	41
325	Caucasian/White	17	1995	22	Female	Adjuvant	11	11	herpes simplex left lower lip	No	Possible	Mild	Not applicable	Recovered/ resolved	21/11/2017	.	Herpes simplex	Herpes simplex	Infections and infestations	5	67	77
326	Caucasian/White	17	1995	22	Female	Adjuvant	12	12	Herpes simplex upper lip	No	Possible	Mild	Not applicable	Recovered/ resolved	01/12/2017	.	Herpes simplex	Herpes simplex	Infections and infestations	15	77	87
327	Caucasian/White	18	1994	23	Female	Placebo	1	1	Headache	No	Possible	Mild	Dose not changed	Recovered/ resolved	08/09/2017	.	Headache	Headache	Nervous system disorders	0	1	3
328	Caucasian/White	18	1994	23	Female	Placebo	2	2	Fatigue	No	Possible	Mild	Dose not changed	Recovered/ resolved	08/09/2017	.	Fatigue	Fatigue	General disorders and administration site conditions	0	1	3
329	Caucasian/White	18	1994	23	Female	Placebo	4	4	Pain at injection site	No	(Definitely) related	Mild	Dose not changed	Recovered/ resolved	06/09/2017	.	Injection site pain	Injection site pain	General disorders and administration site conditions	0	1	1
330	Caucasian/White	18	1994	23	Female	Placebo	5	5	warmth at injection site	No	(Definitely) related	Mild	Dose not changed	Recovered/ resolved	06/09/2017	.	Injection site warmth	Injection site warmth	General disorders and administration site conditions	0	1	1
331	Caucasian/White	18	1994	23	Female	Placebo	3	3	Induration at injection site	No	(Definitely) related	Mild	Dose not changed	Recovered/ resolved	13/09/2017	.	Injection site induration	Injection site induration	General disorders and administration site conditions	2	3	8
332	Caucasian/White	18	1994	23	Female	Placebo	7	7	intermittent headache	No	Unlikely	Mild	Dose not changed	Recovered/ resolved	02/10/2017	.	Intermittent headache	Headache	Nervous system disorders	3	4	27
333	Caucasian/White	18	1994	23	Female	Placebo	6	6	intermittent backpain	No	Unlikely	Mild	Dose not changed	Recovered/ resolved	19/09/2017	.	Back pain	Back pain	Musculoskeletal and connective tissue disorders	9	10	14
334	Caucasian/White	18	1994	23	Female	Placebo	10	10	Irregular menstruation	No	Possible	Mild	Dose not changed	Recovered/ resolved	10/11/2017	.	Irregular menstruation	Menstruation irregular	Reproductive system and breast disorders	10	11	66
335	Caucasian/White	18	1994	23	Female	Placebo	12	12	Intermittent headache	No	Unlikely	Mild	Dose not changed	Recovered/ resolved	28/10/2017	.	Intermittent headache	Headache	Nervous system disorders	7	36	53
336	Caucasian/White	18	1994	23	Female	Placebo	8	8	Diarrhea	No	Possible	Mild	Dose not changed	Recovered/ resolved	04/11/2017	.	Diarrhea	Diarrhoea	Gastrointestinal disorders	2	60	60
337	Caucasian/White	18	1994	23	Female	Placebo	9.01	9	Nervous system disorders-headache, dizziness, paresthesia, anesthesia	No	Possible	Mild	Dose not changed	Recovered/ resolved	06/11/2017	1	Headache	Headache	Nervous system disorders	2	60	62
338	Caucasian/White	18	1994	23	Female	Placebo	9.02	9	Nervous system disorders-headache, dizziness, paresthesia, anesthesia	No	Possible	Mild	Dose not changed	Recovered/ resolved	06/11/2017	2	Dizziness	Dizziness	Nervous system disorders	2	60	62
339	Caucasian/White	18	1994	23	Female	Placebo	9.03	9	Nervous system disorders-headache, dizziness, paresthesia, anesthesia	No	Possible	Mild	Dose not changed	Recovered/ resolved	06/11/2017	3	Paresthesia	Paraesthesia	Nervous system disorders	2	60	62
340	Caucasian/White	18	1994	23	Female	Placebo	9.04	9	Nervous system disorders-headache, dizziness, paresthesia, anesthesia	No	Possible	Mild	Dose not changed	Recovered/ resolved	06/11/2017	4	Anesthesia	Anaesthesia	Nervous system disorders	2	60	62
341	Caucasian/White	18	1994	23	Female	Placebo	13	13	Headache	No	Possible	Mild	Dose not changed	Recovered/ resolved	10/11/2017	.	Headache	Headache	Nervous system disorders	7	65	66
342	Caucasian/White	18	1994	23	Female	Placebo	11	11	Common cold symptoms	No	Possible	Mild	Dose not changed	Recovered/ resolved	23/11/2017	.	Common cold	Nasopharyngitis	Infections and infestations	14	72	79
343	Caucasian/White	18	1994	23	Female	Placebo	14	14	Headache	No	Unlikely	Mild	Dose not changed	Recovered/ resolved	23/11/2017	.	Headache	Headache	Nervous system disorders	15	73	79
344	Black	19	1992	25	Male	150 ug MSP-1	1	1	Pain at injection site	No	(Definitely) related	Mild	Dose not changed	Recovered/ resolved	08/09/2017	.	Injection site pain	Injection site pain	General disorders and administration site conditions	0	1	3
345	Black	19	1992	25	Male	150 ug MSP-1	2	2	Fatigue	No	Possible	Mild	Dose not changed	Recovered/ resolved	07/09/2017	.	Fatigue	Fatigue	General disorders and administration site conditions	0	1	2
346	Black	19	1992	25	Male	150 ug MSP-1	3	3	Myalgia	No	(Definitely) related	Mild	Dose not changed	Recovered/ resolved	07/09/2017	.	Myalgia	Myalgia	Musculoskeletal and connective tissue disorders	0	1	2
347	Black	19	1992	25	Male	150 ug MSP-1	4	4	pain at site of i.v. catheter	No	Not related	Mild	Dose not changed	Recovered/ resolved	20/09/2017	.	Catheter site pain	Catheter site pain	General disorders and administration site conditions	2	3	15
348	Black	19	1992	25	Male	150 ug MSP-1	5	5	flu like symptoms	No	Unlikely	Mild	Dose not changed	Recovered/ resolved	29/09/2017	.	Flu like symptoms	Influenza like illness	General disorders and administration site conditions	21	22	24
349	Black	19	1992	25	Male	150 ug MSP-1	8	8	chills	No	(Definitely) related	Mild	Dose not changed	Recovered/ resolved	29/09/2017	.	Chills	Chills	General disorders and administration site conditions	21	22	24
350	Black	19	1992	25	Male	150 ug MSP-1	9	9	Itching at injection site	No	(Definitely) related	Mild	Dose not changed	Recovered/ resolved	06/10/2017	.	Injection site itching	Injection site pruritus	General disorders and administration site conditions	1	30	31
351	Black	19	1992	25	Male	150 ug MSP-1	6	6	CRP elevation	No	(Definitely) related	Mild	Dose not changed	Recovered/ resolved	12/10/2017	.	CRP increased	C-reactive protein increased	Investigations	2	31	37
352	Black	19	1992	25	Male	150 ug MSP-1	7	7	Fatigue	No	Unlikely	Mild	Dose not changed	Recovered/ resolved	13/10/2017	.	Fatigue	Fatigue	General disorders and administration site conditions	8	37	38
353	Black	19	1992	25	Male	150 ug MSP-1	10	10	Headache	No	Possible	Mild	Dose not changed	Recovered/ resolved	02/11/2017	.	Headache	Headache	Nervous system disorders	0	58	58
354	Black	19	1992	25	Male	150 ug MSP-1	11	11	Dizziness	No	Possible	Mild	Dose not changed	Recovered/ resolved	02/11/2017	.	Dizziness	Dizziness	Nervous system disorders	0	58	58
355	Black	19	1992	25	Male	150 ug MSP-1	14	14	Pain at injection site	No	(Definitely) related	Mild	Dose not changed	Recovered/ resolved	02/11/2017	.	Injection site pain	Injection site pain	General disorders and administration site conditions	0	58	58
356	Black	19	1992	25	Male	150 ug MSP-1	12	12	Warmth at injection site	No	(Definitely) related	Mild	Dose not changed	Recovered/ resolved	04/11/2017	.	Injection site warmth	Injection site warmth	General disorders and administration site conditions	1	59	60
357	Black	19	1992	25	Male	150 ug MSP-1	13	13	CRP elevation	No	Possible	Mild	Dose not changed	Recovered/ resolved	30/11/2017	.	CRP increased	C-reactive protein increased	Investigations	2	60	86
358	Caucasian/White	20	1983	34	Male	Adjuvant	1	1	Pain at injection site	No	(Definitely) related	Mild	Dose not changed	Recovered/ resolved	12/09/2017	.	Injection site pain	Injection site pain	General disorders and administration site conditions	1	2	2
359	Caucasian/White	20	1983	34	Male	Adjuvant	2	2	Induration at injection site	No	(Definitely) related	Mild	Dose not changed	Recovered/ resolved	12/09/2017	.	Injection site induration	Injection site induration	General disorders and administration site conditions	1	2	2
360	Caucasian/White	20	1983	34	Male	Adjuvant	3	3	Hematoma at injection site	No	(Definitely) related	Mild	Dose not changed	Recovered/ resolved	18/09/2017	.	Hematoma injection site	Injection site haematoma	General disorders and administration site conditions	1	2	8
361	Caucasian/White	20	1983	34	Male	Adjuvant	4	4	Blood bilirubin increased, intermittent	No	Possible	Moderate	Dose not changed	Recovered/ resolved	01/12/2017	.	Blood bilirubin increased	Blood bilirubin increased	Investigations	1	2	82
362	Caucasian/White	20	1983	34	Male	Adjuvant	5	5	Pain at injection site	No	(Definitely) related	Mild	Dose not changed	Recovered/ resolved	11/10/2017	.	Injection site pain	Injection site pain	General disorders and administration site conditions	1	30	31
363	Caucasian/White	20	1983	34	Male	Adjuvant	6	6	Headache	No	Unlikely	Mild	Dose not changed	Recovered/ resolved	14/10/2017	.	Headache	Headache	Nervous system disorders	5	34	34
364	Caucasian/White	20	1983	34	Male	Adjuvant	7	7	hematoma right upper arm	No	Unlikely	Mild	Dose not changed	Recovered/ resolved	01/12/2017	.	Hematoma	Haematoma	Vascular disorders	9	66	82
365	Caucasian/White	20	1983	34	Male	Adjuvant	8	8	CRP increased	No	Unlikely	Mild	Dose not changed	Recovered/ resolved	01/12/2017	.	CRP increased	C-reactive protein increased	Investigations	11	68	82
366	Caucasian/White	21	1978	39	Female	25 ug MSP-1	1	1	Pain at injection site	No	(Definitely) related	Mild	Dose not changed	Recovered/ resolved	12/09/2017	.	Injection site pain	Injection site pain	General disorders and administration site conditions	0	1	2
367	Caucasian/White	21	1978	39	Female	25 ug MSP-1	2	2	Warmth at injection site	No	(Definitely) related	Mild	Dose not changed	Recovered/ resolved	12/09/2017	.	Injection site warmth	Injection site warmth	General disorders and administration site conditions	1	2	2
368	Caucasian/White	21	1978	39	Female	25 ug MSP-1	3	3	neck pain	No	Unlikely	Mild	Dose not changed	Recovered/ resolved	18/09/2017	.	Neck pain	Neck pain	Musculoskeletal and connective tissue disorders	1	2	8
369	Caucasian/White	21	1978	39	Female	25 ug MSP-1	6	6	Warmth at injection site	No	(Definitely) related	Mild	Dose not changed	Recovered/ resolved	18/09/2017	.	Injection site warmth	Injection site warmth	General disorders and administration site conditions	1	2	8
370	Caucasian/White	21	1978	39	Female	25 ug MSP-1	7	7	pain at injection site	No	(Definitely) related	Mild	Dose not changed	Recovered/ resolved	18/09/2017	.	Injection site pain	Injection site pain	General disorders and administration site conditions	1	2	8
371	Caucasian/White	21	1978	39	Female	25 ug MSP-1	8	8	neck pain	No	Possible	Mild	Dose not changed	Recovered/ resolved	25/09/2017	.	Neck pain	Neck pain	Musculoskeletal and connective tissue disorders	1	2	15
372	Caucasian/White	21	1978	39	Female	25 ug MSP-1	4	4	headache	No	(Definitely) related	Mild	Dose not changed	Recovered/ resolved	19/09/2017	.	Headache	Headache	Nervous system disorders	7	8	9
373	Caucasian/White	21	1978	39	Female	25 ug MSP-1	5	5	Hematoma leg	No	Not related	Mild	Dose not changed	Recovered/ resolved	16/10/2017	.	Hematoma	Haematoma	Vascular disorders	14	15	36
374	Caucasian/White	21	1978	39	Female	25 ug MSP-1	10	11	upper respiratory infection	No	Unlikely	Mild	Dose not changed	Recovered/ resolved	07/10/2017	.	Upper respiratory infection	Upper respiratory tract infection	Infections and infestations	23	24	27
375	Caucasian/White	21	1978	39	Female	25 ug MSP-1	11	12	vertigo	No	Unlikely	Mild	Dose not changed	Recovered/ resolved	10/10/2017	.	Vertigo	Vertigo	Ear and labyrinth disorders	0	29	30
376	Caucasian/White	21	1978	39	Female	25 ug MSP-1	12	13	nausea	No	Unlikely	Mild	Dose not changed	Recovered/ resolved	10/10/2017	.	Nausea	Nausea	Gastrointestinal disorders	0	29	30
377	Caucasian/White	21	1978	39	Female	25 ug MSP-1	13	14	sweating	No	Unlikely	Mild	Dose not changed	Recovered/ resolved	10/10/2017	.	Sweating	Hyperhidrosis	Skin and subcutaneous tissue disorders	0	29	30

378	Caucasian/White	21	1978	39	Female	25 ug MSP-1	9	10	headache	No	Not related	Mild	Dose not changed	Recovered/ resolved	19/10/2017	.	Headache	Headache	Nervous system disorders	10	39	39
379	Caucasian/White	21	1978	39	Female	25 ug MSP-1	14	15	common cold	No	Not related	Mild	Dose not changed	Recovered/ resolved	10/11/2017	.	Common cold	Nasopharyngitis	Infections and infestations	27	56	61
380	Caucasian/White	21	1978	39	Female	25 ug MSP-1	15	16	headache	No	Possible	Moderate	Dose not changed	Recovered/ resolved	21/11/2017	.	Headache	Headache	Nervous system disorders	4	70	72
381	Caucasian/White	21	1978	39	Female	25 ug MSP-1	16	17	muscle pain	No	Possible	Mild	Dose not changed	Recovered/ resolved	21/11/2017	.	Muscle pain	Myalgia	Musculoskeletal and connective tissue disorders	4	70	72
382	Caucasian/White	21	1978	39	Female	25 ug MSP-1	17	18	joint ache	No	Possible	Mild	Dose not changed	Recovered/ resolved	21/11/2017	.	Joint ache	Arthralgia	Musculoskeletal and connective tissue disorders	4	70	72
383	Caucasian/White	21	1978	39	Female	25 ug MSP-1	18	19	fever	No	Possible	Moderate	Dose not changed	Recovered/ resolved	21/11/2017	.	Fever	Pyrexia	General disorders and administration site conditions	4	70	72
384	Caucasian/White	21	1978	39	Female	25 ug MSP-1	19	20	Fatigue	No	Possible	Mild	Dose not changed	Recovered/ resolved	22/11/2017	.	Fatigue	Fatigue	General disorders and administration site conditions	4	70	73
385	Caucasian/White	21	1978	39	Female	25 ug MSP-1	20	21	Tonsillitis	No	Possible	Moderate	Dose not changed	Recovered/ resolved	12/12/2017	.	Tonsillitis	Tonsillitis	Infections and infestations	4	70	93
386	Caucasian/White	21	1978	39	Female	25 ug MSP-1	21	22	swollen cervical lymphatic nodules	No	Possible	Mild	Dose not changed	Recovered/ resolved	12/12/2017	.	Swollen lymph nodes	Lymphadenopathy	Blood and lymphatic system disorders	4	70	93
387	Caucasian/White	21	1978	39	Female	25 ug MSP-1	30	31	Chills	No	Possible	Mild	Not applicable	Recovered/ resolved	21/11/2017	.	Chills	Chills	General disorders and administration site conditions	5	71	72
388	Caucasian/White	21	1978	39	Female	25 ug MSP-1	31	32	Sweating	No	Possible	Mild	Not applicable	Recovered/ resolved	22/11/2017	.	Sweating	Hyperhidrosis	Skin and subcutaneous tissue disorders	6	72	73
389	Caucasian/White	21	1978	39	Female	25 ug MSP-1	22	23	Cough	No	Unlikely	Mild	Dose not changed	Recovered/ resolved	02/12/2017	.	Cough	Cough	Respiratory, thoracic and mediastinal disorders	14	80	83
390	Caucasian/White	21	1978	39	Female	25 ug MSP-1	23	24	AST increased	No	Possible	Mild	Dose not changed	Recovered/ resolved	12/12/2017	.	AST increased	Aspartate aminotransferase increased	Investigations	14	80	93
391	Caucasian/White	21	1978	39	Female	25 ug MSP-1	24	25	ALT increased	No	Possible	Mild	Dose not changed	Recovered/ resolved	12/12/2017	.	ALT increased	Alanine aminotransferase increased	Investigations	14	80	93
392	Caucasian/White	21	1978	39	Female	25 ug MSP-1	25	26	Bronchial infection	No	Not related	Moderate	Not applicable	Recovered/ resolved	07/02/2018	.	Bronchial infection	Bronchitis	Infections and infestations	77	143	150
393	Caucasian/White	21	1978	39	Female	25 ug MSP-1	26	27	Pain at injection site	No	(Definitely) related	Mild	Not applicable	Recovered/ resolved	09/03/2018	.	Injection site pain	Injection site pain	General disorders and administration site conditions	0	178	180
394	Caucasian/White	21	1978	39	Female	25 ug MSP-1	27	28	Induration at injection site	No	(Definitely) related	Mild	Not applicable	Recovered/ resolved	09/03/2018	.	Injection site induration	Injection site induration	General disorders and administration site conditions	1	179	180
395	Caucasian/White	21	1978	39	Female	25 ug MSP-1	28	29	CRP increased	No	Possible	Mild	Not applicable	Recovered/ resolved	14/03/2018	.	CRP increased	C-reactive protein increased	Investigations	1	179	185
396	Caucasian/White	21	1978	39	Female	25 ug MSP-1	29	30	Haematoma at injection site	No	(Definitely) related	Mild	Not applicable	Recovered/ resolved	06/04/2018	.	Haematoma injection site	Injection site haematoma	General disorders and administration site conditions	2	180	208
397	Chinese/Swiss	22	1978	39	Female	150 ug MSP-1	2	2	Pain at injection site	No	(Definitely) related	Mild	Dose not changed	Recovered/ resolved	18/09/2017	.	Injection site pain	Injection site pain	General disorders and administration site conditions	0	1	8
398	Chinese/Swiss	22	1978	39	Female	150 ug MSP-1	1	1	Lymphocyte count decreased	No	Possible	Mild	Dose not changed	Recovered/ resolved	18/09/2017	.	Lymphocyte count decreased	Lymphocyte count decreased	Investigations	1	2	8
399	Chinese/Swiss	22	1978	39	Female	150 ug MSP-1	3	3	Erythema at injection site	No	(Definitely) related	Mild	Dose not changed	Recovered/ resolved	18/09/2017	.	Injection site erythema	Injection site erythema	General disorders and administration site conditions	1	2	8
400	Chinese/Swiss	22	1978	39	Female	150 ug MSP-1	8	8	itching at injection site	No	Probable	Mild	Dose not changed	Recovered/ resolved	13/09/2017	.	Injection site itching	Injection site pruritus	General disorders and administration site conditions	2	3	3
401	Chinese/Swiss	22	1978	39	Female	150 ug MSP-1	9.01	9	Erythematous plaque at right upper leg (insect bite9	No	Not related	Mild	Dose not changed	Recovered/ resolved	23/10/2017	1	Erythematous rash	Rash erythematous	Skin and subcutaneous tissue disorders	21	22	43
402	Chinese/Swiss	22	1978	39	Female	150 ug MSP-1	9.02	9	Erythematous plaque at right upper leg (insect bite9	No	Not related	Mild	Dose not changed	Recovered/ resolved	23/10/2017	2	Insect bite NOS	Arthropod bite	Injury, poisoning and procedural complications	21	22	43
403	Chinese/Swiss	22	1978	39	Female	150 ug MSP-1	4	4	Pain at injection site	No	(Definitely) related	Mild	Dose not changed	Recovered/ resolved	11/10/2017	.	Injection site pain	Injection site pain	General disorders and administration site conditions	1	30	31
404	Chinese/Swiss	22	1978	39	Female	150 ug MSP-1	5	5	Warmth at injection site	No	(Definitely) related	Mild	Dose not changed	Recovered/ resolved	11/10/2017	.	Injection site warmth	Injection site warmth	General disorders and administration site conditions	1	30	31
405	Chinese/Swiss	22	1978	39	Female	150 ug MSP-1	6	6	cold symptoms	No	Unlikely	Mild	Dose not changed	Recovered/ resolved	27/10/2017	.	Cold symptoms	Nasopharyngitis	Infections and infestations	11	40	47
406	Chinese/Swiss	22	1978	39	Female	150 ug MSP-1	7	7	lymphocyte count decreased	No	Possible	Moderate	Dose not changed	Recovered/ resolved	06/11/2017	.	Lymphocyte count decreased	Lymphocyte count decreased	Investigations	14	43	57
407	Chinese/Swiss	22	1978	39	Female	150 ug MSP-1	10	10	Pain at injection site	No	(Definitely) related	Mild	Not applicable	Recovered/ resolved	23/02/2018	.	Injection site pain	Injection site pain	General disorders and administration site conditions	0	164	166
408	Chinese/Swiss	22	1978	39	Female	150 ug MSP-1	11	11	Erythema at injection site	No	(Definitely) related	Mild	Not applicable	Recovered/ resolved	23/02/2018	.	Injection site erythema	Injection site erythema	General disorders and administration site conditions	1	165	166
409	Chinese/Swiss	22	1978	39	Female	150 ug MSP-1	12	12	Edma at injection site	No	(Definitely) related	Mild	Not applicable	Recovered/ resolved	23/02/2018	.	Edema injection site	Injection site oedema	General disorders and administration site conditions	1	165	166
410	Chinese/Swiss	22	1978	39	Female	150 ug MSP-1	13	13	warmth at injection site	No	(Definitely) related	Mild	Not applicable	Recovered/ resolved	23/02/2018	.	Injection site warmth	Injection site warmth	General disorders and administration site conditions	1	165	166
411	Chinese/Swiss	22	1978	39	Female	150 ug MSP-1	14	14	pruritus at injection site	No	(Definitely) related	Mild	Not applicable	Recovered/ resolved	23/02/2018	.	Injection site pruritus	Injection site pruritus	General disorders and administration site conditions	1	165	166
412	Chinese/Swiss	22	1978	39	Female	150 ug MSP-1	15	15	Lymphopenia	No	Possible	Mild	Not applicable	Recovered/ resolved	28/02/2018	.	Lymphopenia	Lymphopenia	Blood and lymphatic system disorders	2	166	171
413	Chinese/Swiss	22	1978	39	Female	150 ug MSP-1	16	16	Common cold symptoms	No	Possible	Mild	Not applicable	Recovered/ resolved	25/02/2018	.	Common cold	Nasopharyngitis	Infections and infestations	4	168	168
414	Chinese/Swiss	22	1978	39	Female	150 ug MSP-1	17	17	Rash maculo-papular	No	Possible	Mild	Not applicable	Recovered/ resolved	05/03/2018	.	Rash maculo-papular	Rash maculo-papular	Skin and subcutaneous tissue disorders	11	175	176
415	Chinese/Swiss	22	1978	39	Female	150 ug MSP-1	18	18	Monocytopenia	No	Possible	Mild	Not applicable	Recovered/ resolved	19/03/2018	.	Monocytopenia	Monocytopenia	Blood and lymphatic system disorders	14	178	190
416	Caucasian/White	23	1971	46	Male	Placebo	1	1	Itching at injection site	No	(Definitely) related	Mild	Dose not changed	Recovered/ resolved	21/09/2017	.	Injection site itching	Injection site pruritus	General disorders and administration site conditions	1	2	3
417	Caucasian/White	23	1971	46	Male	Placebo	2	2	Vertigo	No	Possible	Mild	Dose not changed	Recovered/ resolved	21/09/2017	.	Vertigo	Vertigo	Ear and labyrinth disorders	1	2	3
418	Caucasian/White	23	1971	46	Male	Placebo	3	3	Proteinuria	No	Unlikely	Mild	Dose not changed	Recovered/ resolved	21/09/2017	.	Proteinuria	Proteinuria	Renal and urinary disorders	1	2	3
419	Caucasian/White	23	1971	46	Male	Placebo	4	4	Headache	No	Unlikely	Mild	Dose not changed	Recovered/ resolved	25/09/2017	.	Headache	Headache	Nervous system disorders	4	5	7
420	Caucasian/White	23	1971	46	Male	Placebo	7	7	Fatigue	No	Possible	Mild	Dose not changed	Recovered/ resolved	26/09/2017	.	Fatigue	Fatigue	General disorders and administration site conditions	7	8	8
421	Caucasian/White	23	1971	46	Male	Placebo	5	5	headache	No	Possible	Mild	Dose not changed	Recovered/ resolved	28/09/2017	.	Headache	Headache	Nervous system disorders	8	9	10
422	Caucasian/White	23	1971	46	Male	Placebo	6	6	erythematous macula with central crusta	No	Not related	Mild	Dose not changed	Recovered/ resolved	17/10/2017	.	Erythematous rash	Rash erythematous	Skin and subcutaneous tissue disorders	10	11	29
423	Caucasian/White	23	1971	46	Male	Placebo	8	8	Headache	No	Possible	Mild	Dose not changed	Recovered/ resolved	18/10/2017	.	Headache	Headache	Nervous system disorders	1	30	30
424	Caucasian/White	23	1971	46	Male	Placebo	9	9	Itching at injection site	No	(Definitely) related	Mild	Dose not changed	Recovered/ resolved	18/10/2017	.	Injection site itching	Injection site pruritus	General disorders and administration site conditions	1	30	30
425	Caucasian/White	23	1971	46	Male	Placebo	10	10	Headache, intermittent	No	Possible	Mild	Dose not changed	Recovered/ resolved	28/10/2017	.	Headache	Headache	Nervous system disorders	2	31	40
426	Caucasian/White	23	1971	46	Male	Placebo	11	11	Itching at injection site	No	(Definitely) related	Mild	Dose not changed	Recovered/ resolved	18/11/2017	.	Injection site itching	Injection site pruritus	General disorders and administration site conditions	1	59	61
427	Caucasian/White	24	1993	24	Female	25 ug MSP-1	1	1	warmth at injection site	No	(Definitely) related	Mild	Dose not changed	Recovered/ resolved	26/09/2017	.	Injection site warmth	Injection site warmth	General disorders and administration site conditions	0	1	1
428	Caucasian/White	24	1993	24	Female	25 ug MSP-1	2	2	pain at injection site	No	(Definitely) related	Mild	Dose not changed	Recovered/ resolved	28/09/2017	.	Injection site pain	Injection site pain	General disorders and administration site conditions	1	2	3
429	Caucasian/White	24	1993	24	Female	25 ug MSP-1	3	3	Hematoma at site of iv catheter	No	Not related	Mild	Dose not changed	Recovered/ resolved	10/10/2017	.	Catheter site hematoma	Catheter site haematoma	General disorders and administration site conditions	2	3	15
430	Caucasian/White	24	1993	24	Female	25 ug MSP-1	4	4	Common Cold	No	Unlikely	Mild	Dose not changed	Recovered/ resolved	18/10/2017	.	Common cold	Nasopharyngitis	Infections and infestations	18	19	23
431	Caucasian/White	24	1993	24	Female	25 ug MSP-1	15	15	Amnesia after alcohol intake	No	Not related	Mild	Dose not changed	Recovered/ resolved	20/10/2017	.	Acute alcoholic intoxication	Alcohol poisoning	Injury, poisoning and procedural complications	24	25	25
432	Caucasian/White	24	1993	24	Female	25 ug MSP-1	5	5	Fatigue	No	Possible	Moderate	Dose not changed	Recovered/ resolved	27/10/2017	.	Fatigue	Fatigue	General disorders and administration site conditions	1	30	32
433	Caucasian/White	24	1993	24	Female	25 ug MSP-1	6	6	Headache	No	Possible	Moderate	Dose not changed	Recovered/ resolved	27/10/2017	.	Headache	Headache	Nervous system disorders	1	30	32
434	Caucasian/White	24	1993	24	Female	25 ug MSP-1	7	7	Induration at injection site	No	(Definitely) related	Mild	Dose not changed	Recovered/ resolved	30/10/2017	.	Injection site induration	Injection site induration	General disorders and administration site conditions	2	31	35
435	Caucasian/White	24	1993	24	Female	25 ug MSP-1	8	8	Warmth at injection site	No	(Definitely) related	Mild	Dose not changed	Recovered/ resolved	30/10/2017	.	Injection site warmth	Injection site warmth	General disorders and administration site conditions	2	31	35
436	Caucasian/White	24	1993	24	Female	25 ug MSP-1	16	16	Amnesia after alcohol intake	No	Not related	Mild	Dose not changed	Recovered/ resolved	31/10/2017	.	Acute alcoholic intoxication	Alcohol poisoning	Injury, poisoning and procedural complications	7	36	36
437	Caucasian/White	24	1993	24	Female	25 ug MSP-1	11	11	Pain at injection site	No	(Definitely) related	Mild	Not applicable	Recovered/ resolved	21/11/2017	.	Injection site pain	Injection site pain	General disorders and administration site conditions	0	57	57
438	Caucasian/White	24	1993	24	Female	25 ug MSP-1	12	12	chills	No	Possible	Mild	Not applicable	Recovered/ resolved	21/11/2017	.	Chills	Chills	General disorders and administration site conditions	0	57	57
439	Caucasian/White	24	1993	24	Female	25 ug MSP-1	13	13	Nausea	No	Possible	Mild	Dose not changed	Recovered/ resolved	21/11/2017	.	Nausea	Nausea	Gastrointestinal disorders	0	57	57
440	Caucasian/White	24	1993	24	Female	25 ug MSP-1	14	14	Headache	No	Possible	Mild	Not applicable	Recovered/ resolved	24/11/2017	.	Headache	Headache	Nervous system disorders	2	59	60
441	Caucasian/White	24	1993	24	Female	25 ug MSP-1	9	9	Headache	No	Possible	Mild	Not applicable	Recovered/ resolved	26/11/2017	.	Headache	Headache	Nervous system disorders	4	61	62
442	Caucasian/White	24	1993	24	Female	25 ug MSP-1	10	10	White blood cell count decreased	No	Possible	Mild	Not applicable	Recovered/ resolved	19/12/2017	.	White blood cell count decreased	White blood cell count decreased	Investigations	14	71	85
443	Caucasian/White	24	1993	24	Female	25 ug MSP-1	17	17	Amnesia after alcohol intake	No	Not related	Mild	Not applicable	Recovered/ resolved	14/01/2018	.	Acute alcoholic intoxication	Alcohol poisoning	Injury, poisoning and procedural complications	53	110	111
444	Caucasian/White	25	1964	53	Female	25 ug MSP-1	8	8	necrotic pulp of a tooth (upper dental arch left)	No	Not related	Mild	Not applicable	Recovered/ resolved	01/02/2018	.	Necrosis of the pulp	Dental necrosis	Gastrointestinal disorders	.	.	129
445	Caucasian/White	25	1964	53	Female	25 ug MSP-1	1	1	hematome at injection site	No	(Definitely) related	Mild	Dose not changed	Recovered/ resolved	05/10/2017	.	Hematoma injection site	Injection site haematoma	General disorders and administration site conditions	1	2	10
446	Caucasian/White	25	1964	53	Female	25 ug MSP-1	2	2	sore throat	No	Unlikely	Mild	Dose not changed	Recovered/ resolved	30/09/2017	.	Sore throat	Oropharyngeal pain	Respiratory, thoracic and mediastinal disorders	3	4	5
447	Caucasian/White	25	1964	53	Female	25 ug MSP-1	3	3	Pain at injection site	No	(Definitely) related	Mild	Dose not changed	Recovered/ resolved	26/10/2017	.	Injection site pain	Injection site pain	General disorders and administration site conditions	0	29	31
448	Caucasian/White	25	1964	53	Female	25 ug MSP-1	5	5	Induration at injection site	No	Probable	Mild	Dose not changed	Recovered/ resolved	24/10/2017	.	Injection site induration	Injection site induration	General disorders and administration site conditions	0	29	29
449	Caucasian/White	25	1964	53	Female	25 ug MSP-1	4	4	Lankopenia	No	Possible	Mild	Dose not changed	Recovered/ resolved	07/11/2017	.	Leucopenia	Leukopenia	Blood and lymphatic system disorders	6	35	43
450	Caucasian/White	25	1964	53	Female	25 ug MSP-1	6	6	muscle pain at injection site	No	(Definitely) related	Mild	Dose not changed	Recovered/ resolved	23/11/2017	.	Injection site muscle pain	Injection site pain	General disorders and administration site conditions	0	57	59
451	Caucasian/White	25	1964	53	Female	25 ug MSP-1	7	7	common cold symptoms	No	Unlikely	Mild	Not applicable	Recovered/ resolved	21/12/2017	.	Common cold	Nasopharyngitis	Infections and infestations	24	81	87
452	Caucasian/White	25	1964	53	Female	25 ug MSP-1	9	9	Induration of injection site	No	(Definitely) related	Mild	Dose not changed	Recovered/ resolved	07/03/2018	.	Injection site induration	Injection site induration	General disorders and administration site conditions	0	163	163
453	Caucasian/White	25	1964	53	Female	25 ug MSP-1	10	10	sore throat	No	(Definitely) related	Mild	Dose not changed	Recovered/ resolved	13/03/2018	.	Sore throat	Oropharyngeal pain	Respiratory, thoracic and mediastinal disorders	2	165	169
454	Caucasian/White	25	1964	53	Female	25 ug MSP-1	11	11	sore throat	No	Possible	Mild	Dose not changed	Recovered/ resolved	31/03/2018	.	Sore throat	Oropharyngeal pain	Respiratory, thoracic and mediastinal disorders	24	187	187

455	Caucasian/White	25	1964	53	Female	25 ug MSP-1	12	12 Muscle pain	No	Unlikely	Moderate	Dose not changed	Recovered/ resolved	15/06/2018	.	Muscle pain	Myalgia	Musculoskeletal and connective tissue disorders	38	201	263
456	Caucasian/White	25	1964	53	Female	25 ug MSP-1	13.01	13 Pain and swelling at joints	No	Unlikely	Moderate	Dose not changed	Recovered/ resolved	15/06/2018	1	Joint swelling	Joint swelling	Musculoskeletal and connective tissue disorders	66	229	263
457	Caucasian/White	25	1964	53	Female	25 ug MSP-1	13.02	13 Pain and swelling at joints	No	Unlikely	Moderate	Dose not changed	Recovered/ resolved	15/06/2018	2	Joint pain	Arthralgia	Musculoskeletal and connective tissue disorders	66	229	263
458	Caucasian/White	26	1962	55	Female	150 ug MSP-1	1	1 pain at injection site	No	Probable	Mild	Dose not changed	Recovered/ resolved	05/10/2017	.	Injection site pain	Injection site pain	General disorders and administration site conditions	2	3	9
459	Caucasian/White	26	1962	55	Female	150 ug MSP-1	3	3 Headache	No	Unlikely	Mild	Dose not changed	Recovered/ resolved	18/10/2017	.	Headache	Headache	Nervous system disorders	21	22	22
460	Caucasian/White	26	1962	55	Female	150 ug MSP-1	2	2 intermittent, swolen lymphatic nodes, indolent, cervical left side	No	Unlikely	Mild	Dose not changed	Recovered/ resolved	18/12/2017	.	Swollen lymph nodes	Lymphadenopathy	Blood and lymphatic system disorders	0	57	83
461	Caucasian/White	26	1962	55	Female	150 ug MSP-1	4	4 Urinary tract infection	No	Not relatec	Moderate	Dose not changed	Recovered/ resolved	01/01/2018	.	Urinary tract infection	Urinary tract infection	Infections and infestations	40	97	97
462	Caucasian/White	26	1962	55	Female	150 ug MSP-1	5	5 Swollen lymphatic nodule cervical left side, indolent	No	Unlikely	Mild	Dose not changed	Recovered/ resolved	09/05/2018	.	Lymph nodes cervical swollen	Lymphadenopathy	Blood and lymphatic system disorders	7	204	225
463	Caucasian/White	27	1995	22	Female	150 ug MSP-1	2	2 Headache, intermittent	No	Possible	Mild	Dose not changed	Recovered/ resolved	03/10/2017	.	Headache	Headache	Nervous system disorders	0	1	7
464	Caucasian/White	27	1995	22	Female	150 ug MSP-1	3	3 Fatigue intermittent	No	Possible	Mild	Dose not changed	Recovered/ resolved	01/10/2017	.	Fatigue	Fatigue	General disorders and administration site conditions	0	1	5
465	Caucasian/White	27	1995	22	Female	150 ug MSP-1	1	1 Pain at injection site	No	(Definitely) related	Mild	Dose not changed	Recovered/ resolved	01/10/2017	.	Injection site pain	Injection site pain	General disorders and administration site conditions	1	2	5
466	Caucasian/White	27	1995	22	Female	150 ug MSP-1	4	4 Lymphocyte count decreased	No	(Definitely) related	Mild	Dose not changed	Recovered/ resolved	24/10/2017	.	Lymphocyte count decreased	Lymphocyte count decreased	Investigations	2	3	28
467	Caucasian/White	27	1995	22	Female	150 ug MSP-1	5	5 Myopathy at injection site	No	Probable	Mild	Dose not changed	Recovered/ resolved	30/09/2017	.	Myopathy	Myopathy	Musculoskeletal and connective tissue disorders	2	3	4
468	Caucasian/White	27	1995	22	Female	150 ug MSP-1	6	6 Warmth at injection site	No	(Definitely) related	Mild	Dose not changed	Recovered/ resolved	30/09/2017	.	Injection site warmth	Injection site warmth	General disorders and administration site conditions	2	3	4
469	Caucasian/White	27	1995	22	Female	150 ug MSP-1	12	12 sweating	No	Possible	Mild	Dose not changed	Recovered/ resolved	30/09/2017	.	Sweating	Hyperhidrosis	Skin and subcutaneous tissue disorders	3	4	4
470	Caucasian/White	27	1995	22	Female	150 ug MSP-1	7	7 Medical palpebral inflamation on the left side	No	Not relatec	Mild	Dose not changed	Recovered/ resolved	31/10/2017	.	Inflammation of eyelids	Blepharitis	Eye disorders	27	28	35
471	Caucasian/White	27	1995	22	Female	150 ug MSP-1	8	8 Pain at injection site	No	(Definitely) related	Mild	Dose not changed	Recovered/ resolved	02/11/2017	.	Injection site pain	Injection site pain	General disorders and administration site conditions	1	30	37
472	Caucasian/White	27	1995	22	Female	150 ug MSP-1	9	9 fatigue	No	Probable	Mild	Dose not changed	Recovered/ resolved	26/10/2017	.	Fatigue	Fatigue	General disorders and administration site conditions	1	30	30
473	Caucasian/White	27	1995	22	Female	150 ug MSP-1	10	10 cephalgie 20:00 Uhr	No	Probable	Mild	Dose not changed	Recovered/ resolved	26/10/2017	.	Cephalgia	Headache	Nervous system disorders	1	30	30
474	Caucasian/White	27	1995	22	Female	150 ug MSP-1	11	11 CRP increase	No	(Definitely) related	Mild	Dose not changed	Recovered/ resolved	02/11/2017	.	C-reactive protein increased	C-reactive protein increased	Investigations	2	31	37
475	Caucasian/White	27	1995	22	Female	150 ug MSP-1	16.01	16 Headache / head contusion after swimming pool accident	No	Not relatec	Mild	Dose not changed	Recovered/ resolved	04/11/2017	1	Headache	Headache	Nervous system disorders	9	38	39
476	Caucasian/White	27	1995	22	Female	150 ug MSP-1	16.02	16 Headache / head contusion after swimming pool accident	No	Not relatec	Mild	Dose not changed	Recovered/ resolved	04/11/2017	2	Accident	Accident	Injury, poisoning and procedural complications	9	38	39
477	Caucasian/White	27	1995	22	Female	150 ug MSP-1	13	13 white bloodcell count decreased	No	Possible	Mild	Dose not changed	Recovered/ resolved	24/11/2017	.	White blood cell count decreased	White blood cell count decreased	Investigations	27	56	59
478	Caucasian/White	27	1995	22	Female	150 ug MSP-1	17	17 Erythema close to injection site	No	(Definitely) related	Mild	Dose not changed	Recovered/ resolved	25/11/2017	.	Injection site erythema	Injection site erythema	General disorders and administration site conditions	0	57	60
479	Caucasian/White	27	1995	22	Female	150 ug MSP-1	18	18 Pain at injection site	No	(Definitely) related	Mild	Dose not changed	Recovered/ resolved	24/11/2017	.	Injection site pain	Injection site pain	General disorders and administration site conditions	1	58	59
480	Caucasian/White	27	1995	22	Female	150 ug MSP-1	19	19 CRP increased	No	Possible	Mild	Dose not changed	Recovered/ resolved	28/11/2017	.	CRP increased	C-reactive protein increased	Investigations	2	59	63
481	Caucasian/White	27	1995	22	Female	150 ug MSP-1	14	14 Headache	No	Possible	Mild	Not applicable	Recovered/ resolved	26/11/2017	.	Headache	Headache	Nervous system disorders	4	61	61
482	Caucasian/White	27	1995	22	Female	150 ug MSP-1	15	15 Flu like symptoms	No	Unlikely	Moderate	Dose not changed	Recovered/ resolved	12/12/2017	.	Flu like symptoms	Influenza like illness	General disorders and administration site conditions	12	69	77
483	Caucasian/White	27	1995	22	Female	150 ug MSP-1	20	20 Headache, intermittent	No	Unlikely	Mild	Dose not changed	Recovered/ resolved	11/12/2017	.	Headache	Headache	Nervous system disorders	14	71	76
484	Caucasian/White	27	1995	22	Female	150 ug MSP-1	21.01	21 Hematoma of the left knee (due to by bicycle accident)	No	Not relatec	Mild	Not applicable	Recovered/ resolved	20/12/2017	1	Hematoma	Haematoma	Vascular disorders	21	78	85
485	Caucasian/White	27	1995	22	Female	150 ug MSP-1	21.02	21 Hematoma of the left knee (due to by bicycle accident)	No	Not relatec	Mild	Not applicable	Recovered/ resolved	20/12/2017	2	Cycling accident	Road traffic accident	Injury, poisoning and procedural complications	21	78	85
486	Caucasian/White	27	1995	22	Female	150 ug MSP-1	22	22 white blood cell count decreased	No	Unlikely	Mild	Not applicable	Not recovered/ not resolved	.	.	White blood cell count decreased	White blood cell count decreased	Investigations	26	83	.
487	Caucasian/White	28	1998	19	Male	25 ug MSP-1	1	1 pain at injection site	No	(Definitely) related	Mild	Dose not changed	Recovered/ resolved	28/09/2017	.	Injection site pain	Injection site pain	General disorders and administration site conditions	0	1	2
488	Caucasian/White	28	1998	19	Male	25 ug MSP-1	2	2 sweating	No	Unlikely	Moderate	Dose not changed	Recovered/ resolved	04/11/2017	.	Sweating	Hyperhidrosis	Skin and subcutaneous tissue disorders	6	35	39
489	Caucasian/White	28	1998	19	Male	25 ug MSP-1	3	3 headache	No	Unlikely	Mild	Dose not changed	Recovered/ resolved	04/11/2017	.	Headache	Headache	Nervous system disorders	6	35	39
490	Caucasian/White	28	1998	19	Male	25 ug MSP-1	4	4 common cold	No	Unlikely	Moderate	Dose not changed	Recovered/ resolved	21/11/2017	.	Common cold	Nasopharyngitis	Infections and infestations	6	35	56
491	Caucasian/White	28	1998	19	Male	25 ug MSP-1	5	5 Dizziness	No	Unlikely	Mild	Dose not changed	Recovered/ resolved	05/11/2017	.	Dizziness	Dizziness	Nervous system disorders	6	35	40
492	Caucasian/White	28	1998	19	Male	25 ug MSP-1	6	6 Fever	No	Unlikely	Mild	Dose not changed	Recovered/ resolved	01/11/2017	.	Fever	Pyrexia	General disorders and administration site conditions	7	36	36
493	Caucasian/White	28	1998	19	Male	25 ug MSP-1	7	7 upper respiratory tract infection	No	Not relatec	Moderate	Dose not changed	Recovered/ resolved	04/03/2018	.	Upper respiratory tract infection	Upper respiratory tract infection	Infections and infestations	102	159	159
494	Caucasian/White	28	1998	19	Male	25 ug MSP-1	8	8 pain at injection site	No	(Definitely) related	Mild	Not applicable	Recovered/ resolved	23/03/2018	.	Injection site pain	Injection site pain	General disorders and administration site conditions	1	177	178
495	Caucasian/White	29	1994	23	Male	150 ug MSP-1	1	1 Pain at injection site	No	(Definitely) related	Mild	Dose not changed	Recovered/ resolved	27/09/2017	.	Injection site pain	Injection site pain	General disorders and administration site conditions	0	1	1
496	Caucasian/White	29	1994	23	Male	150 ug MSP-1	2	2 feeling of warmth (whole body)	No	(Definitely) related	Mild	Dose not changed	Recovered/ resolved	27/09/2017	.	Feeling of warmth	Feeling hot	General disorders and administration site conditions	0	1	1
497	Caucasian/White	29	1994	23	Male	150 ug MSP-1	3	3 Pain at injection site	No	(Definitely) related	Mild	Dose not changed	Recovered/ resolved	26/10/2017	.	Injection site pain	Injection site pain	General disorders and administration site conditions	0	29	30
498	Caucasian/White	29	1994	23	Male	150 ug MSP-1	4	4 increased crp	No	(Definitely) related	Mild	Dose not changed	Recovered/ resolved	02/11/2017	.	CRP increased	C-reactive protein increased	Investigations	2	31	37
499	Caucasian/White	29	1994	23	Male	150 ug MSP-1	5	5 Induration at injection site	No	Probable	Mild	Dose not changed	Recovered/ resolved	27/11/2017	.	Injection site induration	Injection site induration	General disorders and administration site conditions	1	58	62
500	Caucasian/White	29	1994	23	Male	150 ug MSP-1	6	6 Hematome at injection site	No	(Definitely) related	Mild	Dose not changed	Recovered/ resolved	29/11/2017	.	Hematoma injection site	Injection site haematoma	General disorders and administration site conditions	5	62	64
501	Caucasian/White	29	1994	23	Male	150 ug MSP-1	7	7 common cold	No	Possible	Mild	Not applicable	Recovered/ resolved	30/11/2017	.	Common cold	Nasopharyngitis	Infections and infestations	7	64	65
502	Caucasian/White	29	1994	23	Male	150 ug MSP-1	10	10 Warmth at injection site	No	(Definitely) related	Mild	Not applicable	Recovered/ resolved	21/03/2018	.	Injection site warmth	Injection site warmth	General disorders and administration site conditions	0	176	176
503	Caucasian/White	29	1994	23	Male	150 ug MSP-1	8	8 Pain at injection site	No	(Definitely) related	Mild	Not applicable	Recovered/ resolved	22/03/2018	.	Injection site pain	Injection site pain	General disorders and administration site conditions	1	177	177
504	Caucasian/White	29	1994	23	Male	150 ug MSP-1	9	9 Blood Bilirubin increased	No	Possible	Mild	Not applicable	Recovered/ resolved	18/04/2018	.	Blood bilirubin increased	Blood bilirubin increased	Investigations	14	190	204
505	Caucasian/White	29	1994	23	Male	150 ug MSP-1	11	11 traumatic Tandonrupture	No	Not relatec	Severe	Not applicable	Recovered/ resolved	30/05/2018	.	Tendon rupture	Tendon rupture	Injury, poisoning and procedural complications	50	226	246
506	Caucasian/White	30	1992	25	Female	25 ug MSP-1	1	1 Pain at injection site	No	(Definitely) related	Mild	Dose not changed	Recovered/ resolved	07/10/2017	.	Injection site pain	Injection site pain	General disorders and administration site conditions	1	2	4
507	Caucasian/White	30	1992	25	Female	25 ug MSP-1	2	2 Vomiting	No	Unlikely	Mild	Dose not changed	Recovered/ resolved	05/10/2017	.	Vomiting	Vomiting	Gastrointestinal disorders	1	2	2
508	Caucasian/White	30	1992	25	Female	25 ug MSP-1	3	3 Pain at injection site	No	(Definitely) related	Mild	Dose not changed	Recovered/ resolved	05/11/2017	.	Injection site pain	Injection site pain	General disorders and administration site conditions	0	30	33
509	Caucasian/White	30	1992	25	Female	25 ug MSP-1	5	5 Warmth at injection site, intermittent	No	(Definitely) related	Mild	Dose not changed	Recovered/ resolved	09/11/2017	.	Injection site warmth	Injection site warmth	General disorders and administration site conditions	0	30	37
510	Caucasian/White	30	1992	25	Female	25 ug MSP-1	9	9 Erythema at injection site	No	(Definitely) related	Mild	Dose not changed	Recovered/ resolved	02/11/2017	.	Injection site erythema	Injection site erythema	General disorders and administration site conditions	0	30	30
511	Caucasian/White	30	1992	25	Female	25 ug MSP-1	4	4 haematome at injection site	No	(Definitely) related	Mild	Dose not changed	Recovered/ resolved	12/11/2017	.	Haematoma injection site	Injection site haematoma	General disorders and administration site conditions	1	31	40
512	Caucasian/White	30	1992	25	Female	25 ug MSP-1	6	6 CRP elevation	No	Possible	Mild	Dose not changed	Recovered/ resolved	09/11/2017	.	CRP increased	C-reactive protein increased	Investigations	2	32	37
513	Caucasian/White	30	1992	25	Female	25 ug MSP-1	8	8 common cold	No	Possible	Mild	Dose not changed	Recovered/ resolved	16/11/2017	.	Common cold	Nasopharyngitis	Infections and infestations	3	33	44
514	Caucasian/White	30	1992	25	Female	25 ug MSP-1	7	7 Exanthema in both forearms	No	Unlikely	Mild	Dose not changed	Recovered/ resolved	08/11/2017	.	Exanthema	Rash	Skin and subcutaneous tissue disorders	4	34	36
515	Caucasian/White	30	1992	25	Female	25 ug MSP-1	10	10 headache	No	Unlikely	Moderate	Dose not changed	Recovered/ resolved	16/11/2017	.	Headache	Headache	Nervous system disorders	13	43	44
516	Caucasian/White	30	1992	25	Female	25 ug MSP-1	11	11 warmth at injection site	No	(Definitely) related	Mild	Dose not changed	Recovered/ resolved	01/12/2017	.	Injection site warmth	Injection site warmth	General disorders and administration site conditions	1	58	59
517	Caucasian/White	30	1992	25	Female	25 ug MSP-1	12	12 Pain at injection site	No	(Definitely) related	Mild	Dose not changed	Recovered/ resolved	01/12/2017	.	Injection site pain	Injection site pain	General disorders and administration site conditions	1	58	59
518	Caucasian/White	30	1992	25	Female	25 ug MSP-1	13	13 Uricaria	No	Not relatec	Moderate	Dose not changed	Recovered/ resolved	01/01/2018	.	Urticaria	Urticaria	Skin and subcutaneous tissue disorders	33	90	90
519	Caucasian/White	30	1992	25	Female	25 ug MSP-1	14	14 Contact Dermatitis	No	Not relatec	Mild	Not applicable	Recovered/ resolved	13/04/2018	.	Contact dermatitis	Dermatitis contact	Skin and subcutaneous tissue disorders	1	191	192
520	Caucasian/White	30	1992	25	Female	25 ug MSP-1	15	15 pain at injection site	No	(Definitely) related	Mild	Not applicable	Recovered/ resolved	13/04/2018	.	Injection site pain	Injection site pain	General disorders and administration site conditions	1	191	192
521	Caucasian/White	30	1992	25	Female	25 ug MSP-1	16	16 Erythema at i.v. catheter site due to adhesive bondage	No	Not relatec	Mild	Not applicable	Recovered/ resolved	13/04/2018	.	Catheter site erythema	Catheter site erythema	General disorders and administration site conditions	1	191	192
522	Caucasian/White	31	1995	22	Female	25 ug MSP-1	1	1 common cold	No	Probable	Mild	Dose not changed	Recovered/ resolved	06/10/2017	.	Common cold	Nasopharyngitis	Infections and infestations	1	2	3
523	Caucasian/White	31	1995	22	Female	25 ug MSP-1	5	5 headache, intermittent	No	Possible	Mild	Dose not changed	Recovered/ resolved	16/10/2017	.	Headache	Headache	Nervous system disorders	2	3	13
524	Caucasian/White	31	1995	22	Female	25 ug MSP-1	6	6 headache	No	Possible	Mild	Dose not changed	Recovered/ resolved	24/10/2017	.	Headache	Headache	Nervous system disorders	20	21	21
525	Caucasian/White	31	1995	22	Female	25 ug MSP-1	2	2 haematoma at injection site	No	(Definitely) related	Mild	Dose not changed	Recovered/ resolved	08/11/2017	.	Haematoma injection site	Injection site haematoma	General disorders and administration site conditions	0	30	36
526	Caucasian/White	31	1995	22	Female	25 ug MSP-1	3	3 warmth at injection site	No	(Definitely) related	Mild	Dose not changed	Recovered/ resolved	08/11/2017	.	Injection site warmth	Injection site warmth	General disorders and administration site conditions	0	30	36
527	Caucasian/White	31	1995	22	Female	25 ug MSP-1	4	4 pain at injection site	No	(Definitely) related	Mild	Dose not changed	Recovered/ resolved	08/11/2017	.	Injection site pain	Injection site pain	General disorders and administration site conditions	0	30	36

528	Caucasian/White	31	1995	22	Female	25 ug MSP-1	7	7	common cold	No	Not related	Mild	Dose not changed	Recovered/ resolved	27/11/2017	.	Common cold	Nasopharyngitis	Infections and infestations	18	48	55
529	Caucasian/White	31	1995	22	Female	25 ug MSP-1	10	10	pain at injection site	No	(Definitely) related	Mild	Not applicable	Recovered/ resolved	01/12/2017	.	Injection site pain	Injection site pain	General disorders and administration site conditions	0	57	59
530	Caucasian/White	31	1995	22	Female	25 ug MSP-1	8	8	warmth at injection site	No	(Definitely) related	Mild	Dose not changed	Recovered/ resolved	06/12/2017	.	Injection site warmth	Injection site warmth	General disorders and administration site conditions	1	58	64
531	Caucasian/White	31	1995	22	Female	25 ug MSP-1	9	9	induration at injection site	No	(Definitely) related	Mild	Dose not changed	Recovered/ resolved	01/12/2017	.	Injection site induration	Injection site induration	General disorders and administration site conditions	1	58	59
532	Caucasian/White	31	1995	22	Female	25 ug MSP-1	11	11	Myalgia at upper arm, left	No	(Definitely) related	Mild	Dose not changed	Recovered/ resolved	01/12/2017	.	Myalgia upper extremities	Myalgia	Musculoskeletal and connective tissue disorders	1	58	59
533	Caucasian/White	31	1995	22	Female	25 ug MSP-1	12	12	Headache	No	Unlikely	Mild	Not applicable	Recovered/ resolved	13/12/2017	.	Headache	Headache	Nervous system disorders	14	71	71
534	Caucasian/White	31	1995	22	Female	25 ug MSP-1	13	13	Headache	No	Unlikely	Moderate	Not applicable	Recovered/ resolved	20/12/2017	.	Headache	Headache	Nervous system disorders	20	77	78
535	Caucasian/White	31	1995	22	Female	25 ug MSP-1	14	14	Pain at injection site	No	(Definitely) related	Moderate	Not applicable	Recovered/ resolved	23/03/2018	.	Injection site pain	Injection site pain	General disorders and administration site conditions	1	170	171
536	Caucasian/White	31	1995	22	Female	25 ug MSP-1	15	15	CRP incraesed	No	(Definitely) related	Moderate	Not applicable	Recovered/ resolved	28/03/2018	.	C-reactive protein increased	C-reactive protein increased	Investigations	2	171	176
537	Caucasian/White	31	1995	22	Female	25 ug MSP-1	16	16	Lymphopenia	No	Possible	Mild	Not applicable	Recovered/ resolved	28/03/2018	.	Lymphopenia	Lymphopenia	Blood and lymphatic system disorders	2	171	176
538	Caucasian/White	31	1995	22	Female	25 ug MSP-1	17	17	Ear pain, left	No	Unlikely	Mild	Not applicable	Recovered/ resolved	27/03/2018	.	Ear pain	Ear pain	Ear and labyrinth disorders	5	174	175
539	Caucasian/White	31	1995	22	Female	25 ug MSP-1	18	18	Headache	No	Possible	Mild	Not applicable	Recovered/ resolved	27/03/2018	.	Headache	Headache	Nervous system disorders	5	174	175
540	Caucasian/White	31	1995	22	Female	25 ug MSP-1	19	19	Gastroenteritis	No	Unlikely	Mild	Not applicable	Recovered/ resolved	07/04/2018	.	Gastroenteritis	Gastroenteritis	Infections and infestations	13	182	186
541	Caucasian/White	31	1995	22	Female	25 ug MSP-1	20	20	Lymphopenia	No	Possible	Mild	Not applicable	Recovered/ resolved	18/04/2018	.	Lymphopenia	Lymphopenia	Blood and lymphatic system disorders	14	183	197
542	Caucasian/White	31	1995	22	Female	25 ug MSP-1	21	21	Headache	No	Possible	Mild	Not applicable	Recovered/ resolved	17/04/2018	.	Headache	Headache	Nervous system disorders	27	196	196
543	Caucasian/White	32	1996	21	Female	150 ug MSP-1	1	1	pain at injection site	No	(Definitely) related	Mild	Dose not changed	Recovered/ resolved	07/10/2017	.	Injection site pain	Injection site pain	General disorders and administration site conditions	0	1	4
544	Caucasian/White	32	1996	21	Female	150 ug MSP-1	3	3	fatigue	No	Probable	Mild	Dose not changed	Recovered/ resolved	05/10/2017	.	Fatigue	Fatigue	General disorders and administration site conditions	0	1	2
545	Caucasian/White	32	1996	21	Female	150 ug MSP-1	5	5	cephalgie	No	Probable	Mild	Dose not changed	Recovered/ resolved	05/10/2017	.	Cephalgia	Headache	Nervous system disorders	0	1	2
546	Caucasian/White	32	1996	21	Female	150 ug MSP-1	6	6	sweating	No	Probable	Mild	Dose not changed	Recovered/ resolved	05/10/2017	.	Sweating	Hyperhidrosis	Skin and subcutaneous tissue disorders	0	1	2
547	Caucasian/White	32	1996	21	Female	150 ug MSP-1	2	2	warmth at injection site	No	Probable	Mild	Dose not changed	Recovered/ resolved	07/10/2017	.	Injection site warmth	Injection site warmth	General disorders and administration site conditions	2	3	4
548	Caucasian/White	32	1996	21	Female	150 ug MSP-1	4	4	hematoma at injection site	No	Probable	Mild	Dose not changed	Recovered/ resolved	13/10/2017	.	Hematoma injection site	Injection site haematoma	General disorders and administration site conditions	5	6	10
549	Caucasian/White	32	1996	21	Female	150 ug MSP-1	7	7	pain at injection site	No	(Definitely) related	Mild	Dose not changed	Recovered/ resolved	08/11/2017	.	Injection site pain	Injection site pain	General disorders and administration site conditions	1	31	36
550	Caucasian/White	32	1996	21	Female	150 ug MSP-1	8	8	swelling at injection site	No	(Definitely) related	Mild	Dose not changed	Recovered/ resolved	04/11/2017	.	Injection site swelling	Injection site swelling	General disorders and administration site conditions	1	31	32
551	Caucasian/White	32	1996	21	Female	150 ug MSP-1	9	9	pruritus at injection site	No	(Definitely) related	Mild	Dose not changed	Recovered/ resolved	04/11/2017	.	Injection site pruritus	Injection site pruritus	General disorders and administration site conditions	1	31	32
552	Caucasian/White	32	1996	21	Female	150 ug MSP-1	10	10	warmth at injection site	No	(Definitely) related	Mild	Dose not changed	Recovered/ resolved	08/11/2017	.	Injection site warmth	Injection site warmth	General disorders and administration site conditions	1	31	36
553	Caucasian/White	32	1996	21	Female	150 ug MSP-1	11	11	Erythema at injection site	No	(Definitely) related	Mild	Dose not changed	Recovered/ resolved	08/11/2017	.	Injection site erythema	Injection site erythema	General disorders and administration site conditions	2	32	36
554	Caucasian/White	32	1996	21	Female	150 ug MSP-1	12	12	Rash (sural, both sides)	No	Possible	Mild	Dose not changed	Recovered/ resolved	06/11/2017	.	Rash both legs	Rash	Skin and subcutaneous tissue disorders	2	32	34
555	Caucasian/White	32	1996	21	Female	150 ug MSP-1	13	13	warmth at injection site	No	(Definitely) related	Mild	Dose not changed	Recovered/ resolved	29/11/2017	.	Injection site warmth	Injection site warmth	General disorders and administration site conditions	0	57	57
556	Caucasian/White	32	1996	21	Female	150 ug MSP-1	14	14	Abdominal pain	No	Unlikely	Mild	Dose not changed	Recovered/ resolved	07/12/2017	.	Abdominal pain	Abdominal pain	Gastrointestinal disorders	7	64	65
557	Caucasian/White	32	1996	21	Female	150 ug MSP-1	15	15	Nausea	No	Unlikely	Mild	Dose not changed	Recovered/ resolved	07/12/2017	.	Nausea	Nausea	Gastrointestinal disorders	7	64	65
558	Caucasian/White	32	1996	21	Female	150 ug MSP-1	16	16	Diarrhea	No	Unlikely	Mild	Dose not changed	Recovered/ resolved	07/12/2017	.	Diarrhea	Diarrhoea	Gastrointestinal disorders	7	64	65
559	Caucasian/White	32	1996	21	Female	150 ug MSP-1	17	17	Hematoma at place of i.v. catheter left	No	Not related	Mild	Not applicable	Recovered/ resolved	18/04/2018	.	Catheter site haematoma	Catheter site haematoma	General disorders and administration site conditions	1	191	197
560	Caucasian/White	32	1996	21	Female	150 ug MSP-1	19	19	CRP increased	No	Possible	Mild	Not applicable	Recovered/ resolved	25/04/2018	.	CRP increased	C-reactive protein increased	Investigations	2	192	204
561	Caucasian/White	32	1996	21	Female	150 ug MSP-1	18	18	Inversion ankl sprain left	No	Not related	Mild	Not applicable	Recovering/ resolving	30/11/2018	.	Ankle sprain	Ligament sprain	Injury, poisoning and procedural complications	4	194	423
562	Caucasian/White	32	1996	21	Female	150 ug MSP-1	20	20	Common cold symptoms	No	Possible	Mild	Not applicable	Recovered/ resolved	13/05/2018	.	Common cold	Nasopharyngitis	Infections and infestations	25	215	222

Variables in Creation Order			
#	Variable	Type	Label
1	ethnic	Char	
2	STUDYID	Char	Study ID
3	SITEID	Char	Currently treating site
4	USUBJID	Num	Participant ID
5	BRTHDTC	Char	Year of birth
6	AGE	Num	Age at day of inclusion
7	SEX	Num	Gender
8	armcd	Num	Treatment group
16	AESEQ	Num	AE No. (unified per patient)
17	AESPID	Num	AE CRF No
18	AETERM	Char	Description of AE (only one finding per row)
19	AESER	Num	Serious?
20	AEREL	Num	Relatedness
22	AESEV	Num	CTCAE-grade
23	AEINTER	Num	Intermittent
24	AEACN	Num	Action taken
25	AEOUT	Num	Outcome
30	Split_No_	Num	Split No#
31	LLT	Num	LLT
32	PT	Num	PT
33	SOC	Num	SOC
34	LLT_Coded_Term	Char	LLT_Coded_Term
35	PT_Coded_Term	Char	PT_Coded_Term
36	SOC_Coded_Term	Char	SOC_Coded_Term
49	aedosdur	Num	
50	aestdy	Num	Study Day of start of AE
51	aendy	Num	Study Day of end of AE

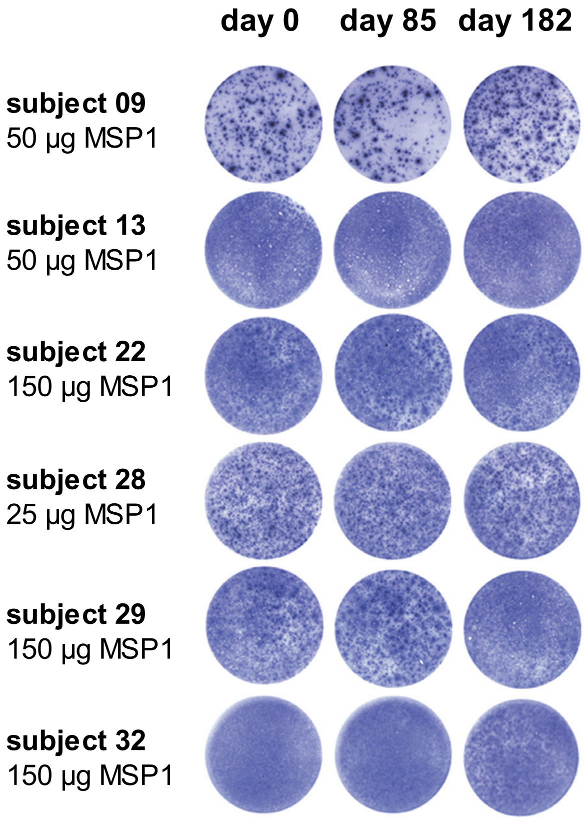

Supplementary Fig. 1 PBMCs from six volunteers immunized three times with SumayaVac-1 were stimulated with a monoclonal antibody against CD3 in the cultured ELISpot assay.
